# SFRP1 is a possible candidate for epigenetic therapy in non-small cell lung cancer

**DOI:** 10.1186/s12920-016-0196-3

**Published:** 2016-08-12

**Authors:** Y-h. Taguchi, Mitsuo Iwadate, Hideaki Umeyama

**Affiliations:** 1 Department of Physics, Chuo University, 1-13-27 Kasuga, Bunkyo-ku, 112-8551 Tokyo, Japan; 2 Department of Biological Science, Chuo University, 1-13-27 Kasuga, Bunkyo-ku, 112-8551 Tokyo, Japan

## Abstract

**Background:**

Non-small cell lung cancer (NSCLC) remains a lethal disease despite many proposed treatments. Recent studies have indicated that epigenetic therapy, which targets epigenetic effects, might be a new therapeutic methodology for NSCLC. However, it is not clear which objects (e.g., genes) this treatment specifically targets. Secreted frizzled-related proteins (SFRPs) are promising candidates for epigenetic therapy in many cancers, but there have been no reports of SFRPs targeted by epigenetic therapy for NSCLC.

**Methods:**

This study performed a meta-analysis of reprogrammed NSCLC cell lines instead of the direct examination of epigenetic therapy treatment to identify epigenetic therapy targets. In addition, mRNA expression/promoter methylation profiles were processed by recently proposed principal component analysis based unsupervised feature extraction and categorical regression analysis based feature extraction.

**Results:**

The Wnt/β-catenin signalling pathway was extensively enriched among 32 genes identified by feature extraction. Among the genes identified, SFRP1 was specifically indicated to target β-catenin, and thus might be targeted by epigenetic therapy in NSCLC cell lines. A histone deacetylase inhibitor might reactivate SFRP1 based upon the re-analysis of a public domain data set. Numerical computation validated the binding of SFRP1 to WNT1 to suppress Wnt signalling pathway activation in NSCLC.

**Conclusions:**

The meta-analysis of reprogrammed NSCLC cell lines identified SFRP1 as a promising target of epigenetic therapy for NSCLC.

**Electronic supplementary material:**

The online version of this article (doi:10.1186/s12920-016-0196-3) contains supplementary material, which is available to authorized users.

## Background

Non-small cell lung cancer (NSCLC) is still lethal despite many proposed therapeutic strategies. Among the many alternative strategies, epigenetic therapy is regarded as a promising method [1], and a histone deacetylase (HDAC) inhibitor [2] or DNA methyltransferase inhibitor [3] were shown to be promising NSCLC treatments, especially when combined [1]. There has been extensive research regarding the clinical usefulness of epigenetic therapy for NSCLC; however, studies investigating the target genes of these treatments are limited, although some promising candidates have been proposed [4]. The potential reasons for the small number of epigenetic therapy target gene reports might be the difficulty of in vitro studies [5]. Compared with many clinical studies regarding the efficiency of epigenetic therapy, there have been few in vitro studies of epigenetic therapy [6, 7]. Thus, alternative strategies to direct in vitro experiments for epigenetic therapy such as the investigation of reprogrammed cancer cell lines are required to investigate the effect of epigenetic therapy in NSCLC.

It is thought that epigenetic therapy targets epigenetic effects, e.g., DNA methylation and/or histone modification, which might be affected by reprogramming. Thus, a detailed and extensive comparative study might indirectly identify the effect of epigenetic therapy in NSCLC cell lines.

This study performed a meta-analysis of reprogrammed NSCLC cell lines to identify genes associated with epigenetic alterations and expression changes during reprogramming and to identify promising candidate genes for targets of epigenetic therapy. Among those identified, secreted frizzled-related protein (SFRP)1 was of interest. Using in vitro epigenetic therapy experiments, we confirmed that SFRP1 mRNA expression and its histone modification were altered. Furthermore, SFRP1 might suppress the Wnt signalling pathway by binding to Wnt genes. An *in silico* study indicated the potential binding of SFRP1 with WNT1; thus, the reactivation of SFRP1 suppressed in NSCLC might be a promising candidate target for the epigenetic therapy of NSCLC.

## Results

### Identification of biologically significant genes

To identify genes targeted by epigenetic therapy in NSCLC, we analysed gene expression and promoter methylation in reprogrammed NSCLC cell lines [8]. Although it is useful to consider histone modification and promoter methylation together because epigenetic therapies targets both, suitable data sets were not publically available for histone modification; therefore, as promoter methylation often reflects the effect of histone modification [9], a data set containing gene expression and promoter methylation information was analysed. The primary aim of this analysis was to identify genes associated with aberrant gene expression and promoter methylation during reprogramming because associated genes are most likely targeted by epigenetic therapy.

Although promoter methylation was generally expected to be negatively correlated with gene expression, this was not always observed, especially when histone modification was also considered [10]. Because this study aimed to identify targets of epigenetic therapy including both DNA methylation and histone modification, we did not restrict candidate biologically significant genes such as those associated with negative correlations between promoter methylation and gene expression, but considered all genes associated with significant correlations between promoter methylation and gene expression independent of the direction.

**Fig. 1:**
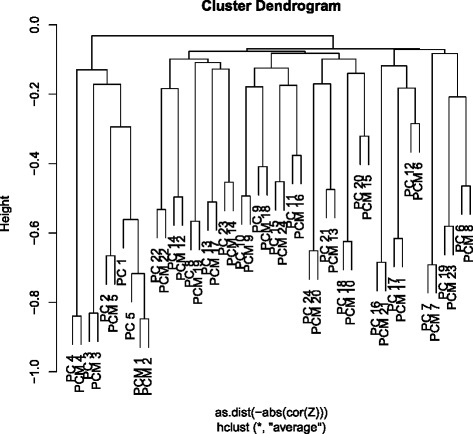
Hierarchical clustering of PCs. Vertical axes represent distance that is negative signed absolute correlation coefficients between PCs. PCs with “M” indicate promoter methylation and those without “M” were computed from gene expression

To select biologically significant genes, we used principal component analysis (PCA) based unsupervised feature extraction (FE) [11–24]. PCA based unsupervised FE is useful when there is no information regarding how to order multiple classes. It also allows us to restrict number of pairs whose correlations must be computed, which can reduce the possibility that selected genes are rejected because of *P*-values adjustments based on multiple comparison correction criteria. Therefore, because many cell lines, including those that were reprogrammed and differentiated, were used in this study, PCA based unsupervised FE was a suitable method for analysis. To select principal components (PCs) with a significant correlation between gene expression and promoter methylation for FE, we performed hierarchical clustering (see Methods) to identify a pair of PCs associated with a high correlation between promoter methylation and gene expression. PC3 and PC4 were the most suitable candidate pairs (Fig. 1).

One may wonder why H1, IMR90, and iPSIMR90 must be included in the analysis. As can be seen in Additional file 1, reprogrammed cell lines have similar values to H1 or iPSIMR90 that are pluripotent; this suggested that the inclusion of H1 and iPSIMR90 guaranteed that cell lines were correctly reprogrammed. Similarly, inclusion of IMR90 guaranteed that reprogrammed cell lines are often distinct from IMR90 that is differentiated. Thus, H1, IMR90, and iPSIMR90 are worthwhile being included.

To determine the stability of pairs of PC3 and PC4 between gene expression and promoter methylation we constructed hierarchical clustering with only 23 samples (because there are 24 samples, there was a sequential removal of one specific sample from the 24 samples; see Methods and full results are Additional file 2). Pairs of PC3 and PC4 between gene expression and promoter methylation were conserved for 22 hierarchical clusters among 24 samples. Thus, the pairs of PC3 and PC4 in Fig. 1 are not accidental but robust.

Although we previously [24] considered only PC1 and PC2 when analysing the same data set for another study, gene expression and promoter methylation showed a lower correlation when compared with PC3 and PC4 in this study. This might explain why promising candidate genes were not identified in our previous study [24]. Therefore, we used PC3 and PC4 for gene selection in this study. In addition to PCA based unsupervised FE, we used another FE that is also suitable for multiclasses that lack a pre-decided order, FE based upon categorical regression (see Methods).

Table 1 summarizes the genes selected by PCA based unsupervised FE and categorical regression based FE. In total, we identified 32 unique gene candidates (three genes were identified by more than one method). Gene expression and promoter methylation of specific PCs and genes and their correlation information is summarized in Table 1 and detailed in Additional files 1, 3, 4 and 5.

**Table 1:** Genes selected by FEs

	(A)	(B)	(C)	(D)		(A)	(B)	(C)	(D)		(A)	(B)	(C)	(D)
Categorical regression					SFRP1	○	○	○	○	LAMC2	○	○	○	○
SALL4	○	○	○	○	SLC16A12					HMGA1	○	○	○	○
TACSTD1*	○	○	○	○	HOXA5	○	○	○	○	LAD1		○	○	
ANGPT1	○		○	○	KIF1A		○	○	○	PFKFB3	○		○	○
TACSTD2*	○	○	○	○	H2AFY	○	○	○		DEFB1	○		○	
IGSF21					ATP5G2					SRGN	○	○	○	
EFNB1	○		○	○	TM4SF1	○	○	○	○	UCHL1	○	○	○	○
MEST	○	○	○	○	GPR56*	○	○	○	○	ALDH3A1	○	○	○	○
SCG3			○		S100P	○	○	○	○	EPB41L3	○		○	○
PCA based unsupervised FE (PC4)					PCA based unsupervised FE (PC4)					RTN1	○	○	○	
F2R	○	○	○	○	SPINT2	○	○	○	○	LAMA1	○		○	○
DKK3	○	○	○	○	CDH1	○	○	○	○					

Genes with asterisk were selected by more than one method

(A) Associations with cancer related genes reported by Gendoo server. (B) Significant negative correlations (*P* < 0.05) between gene expression and promoter methylation. (C) At least one study reported a direct/indirect relationship with NSCLC. (D) At least one study reported a direct/indirect relationship with Wnt/β-catenin signalling pathways. Asterisked three genes are also identified by PCA based unsupervised FE with PC4

### Biological significance of selected genes

#### Disease association of genes

To validate the biological significance of the selected genes, we used the Gendoo server [25] to search the literature for genes associated with diseases. For most of the genes examined (excluding LAD1, KIF1A, SLC16A12, SCG3 and IGSF21), there were significant associations with cancer-related diseases as summarized in Table 1A (and Additional file 6). Many oncogenes and tumor suppressors are not unique for specific cancers but are related to several cancers. This suggested that the genes selected in this study have the potential to be involved with NSCLC tumourigenesis.

#### Pathway/Gene Ontology (GO) term analysis

Because disease association is not always informative regarding how the genes are involved in tumourigenesis, we uploaded a list including gene IDs to two gene annotation servers [26, 27] (Table 2). The selection of “extracellular region” was reasonable, because this is a reprogramming study, thus cell surface receptors should be activated to initiate differentiation, which is related to another selected GO term, “cell proliferation”. When mapping selected genes to the Kyoto Encyclopedia of Genes and Genomes (KEGG) pathway, most were at the cell surface (see Additional file 7).

**Table 2:** Results from various annotation servers

		*P*-value	Number of genes
Targetmine			
GO Term	Extracellular region [GO:0005576]	2.03 × 10^−3^	21
	Lateral plasma membrane [GO:0016328]	8.55 × 10^−3^	3
GOSlim Term	Extracellular region [GO:0005576]	6.43 × 10^−5^	21
	Locomotion [GO:0040011]	1.34 × 10^−2^	9
	Cell adhesion [GO:007155]	1.34 × 10^−2^	7
	Cell junction organisation [GO:0034330]	1.74 × 10^−2^	4
	Anatomical structure development [GO:0048856]	1.76 × 10^−2^	14
g:Profiler			
GO Term	Cell proliferation [GO:0008283]	1.42 × 10^−2^	12
	Regulation of cell proliferation [GO:0042127]	4.83 × 10^−2^	10
	Regulation of cell adhesion [GO:0030155]	1.58 × 10^−2^	6
	Cellular component movement [GO:0006928]	3.67 × 10^−3^	11
	Extracellular region [GO:0005576]	3.94 × 10^−4^	19
TF	PPAR, HNF-4, COUP, PAR [TF:M00762 4]	2.72 × 10^−2^	18

Adjusted *P*-values for target-mining is based on BH criterion

#### Literature search

To determine whether the selected genes were specifically related to NSCLC and pluripotency because the data analysed was from reprogramming experiments, we performed an extensive literature search. Studies regarding the relationship between NSCLC and proteins reported to bind to any of the 32 genes listed in Table 1 were collected and analysed with BioGrid [28], which reports literature-based protein-protein interactions. Most of the genes identified were specifically related to NSCLC tumourigenesis and some were also related to pluripotency (Table 1C and Additional file 8). Thus, this methodology is promising.

## Discussion

### The Wnt/β-catenin signalling pathway as an NSCLC therapy target

Although we found that the genes identified in this study were mostly related to NSCLC tumourigenesis, genes should be selected according to their potential for epigenetic therapy of NSCLC. However, as can be seen in Table 2, no significant pathway enrichments were detected. In order to investigate pathway enrichment, we performed literature searches manually. Then we have found that multiple genes selected in this study were related to the Wnt/β-catenin signalling pathway (Table 1D) that was recently reported to be a major pathway in NSCLC tumourigenesis [29]. One may wonder why Wnt/β-catenin signalling pathway was not detected in enrichment analyses in Table 2. First of all, even if no significant enrichments were detected, it does not always mean the lack of enrichment, but often simply means the lack of ability of the specific statistical tests. Second, as can be seen in the following, some genes detected by literature searches, e.g., EPCAM and TACSTD2, are not included into KEGG pathway. This means that we need more sophisticated investigations than simple enrichment analyses. This is the reason why we additionally performed literature searches.

SALL4 is part of the Wnt signalling pathway [30] and regulates the stemness of EPCAM-positive hepatocellular carcinomas [31, 32]. EPCAM was recently reported to be an endoderm-specific Wnt derepressor [32]. ANGPT1 was reported to be upregulated via the overexpression of β-catenin that is a key factor of the Wnt signalling pathway [33]. TACSTD2 was proposed to be a Wnt target [34] identified through consistent gene expression changes in APC-mutant intestinal adenomas from humans and mice; EFNB1 :Eph-related receptor is a Wnt signalling target gene in colorectal cancer [35] that binds to the EFNB1 ligand. MEST inhibits Wnt signalling through the regulation of LRP6 glycosylation [36]. F2R(PAR1) stabilizes β-catenin in mammary gland tissues [37]. DKK3 binds to LRP5/6 and inhibits the initiation of Wnt signalling [29]. SFRP1 binds to FZD and WNT to suppress the activation of Wnt signalling [29]. HOXA5 expression increased the retention of β-catenin in adherens junctions and reduced permeability [38]. KIF1A binds to at least two β-catenin binding proteins [39], ESR1 [40] and AR [41]. TM4SF1 might have a role in coordinating Wnt signalling and migration during endocrine pancreas specification [42], and TM4SF1 and TM4SF4 belong to the tetraspanin L6 domain family. GPR56: The Wnt/β-catenin signalling pathway regulates genes involved in cell proliferation, survival, migration and invasion through by the regulation of T-cell factor (TCF)-4 transcription factor proteins that activate GPR56 in HCC [43]. S100P: Increased expression of S100P promoted cellular proliferation by increasing the nuclear translocation of β-catenin in endometrial cancer [44]. SPINT2: The epigenetic silencing of SPINT2 promoted cancer cell motility via HGF-MET pathway activation in melanoma [45], and β-catenin formed a complex with c-Met (HGF receptor) [46]. CDH1 (E-cadherin) is involved in the inactivation of Wnt/β-catenin signalling in urothelial carcinoma and normal urothelial cells [47]. LAMC2 (Laminin γ2) mediated the Wnt5a-induced invasion of gastric cancer cells [48]. HMGA1 interacted with β-catenin to positively regulate Wnt/β-catenin signaling in colorectal cancer cells [49]. PFKFB3: The altered expression of PFKFB3 is associated with Wnt signalling pathway genes [50]. UCHL1 is a colorectal cancer oncogene that activated the β-catenin/TCF pathway through its deubiquitinating activity [51]. ALDH3A1 is overexpressed in a subset of hepatocellular carcinoma characterized by activation of the Wnt/β-catenin pathway [52]. EPB41L3 (DAL1) binds to YWHAZ [53], and the YWHAZ/β-catenin axis promoted epithelial-mesenchymal transition and lung cancer metastasis [54]. LAMA1 (laminin): Overexpression of the Wnt antagonist FRZB1 decreased RNA levels of the essential basement membrane genes fibronectin and laminin [55].

### β-catenin is often reported to be related to NSCLC

Although β-catenin is extensively related to the selected genes in this study, β-catenin was overexpressed in NSCLC [56]. β-catenin expression was also prognostic for improved NSCLC survival [57]. Nuclear β-catenin accumulation was associated with the increased expression of NANOG protein and predicted a poor prognosis of NSCLC [58]. Promoter methylation-mediated silencing of β-catenin enhanced the invasiveness of NSCLC and predicted an adverse prognosis [59]. All of these studies strongly suggest the importance of β-catenin in NSCLC.

### These genes are also related to epigenetic therapy

The following genes were also suggested to be related to epigenetic therapy. Recently, the combined usage of two drugs, romidepsin and decitabine, restored SFRP1 activity in four cancer cell lines, A498, KIJ265T, MDA-231, and BT-20 [60]. This strategy might be useful for NSCLC therapy because an HDAC inhibitor, MPT0E028, enhanced erlotinib-induced cell death in epidermal growth factor receptor tyrosine kinase inhibitor (EGFR-TKI)-resistant NSCLC cells [61] and SAHA, a HDAC inhibitor, had profound anti-growth activity against NSCLC cells [62]. Other evidence includes an organosulfur derivative of the HDAC inhibitor, valproic acid, which sensitised human lung cancer cell lines to apoptosis and to cisplatin cytotoxicity [63]. EGFR-TKI resistance by BIM polymorphism was circumvented when combined with HDAC inhibition [64], and antitumour activity of histone deacetylase inhibitors was observed in NSCLC cells [65]. The effect of HDAC inhibitors can be improved by in silico drug screening [66]. In addition, SALL4 was reported to form a protein complex with HDAC [67–69] (Fig. 4a in [69]), which suggests that epigenetic regulation of the Wnt signalling pathway is a key factor in the tumourigenesis of NSCLC. Interestingly, promoters of SALL4 and SFRP1 were methylated simultaneously [70–72]. Although there have been no reports to suggest a direct relationship between HOXA5 and the Wnt signalling pathway in NSCLC, HOX is related to the Wnt signalling pathway, which controls HOX gene expression in *C. elegans* [73], while HOX genes control Wnt signalling [74]. Furthermore, WNT7A has a strong relationship with HOX genes [75]. In addition, from an evolutionary point of view, HOX and Wnt might be related [76]. Thus, HOXA5 might be involved in Wnt signalling in NSCLC and might also be influenced by HDAC [77].

### SFRP1 is a potential epigenetic therapy target

Overall, we concluded that the Wnt signalling pathway is a likely target of epigenetic therapy in NSCLC cell lines. A previous study suggested that the reactivation of Wnt antagonists, including DKK3 and SFRP1, in NSCLC might be a beneficial epigenetic therapy [78]. Among the genes potentially related to Wnt signalling, we considered SFRP1 to be the most promising candidate gene targeted by epigenetic treatment. A previous study reported that treatment with 5-aza-2’-deoxycytidine, a DNA methyltransferase inhibitor, enhanced SFRP1 expression in NSCLC [79]. Transcriptional silencing of the gene was also due to hypermethylation of its promoter region in NSCLC [80]. SFRP1 has been reported as a marker for NSCLC [81, 82]. In addition, SFRP1 was also reported to be related to β-catenin. For example, SFRP1, SFRP2, and SFRP5 regulate Wnt/β-catenin and planar cell polarity pathways during early trunk formation in mice [83]. Loss of SFRP1 expression was associated with aberrant β-catenin distribution and tumor progression in mucoepidermoid carcinoma of salivary glands [84].

To confirm whether the HDAC inhibitor affected SFRP1 regulation in NSCLC, we analysed a public domain data set. Miyanaga et al. [65] compared various cell lines to determine whether they were resistant to HDAC inhibitors. We investigated SFRP1 expression between HDAC inhibitor-resistant cell lines and non-resistant cell lines for adenocarcinoma and squamous cell carcinoma and found different levels of SFRP1 expression (Table 3). SFRP1 expression was upregulated in non-resistant cell lines compared with resistant cell lines because cell lines with downregulated SFRP1 required greater levels of HDAC suppression to increase SFRP1 expression. In addition, histone acetylation of SFRP1 in NSCLC was enhanced by HDAC inhibitors compared with DKK3 and TACSTD1 [85], but not in CL1-1 generated from the cervix. These results are in accord with the hypothesis that the therapeutic effect of HDAC in NSCLC is caused by the reactivation of SFRP1. Interestingly, the histone acetylation of SALL4 in NSCLC was also enhanced by the HDAC inhibitor in an A549 cell line (Table 3, *P*-values for the CL1-1 cell line were very small, but because of the deacetylation, this effect is not likely to be caused by the HDAC inhibitor). Unfortunately, the microarray analysis by Miyagawa et al. [65], did not include SALL4, thus we cannot check whether SALL4 expression was coincident with HDAC inhibitor resistance.

**Table 3:** Comparison of gene expression between resistant and non-resistant cell lines for adenocarcinoma and squamous cell carcinoma [65], and H3K9K14ac during treatment with an HDAC inhibitor for NSCLC cell lines [85]

Gene expression				
Adenocarcinoma				
		*P*-value	Non-resistant cell lines	resistant cell lines
SFRP1		**4.64 × 10** ^**−4**^	611.06	>92.60
DKK3		6.73 × 10^−2^	263.27	>30.59
Squamous cell carcinoma				
SFRP1		**7.42 × 10** ^**−3**^	304.53	>49.53
DKK3		4.61 × 10^−1^	261.38	<506.25
Histone modification (H3K9K14ac)				
		*P*-value	0 hours	2 hours
	(A549)	**2.90 × 10** ^**−2**^	−1.29	<−0.52
SFRP1	(H1299)	**4.06 × 10** ^**−2**^	−2.51	<−1.85
	(CL1-1)	8.71 × 10^−1^	−1.38	<−1.34
	(A549)	6.19 × 10^−1^	−1.17	<−1.01
DKK3	(H1299)	**1.98 × 10** ^**−3**^	−1.70	<−0.48
	(CL1-1)	1.48 × 10^−1^	−0.59	>−1.13
	(A549)	4.74 × 10^−1^	−1.70	<−1.37
TACSTD1	(H1299)	1.51 × 10^−1^	−2.61	<−2.20
	(CL1-1)	8.62 × 10^−1^	−2.03	>−2.09
	(A549)	**1.71 × 10** ^**−3**^	−2.44	<−1.05
SALL4	(H1299)	5.23 × 10^−1^	−2.62	>−2.86
	(CL1-1)	**1.03 × 10** ^**−4**^	0.97	>−0.59

Significant *P*-values (<0.05) are shown in bold

### Potential of SFRP1 binding to WNT1

**Fig. 2:**
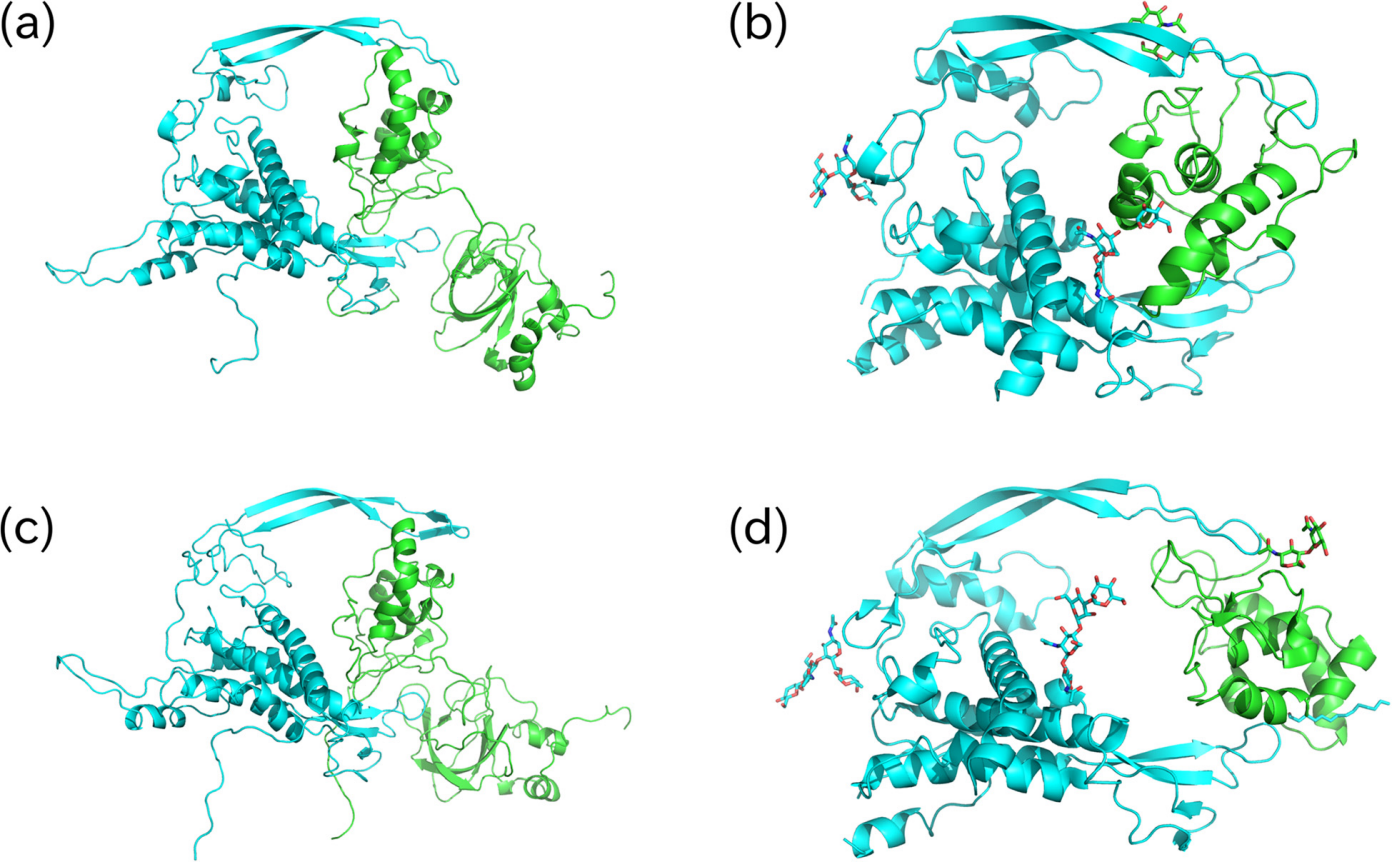
Protein complex. **a** WNT1 (cyan) + SFRP1 (light green) by Fiberdock + ZDOCK, **b** WNT8 (cyan) + CRD of FZ8 (light green) by Fiberdock + ZDOCK, **c** WNT1(cyan) + SFRP1(light green) by GROMACS (time = 2 ns), **d** WNT8 (cyan) + CRD of Fz8 (light green) in PDB (PDB ID: 4F0A)

Next, we validated the ability of SFRP1 to bind to WNT1 as it was the most promising target from our study that affected the Wnt signalling pathway. Although Wnt8 and FZ8 share a cysteine-rich domain (CRD) that forms a protein complex with SFRP1 [86] and FRZB1 that is a similar protein to SFRP1 [87] was reported to bind to WNT1 in *Xenopus* [88], there have been no direct reports investigating the binding of SFRP1 to WNT1 [89]. Therefore, we examined the formation of a SFRP1-WNT1 protein complex using numerical simulation. The tertiary structures of WNT1 and SFRP1 were inferred by RaptorX [90]. Then, the obtained structures were uploaded to the ZDOCK server [91], a rigid body based protein complex predictor. The 10 top ranked protein complex structures obtained were further uploaded to Fiberdock [92] that refines the protein complex structures obtained by other methods by considering the flexible structures of the proteins. Finally, the best candidate (with the minimum energy) reported by Fiberdock was identified as the most reliable candidate for the WNT1-SFRP1 protein complex. Figure 2a shows the structure of the protein complex obtained using this procedure. This structure is very similar to the WNT8-FZ8 complex (Fig. 2d) because the CRDs of SFRP1 and FZ8 were similarly sandwiched by two arms of the Wnt protein. This suggests that SFRP1 can bind to WNT1 and suppress the Wnt signalling pathway.

To confirm the reliability of this structure, we performed two tests. The first was to upload WNT8 and the CRD of FZ8 separately to ZDOCK and Fiberdock, as for the protein tertiary structures of SFRP1 and WNT1 obtained by RaptorX, to determine whether the correct structure was identified as that with the minimum energy. Figure 2b shows a protein complex identified by the combined usage of ZDOCK and Fiberdock. The expected structure contains an FZ8 CRD sandwiched by two arms of WNT8, thus, this confirms the use of our strategy using the combined ZDOCK and Fiberdock systems.

**Fig. 3:**
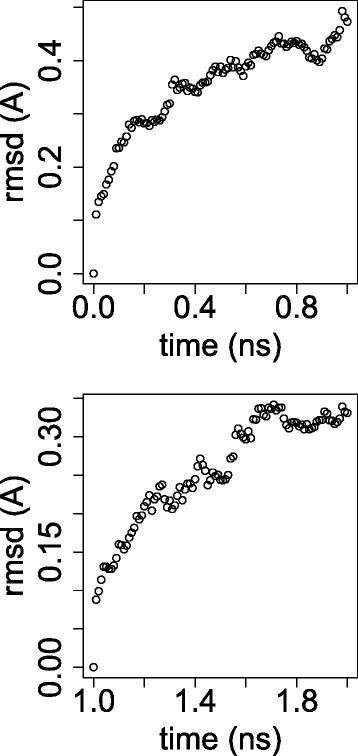
RMSD for GROMACS MD for those from 0 ns to 1 ns and those from 1 ns to 2 ns

The second test was a molecular dynamics (MD) simulation to test the stability of the protein complex predicted by the combined usage of ZDOCK and Fiberdock. The protein complex inferred by ZDOCK and Fiberdock was used as the initial state and MD simulation was performed by GROMACS [93]. The obtained structure was modified to have minimum energy and was simulated under NVT (constant number of molecules, volume and temperature) and NPT (constant number of molecules, pressure and temperature) conditions over 0.1 ns, respectively. Then, a 2 ns equilibration MD was performed. Figure 3 shows the time developments of the root mean square deviation (RMSD) during the first and second 1 ns in equilibration MD. Although the structure heavily fluctuates because RMSD increased with time, the SFRP1 CRD structure was sandwiched by two arms of WNT1 and was maintained even after 2 ns equilibration MD (Fig. 2c) indicating this structure was stable. Thus, SFRP1 binds to WNT1 to suppress the Wnt signalling pathway.

### Possibilities that proteins other than SFRP1 are epigenetic therapy targets

Although this study focused on SFRP1 as a promising candidate for epigenetic therapy of NSCLC, 31 other proteins selected in Table 1 might also have potential for NSCLC epigenetic therapy. SALL4, DKK3 and HOXA5 were suggested to be related to the Wnt signalling pathway. DKK3 suppresses the Wnt signalling path-way by binding to LPR5/6 proteins. Other genes also contribute to many other pathways involved in tumourigenesis (Additional files 6 and 8). Thus, although no strong evidence that proteins other than SFRP1 were epigenetic therapy targets for NSCLC was obtained, it is likely that other proteins identified in this study are epigenetic therapy targets.

## Conclusion

In conclusion, this meta-analysis of reprogrammed NSCLC cell lines indicated that SFRP1 was a promising candidate for NSCLC epigenetic therapy. The reliability of SFRP1 binding to WNT1 to suppress the Wnt signalling pathway was confirmed using numerical investigations. Thus, we propose that SFRP1 is an epigenetic therapy target for NSCLC.

## Methods

### Gene expression and promoter methylation profiles during reprogramming of NSCLC cell lines

Gene expression and promoter methylation profiles were downloaded from the Gene Expression Omnibus (GEO) using GEO ID: GSE35913. Files including gene expression, GSE35911_SampleProbeProfile.txt.gz, were provided as a supplementary file in the subseries GEO ID: GSE35911. Columns annotated as “AVG Signal” were used. Promoter methylation profiles were obtained from “Series Matrix File(s)” in the subseries GEO ID: GSE35912. They consisted of eight cell lines, H1 (ES cell), H358 and H460 (NSCLC), IMR90 (Human Caucasian foetal lung fibroblast), iPCH358, iPCH460, iPSIMR90 (reprogrammed cell lines), and piPCH358 (re-differentiated iPCH358) with three biological replicates. In total, there were 3 replicates × 8 cell lines × 2 properties (gene expression and promoter methylation) = 48 samples. No further normalization processes were applied. Multiple probes attributed to the same gene were not integrated before feature selection and if genes with more than one attributable probes were selected, the genes were regarded as extracted features.

### PCA-based unsupervised FE

PCA based unsupervised FE was performed as previously described [15]. Briefly, *x*_ij_ is *i*th feature that represents gene expression/promoter methylation/histone modification of the *j*th sample. In contrast to the standard usage of PCA, features but not samples were embedded into low dimensional space. Then, *k*th PC score attributed to *i*th feature, *x*_ik_, was expressed as

$$ {x}_{ik}={\sum}_j{c}_{jk}\left({x}_{ij}-{\left\langle {x}_{i'j}\right\rangle}_{i'}\right) $$

where *c*_*jk*_ is the contribution of *j*th sample to *k*th PC (PC loadings) and 〈···〉_*i*_ is the mean over *i*. After identifying biologically critical PCs (in this study, PC3 and PC4, based on hierarchical clustering, Fig. 1), features that are outliers along the specified PC were extracted, because outliers were expected to reflect biological significance (in this study, there was a high correlation between gene expression and promoter methylation) that specified PCs represent. The number of features to be extracted as outliers was decided empirically. In this study, the top 300 outliers, those with larger or smaller (larger absolute values of) *x*_ik_ of kth PC selected for FE, were selected for gene expression and promoter methylation profiles, respectively. Genes listed in Table 1 were those commonly selected as the top ranked outliers for gene expression and methylation profiles in the following four combinations of rankings: larger *x*_ik_ for gene expression and larger *x*_ik_ for promoter methylation; larger *x*_ik_ for gene expression and smaller (negatively larger) *x*_ik_ for promoter methylation; smaller (negatively larger) *x*_ik_ for gene expression and larger *x*_ik_ for promoter methylation; and smaller (negatively larger) *x*_ik_ for gene expression and smaller (negatively larger) *x*_ik_ for promoter methylation.

### Identification of biologically meaningful PCs

Although there are several ways to identify biologically meaningful PCs, this study used the coincidence of *c*_*jk*_ between gene expression and promoter methylation. To identify mostly coincident PCs between gene expression and promoter methylation, we computed the correlation coefficients between *k*th and *k*’th PCs of gene expression or promoter methylation as follows: (all pairs were considered among PCs of gene expression and promoter methylation)

$$ {\uprho}_{kk'}={\left\langle \triangle {c}_{jk}\triangle {c}_{jk'}\right\rangle}_j $$

$$ \triangle {c}_{jk}=\left({c}_{jk}-{\left\langle {c}_{j1k}\right\rangle}_{j1}\right)/{\left[{\left\langle {\left({c}_{j2k}-{\left\langle {c}_{j3k}\right\rangle}_{j3}\right)}^2\right\rangle}_{j2}\right]}^{0.5} $$

where 〈···〉_*j*_ is the mean over *j*. Then, the negative signed absolute value of *ρ*_*kk′*_, − |*ρ*_*kk′*_|, was used as the distance for hierarchical clustering (Unweighted Pair Group Method with Arithmetic mean, [UPGMA], was employed). If a pair of PCs for gene expression and promoter methylation were clustered together with a smaller distance, i.e., with a larger absolute value of correlation coefficient, we employed the pair of PCs of gene expression and promoter methylation, for FE.

### Hierarchical clustering with 23 samples

PCs were computed with 23 samples and hierarchical clustering was performed. As a result, we had 24 hierarchical clusters. Because the correspondence between PCs with a distinct set of 23 samples is incomplete, we re-labelled the first five PCs (PC1, PC2, PC3, PC4 and PC5) and compared them with PCs obtained using 24 samples.

### Categorical regression-based FE

Categorical regression-based FE was defined as follows: *x*_*ij*_ reflects the *i*th feature of the *j*th samples as described above; therefore *x*_*ij*_ can be represented as:

$$ {x}_{ij}={a}_{i0}+{\sum}_l{a}_{il}{\delta}_{jl,} $$

with *δ*_*jl*_ = 1 only when the *j*th sample belongs to the *l*th category (in this study, each category corresponds to the type of cell line), otherwise it is 0. Category summation was determined and *a*_*jl*_s were the fitting parameters. Because independent variables are categorical, the above regression equation belongs to a category of equations often named categorical regression. For each *i*th feature, *P*-values were computed using the lm function implemented in R [94] (this can be easily performed if factors corresponding to the types of cell lines are used as independent variables in lm). Genes were ranked based upon obtained *P*-values and the top 300 ranked significant genes (those with smaller *P*-values) were extracted. Genes listed in Table 1 were those commonly selected as top ranked genes for gene expression and methylation profiles.

### Disease associations with genes

Disease associations with genes were investigated by Gendoo [25], a literature-based disease-gene association database.

### Probe annotation

For gene expression, probe annotations were based on the “Accession” column (for RefSeq gene ID) or “Symbol” column (for gene symbol) in the GSE35911_SampleProbeProfile.txt.gz file. For promoter methylation, GPL849065.txt available from the GEO ID: GSE35912 file was used and the “Accession” column was used to assign a Refseq gene ID to each probe.

### Gene expression for comparison of resistant and non-resistant cell lines

Gene expression profiles used for the comparison between resistant and non-resistant cell lines were obtained from GSE4127 [65]. The data set included in the “Series Matrix File(s)” was used for analysis without further normalization. RERF-LC-MS, PC14, PC9, A549, and RERF-LC-KJ were regarded as non-resistant cell lines and CP7, ABC-1, PC3 and LC2/ad were regarded as resistant cell lines.

### Histone modification during treatment with an HDAC inhibitor

Histone modification profiles used for analysis during treatment with an HDAC inhibitor were obtained from GSE20304 [85]. Data sets included in the “Series Matrix File(s)” were used for analysis without further normalization.

### Inference of protein tertiary structures

Amino acid sequences extracted from Uniprot (Q8N474.1 for SFRP1 HUMAN and P04628.1 for WNT1 HUMAN) were uploaded to the RaptorX server and inferred protein structures were used for further analyses, i.e., uploading to ZDOCK, Fiberdock and MD by GROMACS.

### MD by GROMACS

GROMACS 5.0.4 compiled with enabling mpi (Message Passing Interface) and GPU usage was used for MD. The protein complex of SFRP1 and WNT1 inferred by the combined usage of ZDOCK and Fiberdock (Fig. 2a) was used as the initial structure of the protein complex. Force field used was OPLS-AA/L (all-atom force field) and group 13 “SOL” was employed for embedding ions. At first, energy minimization was performed and NVT and NPT conditions of the simulation followed. Finally, an equilibration run was executed over 2 ns. For all the procedures, we followed the instructions shown in [95].

## Additional files

Additional file 1:
*c*_*jk*_ of PC3 and PC4 employed for feature extraction. The left column corresponds to gene expression and the right column corresponds to promoter methylation. PC3 and PC4 show distinct sample dependence. PC3 represents sample dependence that distinguishes between two NSCLC cell lines and non-NSCLC cell lines, while PC4 represents the distinction between two NSCLC cell lines in addition to that between two NSCLC cell lines and non-NSCLC cell lines. (PDF 7 kb) Additional file 2: Hierarchical clustering with 23 samples. Hierarchical clustering between PCs with 23 samples. Left (right) column corresponds to those before (after) re-labelling. (PDF 77 kb) Additional file 3: FE based upon categorical regression. Gene expression/promoter methylation of genes selected by categorical regression. FE based upon categorical regression does not always guarantee a high correlation between gene expression and promoter methylation, because it simply filters those with distinct expression/methylation among samples. However, among eight genes selected, four had a significant (*P* < 0.05) negative correlation coefficient between gene expression and promoter methylation. This suggested the feasibility of FE based upon categorical regression. The correlation between gene expression and promoter methylation is very high. However, only one gene had a significant positive correlation. (PDF 19 kb) Additional file 4: Genes selected based upon PC3. Gene expression/promoter methylation of genes selected by PCA based unsupervised FE employing PC3. Among eleven selected genes, eight genes had a significant (*P* < 0.05) negative correlation between gene expression and promoter methylation. Because we did not restrict the selection of genes to those with negative correlations, large numbers of genes with negative correlations demonstrate the feasibility of our methodology. (PDF 26 kb) Additional file 5: Genes selected based upon PC4. Gene expression/promoter methylation of genes selected by PCA based unsupervised FE employing PC4. Among 16 selected genes, 12 genes had a significant (*P* < 0.05) negative correlation between gene expression and promoter methylation. Because we did not restrict the selection of genes to those with negative correlations, large numbers of genes with negative correlations indicate the feasibility of our methodology. (PDF 36 kb) Additional file 6: Disease associations of genes in Table 1. Disease associations listed with Gendoo server and associated *P*-values. (PDF 99 kb) Additional file 7: KEGG pathways associated with genes in Table 1. Pathway image files downloaded from KEGG and the html file is linked to these images. (ZIP 963 kb) Additional file 8: Literature searches of genes in Table 1. (PDF 127 kb)
