# Supplementary figures and images for: SFRP1 is a possible candidate for epigenetic therapy in non-small cell lung cancer
Source: BMC Med Genomics. 2016 Aug 12;9(Suppl 1):28. doi: 10.1186/s12920-016-0196-3 (PMC4989892; doi:10.1186/s12920-016-0196-3)

**(a) PC3**

**COR= -8.31e-01 P= 2.48e-07**

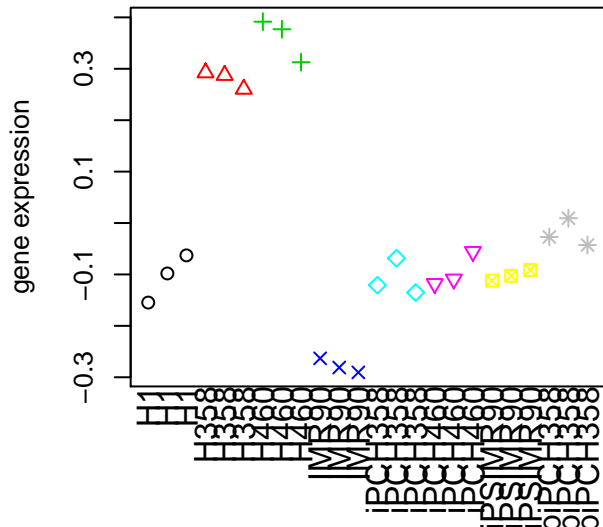

**(b)**

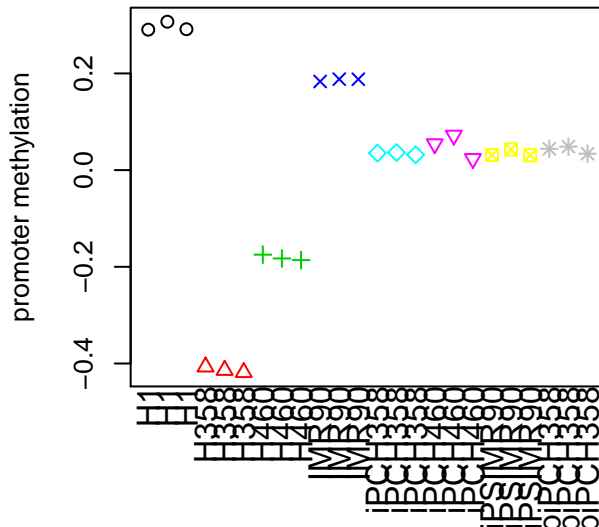

**(c) PC4**

**COR= 8.39e-01 P= 1.48e-07**

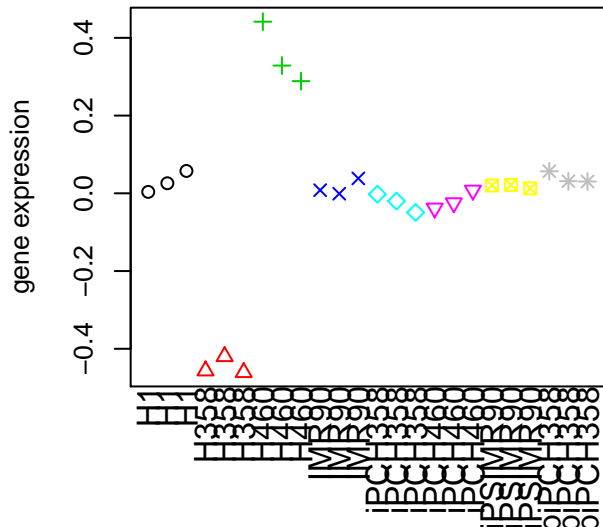

**(d)**

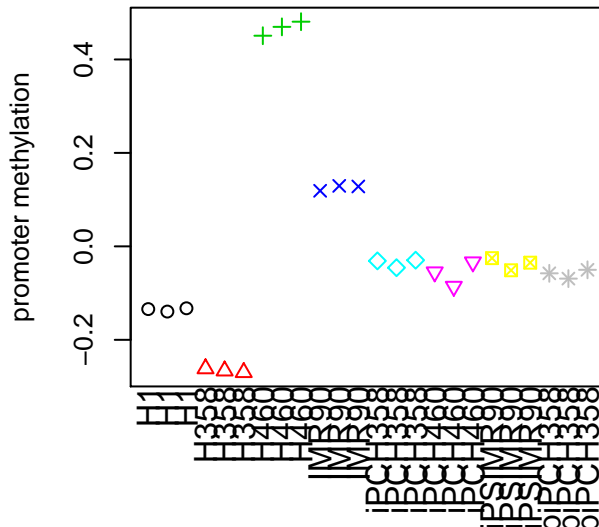

Supplement: Additional file 1: — c jk of PC3 and PC4 employed for feature extraction. The left column corresponds to gene expression and the right column corresponds to promoter methylation. PC3 and PC4 show distinct sample dependence. PC3 represents sample dependence that distinguishes between two NSCLC cell lines and non-NSCLC cell lines, while PC4 represents the distinction between two NSCLC cell lines in addition to that between two NSCLC cell lines and non-NSCLC cell lines. (PDF 7 kb) [file 12920_2016_196_MOESM1_ESM.pdf]

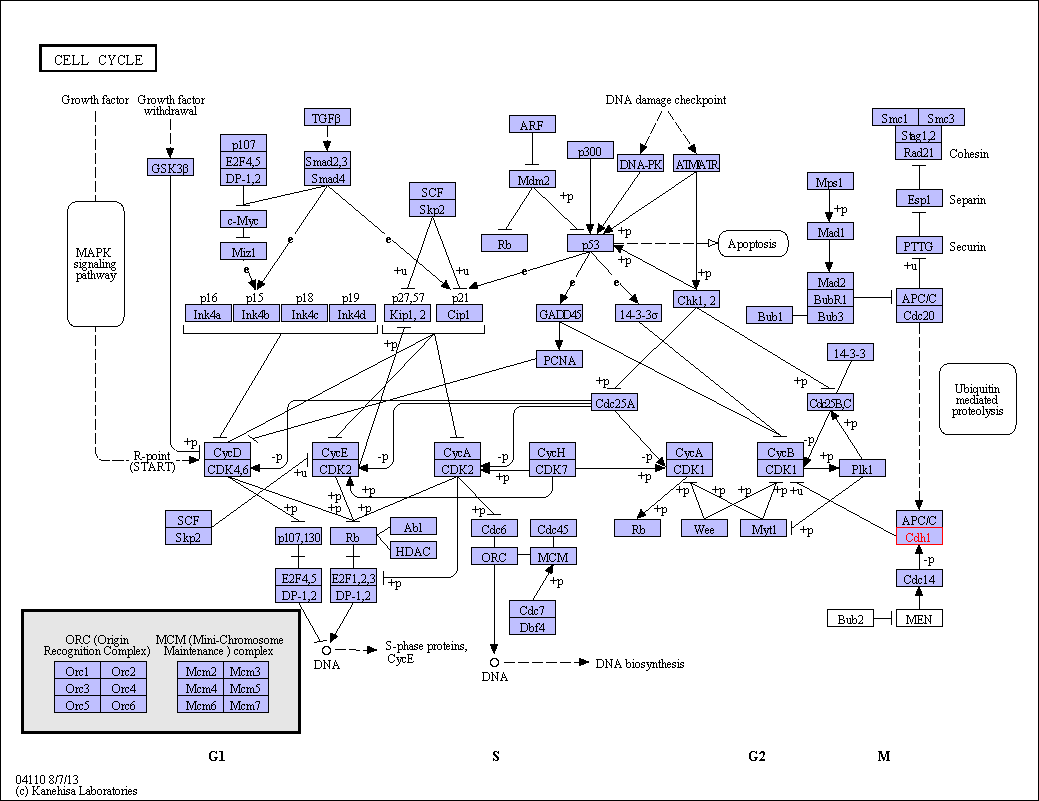

Supplement: Additional file 7: — KEGG pathways associated with genes in Table 1. Pathway image files downloaded from KEGG and the html file is linked to these images. (ZIP 963 kb) [file 12920_2016_196_MOESM7_ESM.zip › Kegg_Figs/ko04110.png]

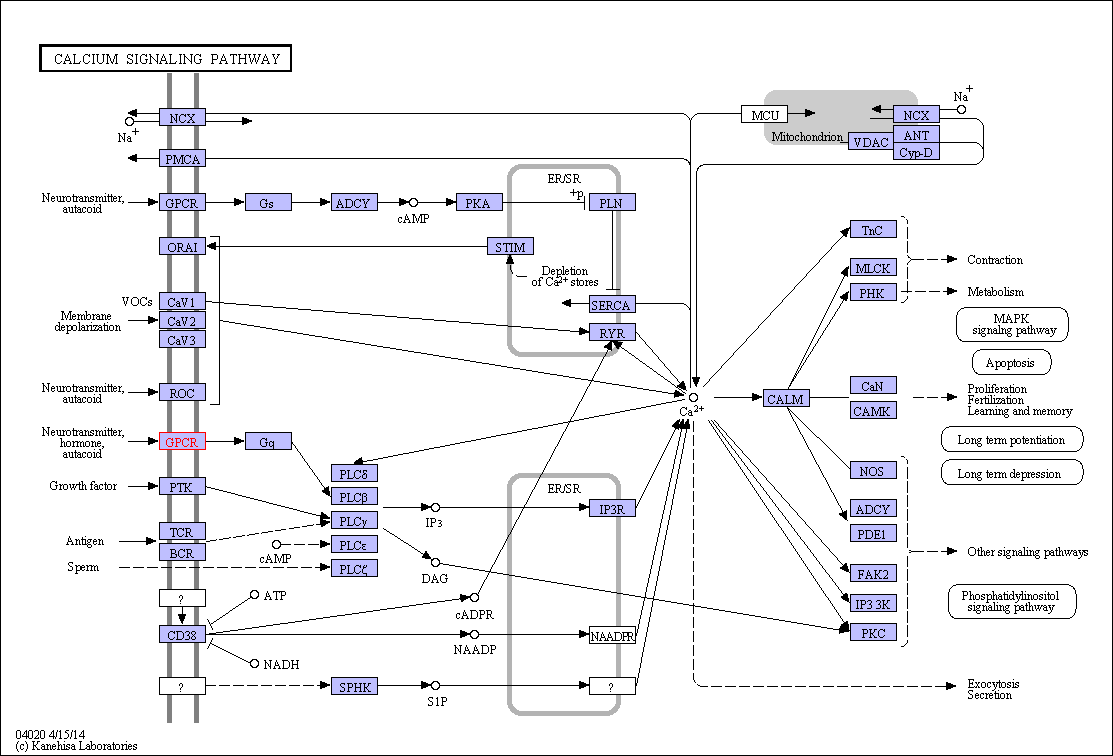

Supplement: Additional file 7: — KEGG pathways associated with genes in Table 1. Pathway image files downloaded from KEGG and the html file is linked to these images. (ZIP 963 kb) [file 12920_2016_196_MOESM7_ESM.zip › Kegg_Figs/ko04020.png]

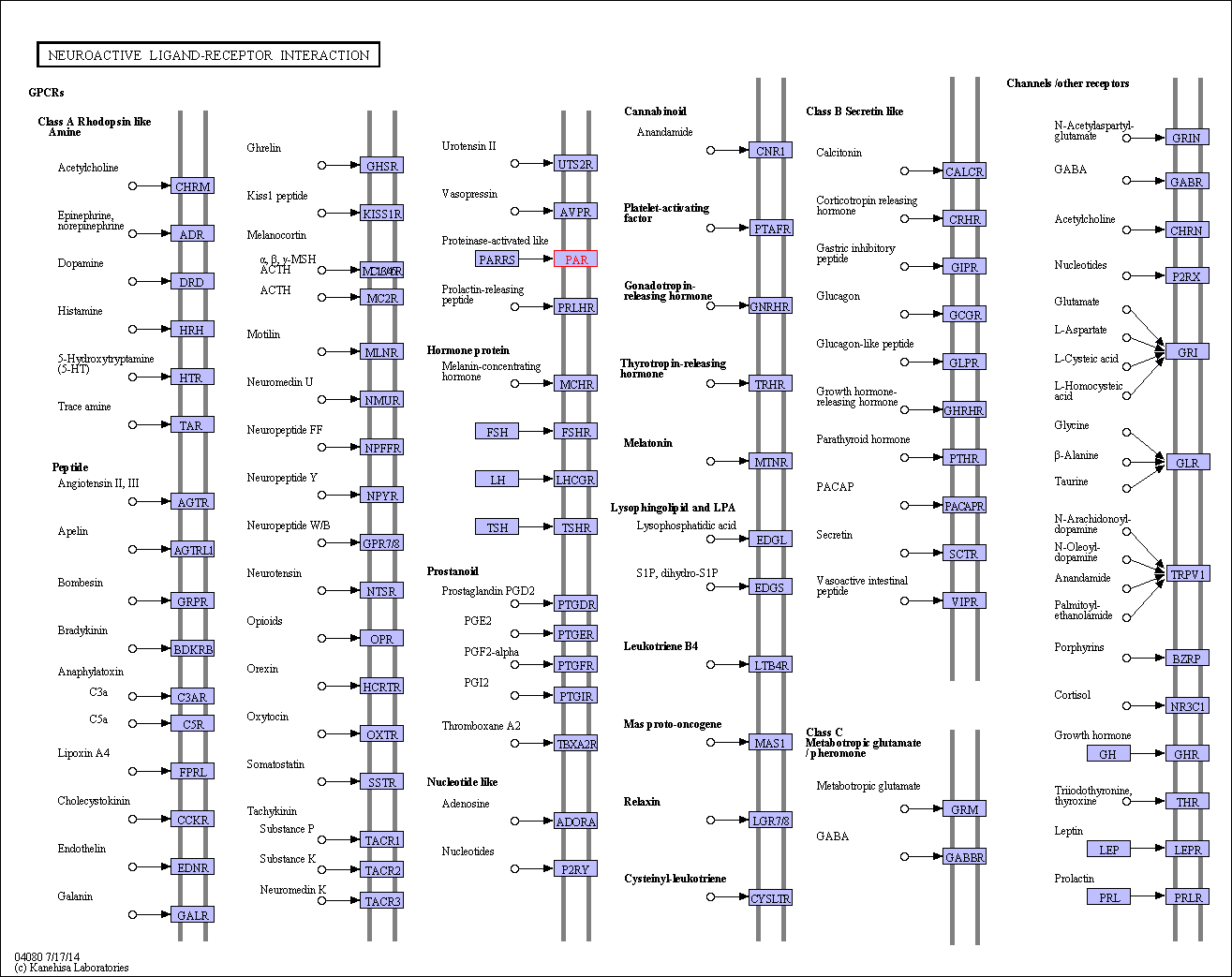

Supplement: Additional file 7: — KEGG pathways associated with genes in Table 1. Pathway image files downloaded from KEGG and the html file is linked to these images. (ZIP 963 kb) [file 12920_2016_196_MOESM7_ESM.zip › Kegg_Figs/ko04080.png]

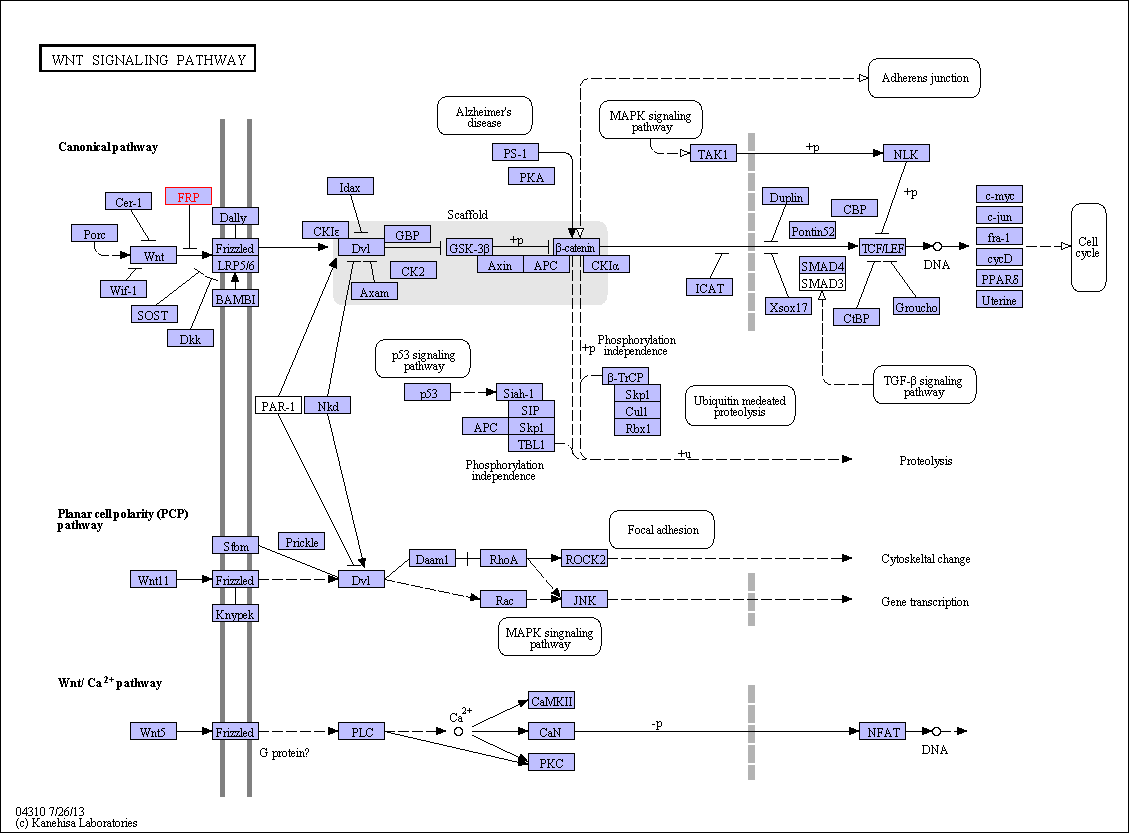

Supplement: Additional file 7: — KEGG pathways associated with genes in Table 1. Pathway image files downloaded from KEGG and the html file is linked to these images. (ZIP 963 kb) [file 12920_2016_196_MOESM7_ESM.zip › Kegg_Figs/ko04310.png]

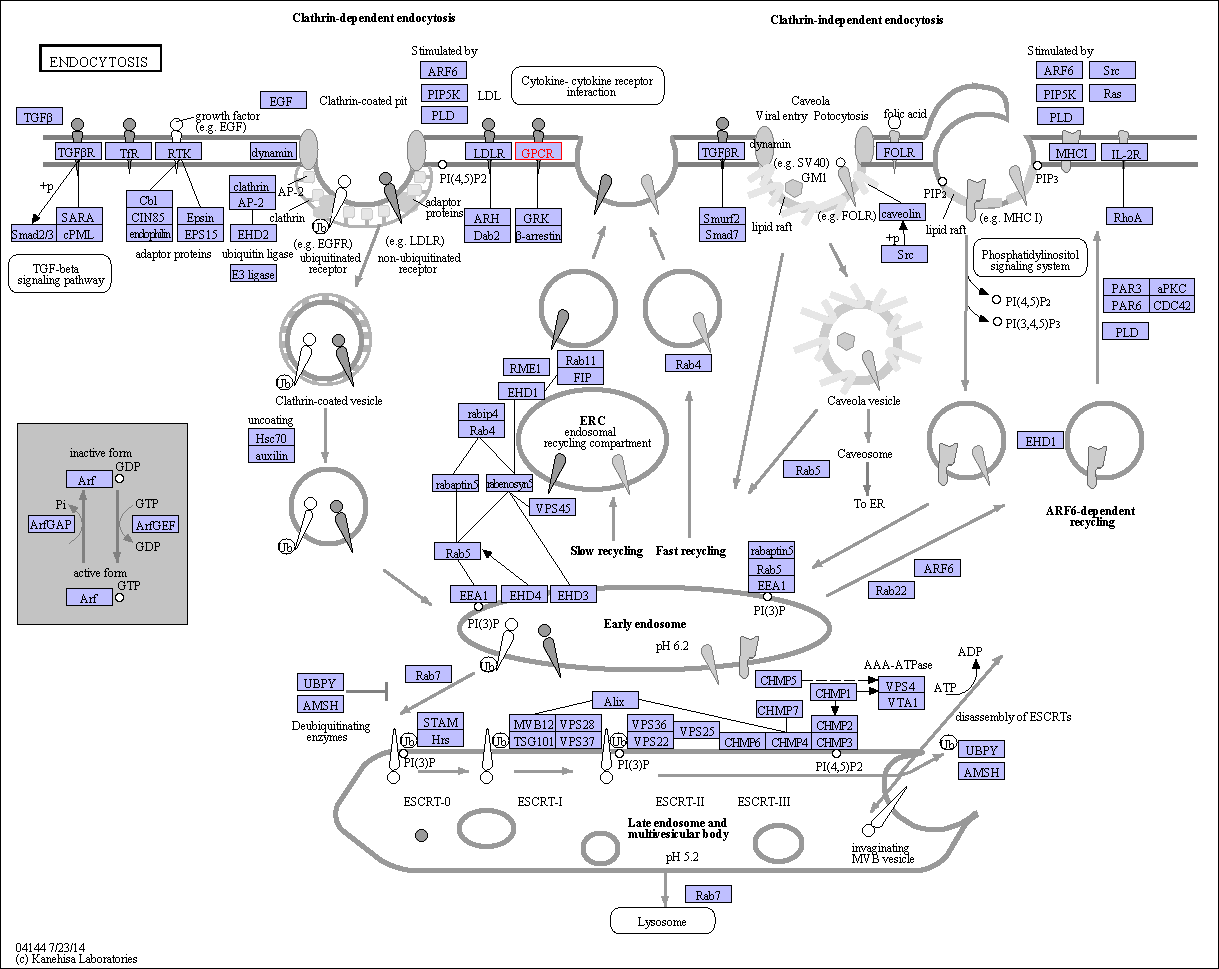

Supplement: Additional file 7: — KEGG pathways associated with genes in Table 1. Pathway image files downloaded from KEGG and the html file is linked to these images. (ZIP 963 kb) [file 12920_2016_196_MOESM7_ESM.zip › Kegg_Figs/ko04144.png]

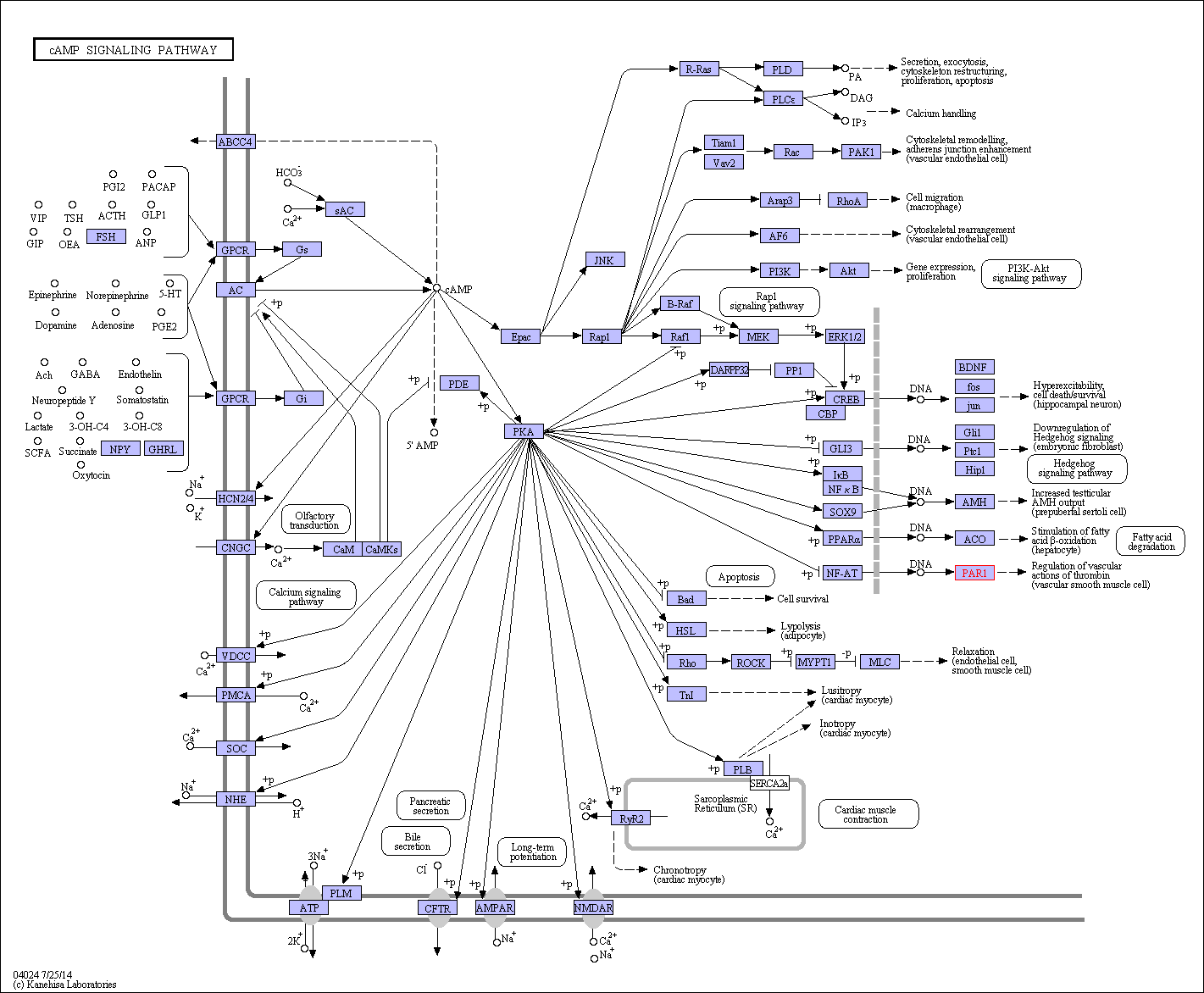

Supplement: Additional file 7: — KEGG pathways associated with genes in Table 1. Pathway image files downloaded from KEGG and the html file is linked to these images. (ZIP 963 kb) [file 12920_2016_196_MOESM7_ESM.zip › Kegg_Figs/ko04024.png]

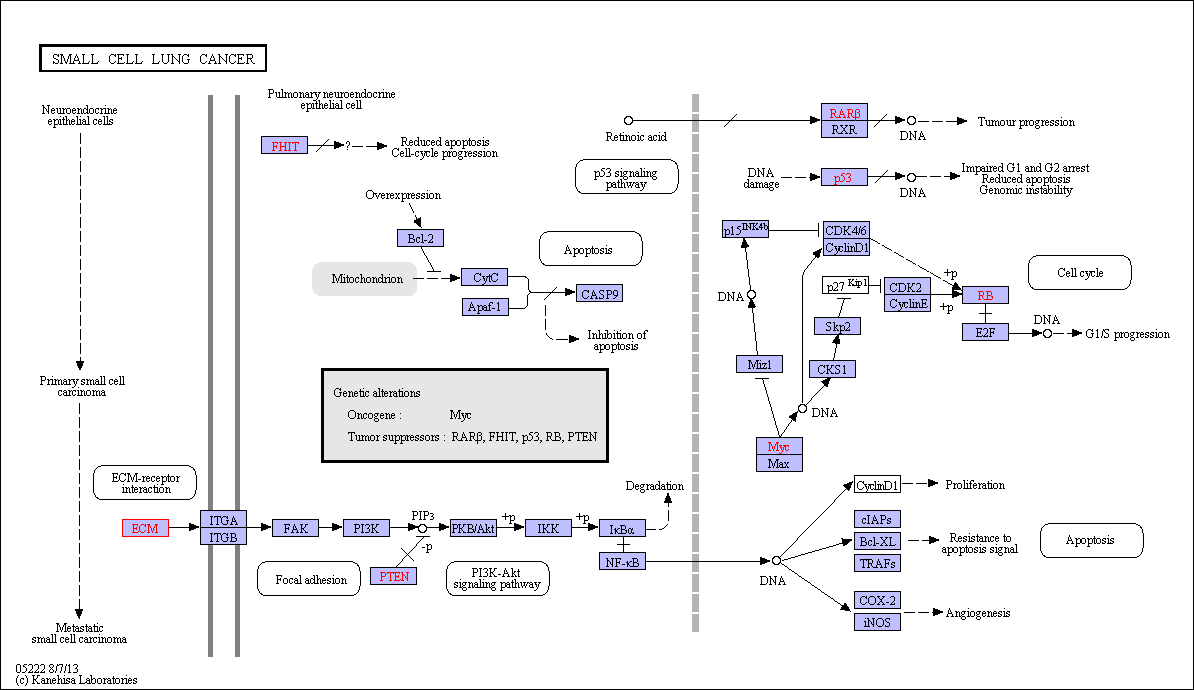

Supplement: Additional file 7: — KEGG pathways associated with genes in Table 1. Pathway image files downloaded from KEGG and the html file is linked to these images. (ZIP 963 kb) [file 12920_2016_196_MOESM7_ESM.zip › Kegg_Figs/ko05222.png]

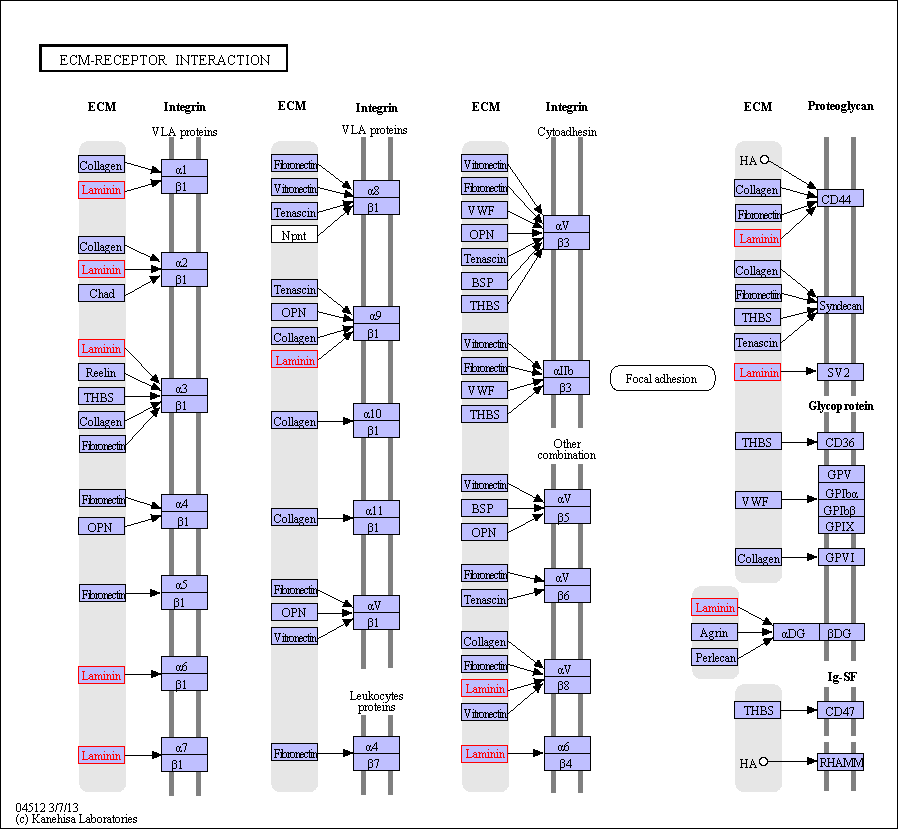

Supplement: Additional file 7: — KEGG pathways associated with genes in Table 1. Pathway image files downloaded from KEGG and the html file is linked to these images. (ZIP 963 kb) [file 12920_2016_196_MOESM7_ESM.zip › Kegg_Figs/ko04512.png]

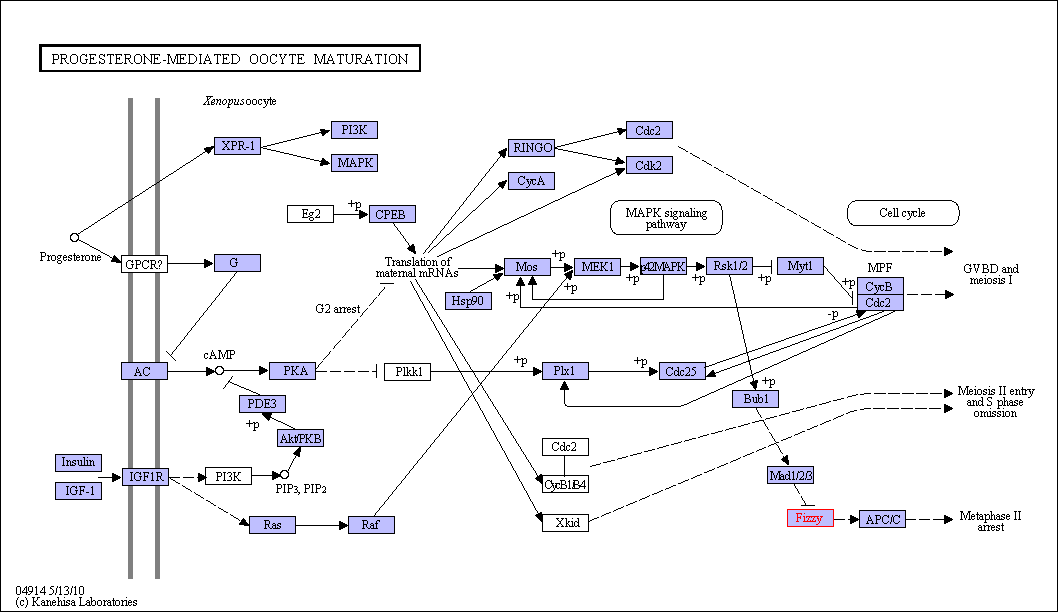

Supplement: Additional file 7: — KEGG pathways associated with genes in Table 1. Pathway image files downloaded from KEGG and the html file is linked to these images. (ZIP 963 kb) [file 12920_2016_196_MOESM7_ESM.zip › Kegg_Figs/ko04914.png]

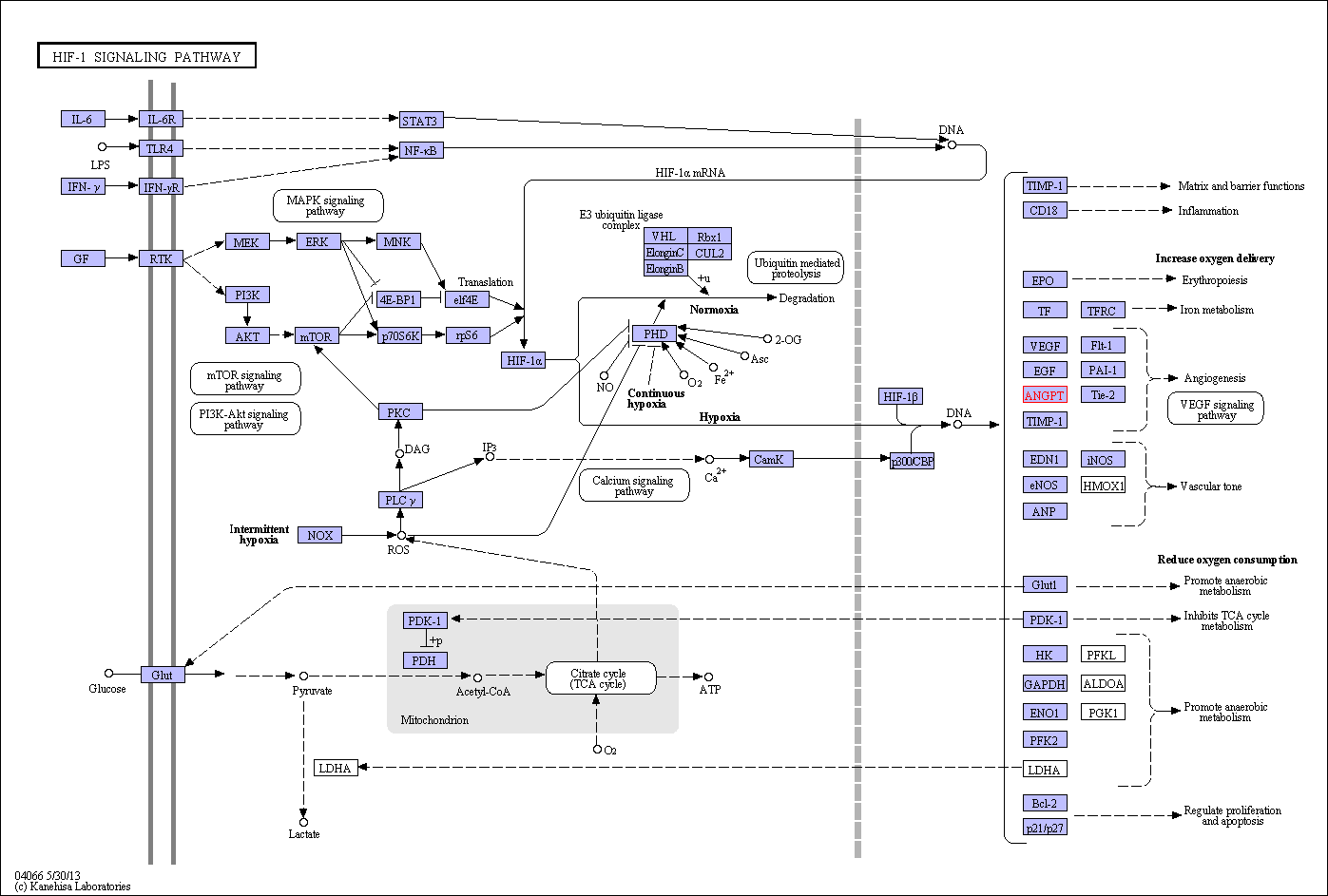

Supplement: Additional file 7: — KEGG pathways associated with genes in Table 1. Pathway image files downloaded from KEGG and the html file is linked to these images. (ZIP 963 kb) [file 12920_2016_196_MOESM7_ESM.zip › Kegg_Figs/ko04066.png]

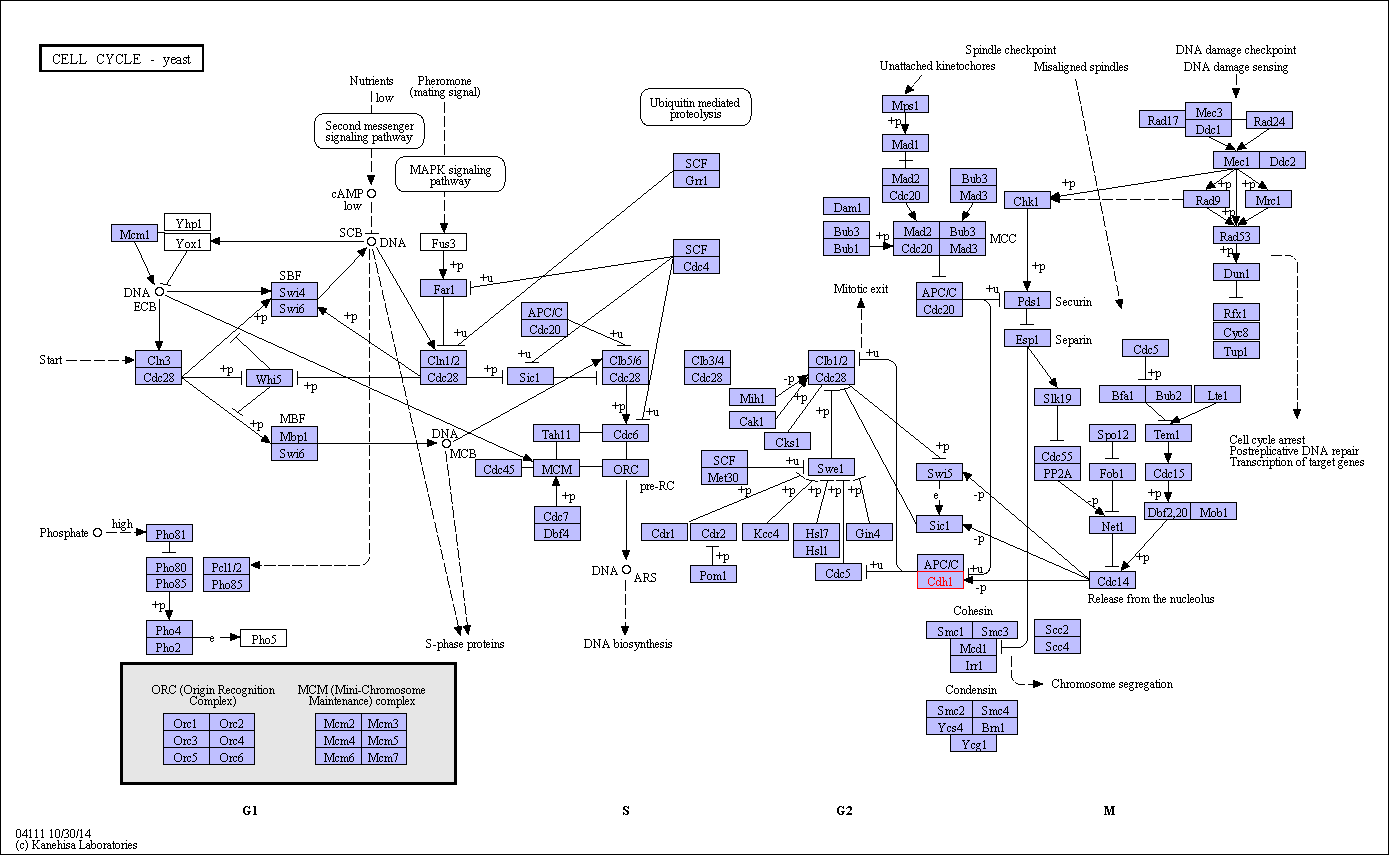

Supplement: Additional file 7: — KEGG pathways associated with genes in Table 1. Pathway image files downloaded from KEGG and the html file is linked to these images. (ZIP 963 kb) [file 12920_2016_196_MOESM7_ESM.zip › Kegg_Figs/ko04111.png]

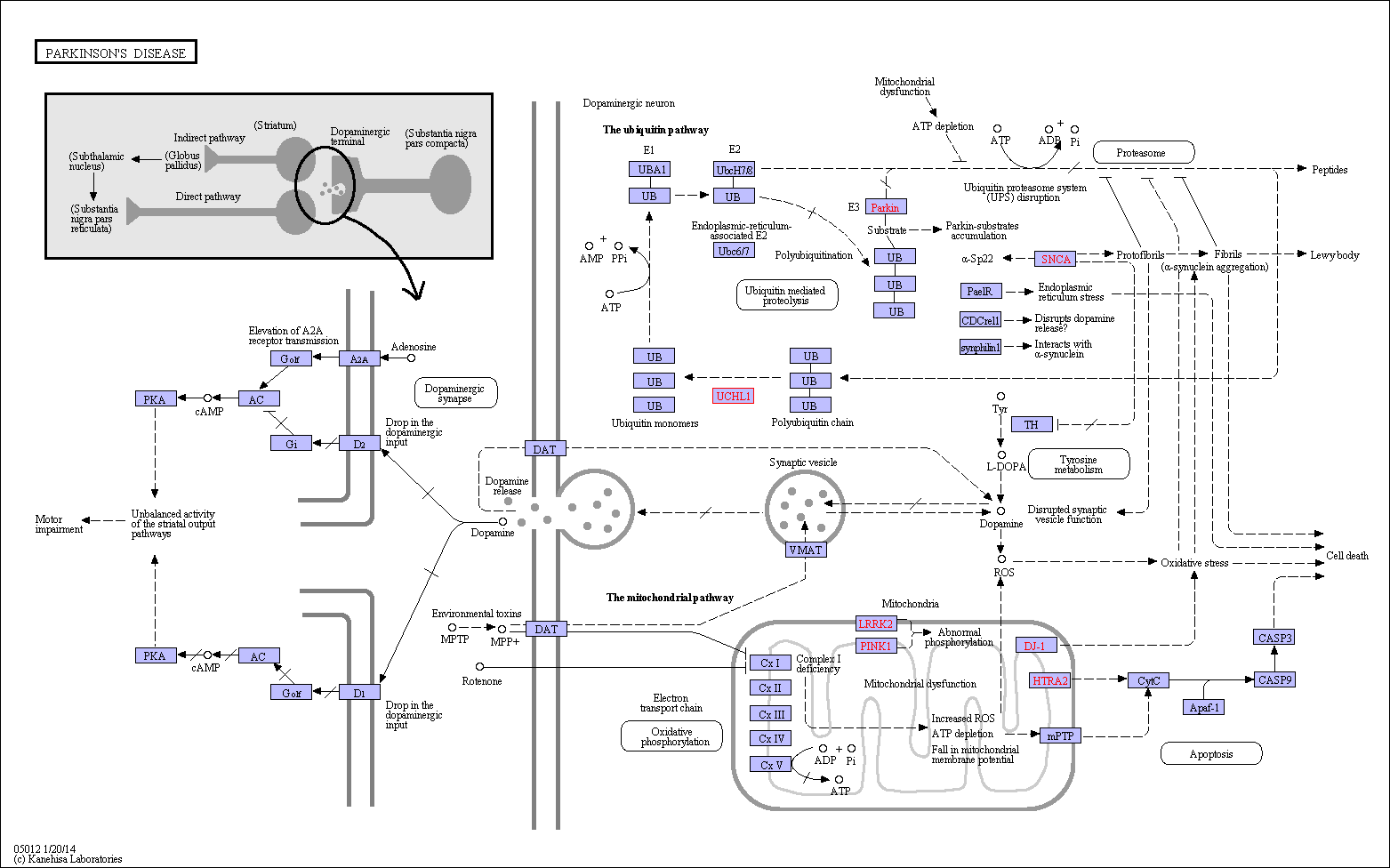

Supplement: Additional file 7: — KEGG pathways associated with genes in Table 1. Pathway image files downloaded from KEGG and the html file is linked to these images. (ZIP 963 kb) [file 12920_2016_196_MOESM7_ESM.zip › Kegg_Figs/ko05012.png]

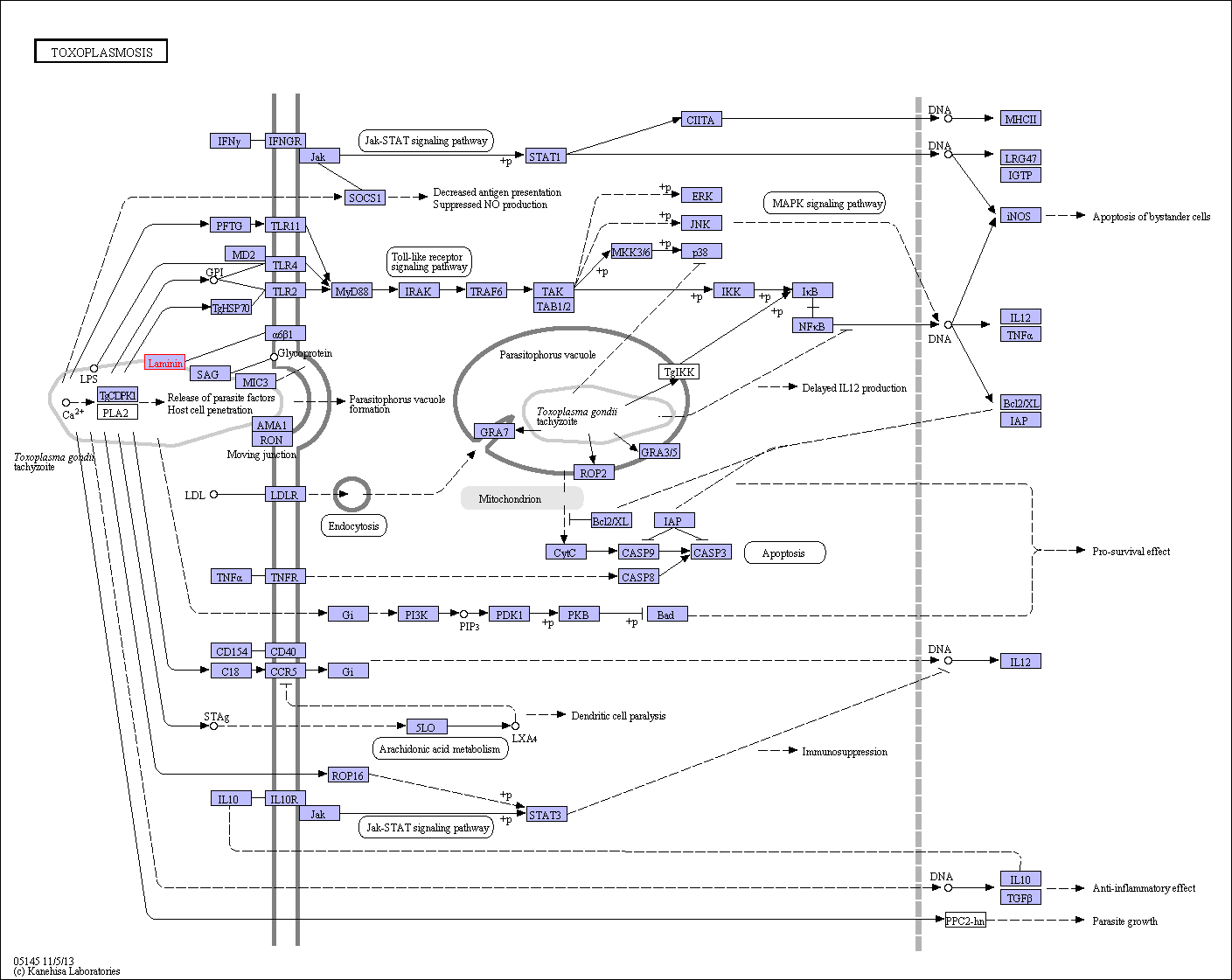

Supplement: Additional file 7: — KEGG pathways associated with genes in Table 1. Pathway image files downloaded from KEGG and the html file is linked to these images. (ZIP 963 kb) [file 12920_2016_196_MOESM7_ESM.zip › Kegg_Figs/ko05145.png]

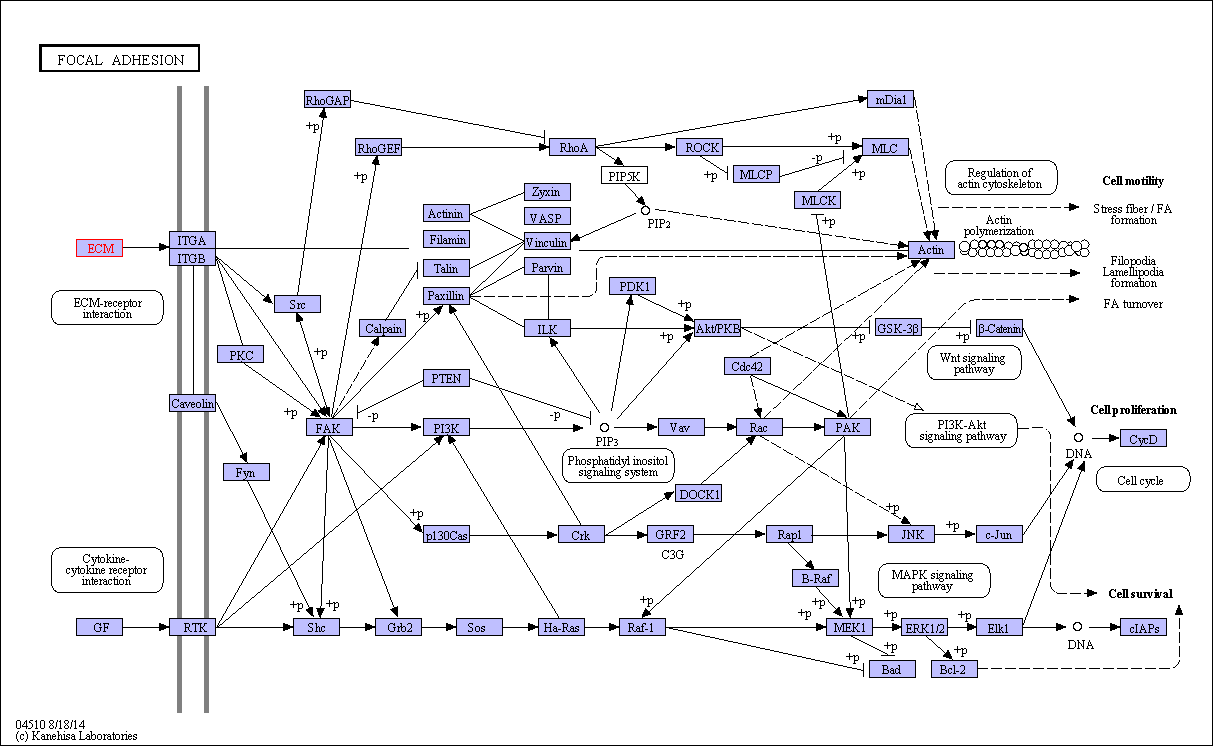

Supplement: Additional file 7: — KEGG pathways associated with genes in Table 1. Pathway image files downloaded from KEGG and the html file is linked to these images. (ZIP 963 kb) [file 12920_2016_196_MOESM7_ESM.zip › Kegg_Figs/ko04510.png]

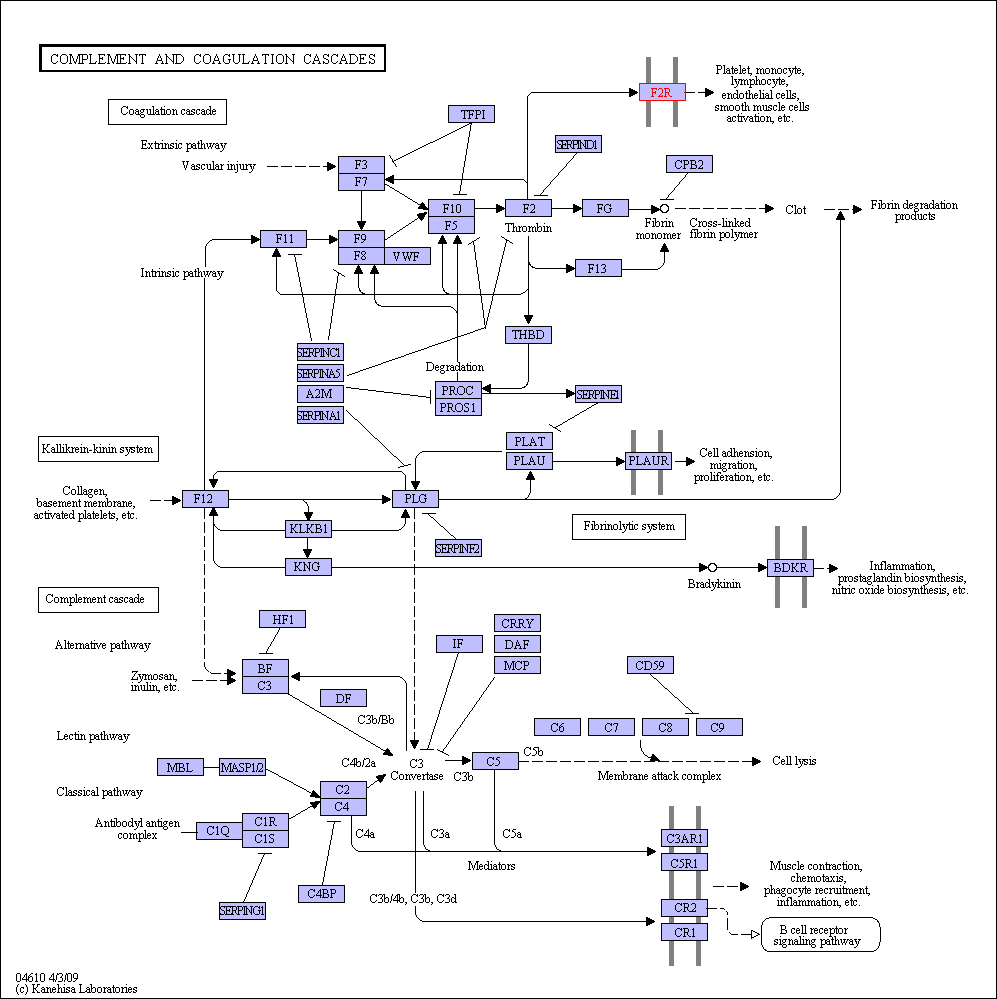

Supplement: Additional file 7: — KEGG pathways associated with genes in Table 1. Pathway image files downloaded from KEGG and the html file is linked to these images. (ZIP 963 kb) [file 12920_2016_196_MOESM7_ESM.zip › Kegg_Figs/ko04610.png]

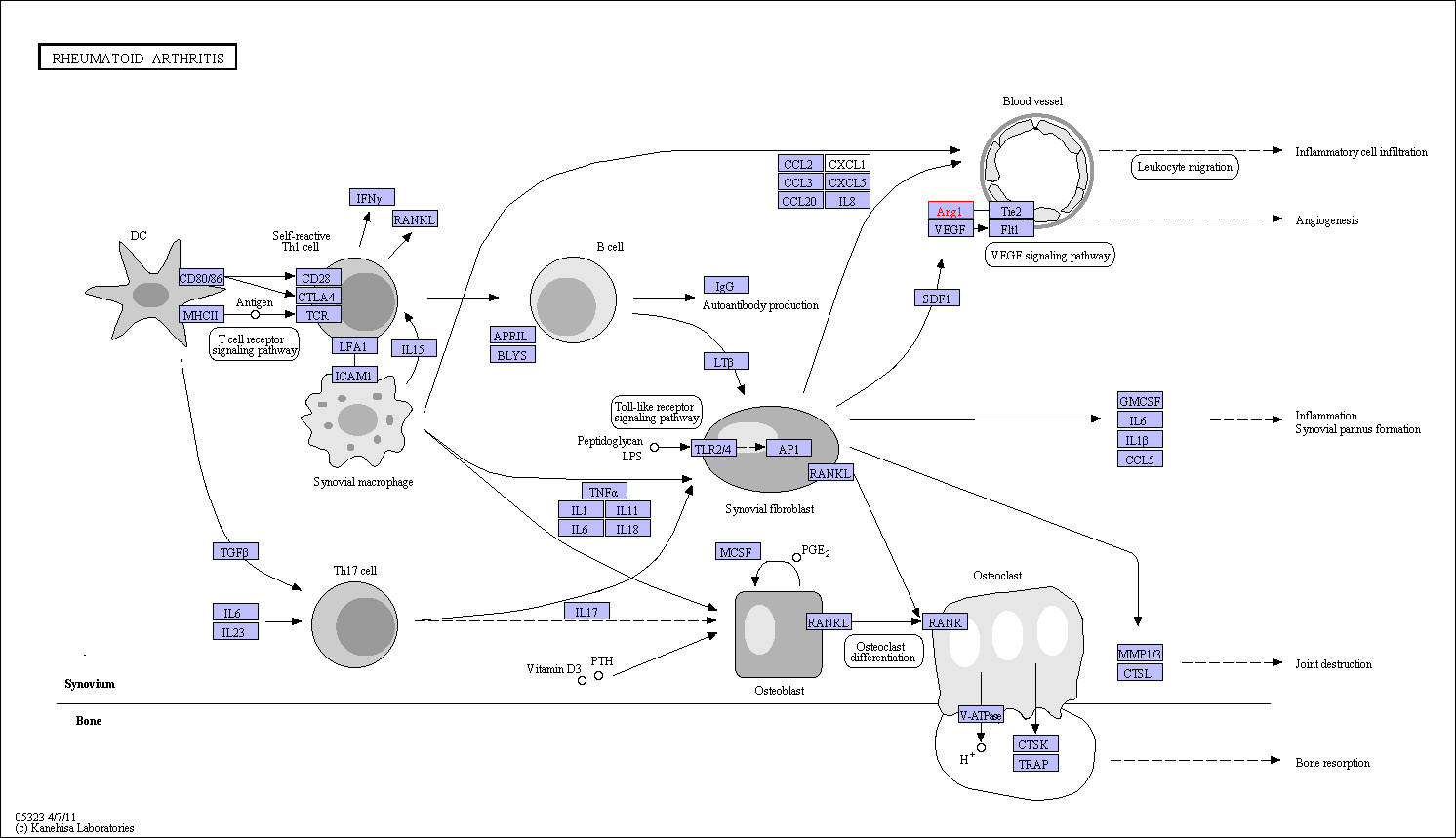

Supplement: Additional file 7: — KEGG pathways associated with genes in Table 1. Pathway image files downloaded from KEGG and the html file is linked to these images. (ZIP 963 kb) [file 12920_2016_196_MOESM7_ESM.zip › Kegg_Figs/ko05323.png]

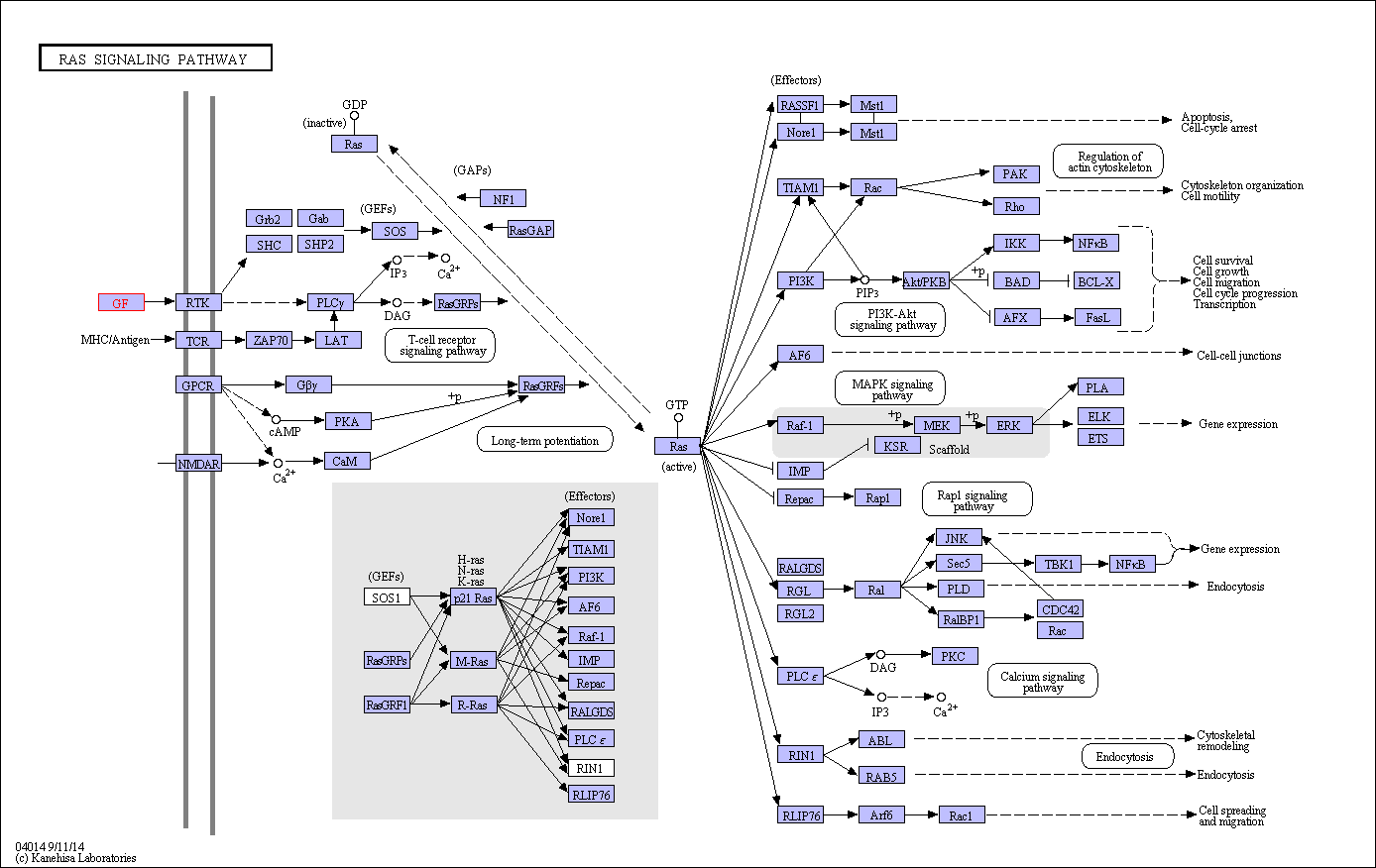

Supplement: Additional file 7: — KEGG pathways associated with genes in Table 1. Pathway image files downloaded from KEGG and the html file is linked to these images. (ZIP 963 kb) [file 12920_2016_196_MOESM7_ESM.zip › Kegg_Figs/ko04014.png]

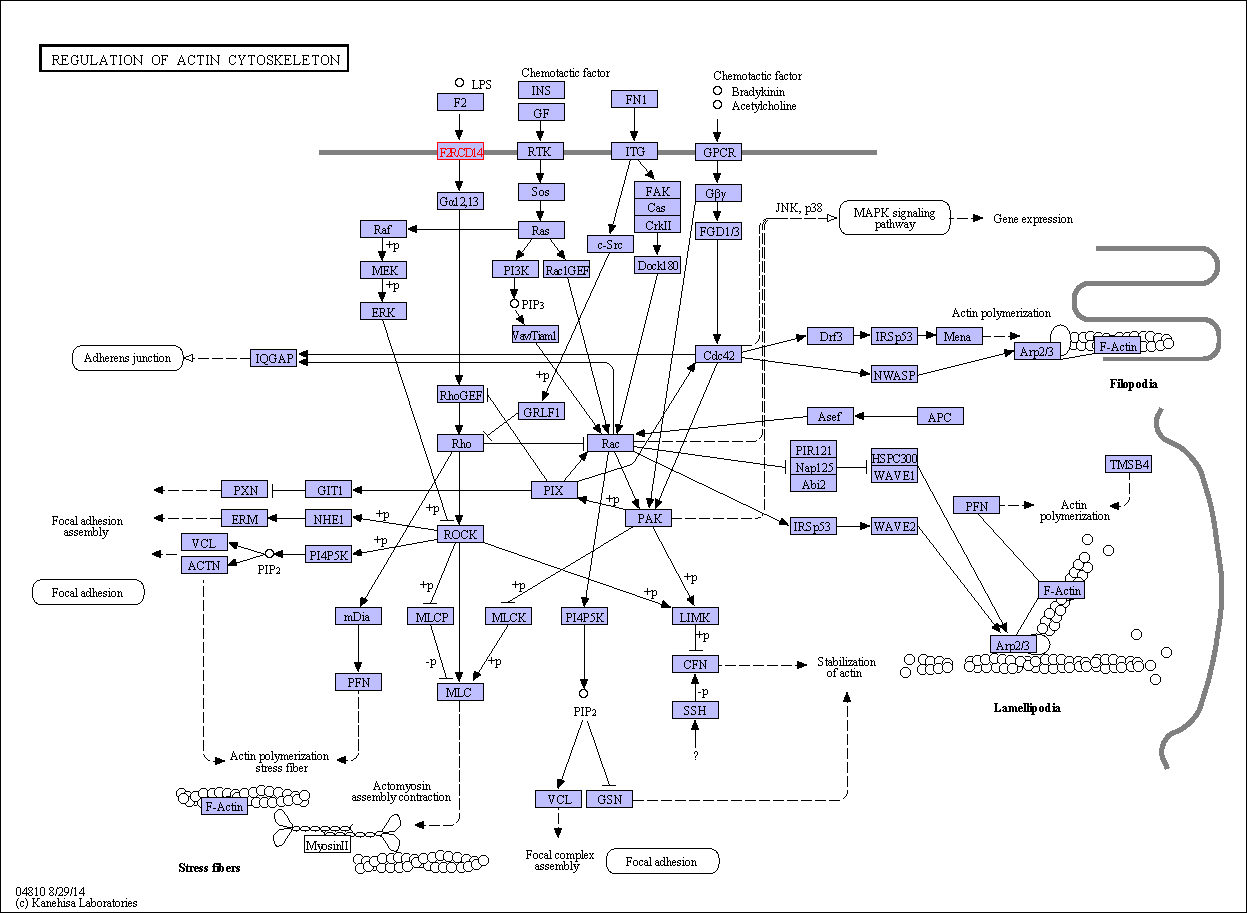

Supplement: Additional file 7: — KEGG pathways associated with genes in Table 1. Pathway image files downloaded from KEGG and the html file is linked to these images. (ZIP 963 kb) [file 12920_2016_196_MOESM7_ESM.zip › Kegg_Figs/ko04810.png]

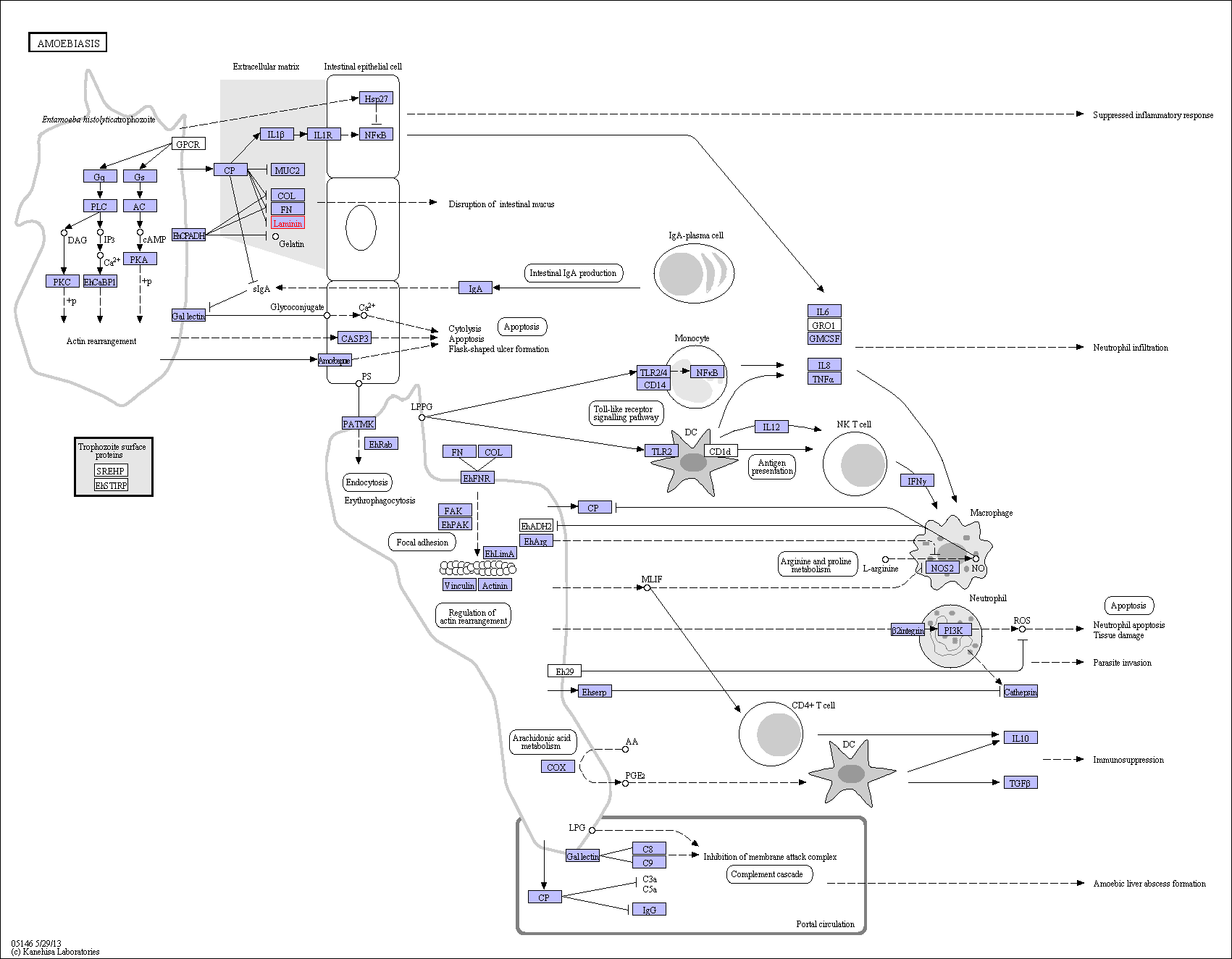

Supplement: Additional file 7: — KEGG pathways associated with genes in Table 1. Pathway image files downloaded from KEGG and the html file is linked to these images. (ZIP 963 kb) [file 12920_2016_196_MOESM7_ESM.zip › Kegg_Figs/ko05146.png]

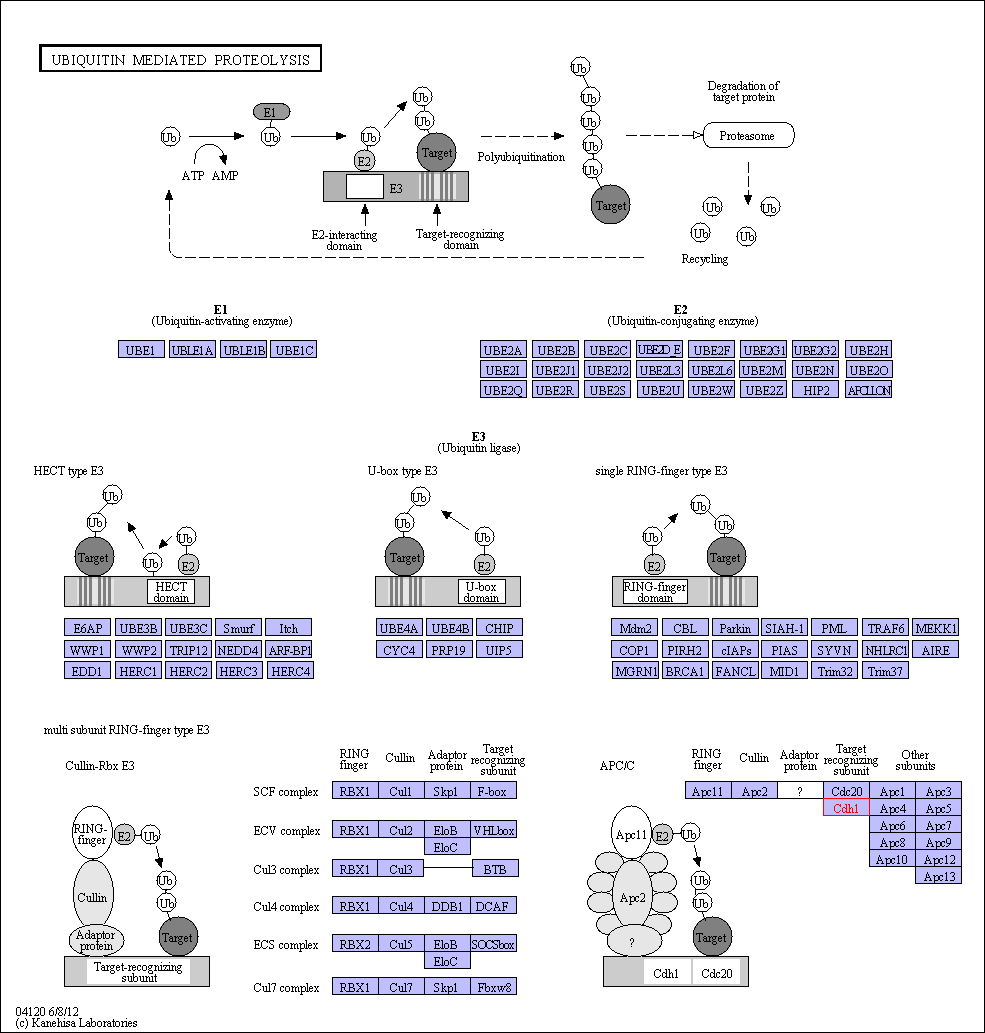

Supplement: Additional file 7: — KEGG pathways associated with genes in Table 1. Pathway image files downloaded from KEGG and the html file is linked to these images. (ZIP 963 kb) [file 12920_2016_196_MOESM7_ESM.zip › Kegg_Figs/ko04120.png]

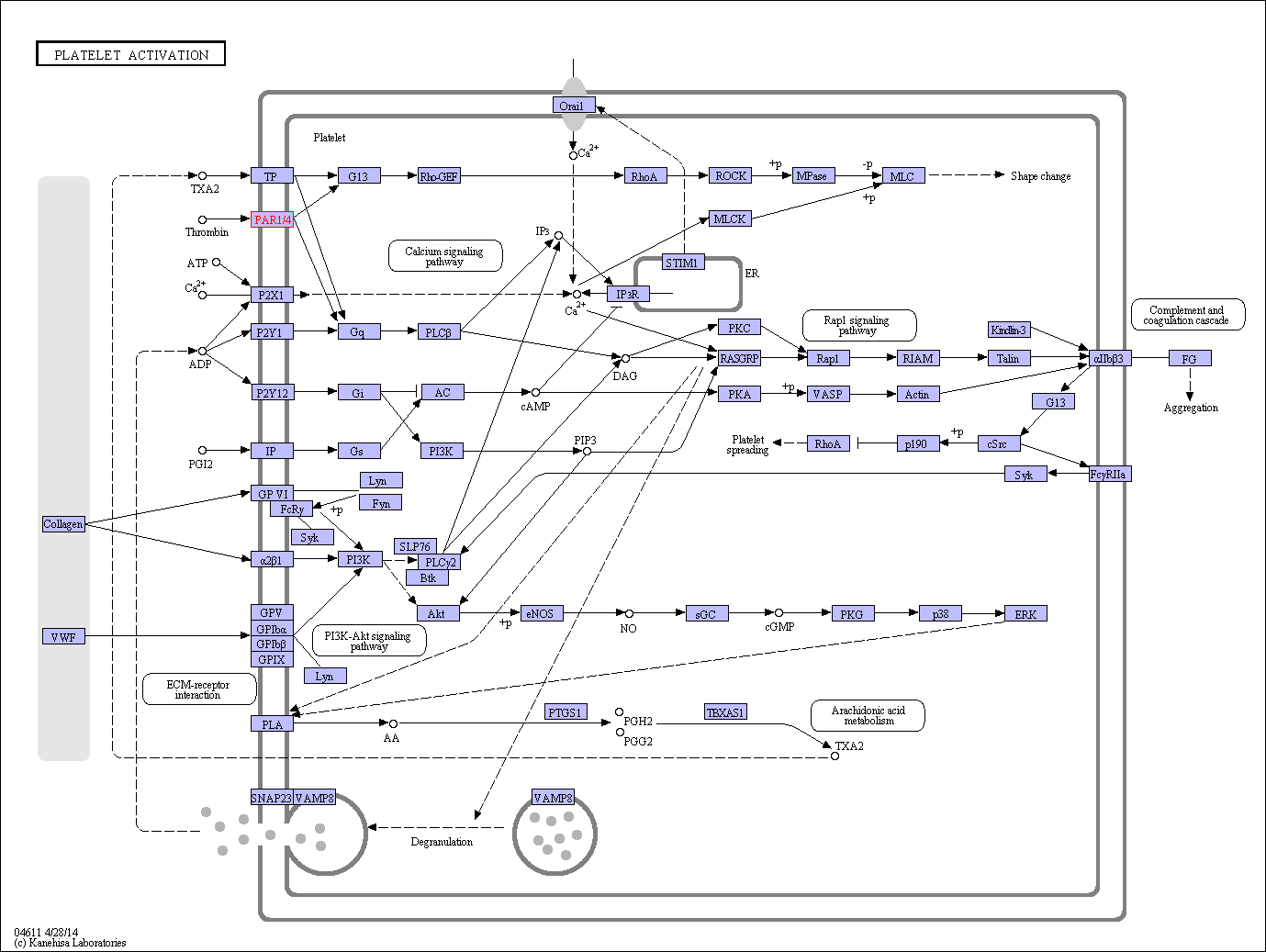

Supplement: Additional file 7: — KEGG pathways associated with genes in Table 1. Pathway image files downloaded from KEGG and the html file is linked to these images. (ZIP 963 kb) [file 12920_2016_196_MOESM7_ESM.zip › Kegg_Figs/ko04611.png]

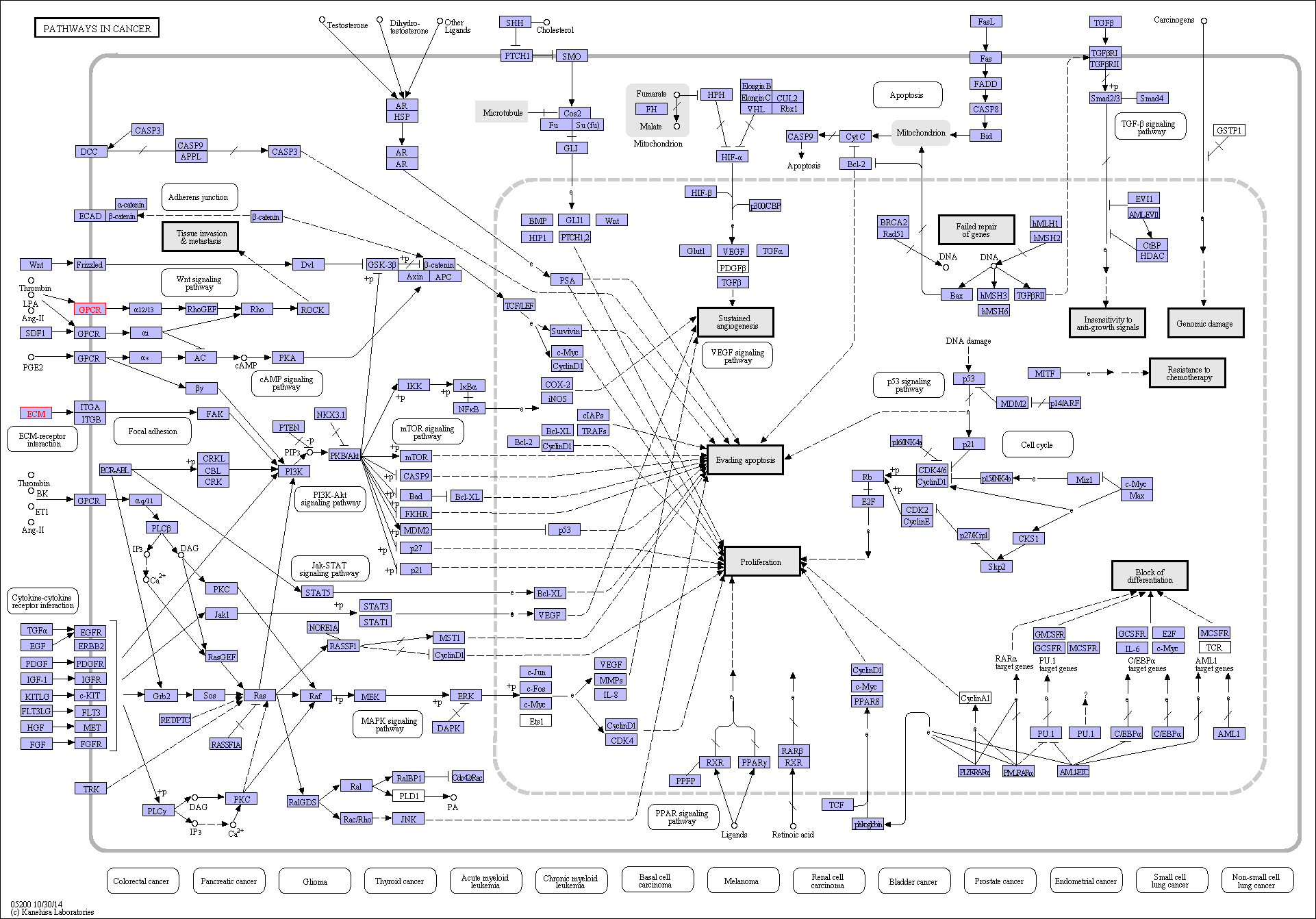

Supplement: Additional file 7: — KEGG pathways associated with genes in Table 1. Pathway image files downloaded from KEGG and the html file is linked to these images. (ZIP 963 kb) [file 12920_2016_196_MOESM7_ESM.zip › Kegg_Figs/ko05200.png]

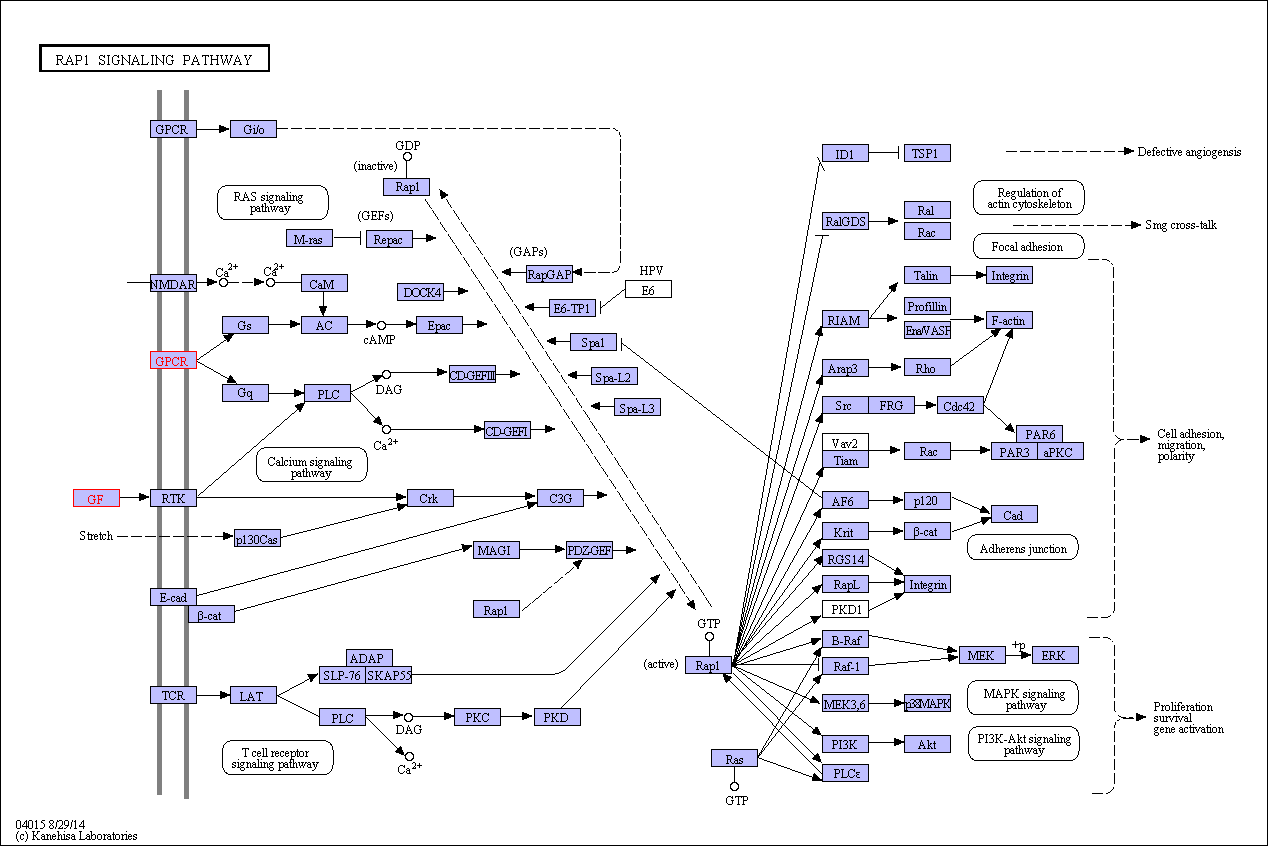

Supplement: Additional file 7: — KEGG pathways associated with genes in Table 1. Pathway image files downloaded from KEGG and the html file is linked to these images. (ZIP 963 kb) [file 12920_2016_196_MOESM7_ESM.zip › Kegg_Figs/ko04015.png]

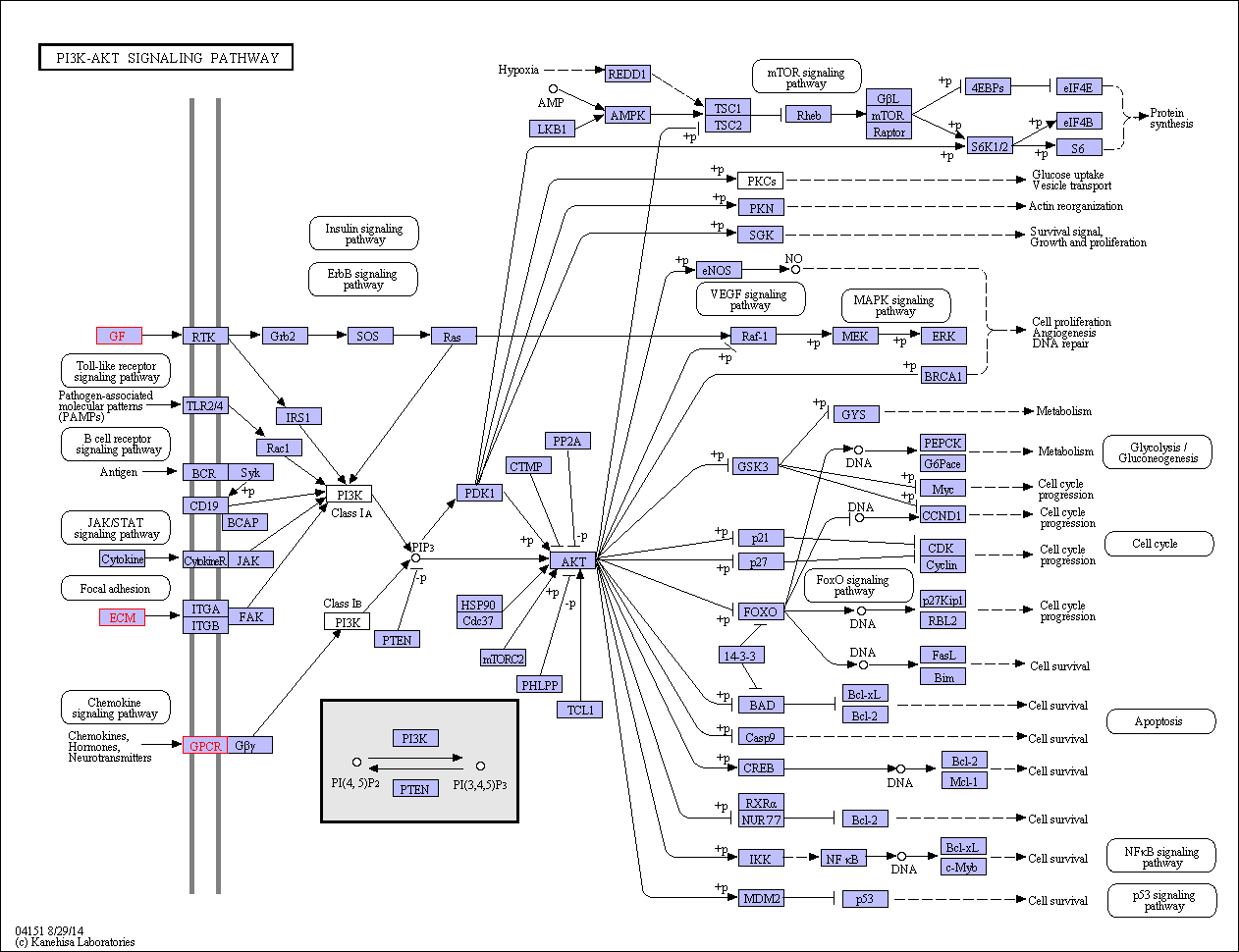

Supplement: Additional file 7: — KEGG pathways associated with genes in Table 1. Pathway image files downloaded from KEGG and the html file is linked to these images. (ZIP 963 kb) [file 12920_2016_196_MOESM7_ESM.zip › Kegg_Figs/ko04151.png]
