# Supplementary material for: SFRP1 is a possible candidate for epigenetic therapy in non-small cell lung cancer
Source: BMC Med Genomics. 2016 Aug 12;9(Suppl 1):28. doi: 10.1186/s12920-016-0196-3 (PMC4989892; doi:10.1186/s12920-016-0196-3)
Supplement: Additional file 2: — Hierarchical clustering with 23 samples. Hierarchical clustering between PCs with 23 samples. Left (right) column corresponds to those before (after) re-labelling. (PDF 77 kb) [file 12920_2016_196_MOESM2_ESM.pdf]

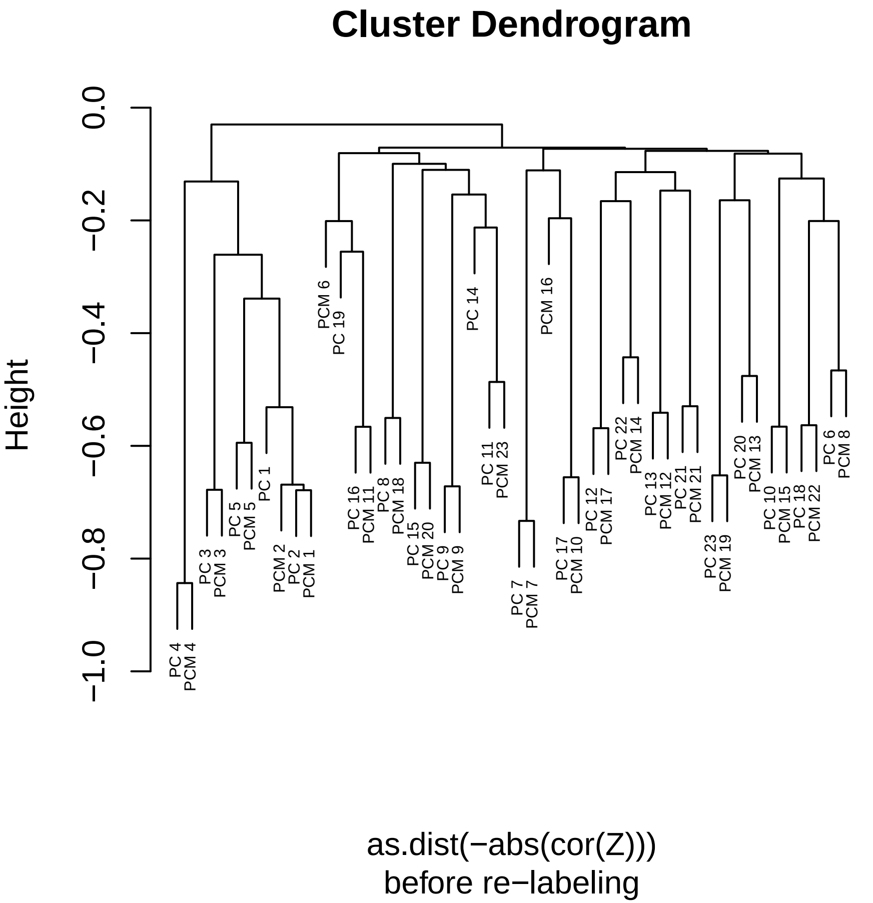
Cluster Dendrogram
0.0
−0.2
−0.4
−0.6
−0.8
−1.0
Height
PC 4
PCM 4
PC 3
PCM 3
PC 5
PCM 5
PC 1
PCM 2
PC 2
PCM 1
PCM 6
PC 19
PC 16
PCM 11
PC 8
PCM 18
PC 15
PCM 20
PC 9
PCM 9
PC 14
PC 11
PCM 23
PC 7
PCM 7
PCM 16
PC 17
PCM 10
PC 12
PCM 17
PC 22
PCM 14
PC 13
PCM 12
PC 21
PCM 21
PC 23
PCM 19
PC 20
PCM 13
PC 10
PCM 15
PC 18
PCM 22
PC 6
PCM 8
as.dist(−abs(cor(Z)))
before re−labeling

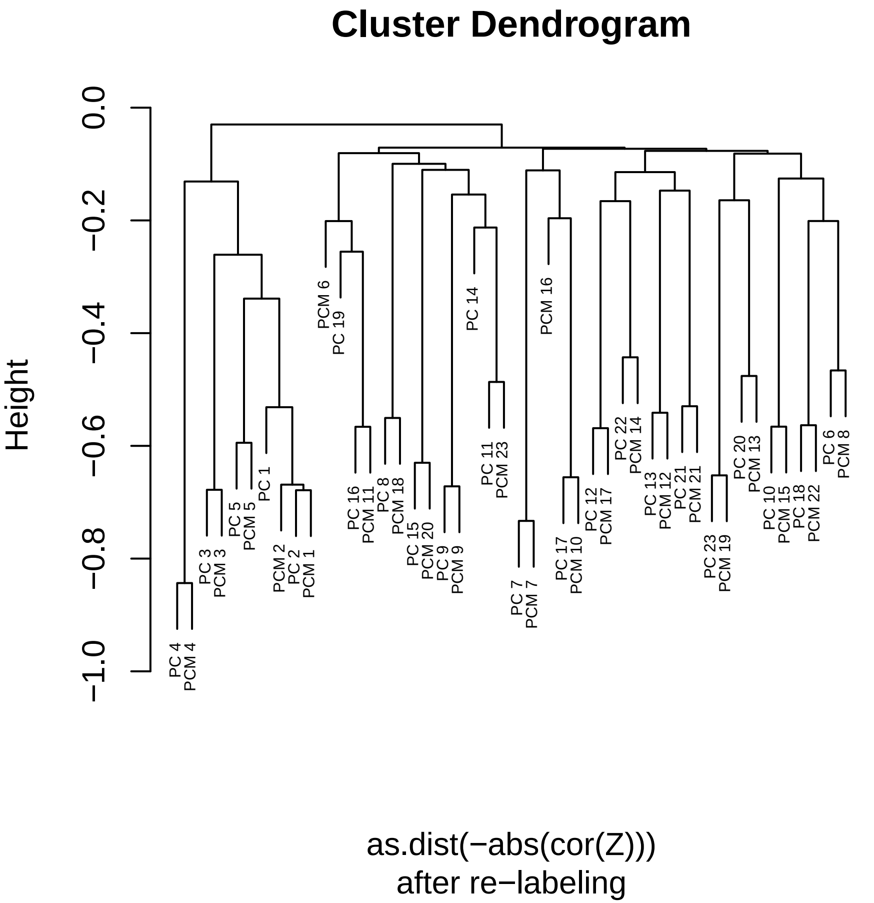
Cluster Dendrogram
0.0
−0.2
−0.4
−0.6
−0.8
−1.0
Height
PC 4
PCM 4
PC 3
PCM 3
PC 5
PCM 5
PC 1
PCM 2
PC 2
PCM 1
PCM 6
PC 19
PC 16
PCM 11
PC 8
PCM 18
PC 15
PCM 20
PC 9
PCM 9
PC 14
PC 11
PCM 23
PC 7
PCM 7
PCM 16
PC 17
PCM 10
PC 12
PCM 17
PC 22
PCM 14
PC 13
PCM 12
PC 21
PCM 21
PC 23
PCM 19
PC 20
PCM 13
PC 10
PCM 15
PC 18
PCM 22
PC 6
PCM 8
as.dist(−abs(cor(Z)))
after re−labeling

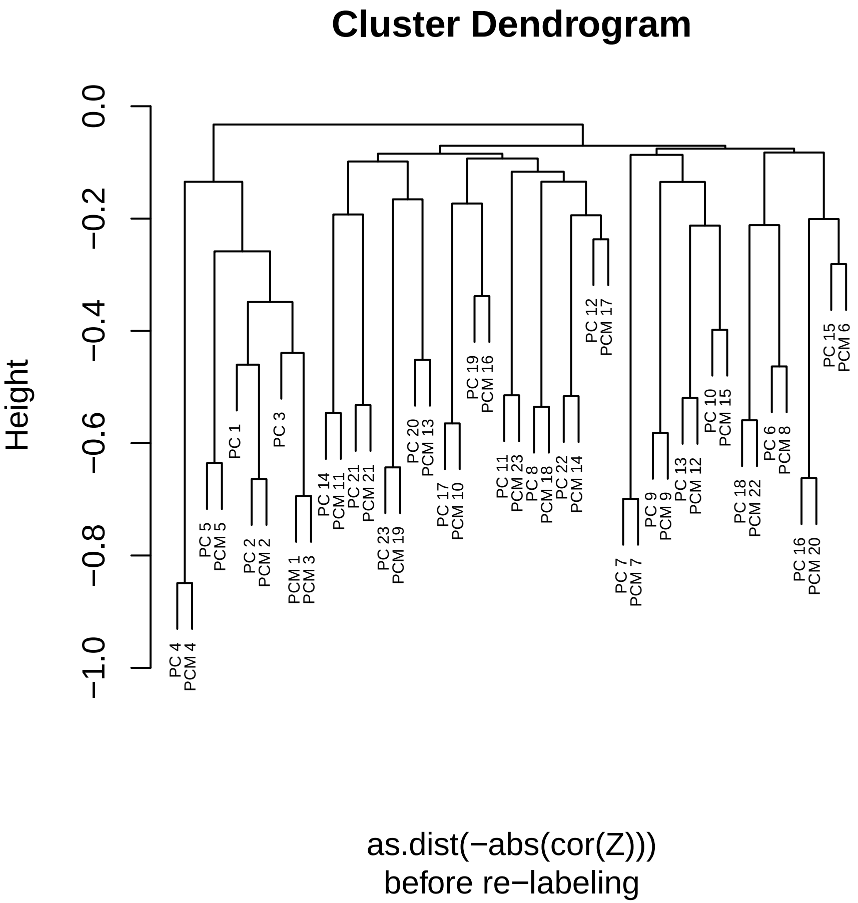
Cluster Dendrogram
Height
0.0
−0.2
−0.4
−0.6
−0.8
−1.0
PC 4 PCM 4
PC 5 PCM 5
PC 1
PC 2 PCM 2
PC 3
PCM 1 PCM 3
PC 14 PCM 11
PC 21 PCM 21
PC 23 PCM 19
PC 20 PCM 13
PC 17 PCM 10
PC 19 PCM 16
PC 11 PCM 23
PC 8 PCM 18
PC 22 PCM 14
PC 12 PCM 17
PC 7 PCM 7
PC 9 PCM 9
PC 13 PCM 12
PC 10 PCM 15
PC 18 PCM 22
PC 6 PCM 8
PC 16 PCM 20
PC 15 PCM 6
as.dist(−abs(cor(Z)))
before re−labeling

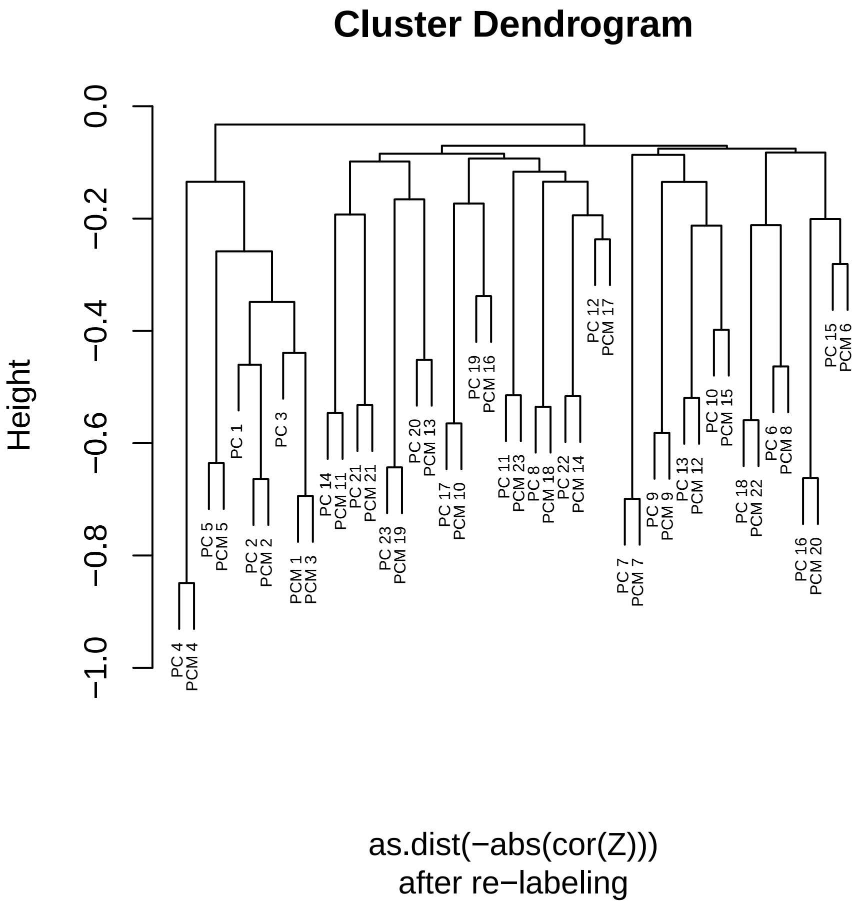
Cluster Dendrogram
Height
0.0
−0.2
−0.4
−0.6
−0.8
−1.0
PC 4 PCM 4
PC 5 PCM 5
PC 1
PC 2 PCM 2
PC 3
PCM 1 PCM 3
PC 14 PCM 11
PC 21 PCM 21
PC 23 PCM 19
PC 20 PCM 13
PC 17 PCM 10
PC 19 PCM 16
PC 11 PCM 23
PC 8 PCM 18
PC 22 PCM 14
PC 12 PCM 17
PC 7 PCM 7
PC 9 PCM 9
PC 13 PCM 12
PC 10 PCM 15
PC 18 PCM 22
PC 6 PCM 8
PC 16 PCM 20
PC 15 PCM 6
as.dist(−abs(cor(Z)))
after re−labeling

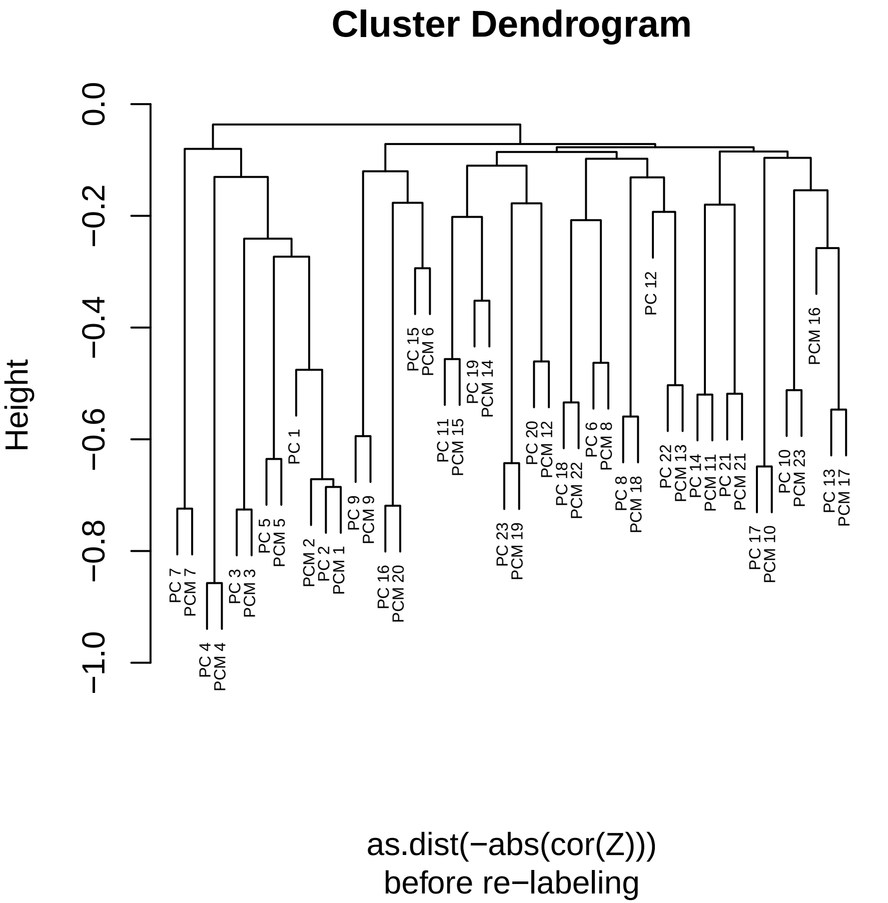
Cluster Dendrogram
Height
0.0
−0.2
−0.4
−0.6
−0.8
−1.0
PC 7
PCM 7
PC 4
PCM 4
PC 3
PCM 3
PC 5
PCM 5
PC 1
PCM 2
PC 2
PCM 1
PC 9
PCM 9
PC 16
PCM 20
PC 15
PCM 6
PC 11
PCM 15
PC 19
PCM 14
PC 23
PCM 19
PC 20
PCM 12
PC 18
PCM 22
PC 6
PCM 8
PC 8
PCM 18
PC 12
PC 22
PCM 13
PC 14
PCM 11
PC 21
PCM 21
PC 17
PCM 10
PC 10
PCM 23
PCM 16
PC 13
PCM 17
as.dist(−abs(cor(Z)))
before re−labeling

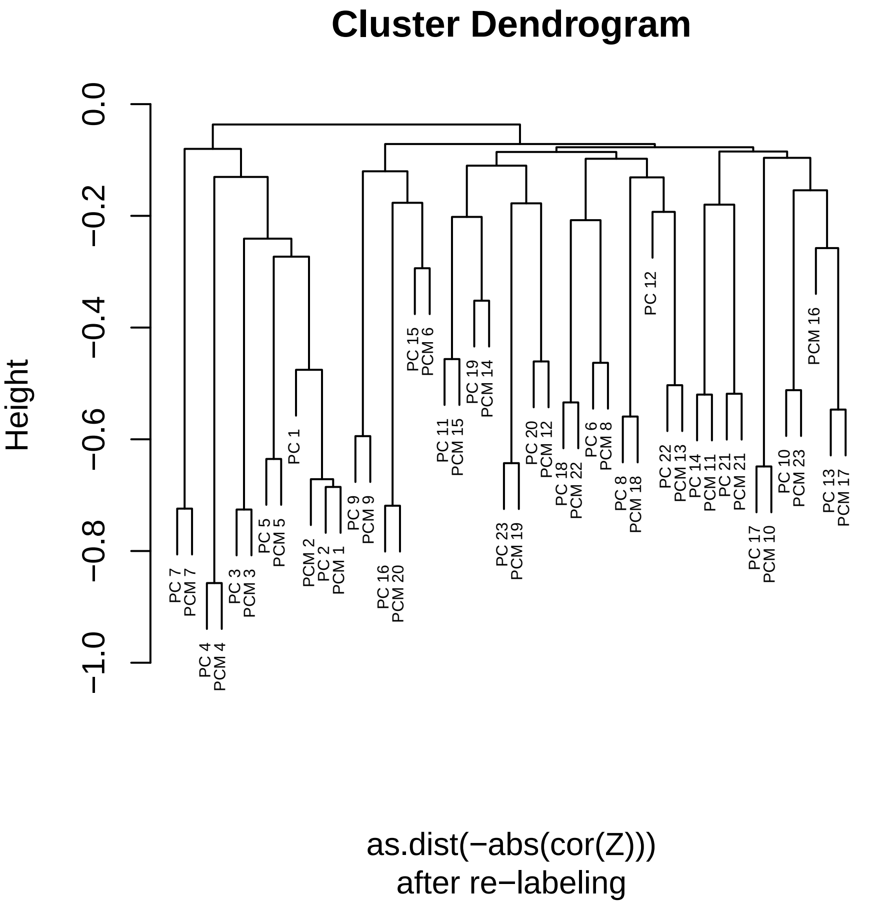
Cluster Dendrogram
Height
0.0
−0.2
−0.4
−0.6
−0.8
−1.0
PC 7
PCM 7
PC 4
PCM 4
PC 3
PCM 3
PC 5
PCM 5
PC 1
PCM 2
PC 2
PCM 1
PC 9
PCM 9
PC 16
PCM 20
PC 15
PCM 6
PC 11
PCM 15
PC 19
PCM 14
PC 23
PCM 19
PC 20
PCM 12
PC 18
PCM 22
PC 6
PCM 8
PC 8
PCM 18
PC 12
PC 22
PCM 13
PC 14
PCM 11
PC 21
PCM 21
PC 17
PCM 10
PC 10
PCM 23
PCM 16
PC 13
PCM 17
as.dist(−abs(cor(Z)))
after re−labeling

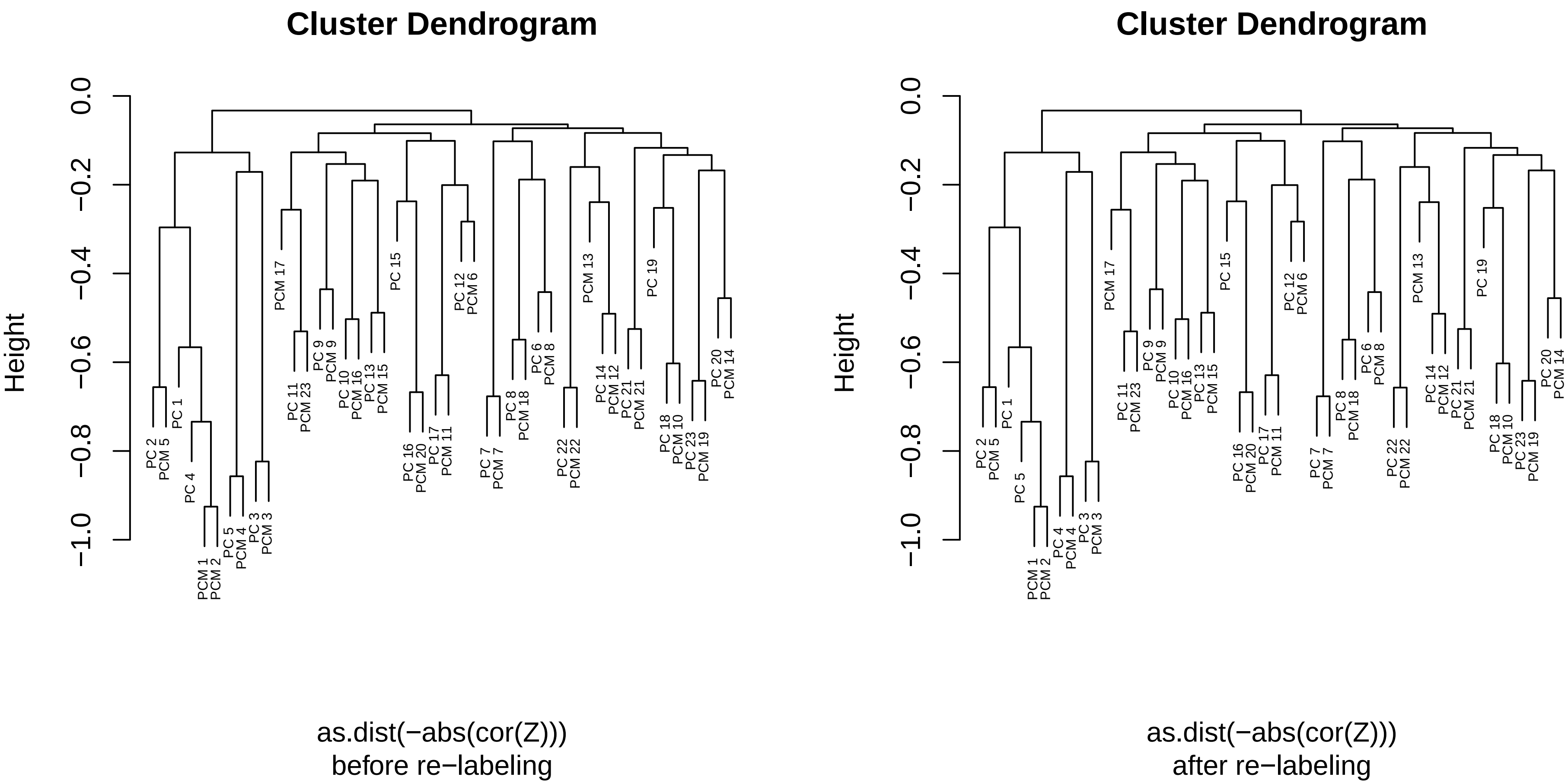
Cluster Dendrogram
Height
0.0
−0.2
−0.4
−0.6
−0.8
−1.0
PC 2
PCM 5
PC 1
PC 4
PCM 1
PCM 2
PC 5
PCM 4
PC 3
PCM 3
PCM 17
PC 11
PCM 23
PC 9
PCM 9
PC 10
PCM 16
PC 13
PCM 15
PC 15
PC 16
PCM 20
PC 17
PCM 11
PC 12
PCM 6
PC 7
PCM 7
PC 8
PCM 18
PC 6
PCM 8
PC 22
PCM 22
PCM 13
PC 14
PCM 12
PC 21
PCM 21
PC 19
PC 18
PCM 10
PC 23
PCM 19
PC 20
PCM 14
as.dist(−abs(cor(Z)))
before re−labeling
Cluster Dendrogram
Height
0.0
−0.2
−0.4
−0.6
−0.8
−1.0
PC 2
PCM 5
PC 1
PC 5
PCM 1
PCM 2
PC 4
PCM 4
PC 3
PCM 3
PCM 17
PC 11
PCM 23
PC 9
PCM 9
PC 10
PCM 16
PC 13
PCM 15
PC 15
PC 16
PCM 20
PC 17
PCM 11
PC 12
PCM 6
PC 7
PCM 7
PC 8
PCM 18
PC 6
PCM 8
PC 22
PCM 22
PCM 13
PC 14
PCM 12
PC 21
PCM 21
PC 19
PC 18
PCM 10
PC 23
PCM 19
PC 20
PCM 14
as.dist(−abs(cor(Z)))
after re−labeling

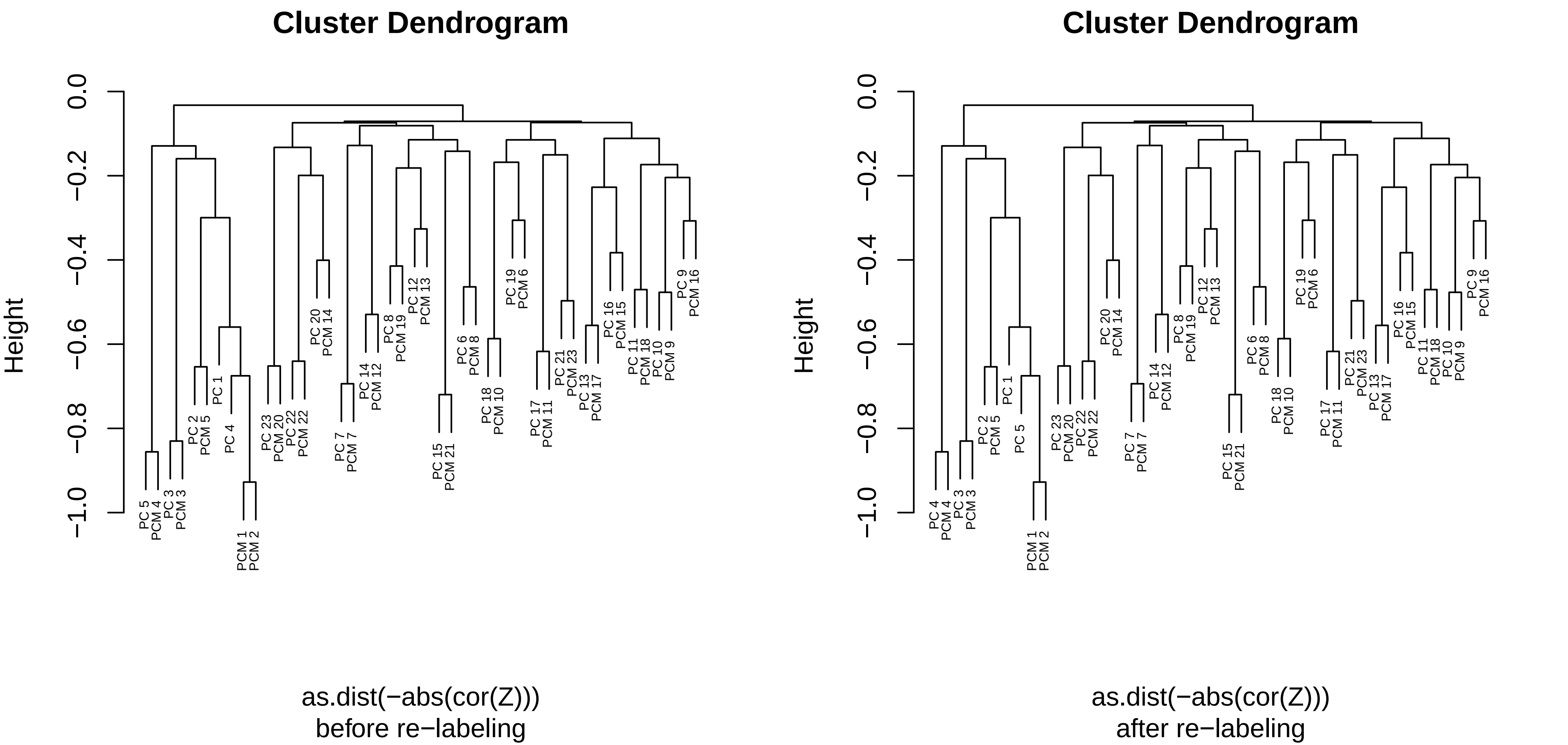
Cluster Dendrogram
Height
0.0
−0.2
−0.4
−0.6
−0.8
−1.0
PC 5
PCM 4
PC 3
PCM 3
PC 2
PCM 5
PC 1
PC 4
PCM 1
PCM 2
PC 23
PCM 20
PC 22
PCM 22
PC 20
PCM 14
PC 7
PCM 7
PC 14
PCM 12
PC 8
PCM 19
PC 12
PCM 13
PC 15
PCM 21
PC 6
PCM 8
PC 18
PCM 10
PC 19
PCM 6
PC 17
PCM 11
PC 21
PCM 23
PC 13
PCM 17
PC 16
PCM 15
PC 11
PCM 18
PC 10
PCM 9
PC 9
PCM 16
as.dist(−abs(cor(Z)))
before re−labeling
Cluster Dendrogram
Height
0.0
−0.2
−0.4
−0.6
−0.8
−1.0
PC 4
PCM 4
PC 3
PCM 3
PC 2
PCM 5
PC 1
PC 5
PCM 1
PCM 2
PC 23
PCM 20
PC 22
PCM 22
PC 20
PCM 14
PC 7
PCM 7
PC 14
PCM 12
PC 8
PCM 19
PC 12
PCM 13
PC 15
PCM 21
PC 6
PCM 8
PC 18
PCM 10
PC 19
PCM 6
PC 17
PCM 11
PC 21
PCM 23
PC 13
PCM 17
PC 16
PCM 15
PC 11
PCM 18
PC 10
PCM 9
PC 9
PCM 16
as.dist(−abs(cor(Z)))
after re−labeling

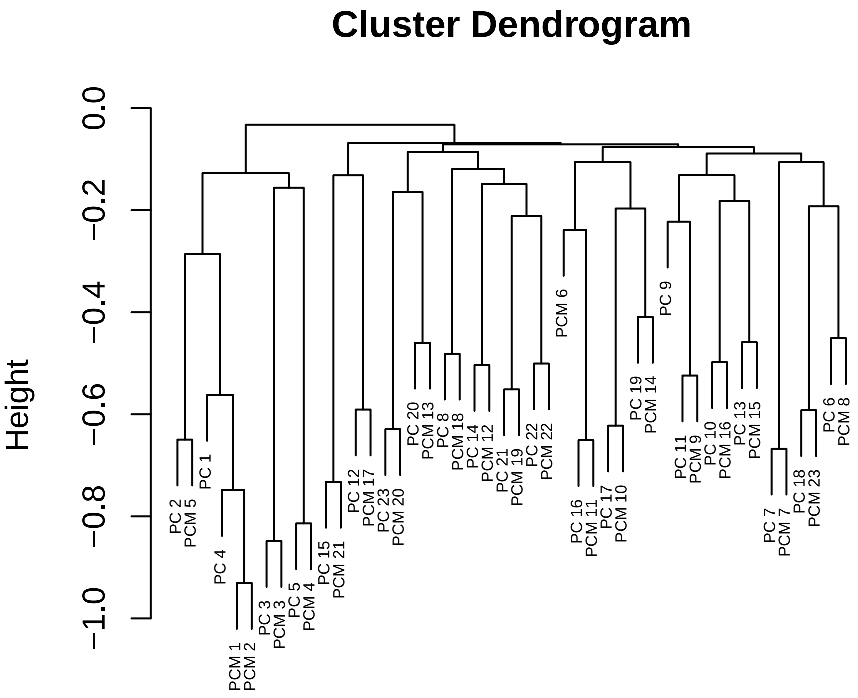

as.dist(−abs(cor(Z)))
before re−labeling

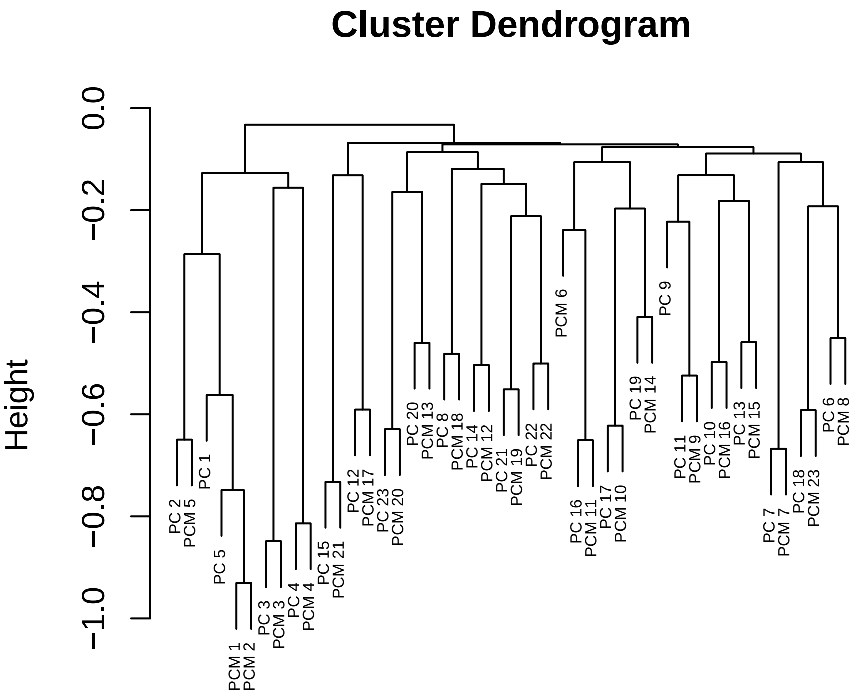

as.dist(−abs(cor(Z)))
after re−labeling

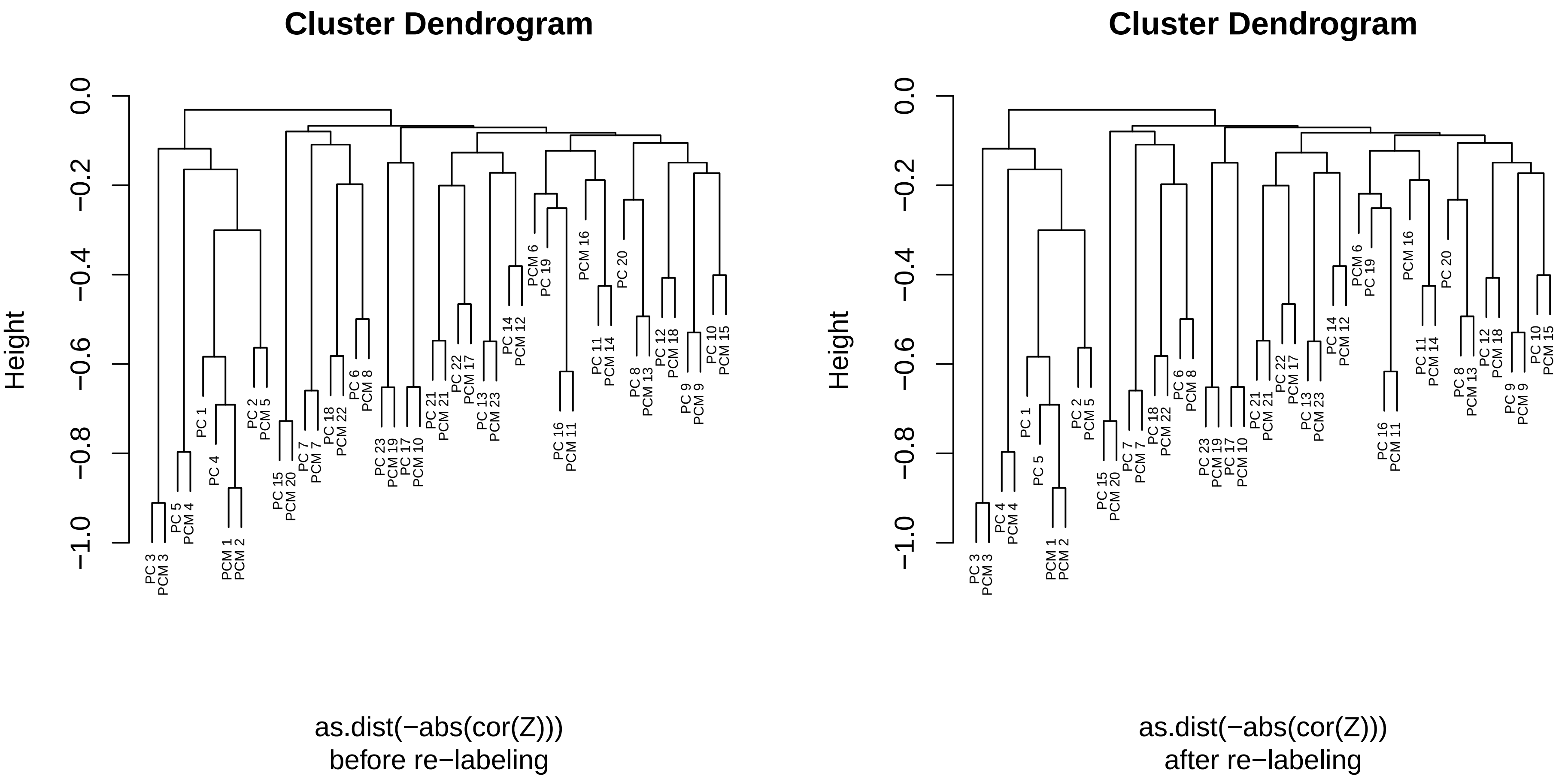
Cluster Dendrogram
Height
0.0
−0.2
−0.4
−0.6
−0.8
−1.0
PC 3 PCM 3 PC 5 PCM 4 PC 1 PC 4 PCM 1 PCM 2 PC 2 PCM 5 PC 15 PCM 20 PC 7 PCM 7 PC 18 PCM 22 PC 6 PCM 8 PC 23 PCM 19 PC 17 PCM 10 PC 21 PCM 21 PC 22 PCM 17 PC 13 PCM 23 PC 14 PCM 12 PCM 6 PC 19 PC 16 PCM 11 PCM 16 PC 11 PCM 14 PC 20 PC 8 PCM 13 PC 12 PCM 18 PC 9 PCM 9 PC 10 PCM 15
as.dist(−abs(cor(Z)))
before re−labeling
Cluster Dendrogram
Height
0.0
−0.2
−0.4
−0.6
−0.8
−1.0
PC 3 PCM 3 PC 4 PCM 4 PC 1 PC 5 PCM 1 PCM 2 PC 2 PCM 5 PC 15 PCM 20 PC 7 PCM 7 PC 18 PCM 22 PC 6 PCM 8 PC 23 PCM 19 PC 17 PCM 10 PC 21 PCM 21 PC 22 PCM 17 PC 13 PCM 23 PC 14 PCM 12 PCM 6 PC 19 PC 16 PCM 11 PCM 16 PC 11 PCM 14 PC 20 PC 8 PCM 13 PC 12 PCM 18 PC 9 PCM 9 PC 10 PCM 15
as.dist(−abs(cor(Z)))
after re−labeling

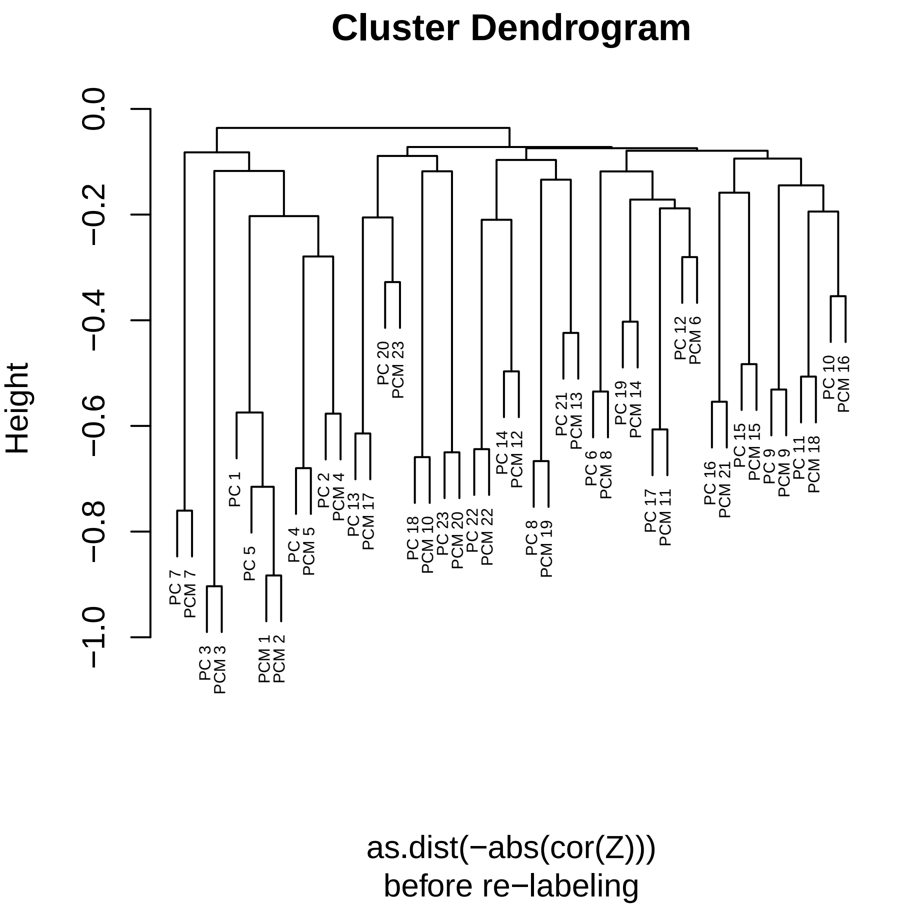
Cluster Dendrogram
Height
0.0
−0.2
−0.4
−0.6
−0.8
−1.0
PC 7
PCM 7
PC 3
PCM 3
PC 1
PC 5
PCM 1
PCM 2
PC 4
PCM 5
PC 2
PCM 4
PC 13
PCM 17
PC 20
PCM 23
PC 18
PCM 10
PC 23
PCM 20
PC 22
PCM 22
PC 14
PCM 12
PC 8
PCM 19
PC 21
PCM 13
PC 6
PCM 8
PC 19
PCM 14
PC 17
PCM 11
PC 12
PCM 6
PC 16
PCM 21
PC 15
PCM 15
PC 9
PCM 9
PC 11
PCM 18
PC 10
PCM 16
as.dist(−abs(cor(Z)))
before re−labeling

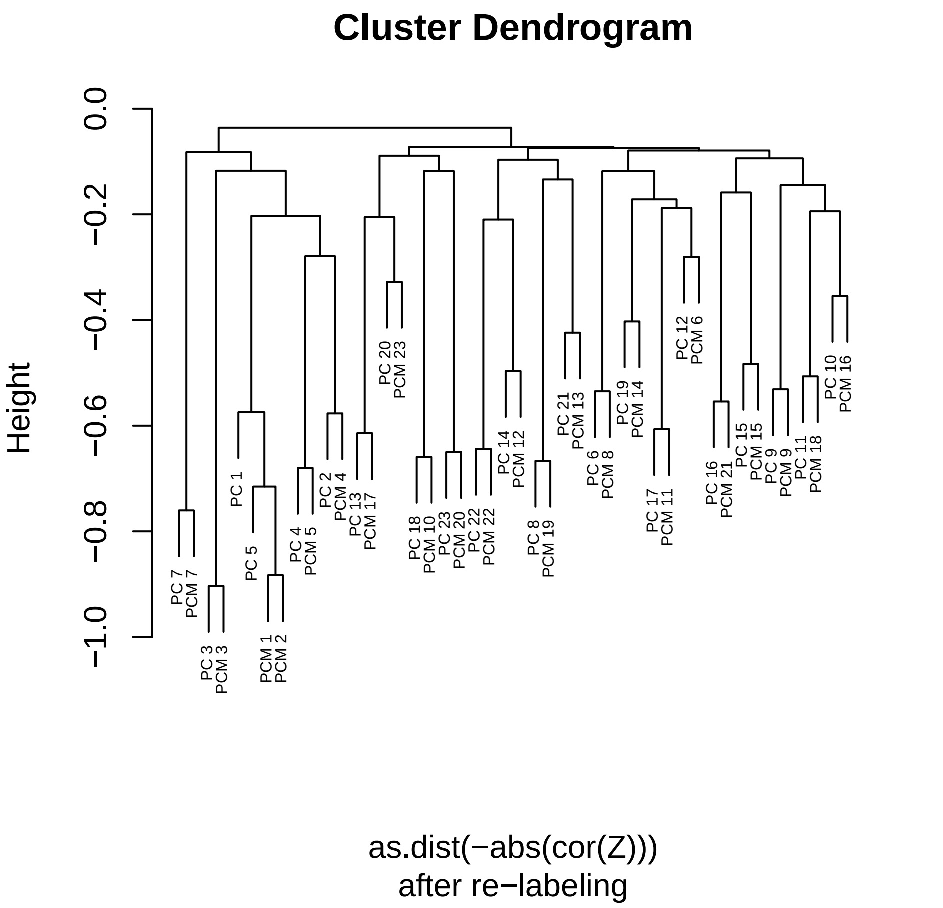
Cluster Dendrogram
Height
0.0
−0.2
−0.4
−0.6
−0.8
−1.0
PC 7
PCM 7
PC 3
PCM 3
PC 1
PC 5
PCM 1
PCM 2
PC 4
PCM 5
PC 2
PCM 4
PC 13
PCM 17
PC 20
PCM 23
PC 18
PCM 10
PC 23
PCM 20
PC 22
PCM 22
PC 14
PCM 12
PC 8
PCM 19
PC 21
PCM 13
PC 6
PCM 8
PC 19
PCM 14
PC 17
PCM 11
PC 12
PCM 6
PC 16
PCM 21
PC 15
PCM 15
PC 9
PCM 9
PC 11
PCM 18
PC 10
PCM 16
as.dist(−abs(cor(Z)))
after re−labeling

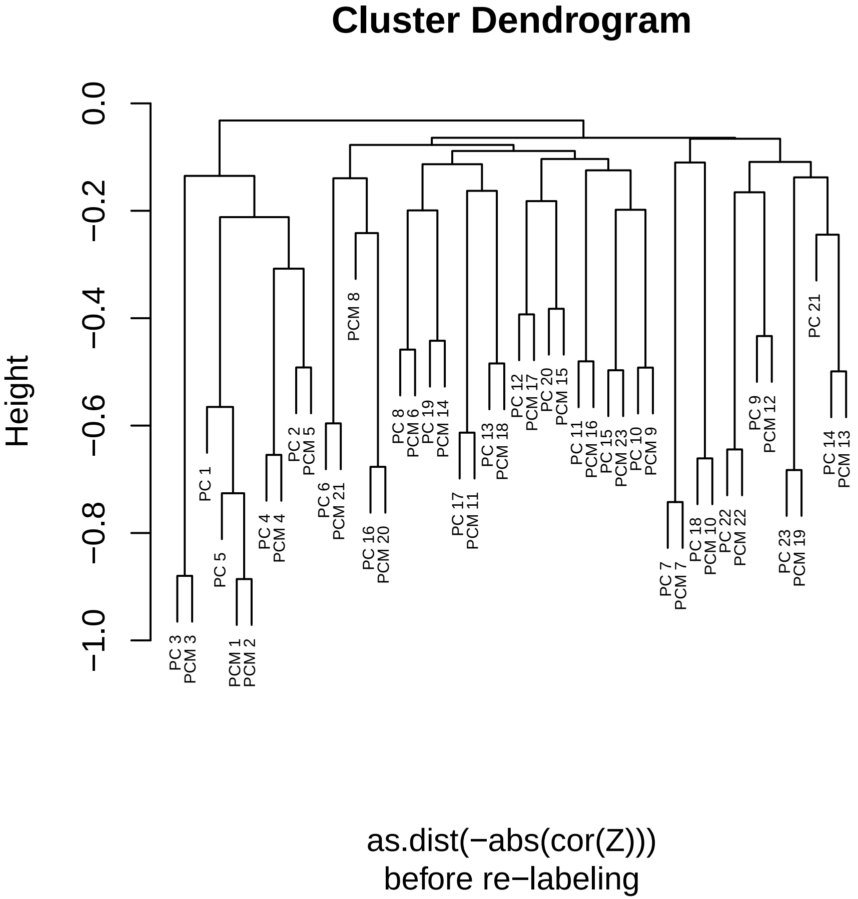
Cluster Dendrogram
Height
0.0
−0.2
−0.4
−0.6
−0.8
−1.0
PC 3
PCM 3
PC 1
PC 5
PCM 1
PCM 2
PC 4
PCM 4
PC 2
PCM 5
PC 6
PCM 21
PCM 8
PC 16
PCM 20
PC 8
PCM 6
PC 19
PCM 14
PC 17
PCM 11
PC 13
PCM 18
PC 12
PCM 17
PC 20
PCM 15
PC 11
PCM 16
PC 15
PCM 23
PC 10
PCM 9
PC 7
PCM 7
PC 18
PCM 10
PC 22
PCM 22
PC 9
PCM 12
PC 23
PCM 19
PC 21
PC 14
PCM 13
as.dist(−abs(cor(Z)))
before re−labeling

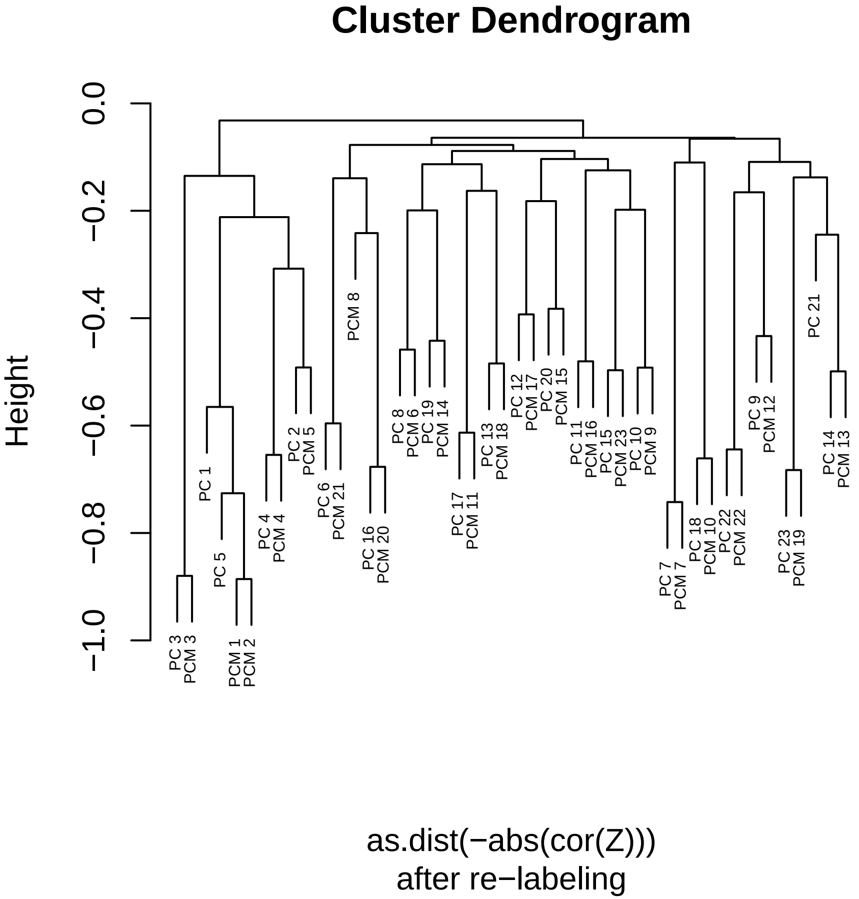
Cluster Dendrogram
Height
0.0
−0.2
−0.4
−0.6
−0.8
−1.0
PC 3
PCM 3
PC 1
PC 5
PCM 1
PCM 2
PC 4
PCM 4
PC 2
PCM 5
PC 6
PCM 21
PCM 8
PC 16
PCM 20
PC 8
PCM 6
PC 19
PCM 14
PC 17
PCM 11
PC 13
PCM 18
PC 12
PCM 17
PC 20
PCM 15
PC 11
PCM 16
PC 15
PCM 23
PC 10
PCM 9
PC 7
PCM 7
PC 18
PCM 10
PC 22
PCM 22
PC 9
PCM 12
PC 23
PCM 19
PC 21
PC 14
PCM 13
as.dist(−abs(cor(Z)))
after re−labeling

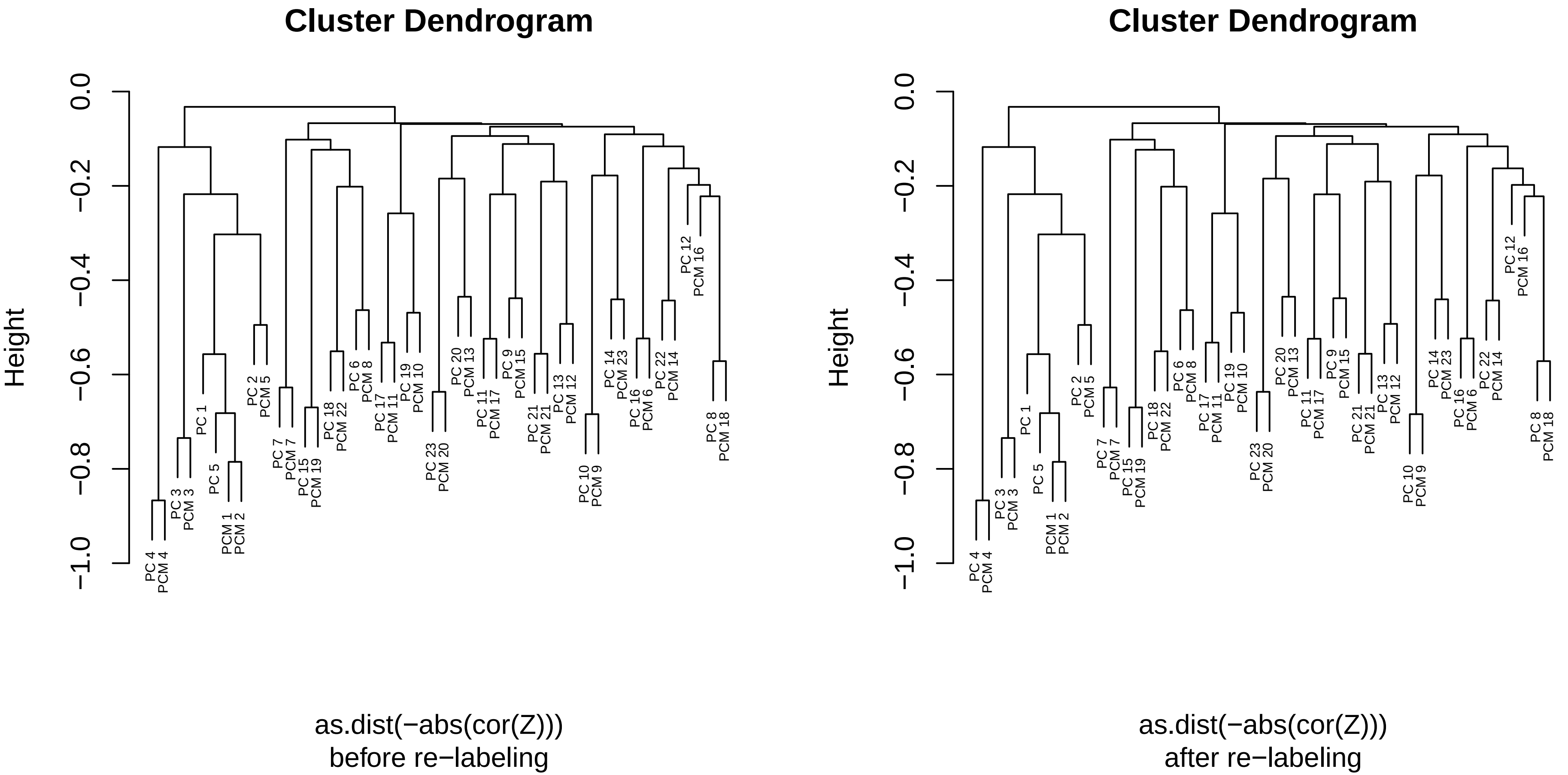
Cluster Dendrogram
Height
0.0
−0.2
−0.4
−0.6
−0.8
−1.0
PC 4
PCM 4
PC 3
PCM 3
PC 1
PC 5
PCM 1
PCM 2
PC 2
PCM 5
PC 7
PCM 7
PC 15
PCM 19
PC 18
PCM 22
PC 6
PCM 8
PC 17
PCM 11
PC 19
PCM 10
PC 23
PCM 20
PC 20
PCM 13
PC 11
PCM 17
PC 9
PCM 15
PC 21
PCM 21
PC 13
PCM 12
PC 10
PCM 9
PC 14
PCM 23
PC 16
PCM 6
PC 22
PCM 14
PC 12
PCM 16
PC 8
PCM 18
as.dist(−abs(cor(Z)))
before re−labeling
Cluster Dendrogram
Height
0.0
−0.2
−0.4
−0.6
−0.8
−1.0
PC 4
PCM 4
PC 3
PCM 3
PC 1
PC 5
PCM 1
PCM 2
PC 2
PCM 5
PC 7
PCM 7
PC 15
PCM 19
PC 18
PCM 22
PC 6
PCM 8
PC 17
PCM 11
PC 19
PCM 10
PC 23
PCM 20
PC 20
PCM 13
PC 11
PCM 17
PC 9
PCM 15
PC 21
PCM 21
PC 13
PCM 12
PC 10
PCM 9
PC 14
PCM 23
PC 16
PCM 6
PC 22
PCM 14
PC 12
PCM 16
PC 8
PCM 18
as.dist(−abs(cor(Z)))
after re−labeling

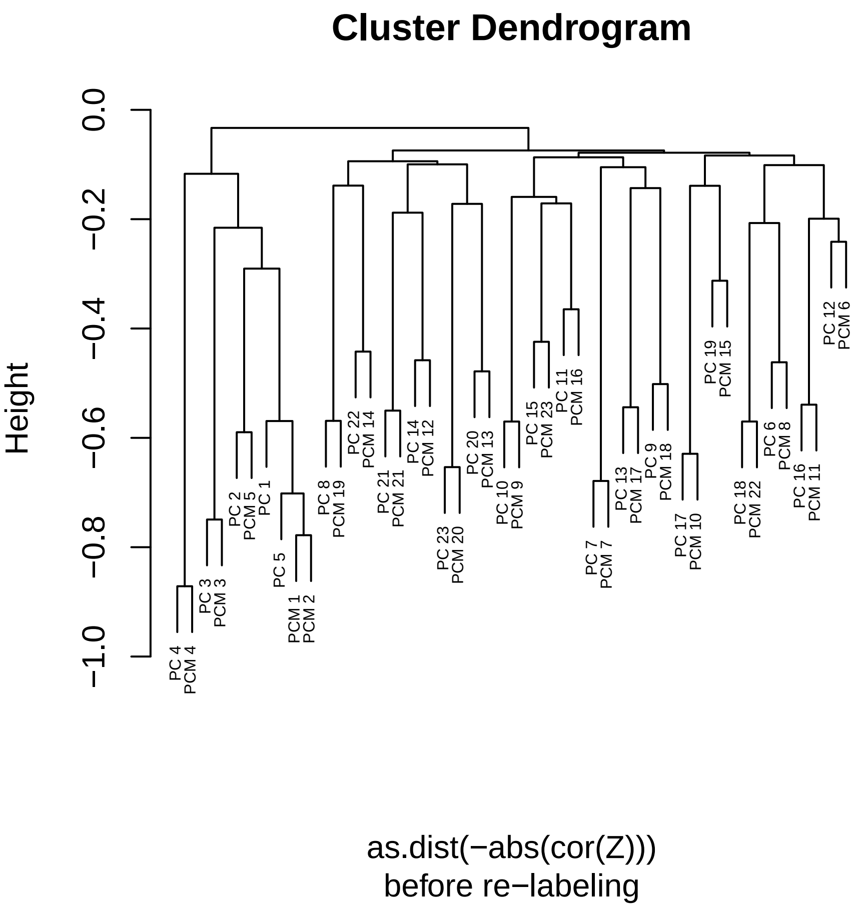
Cluster Dendrogram
Height
0.0
−0.2
−0.4
−0.6
−0.8
−1.0
PC 4
PCM 4
PC 3
PCM 3
PC 2
PCM 5
PC 1
PC 5
PCM 1
PCM 2
PC 8
PCM 19
PC 22
PCM 14
PC 21
PCM 21
PC 14
PCM 12
PC 23
PCM 20
PC 20
PCM 13
PC 10
PCM 9
PC 15
PCM 23
PC 11
PCM 16
PC 7
PCM 7
PC 13
PCM 17
PC 9
PCM 18
PC 17
PCM 10
PC 19
PCM 15
PC 18
PCM 22
PC 6
PCM 8
PC 16
PCM 11
PC 12
PCM 6
as.dist(−abs(cor(Z)))
before re−labeling

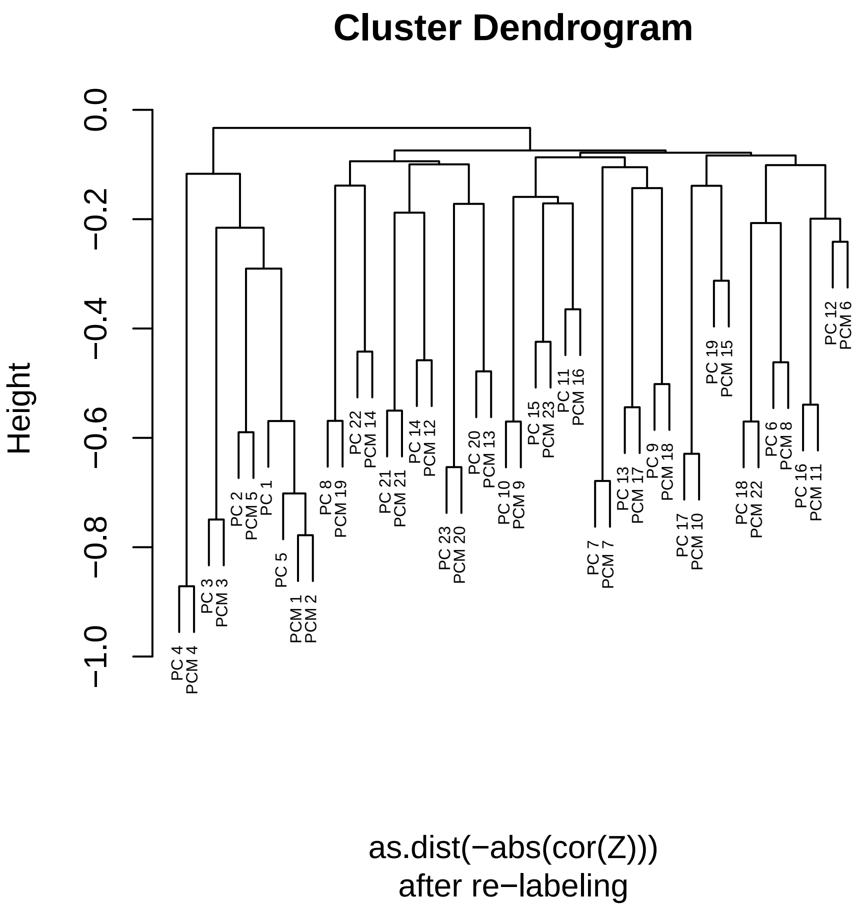
Cluster Dendrogram
Height
0.0
−0.2
−0.4
−0.6
−0.8
−1.0
PC 4
PCM 4
PC 3
PCM 3
PC 2
PCM 5
PC 1
PC 5
PCM 1
PCM 2
PC 8
PCM 19
PC 22
PCM 14
PC 21
PCM 21
PC 14
PCM 12
PC 23
PCM 20
PC 20
PCM 13
PC 10
PCM 9
PC 15
PCM 23
PC 11
PCM 16
PC 7
PCM 7
PC 13
PCM 17
PC 9
PCM 18
PC 17
PCM 10
PC 19
PCM 15
PC 18
PCM 22
PC 6
PCM 8
PC 16
PCM 11
PC 12
PCM 6
as.dist(−abs(cor(Z)))
after re−labeling

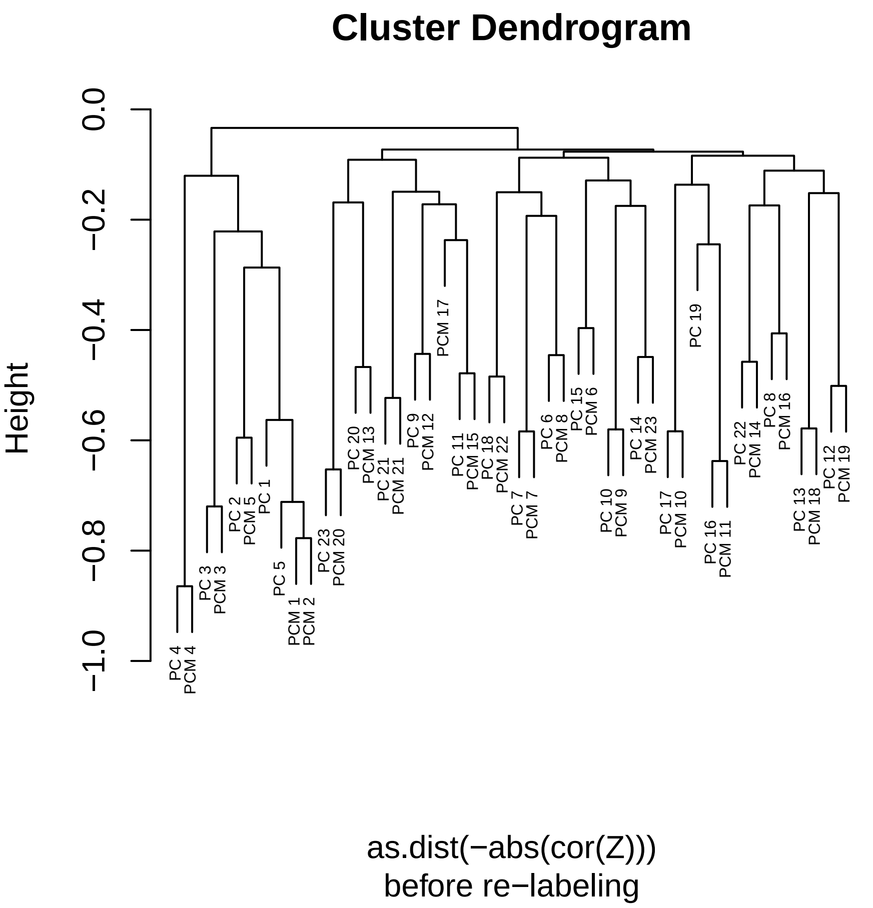

Cluster Dendrogram
Height
0.0
−0.2
−0.4
−0.6
−0.8
−1.0
PC 4
PCM 4
PC 3
PCM 3
PC 2
PCM 5
PC 1
PC 5
PCM 1
PCM 2
PC 23
PCM 20
PC 20
PCM 13
PC 21
PCM 21
PC 9
PCM 12
PCM 17
PC 11
PCM 15
PC 18
PCM 22
PC 7
PCM 7
PC 6
PCM 8
PC 15
PCM 6
PC 10
PCM 9
PC 14
PCM 23
PC 17
PCM 10
PC 19
PC 16
PCM 11
PC 22
PCM 14
PC 8
PCM 16
PC 13
PCM 18
PC 12
PCM 19
as.dist(−abs(cor(Z)))
before re−labeling

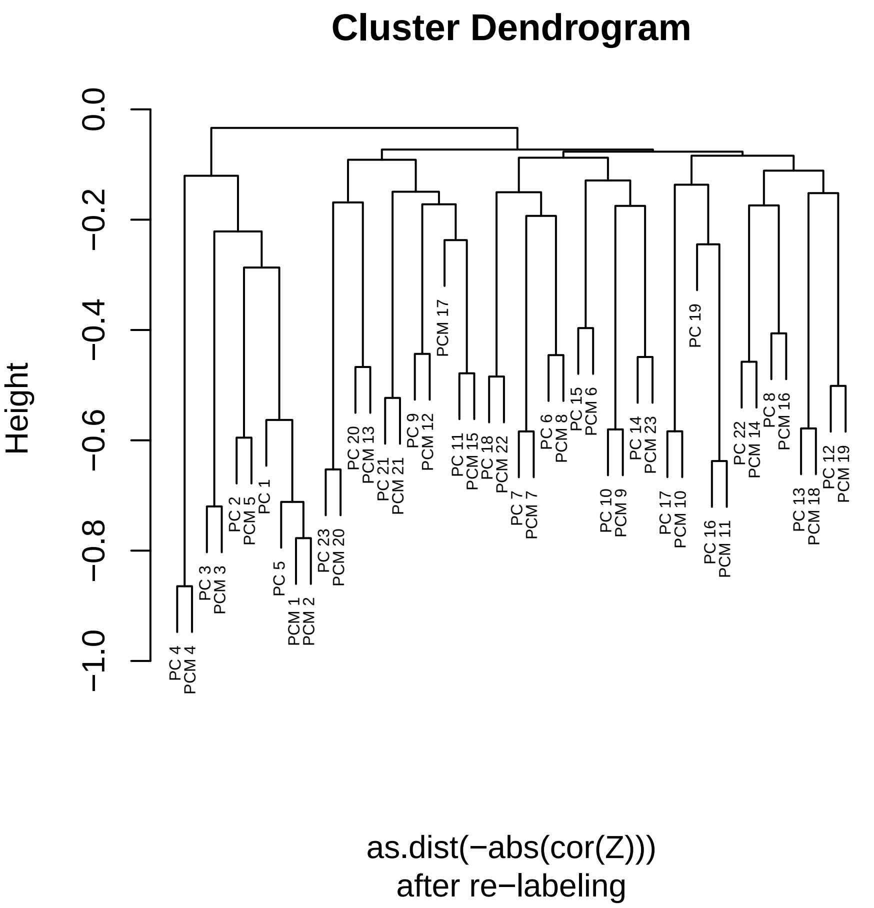

Cluster Dendrogram
Height
0.0
−0.2
−0.4
−0.6
−0.8
−1.0
PC 4
PCM 4
PC 3
PCM 3
PC 2
PCM 5
PC 1
PC 5
PCM 1
PCM 2
PC 23
PCM 20
PC 20
PCM 13
PC 21
PCM 21
PC 9
PCM 12
PCM 17
PC 11
PCM 15
PC 18
PCM 22
PC 7
PCM 7
PC 6
PCM 8
PC 15
PCM 6
PC 10
PCM 9
PC 14
PCM 23
PC 17
PCM 10
PC 19
PC 16
PCM 11
PC 22
PCM 14
PC 8
PCM 16
PC 13
PCM 18
PC 12
PCM 19
as.dist(−abs(cor(Z)))
after re−labeling

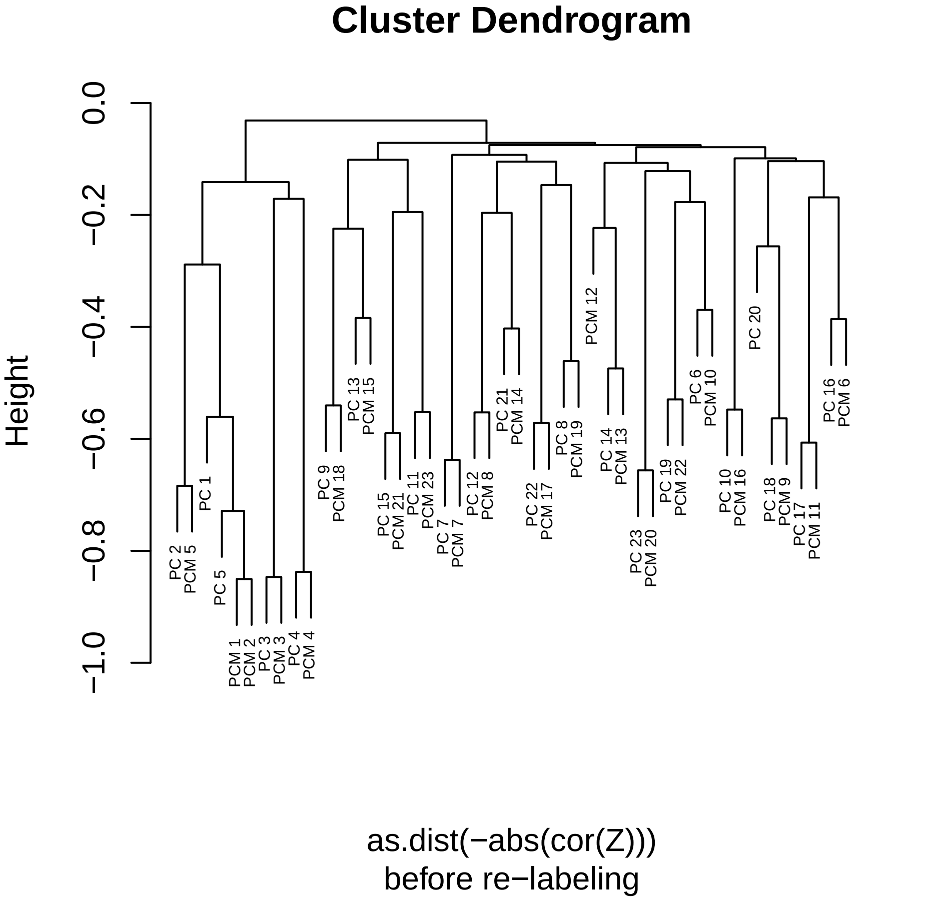
Cluster Dendrogram
Height
0.0
−0.2
−0.4
−0.6
−0.8
−1.0
PC 2
PCM 5
PC 1
PC 5
PCM 1
PCM 2
PC 3
PCM 3
PC 4
PCM 4
PC 9
PCM 18
PC 13
PCM 15
PC 15
PCM 21
PC 11
PCM 23
PC 7
PCM 7
PC 12
PCM 8
PC 21
PCM 14
PC 22
PCM 17
PC 8
PCM 19
PCM 12
PC 14
PCM 13
PC 23
PCM 20
PC 19
PCM 22
PC 6
PCM 10
PC 10
PCM 16
PC 20
PC 18
PCM 9
PC 17
PCM 11
PC 16
PCM 6
as.dist(−abs(cor(Z)))
before re−labeling

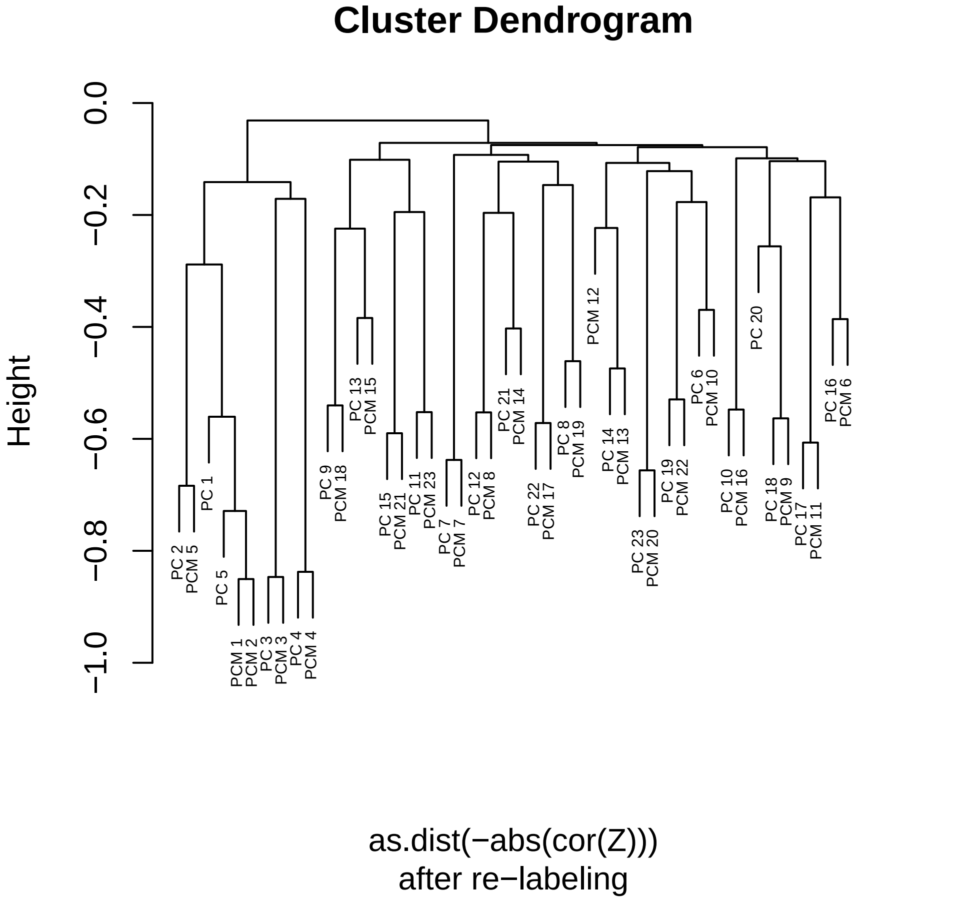
Cluster Dendrogram
Height
0.0
−0.2
−0.4
−0.6
−0.8
−1.0
PC 2
PCM 5
PC 1
PC 5
PCM 1
PCM 2
PC 3
PCM 3
PC 4
PCM 4
PC 9
PCM 18
PC 13
PCM 15
PC 15
PCM 21
PC 11
PCM 23
PC 7
PCM 7
PC 12
PCM 8
PC 21
PCM 14
PC 22
PCM 17
PC 8
PCM 19
PCM 12
PC 14
PCM 13
PC 23
PCM 20
PC 19
PCM 22
PC 6
PCM 10
PC 10
PCM 16
PC 20
PC 18
PCM 9
PC 17
PCM 11
PC 16
PCM 6
as.dist(−abs(cor(Z)))
after re−labeling

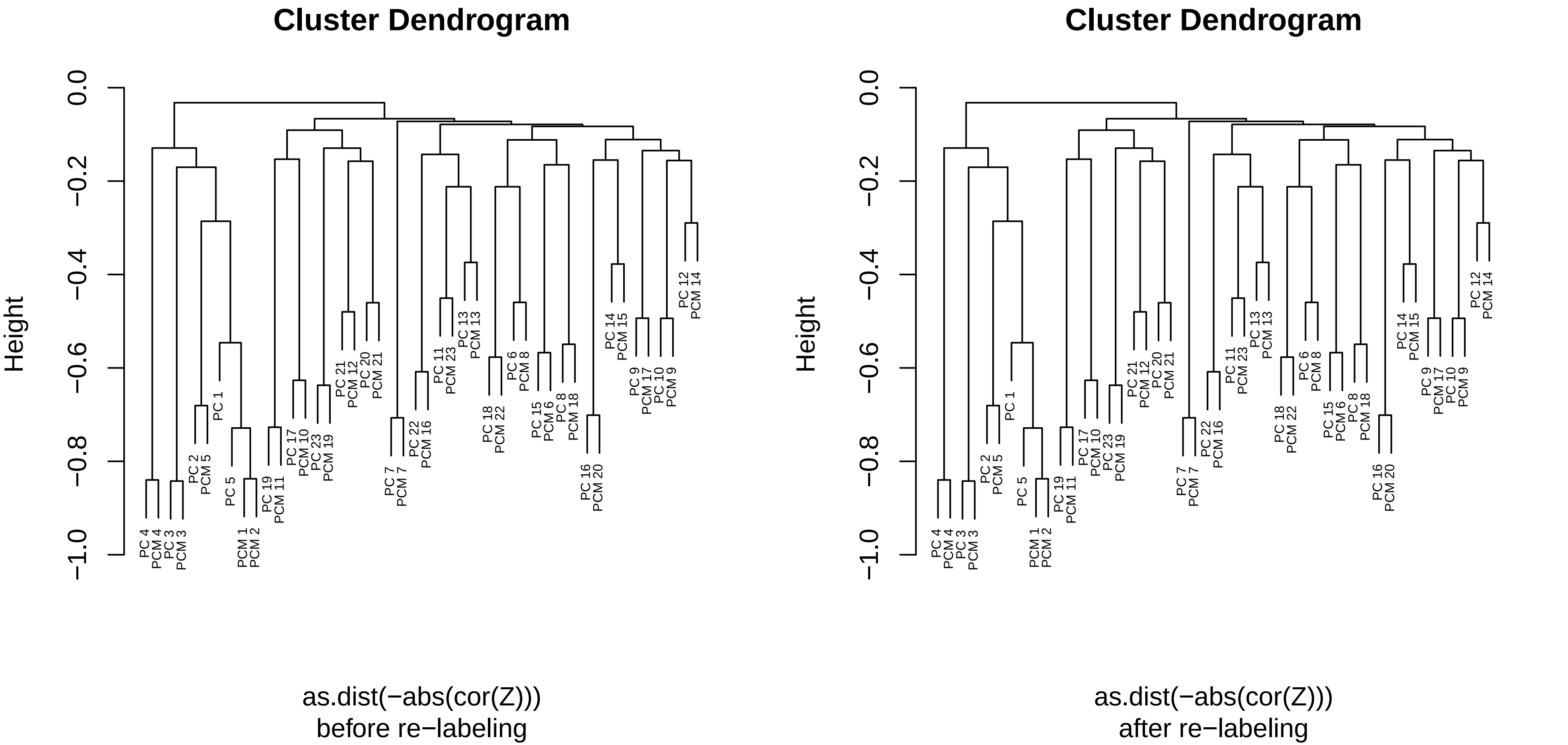
Cluster Dendrogram
Height
0.0
−0.2
−0.4
−0.6
−0.8
−1.0
PC 4
PCM 4
PC 3
PCM 3
PC 2
PCM 5
PC 1
PC 5
PCM 1
PCM 2
PC 19
PCM 11
PC 17
PCM 10
PC 23
PCM 19
PC 21
PCM 12
PC 20
PCM 21
PC 7
PCM 7
PC 22
PCM 16
PC 11
PCM 23
PC 13
PCM 13
PC 18
PCM 22
PC 6
PCM 8
PC 15
PCM 6
PC 8
PCM 18
PC 16
PCM 20
PC 14
PCM 15
PC 9
PCM 17
PC 10
PCM 9
PC 12
PCM 14
as.dist(−abs(cor(Z)))
before re−labeling
Cluster Dendrogram
Height
0.0
−0.2
−0.4
−0.6
−0.8
−1.0
PC 4
PCM 4
PC 3
PCM 3
PC 2
PCM 5
PC 1
PC 5
PCM 1
PCM 2
PC 19
PCM 11
PC 17
PCM 10
PC 23
PCM 19
PC 21
PCM 12
PC 20
PCM 21
PC 7
PCM 7
PC 22
PCM 16
PC 11
PCM 23
PC 13
PCM 13
PC 18
PCM 22
PC 6
PCM 8
PC 15
PCM 6
PC 8
PCM 18
PC 16
PCM 20
PC 14
PCM 15
PC 9
PCM 17
PC 10
PCM 9
PC 12
PCM 14
as.dist(−abs(cor(Z)))
after re−labeling

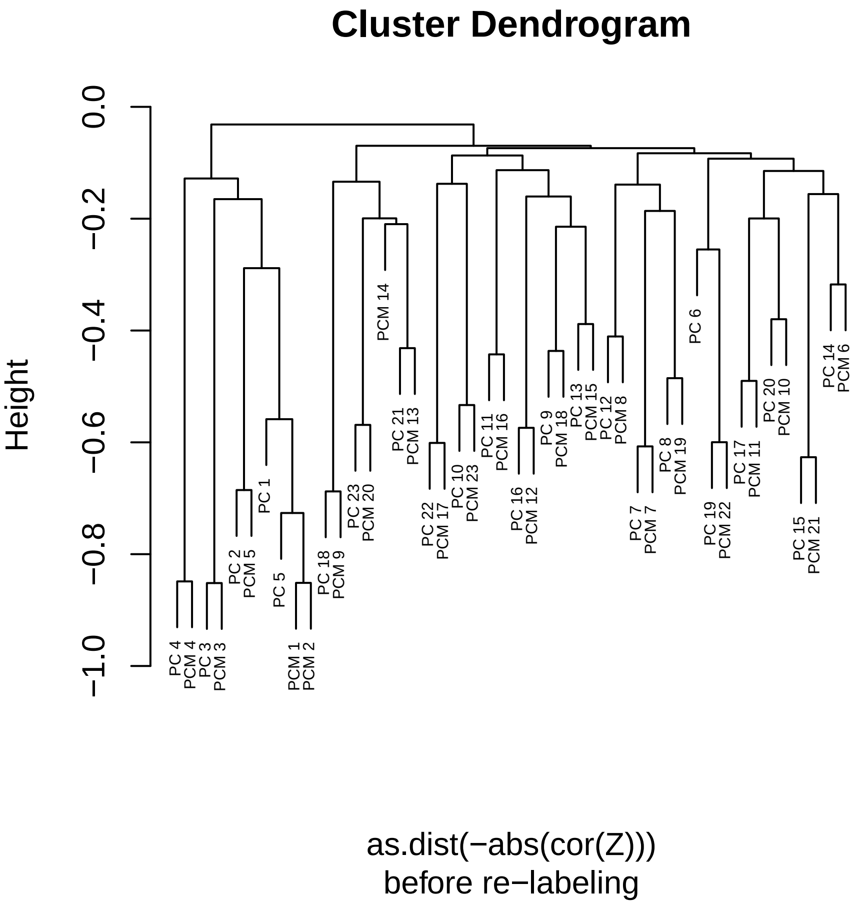
Cluster Dendrogram
Height
0.0
−0.2
−0.4
−0.6
−0.8
−1.0
PC 4
PCM 4
PC 3
PCM 3
PC 2
PCM 5
PC 1
PC 5
PCM 1
PCM 2
PC 18
PCM 9
PC 23
PCM 20
PCM 14
PC 21
PCM 13
PC 22
PCM 17
PC 10
PCM 23
PC 11
PCM 16
PC 16
PCM 12
PC 9
PCM 18
PC 13
PCM 15
PC 12
PCM 8
PC 7
PCM 7
PC 8
PCM 19
PC 6
PC 19
PCM 22
PC 17
PCM 11
PC 20
PCM 10
PC 15
PCM 21
PC 14
PCM 6
as.dist(−abs(cor(Z)))
before re−labeling

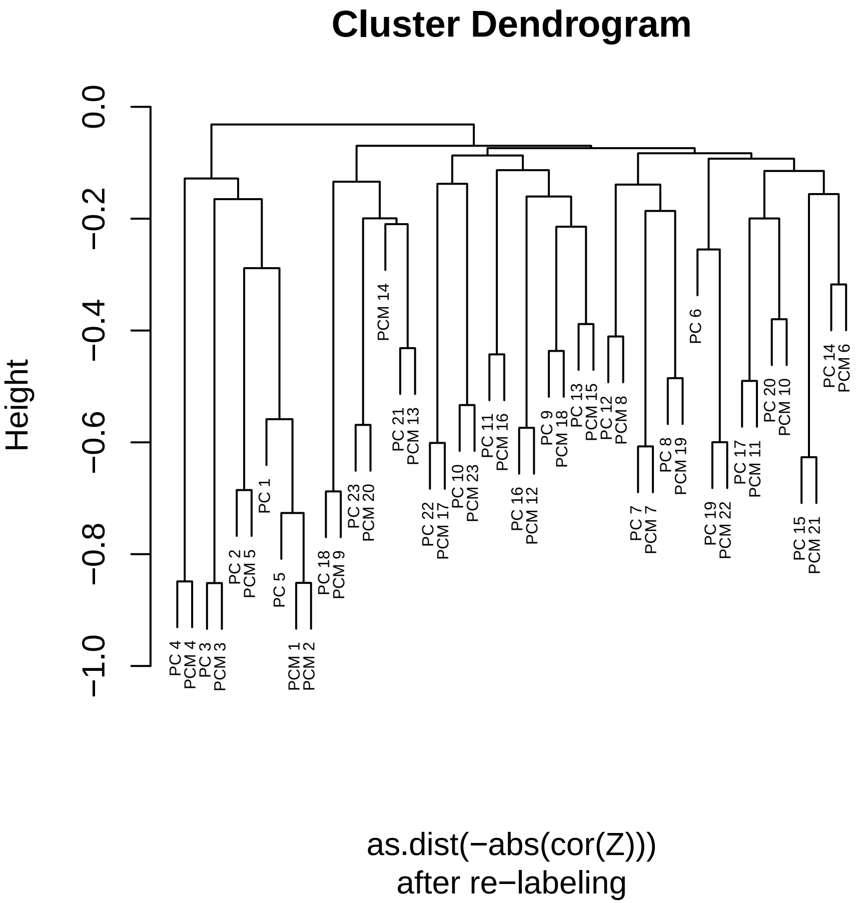
Cluster Dendrogram
Height
0.0
−0.2
−0.4
−0.6
−0.8
−1.0
PC 4
PCM 4
PC 3
PCM 3
PC 2
PCM 5
PC 1
PC 5
PCM 1
PCM 2
PC 18
PCM 9
PC 23
PCM 20
PCM 14
PC 21
PCM 13
PC 22
PCM 17
PC 10
PCM 23
PC 11
PCM 16
PC 16
PCM 12
PC 9
PCM 18
PC 13
PCM 15
PC 12
PCM 8
PC 7
PCM 7
PC 8
PCM 19
PC 6
PC 19
PCM 22
PC 17
PCM 11
PC 20
PCM 10
PC 15
PCM 21
PC 14
PCM 6
as.dist(−abs(cor(Z)))
after re−labeling

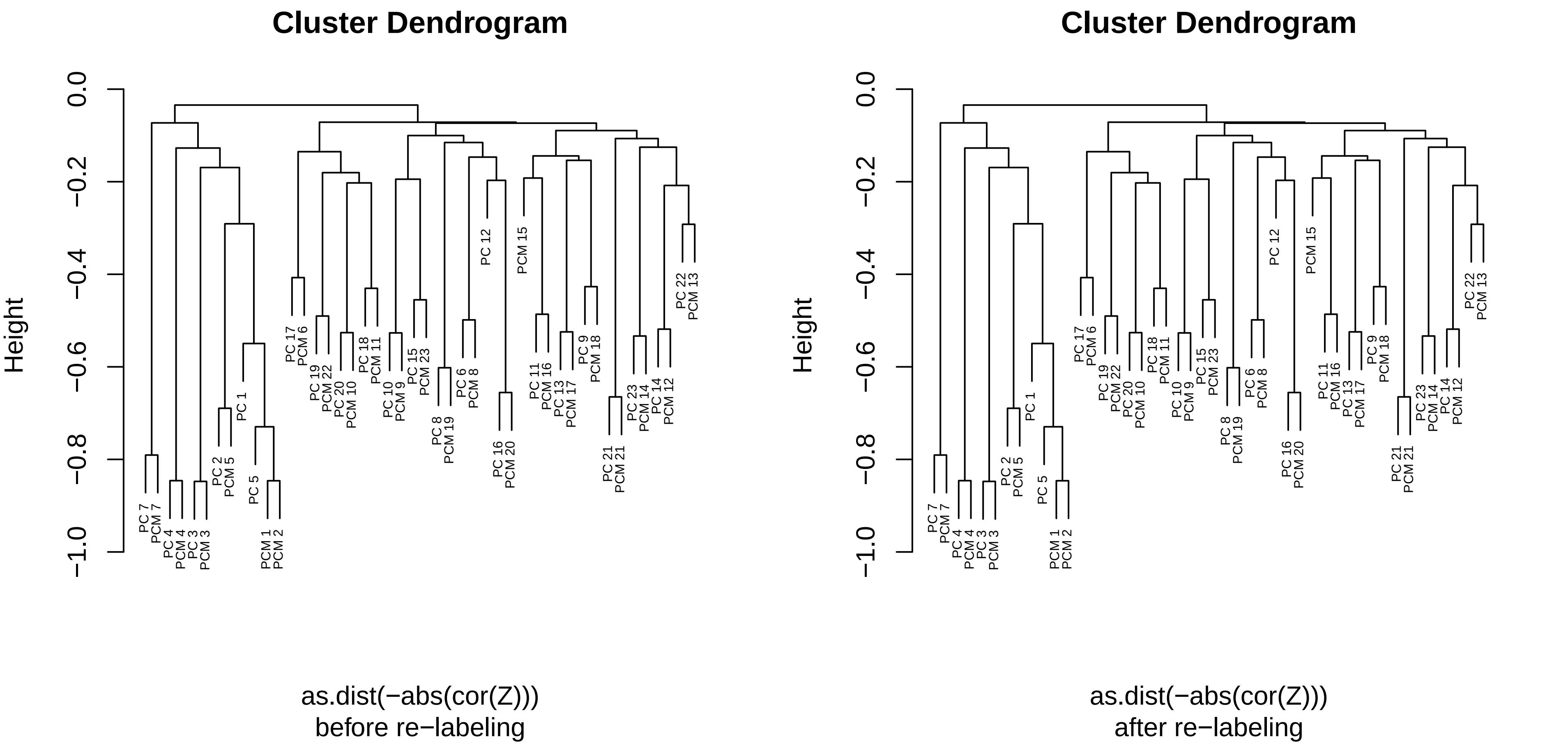
Cluster Dendrogram
Height
0.0
−0.2
−0.4
−0.6
−0.8
−1.0
PC 7
PCM 7
PC 4
PCM 4
PC 3
PCM 3
PC 2
PCM 5
PC 1
PC 5
PCM 1
PCM 2
PC 17
PCM 6
PC 19
PCM 22
PC 20
PCM 10
PC 18
PCM 11
PC 10
PCM 9
PC 15
PCM 23
PC 8
PCM 19
PC 6
PCM 8
PC 12
PC 16
PCM 20
PCM 15
PC 11
PCM 16
PC 13
PCM 17
PC 9
PCM 18
PC 21
PCM 21
PC 23
PCM 14
PC 14
PCM 12
PC 22
PCM 13
as.dist(−abs(cor(Z)))
before re−labeling
Cluster Dendrogram
Height
0.0
−0.2
−0.4
−0.6
−0.8
−1.0
PC 7
PCM 7
PC 4
PCM 4
PC 3
PCM 3
PC 2
PCM 5
PC 1
PC 5
PCM 1
PCM 2
PC 17
PCM 6
PC 19
PCM 22
PC 20
PCM 10
PC 18
PCM 11
PC 10
PCM 9
PC 15
PCM 23
PC 8
PCM 19
PC 6
PCM 8
PC 12
PC 16
PCM 20
PCM 15
PC 11
PCM 16
PC 13
PCM 17
PC 9
PCM 18
PC 21
PCM 21
PC 23
PCM 14
PC 14
PCM 12
PC 22
PCM 13
as.dist(−abs(cor(Z)))
after re−labeling

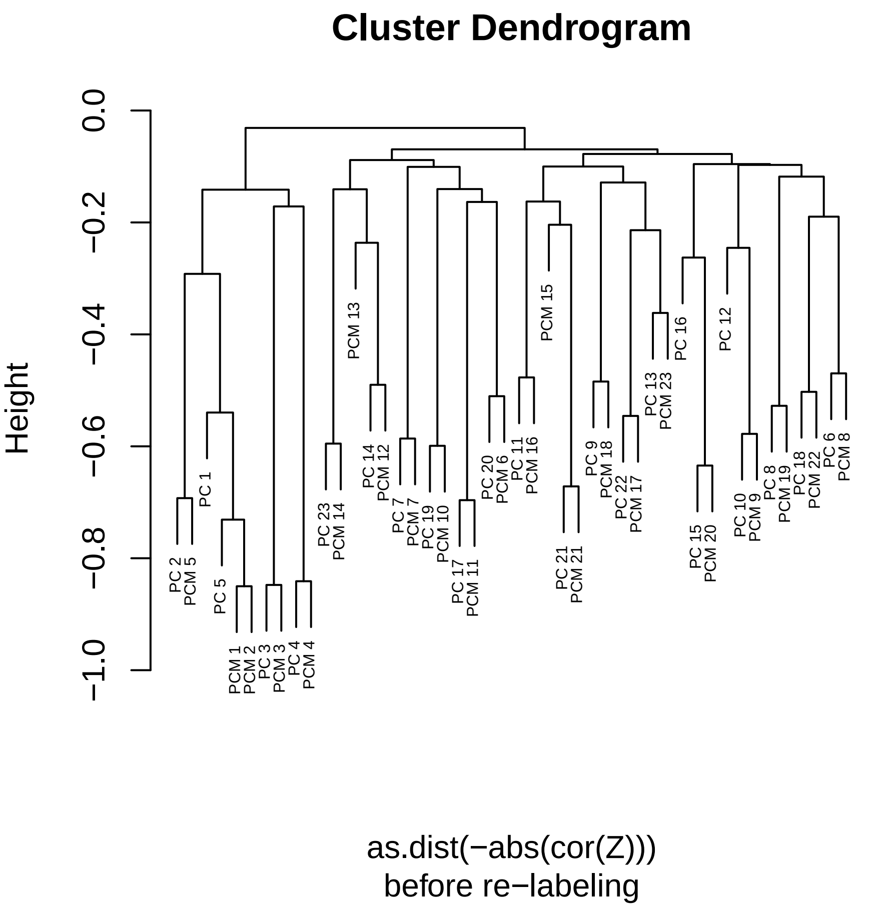
Cluster Dendrogram
Height
0.0
−0.2
−0.4
−0.6
−0.8
−1.0
PC 2
PCM 5
PC 1
PC 5
PCM 1
PCM 2
PC 3
PCM 3
PC 4
PCM 4
PC 23
PCM 14
PCM 13
PC 14
PCM 12
PC 7
PCM 7
PC 19
PCM 10
PC 17
PCM 11
PC 20
PCM 6
PC 11
PCM 16
PCM 15
PC 21
PCM 21
PC 9
PCM 18
PC 22
PCM 17
PC 13
PCM 23
PC 16
PC 15
PCM 20
PC 12
PC 10
PCM 9
PC 8
PCM 19
PC 18
PCM 22
PC 6
PCM 8
as.dist(−abs(cor(Z)))
before re−labeling

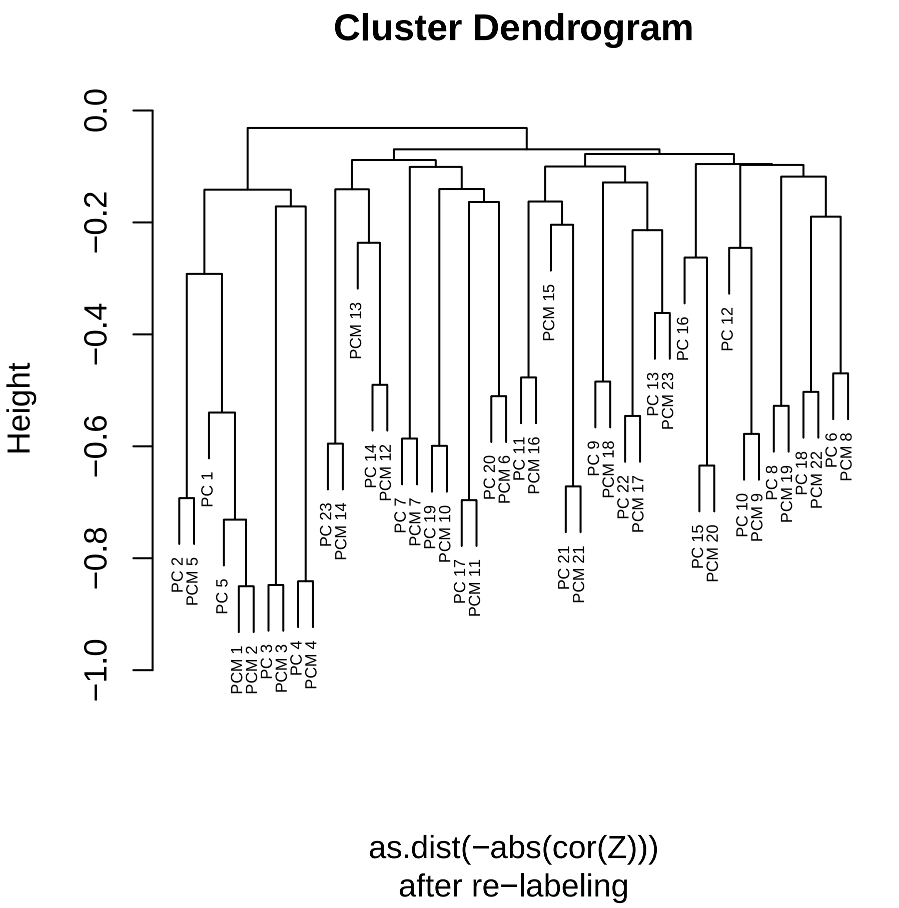
Cluster Dendrogram
Height
0.0
−0.2
−0.4
−0.6
−0.8
−1.0
PC 2
PCM 5
PC 1
PC 5
PCM 1
PCM 2
PC 3
PCM 3
PC 4
PCM 4
PC 23
PCM 14
PCM 13
PC 14
PCM 12
PC 7
PCM 7
PC 19
PCM 10
PC 17
PCM 11
PC 20
PCM 6
PC 11
PCM 16
PCM 15
PC 21
PCM 21
PC 9
PCM 18
PC 22
PCM 17
PC 13
PCM 23
PC 16
PC 15
PCM 20
PC 12
PC 10
PCM 9
PC 8
PCM 19
PC 18
PCM 22
PC 6
PCM 8
as.dist(−abs(cor(Z)))
after re−labeling

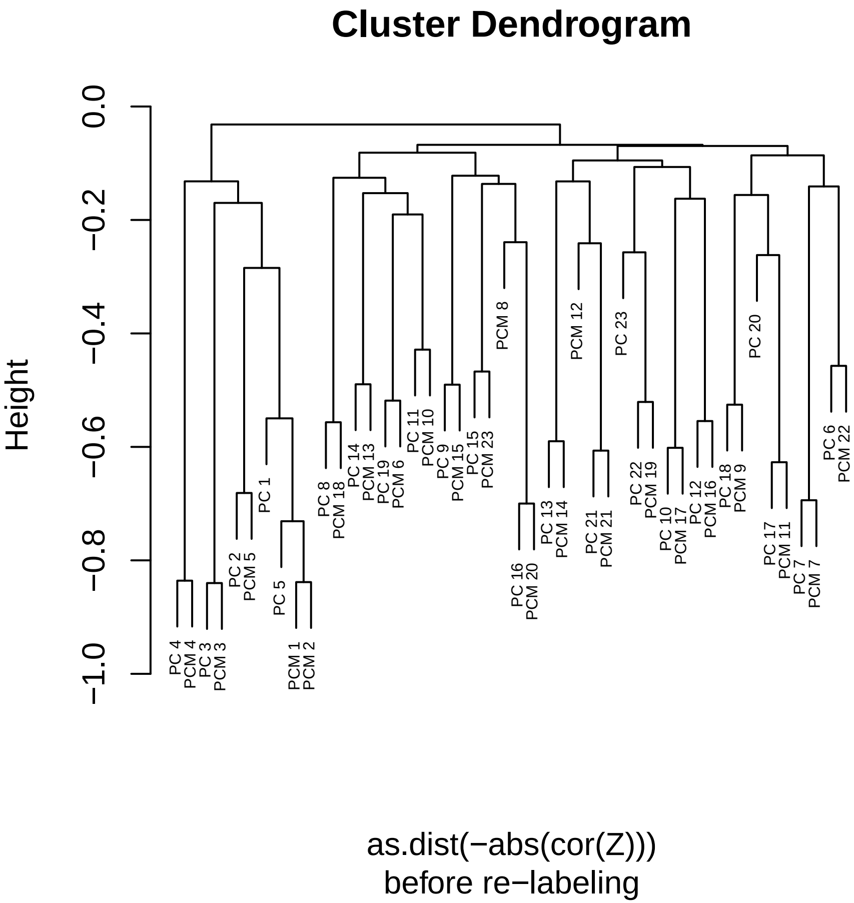
Cluster Dendrogram
Height
0.0
−0.2
−0.4
−0.6
−0.8
−1.0
PC 4
PCM 4
PC 3
PCM 3
PC 2
PCM 5
PC 1
PC 5
PCM 1
PCM 2
PC 8
PCM 18
PC 14
PCM 13
PC 19
PCM 6
PC 11
PCM 10
PC 9
PCM 15
PC 15
PCM 23
PCM 8
PC 16
PCM 20
PC 13
PCM 14
PCM 12
PC 21
PCM 21
PC 23
PC 22
PCM 19
PC 10
PCM 17
PC 12
PCM 16
PC 18
PCM 9
PC 20
PC 17
PCM 11
PC 7
PCM 7
PC 6
PCM 22
as.dist(−abs(cor(Z)))
before re−labeling

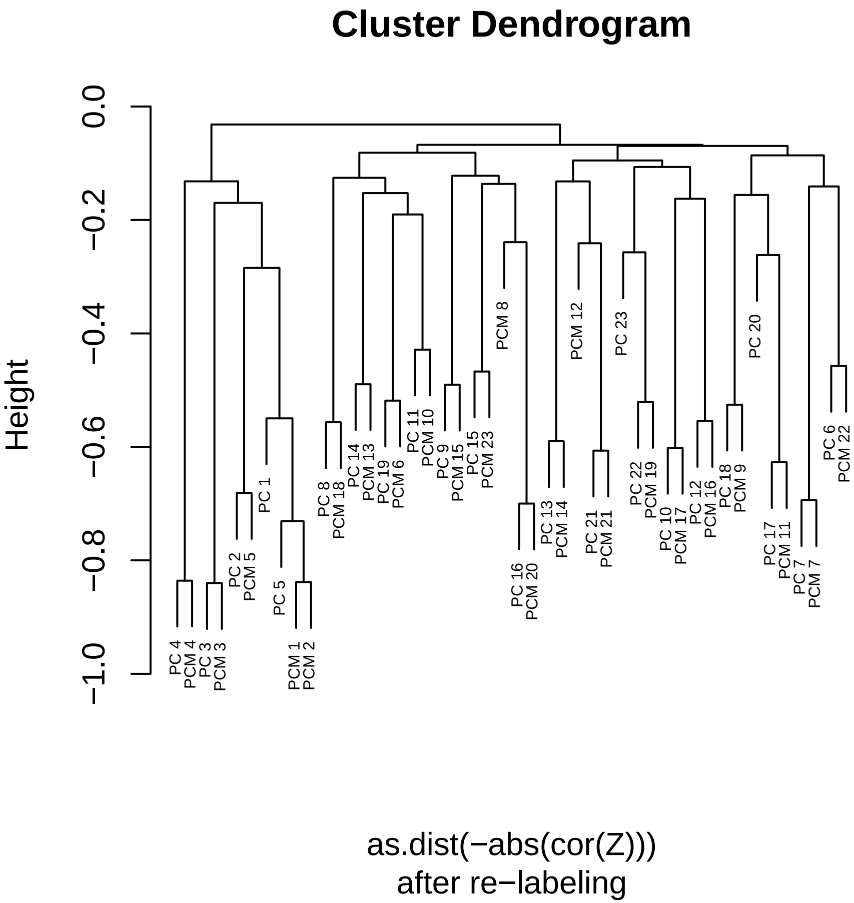
Cluster Dendrogram
Height
0.0
−0.2
−0.4
−0.6
−0.8
−1.0
PC 4
PCM 4
PC 3
PCM 3
PC 2
PCM 5
PC 1
PC 5
PCM 1
PCM 2
PC 8
PCM 18
PC 14
PCM 13
PC 19
PCM 6
PC 11
PCM 10
PC 9
PCM 15
PC 15
PCM 23
PCM 8
PC 16
PCM 20
PC 13
PCM 14
PCM 12
PC 21
PCM 21
PC 23
PC 22
PCM 19
PC 10
PCM 17
PC 12
PCM 16
PC 18
PCM 9
PC 20
PC 17
PCM 11
PC 7
PCM 7
PC 6
PCM 22
as.dist(−abs(cor(Z)))
after re−labeling

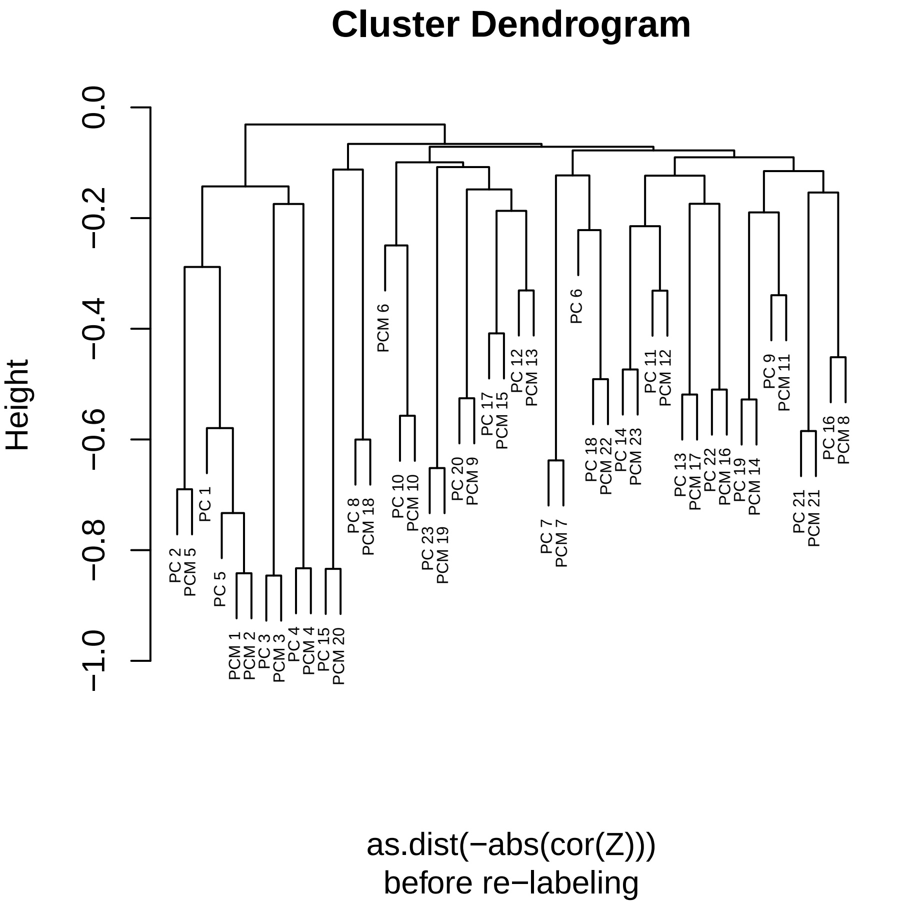
Cluster Dendrogram
Height
0.0
−0.2
−0.4
−0.6
−0.8
−1.0
PC 2
PCM 5
PC 1
PC 5
PCM 1
PCM 2
PC 3
PCM 3
PC 4
PCM 4
PC 15
PCM 20
PC 8
PCM 18
PCM 6
PC 10
PCM 10
PC 23
PCM 19
PC 20
PCM 9
PC 17
PCM 15
PC 12
PCM 13
PC 6
PC 7
PCM 7
PC 18
PCM 22
PC 14
PCM 23
PC 11
PCM 12
PC 13
PCM 17
PC 22
PCM 16
PC 19
PCM 14
PC 9
PCM 11
PC 21
PCM 21
PC 16
PCM 8
as.dist(−abs(cor(Z)))
before re−labeling

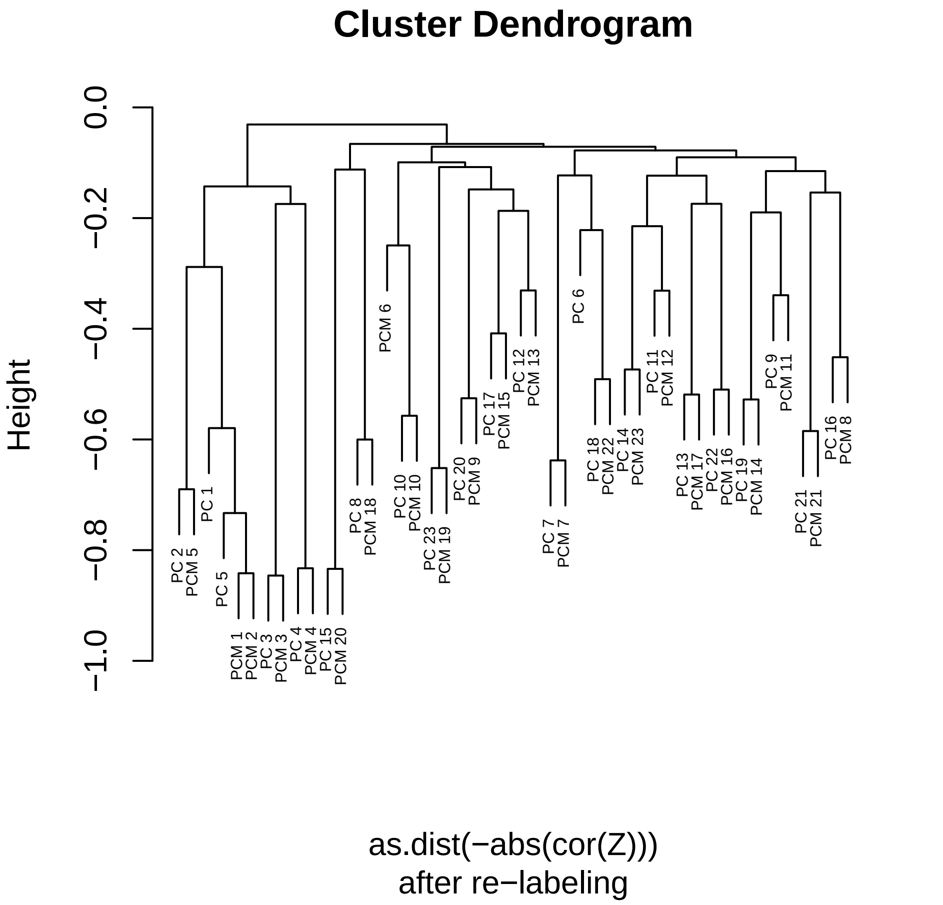
Cluster Dendrogram
Height
0.0
−0.2
−0.4
−0.6
−0.8
−1.0
PC 2
PCM 5
PC 1
PC 5
PCM 1
PCM 2
PC 3
PCM 3
PC 4
PCM 4
PC 15
PCM 20
PC 8
PCM 18
PCM 6
PC 10
PCM 10
PC 23
PCM 19
PC 20
PCM 9
PC 17
PCM 15
PC 12
PCM 13
PC 6
PC 7
PCM 7
PC 18
PCM 22
PC 14
PCM 23
PC 11
PCM 12
PC 13
PCM 17
PC 22
PCM 16
PC 19
PCM 14
PC 9
PCM 11
PC 21
PCM 21
PC 16
PCM 8
as.dist(−abs(cor(Z)))
after re−labeling

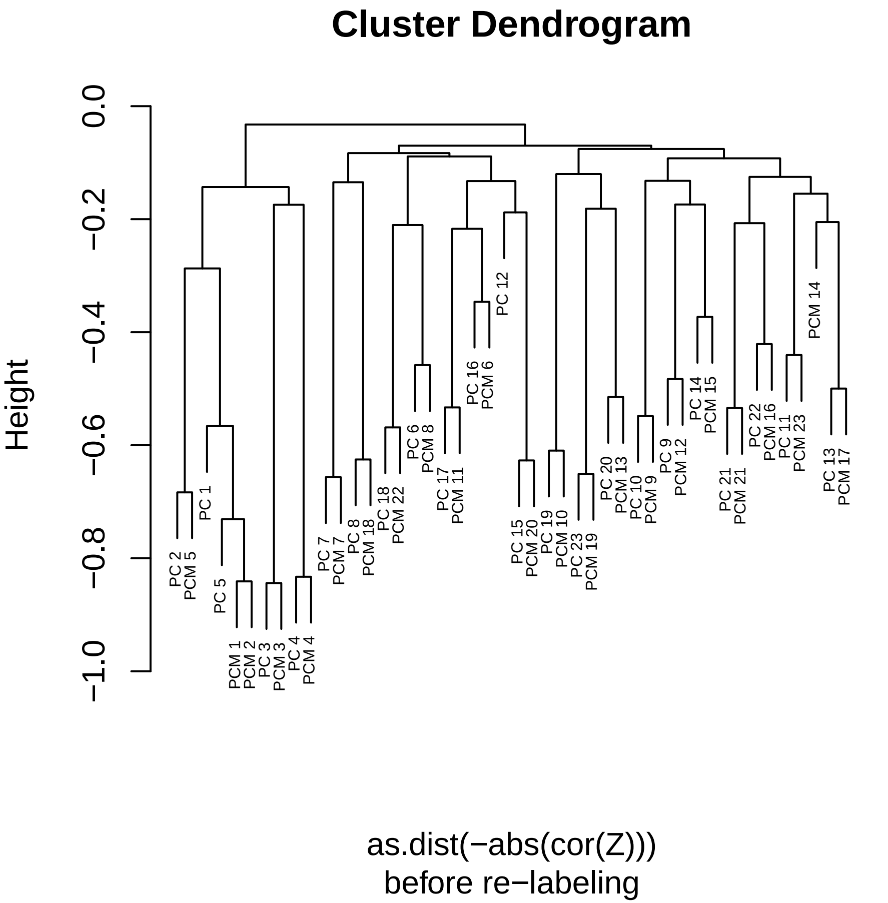

Cluster Dendrogram
Height
0.0
−0.2
−0.4
−0.6
−0.8
−1.0
PC 2
PCM 5
PC 1
PC 5
PCM 1
PCM 2
PC 3
PCM 3
PC 4
PCM 4
PC 7
PCM 7
PC 8
PCM 18
PC 18
PCM 22
PC 6
PCM 8
PC 17
PCM 11
PC 16
PCM 6
PC 12
PC 15
PCM 20
PC 19
PCM 10
PC 23
PCM 19
PC 20
PCM 13
PC 10
PCM 9
PC 9
PCM 12
PC 14
PCM 15
PC 21
PCM 21
PC 22
PCM 16
PC 11
PCM 23
PCM 14
PC 13
PCM 17
as.dist(−abs(cor(Z)))
before re−labeling

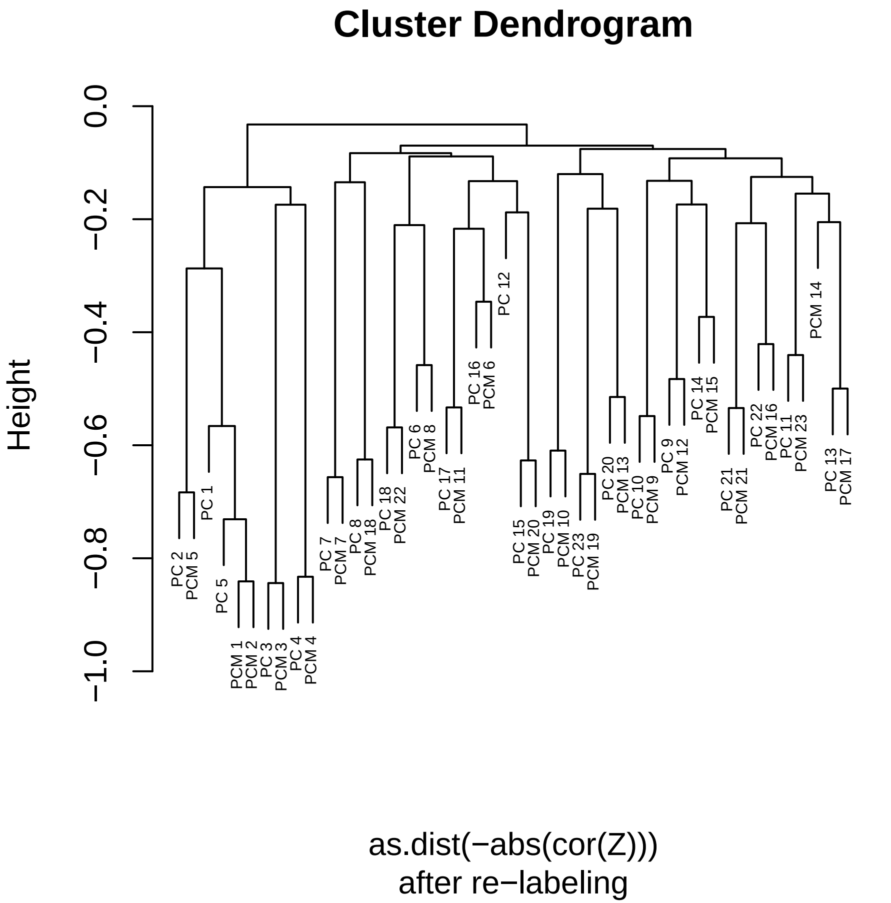

Cluster Dendrogram
Height
0.0
−0.2
−0.4
−0.6
−0.8
−1.0
PC 2
PCM 5
PC 1
PC 5
PCM 1
PCM 2
PC 3
PCM 3
PC 4
PCM 4
PC 7
PCM 7
PC 8
PCM 18
PC 18
PCM 22
PC 6
PCM 8
PC 17
PCM 11
PC 16
PCM 6
PC 12
PC 15
PCM 20
PC 19
PCM 10
PC 23
PCM 19
PC 20
PCM 13
PC 10
PCM 9
PC 9
PCM 12
PC 14
PCM 15
PC 21
PCM 21
PC 22
PCM 16
PC 11
PCM 23
PCM 14
PC 13
PCM 17
as.dist(−abs(cor(Z)))
after re−labeling

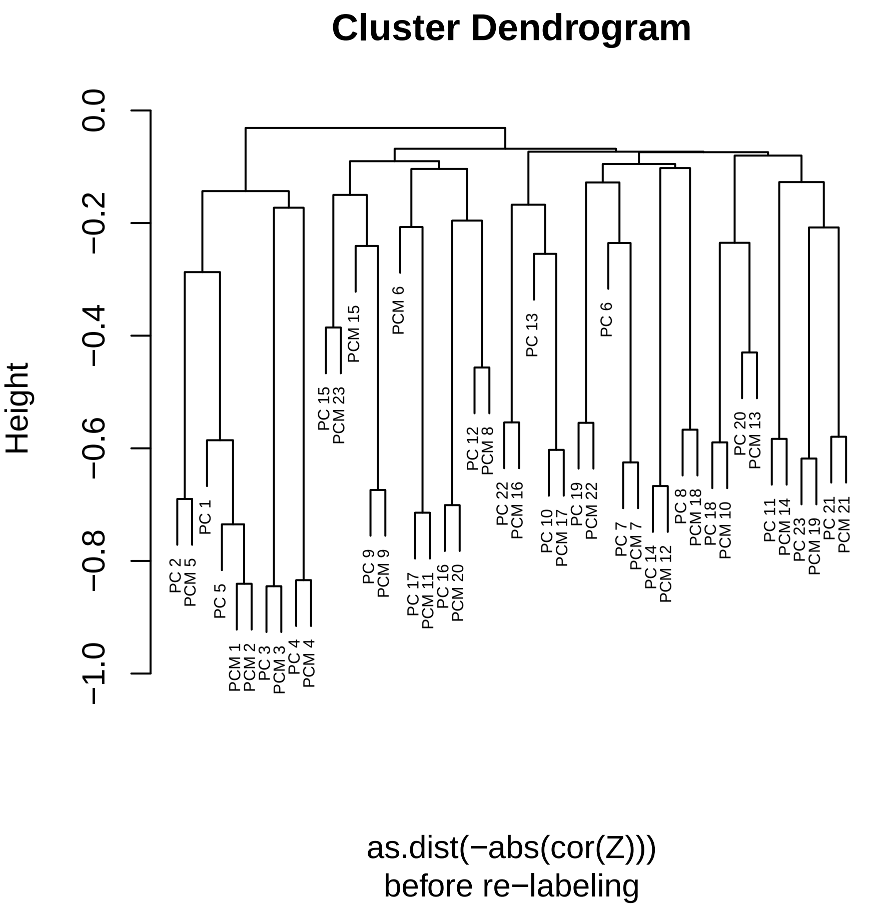

Cluster Dendrogram
Height
0.0
−0.2
−0.4
−0.6
−0.8
−1.0
PC 2
PCM 5
PC 1
PC 5
PCM 1
PCM 2
PC 3
PCM 3
PC 4
PCM 4
PC 15
PCM 23
PCM 15
PC 9
PCM 9
PCM 6
PC 17
PCM 11
PC 16
PCM 20
PC 12
PCM 8
PC 22
PCM 16
PC 13
PC 10
PCM 17
PC 19
PCM 22
PC 6
PC 7
PCM 7
PC 14
PCM 12
PC 8
PCM 18
PC 18
PCM 10
PC 20
PCM 13
PC 11
PCM 14
PC 23
PCM 19
PC 21
PCM 21
as.dist(−abs(cor(Z)))
before re−labeling

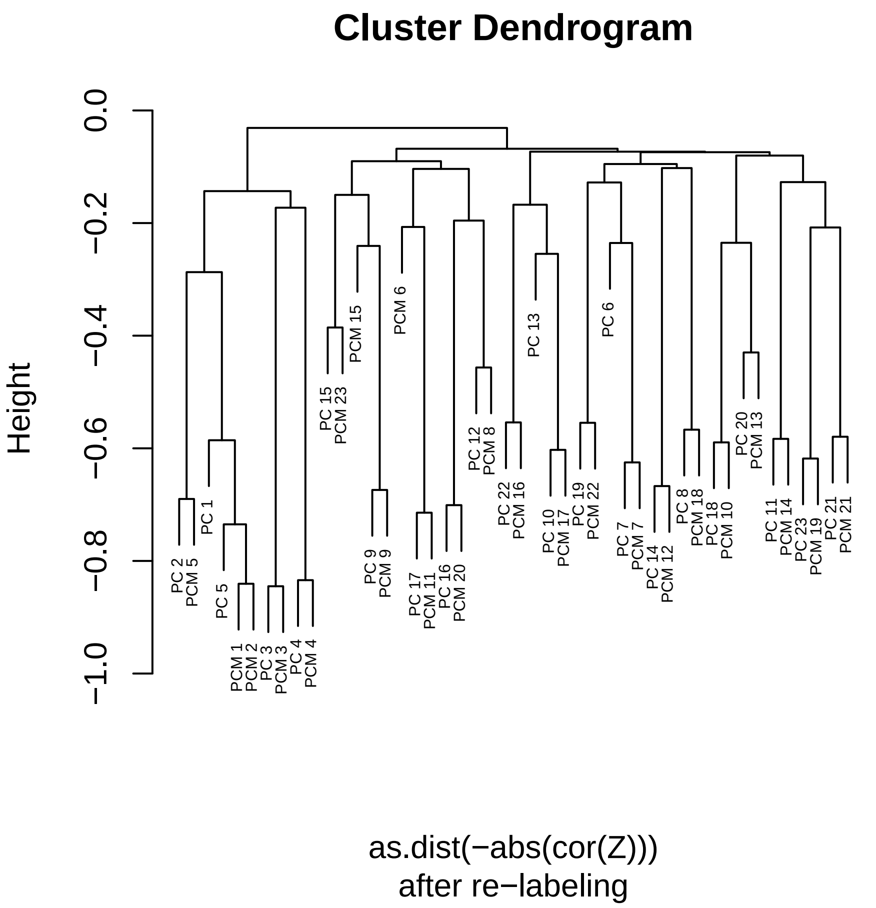

Cluster Dendrogram
Height
0.0
−0.2
−0.4
−0.6
−0.8
−1.0
PC 2
PCM 5
PC 1
PC 5
PCM 1
PCM 2
PC 3
PCM 3
PC 4
PCM 4
PC 15
PCM 23
PCM 15
PC 9
PCM 9
PCM 6
PC 17
PCM 11
PC 16
PCM 20
PC 12
PCM 8
PC 22
PCM 16
PC 13
PC 10
PCM 17
PC 19
PCM 22
PC 6
PC 7
PCM 7
PC 14
PCM 12
PC 8
PCM 18
PC 18
PCM 10
PC 20
PCM 13
PC 11
PCM 14
PC 23
PCM 19
PC 21
PCM 21
as.dist(−abs(cor(Z)))
after re−labeling

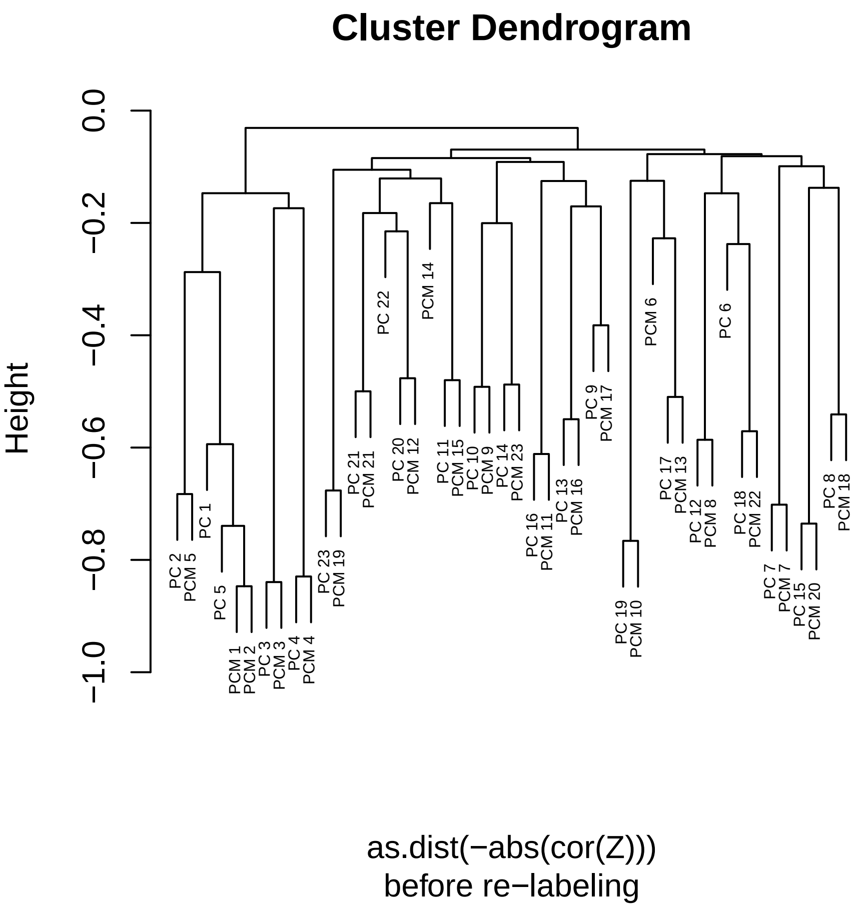

Cluster Dendrogram
Height
0.0
−0.2
−0.4
−0.6
−0.8
−1.0
PC 2
PCM 5
PC 1
PC 5
PCM 1
PCM 2
PC 3
PCM 3
PC 4
PCM 4
PC 23
PCM 19
PC 21
PCM 21
PC 22
PC 20
PCM 12
PCM 14
PC 11
PCM 15
PC 10
PCM 9
PC 14
PCM 23
PC 16
PCM 11
PC 13
PCM 16
PC 9
PCM 17
PC 19
PCM 10
PCM 6
PC 17
PCM 13
PC 12
PCM 8
PC 6
PC 18
PCM 22
PC 7
PCM 7
PC 15
PCM 20
PC 8
PCM 18
as.dist(−abs(cor(Z)))
before re−labeling

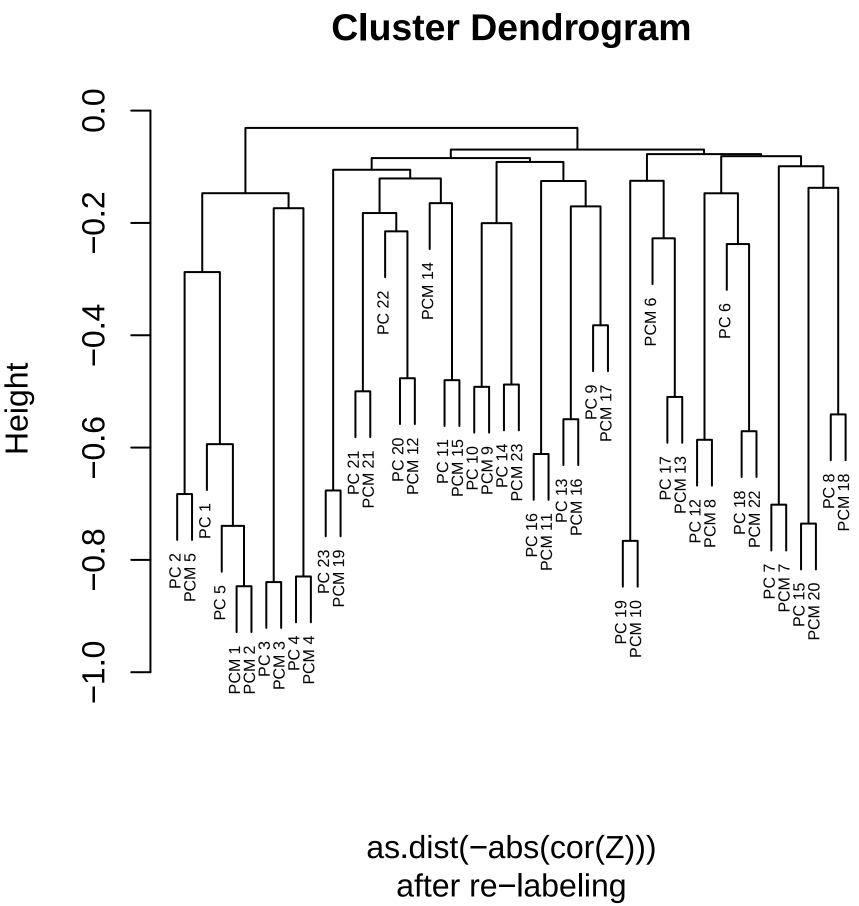

Cluster Dendrogram
Height
0.0
−0.2
−0.4
−0.6
−0.8
−1.0
PC 2
PCM 5
PC 1
PC 5
PCM 1
PCM 2
PC 3
PCM 3
PC 4
PCM 4
PC 23
PCM 19
PC 21
PCM 21
PC 22
PC 20
PCM 12
PCM 14
PC 11
PCM 15
PC 10
PCM 9
PC 14
PCM 23
PC 16
PCM 11
PC 13
PCM 16
PC 9
PCM 17
PC 19
PCM 10
PCM 6
PC 17
PCM 13
PC 12
PCM 8
PC 6
PC 18
PCM 22
PC 7
PCM 7
PC 15
PCM 20
PC 8
PCM 18
as.dist(−abs(cor(Z)))
after re−labeling

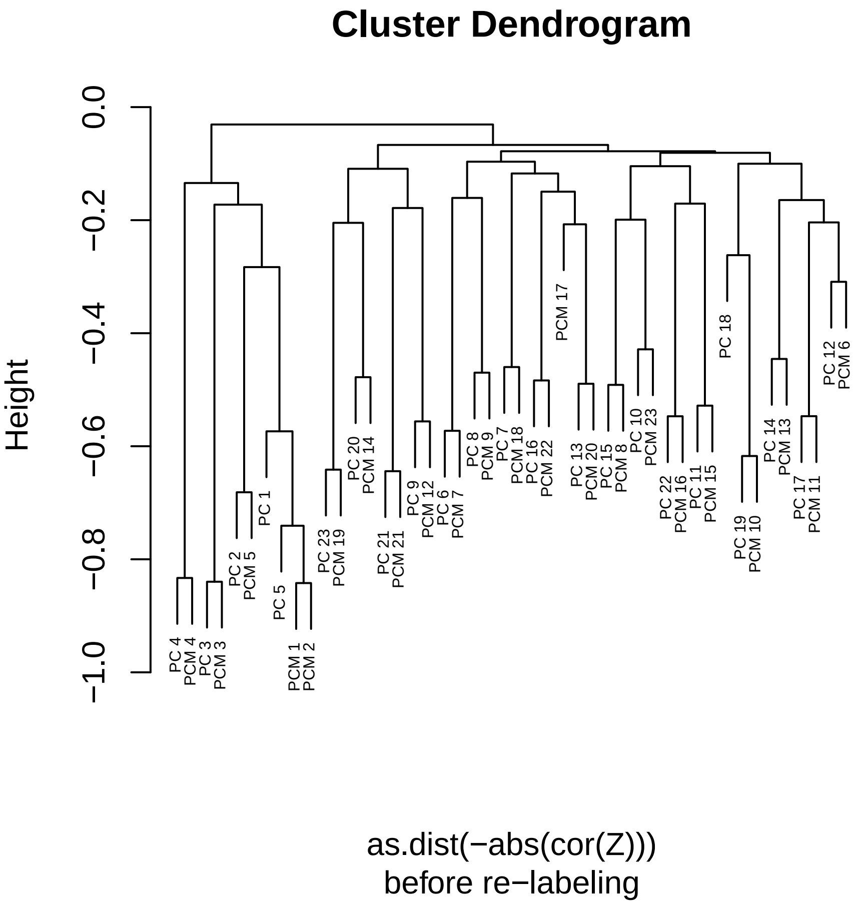
Cluster Dendrogram
Height
0.0
−0.2
−0.4
−0.6
−0.8
−1.0
PC 4
PCM 4
PC 3
PCM 3
PC 2
PCM 5
PC 1
PC 5
PCM 1
PCM 2
PC 23
PCM 19
PC 20
PCM 14
PC 21
PCM 21
PC 9
PCM 12
PC 6
PCM 7
PC 8
PCM 9
PC 7
PCM 18
PC 16
PCM 22
PCM 17
PC 13
PCM 20
PC 15
PCM 8
PC 10
PCM 23
PC 22
PCM 16
PC 11
PCM 15
PC 18
PC 19
PCM 10
PC 14
PCM 13
PC 17
PCM 11
PC 12
PCM 6
as.dist(−abs(cor(Z)))
before re−labeling

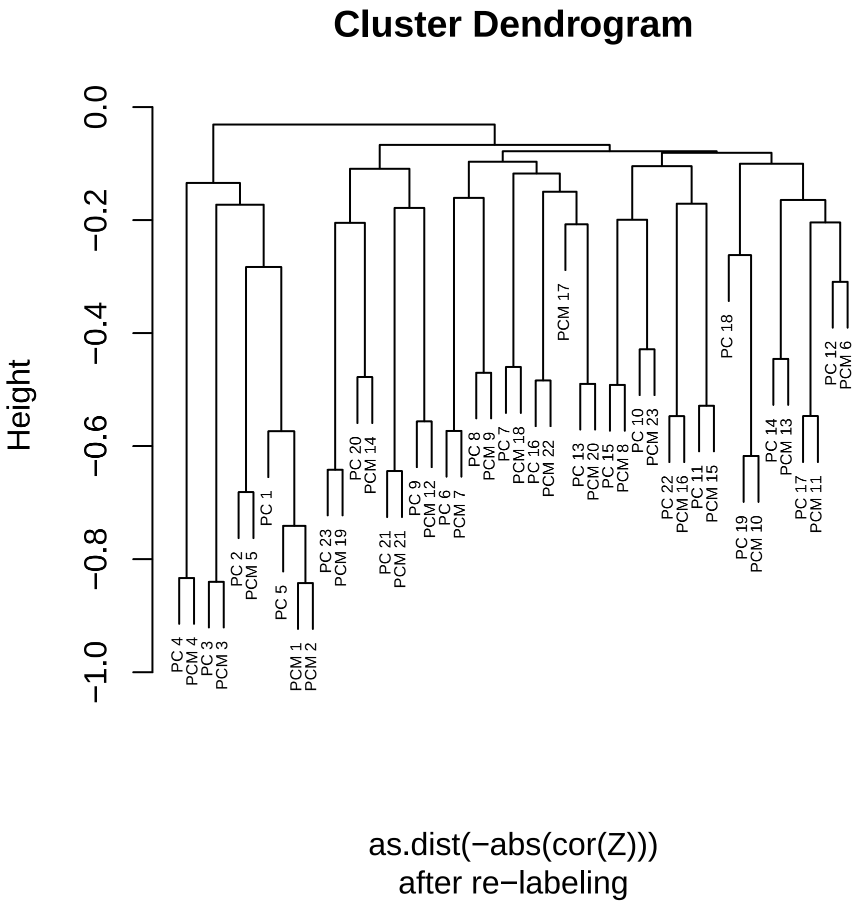
Cluster Dendrogram
Height
0.0
−0.2
−0.4
−0.6
−0.8
−1.0
PC 4
PCM 4
PC 3
PCM 3
PC 2
PCM 5
PC 1
PC 5
PCM 1
PCM 2
PC 23
PCM 19
PC 20
PCM 14
PC 21
PCM 21
PC 9
PCM 12
PC 6
PCM 7
PC 8
PCM 9
PC 7
PCM 18
PC 16
PCM 22
PCM 17
PC 13
PCM 20
PC 15
PCM 8
PC 10
PCM 23
PC 22
PCM 16
PC 11
PCM 15
PC 18
PC 19
PCM 10
PC 14
PCM 13
PC 17
PCM 11
PC 12
PCM 6
as.dist(−abs(cor(Z)))
after re−labeling

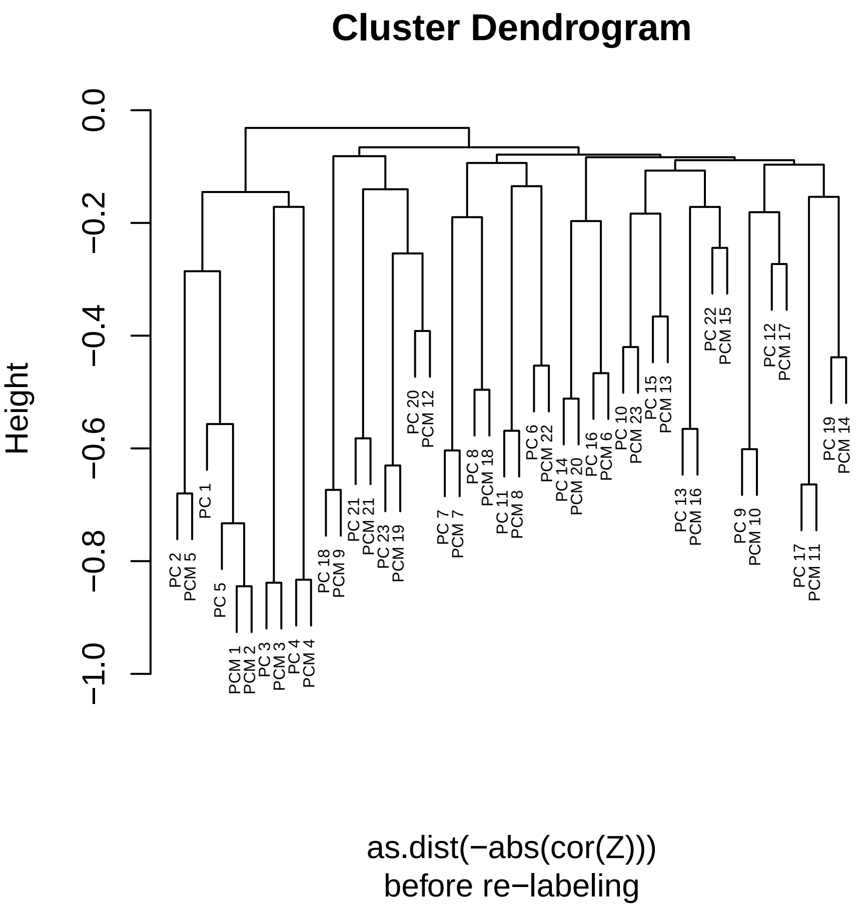
Cluster Dendrogram
Height
0.0
−0.2
−0.4
−0.6
−0.8
−1.0
PC 2
PCM 5
PC 1
PC 5
PCM 1
PCM 2
PC 3
PCM 3
PC 4
PCM 4
PC 18
PCM 9
PC 21
PCM 21
PC 23
PCM 19
PC 20
PCM 12
PC 7
PCM 7
PC 8
PCM 18
PC 11
PCM 8
PC 6
PCM 22
PC 14
PCM 20
PC 16
PCM 6
PC 10
PCM 23
PC 15
PCM 13
PC 13
PCM 16
PC 22
PCM 15
PC 9
PCM 10
PC 12
PCM 17
PC 17
PCM 11
PC 19
PCM 14
as.dist(−abs(cor(Z)))
before re−labeling

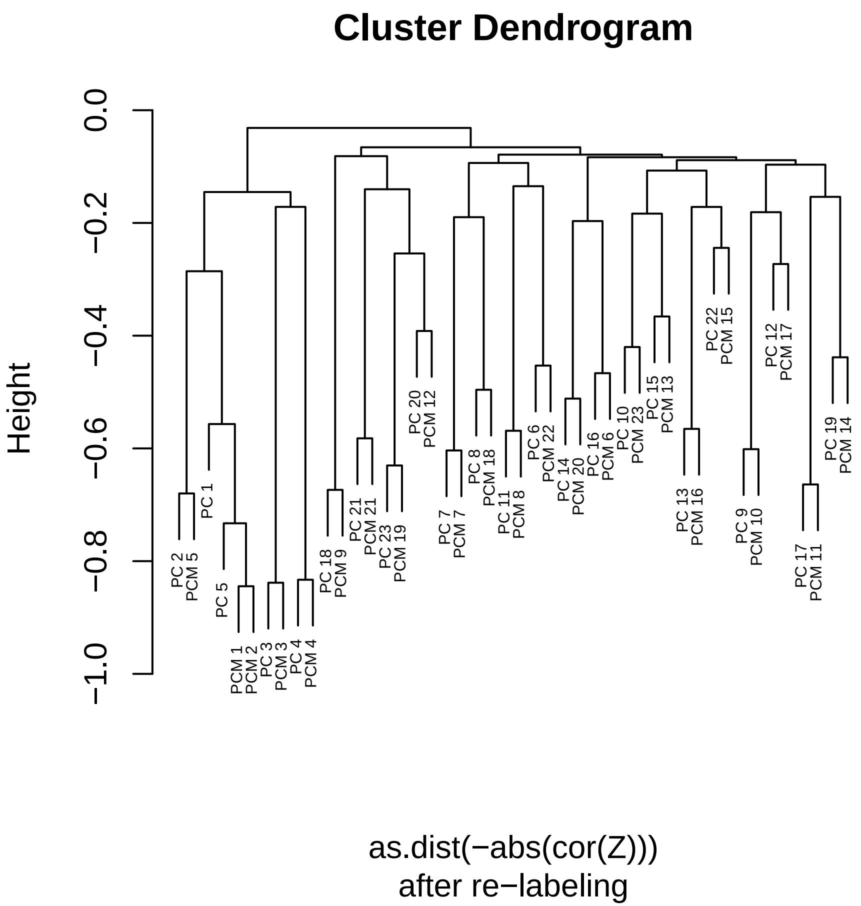
Cluster Dendrogram
Height
0.0
−0.2
−0.4
−0.6
−0.8
−1.0
PC 2
PCM 5
PC 1
PC 5
PCM 1
PCM 2
PC 3
PCM 3
PC 4
PCM 4
PC 18
PCM 9
PC 21
PCM 21
PC 23
PCM 19
PC 20
PCM 12
PC 7
PCM 7
PC 8
PCM 18
PC 11
PCM 8
PC 6
PCM 22
PC 14
PCM 20
PC 16
PCM 6
PC 10
PCM 23
PC 15
PCM 13
PC 13
PCM 16
PC 22
PCM 15
PC 9
PCM 10
PC 12
PCM 17
PC 17
PCM 11
PC 19
PCM 14
as.dist(−abs(cor(Z)))
after re−labeling
