# Supplementary material for: SFRP1 is a possible candidate for epigenetic therapy in non-small cell lung cancer
Source: BMC Med Genomics. 2016 Aug 12;9(Suppl 1):28. doi: 10.1186/s12920-016-0196-3 (PMC4989892; doi:10.1186/s12920-016-0196-3)

NM_020436.2 SALL4
COR= −8.93e−01 P= 2.18e−09

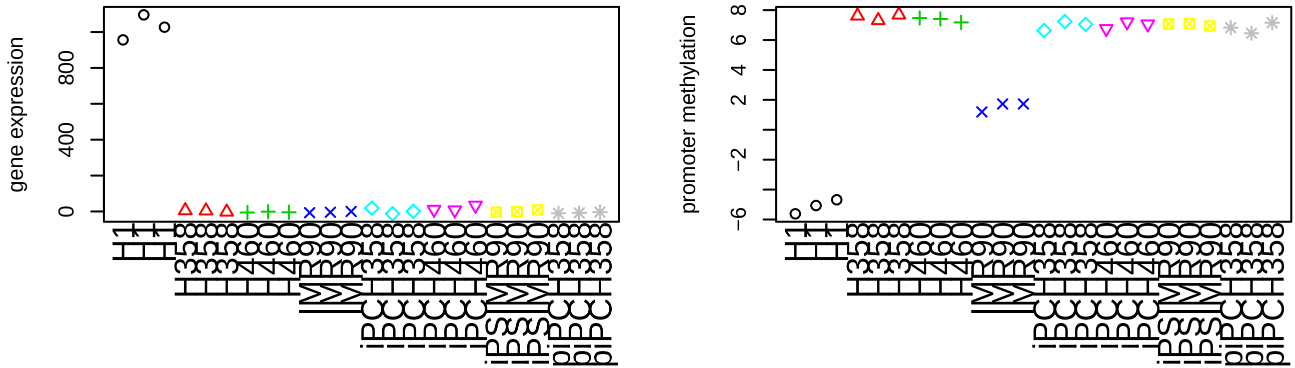

NM_002354.1 TACSTD1
COR= −7.79e−01 P= 3.71e−06

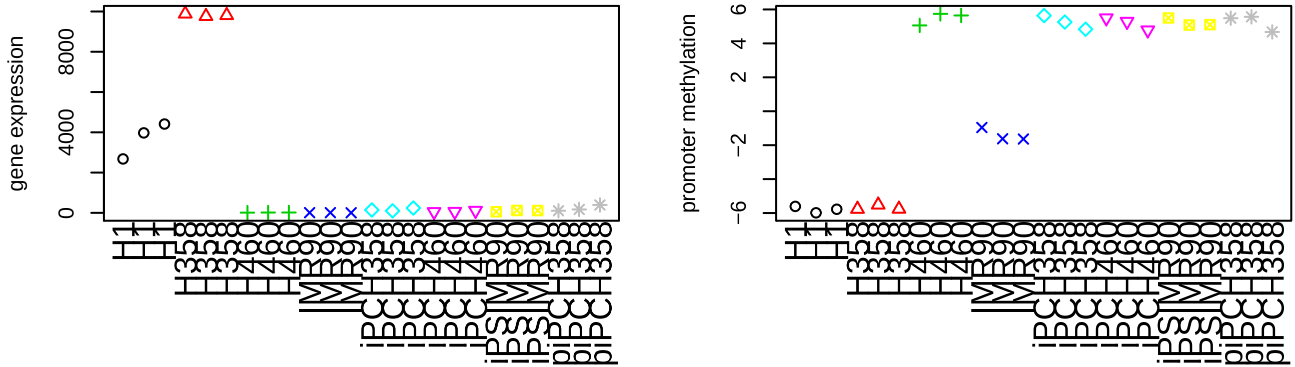

NM_001146.3 ANGPT1
COR= −9.86e−02 P= 3.23e−01

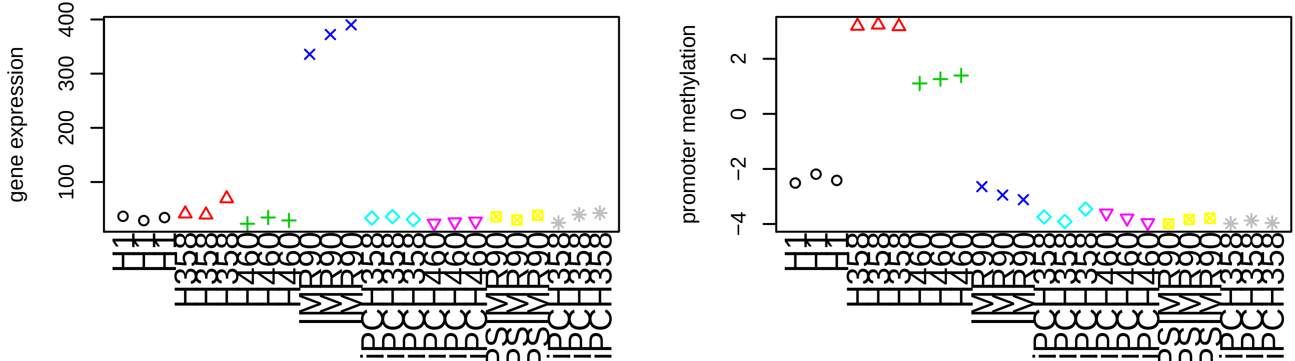

NM_002353.1 TACSTD2
COR= −6.07e−01 P= 8.31e−04

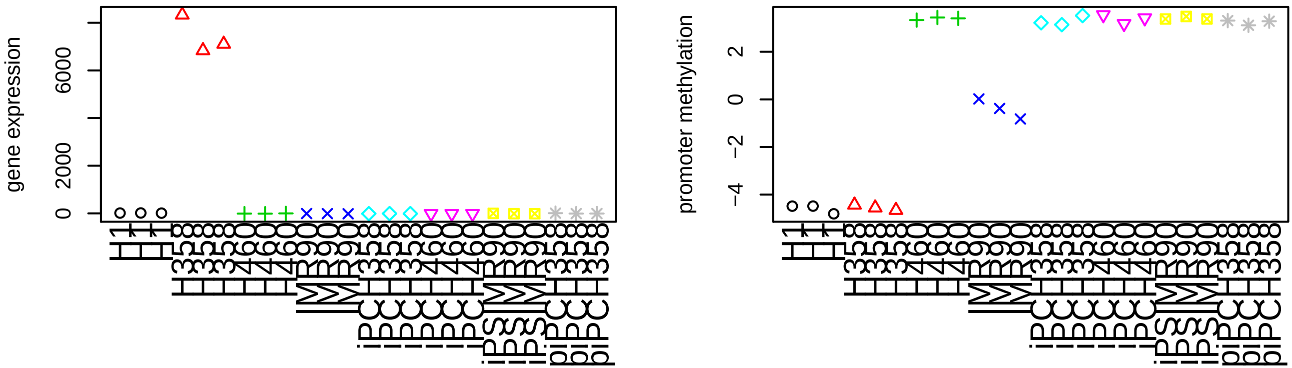

NM_032880.2 IGSF21
COR= −2.97e−01 P= 7.95e−02

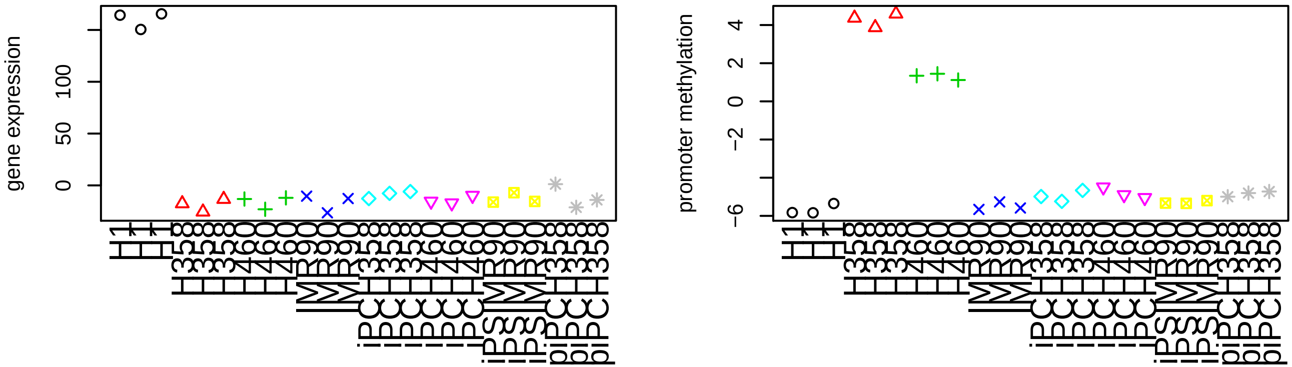

NM_004429.3 EFNB1
COR= 4.59e−01 P= 1.21e−02

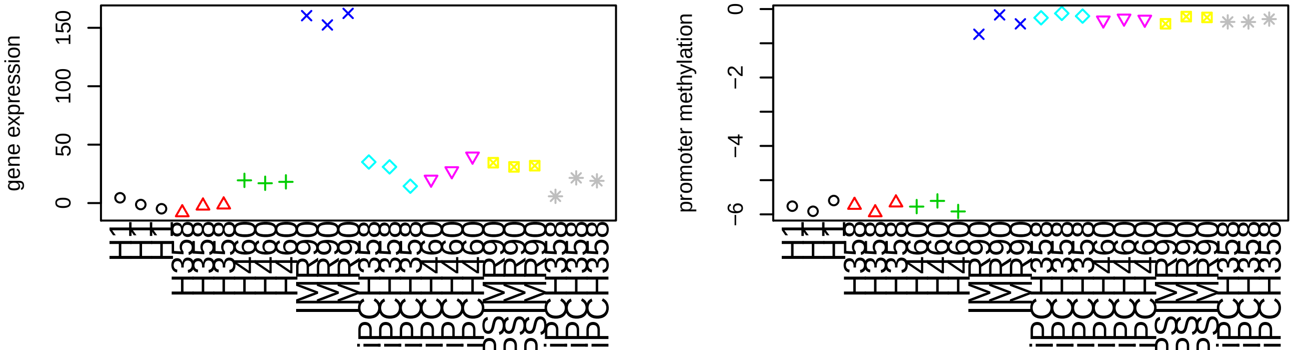

NM_177524.1 MEST
COR= −8.94e−01 P= 1.97e−09

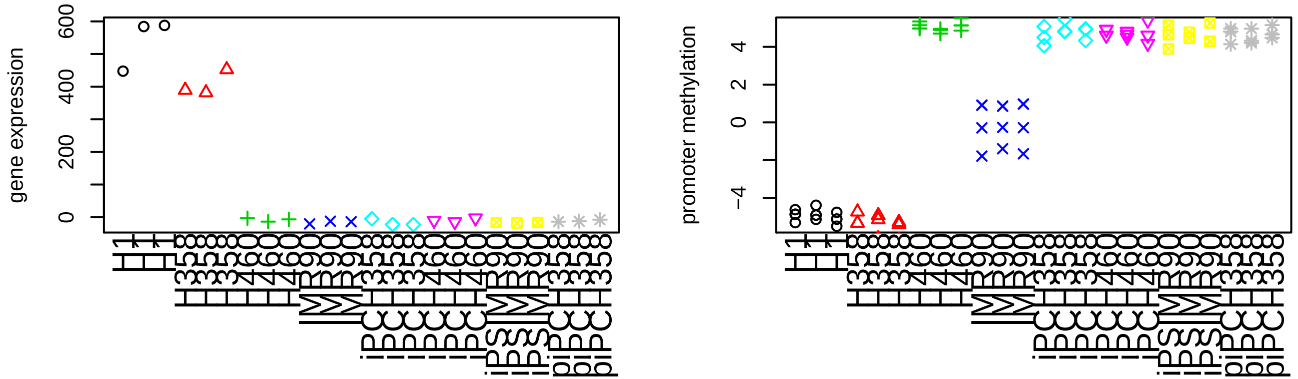

NM_013243.2 SCG3
COR= −1.70e−01 P= 2.14e−01

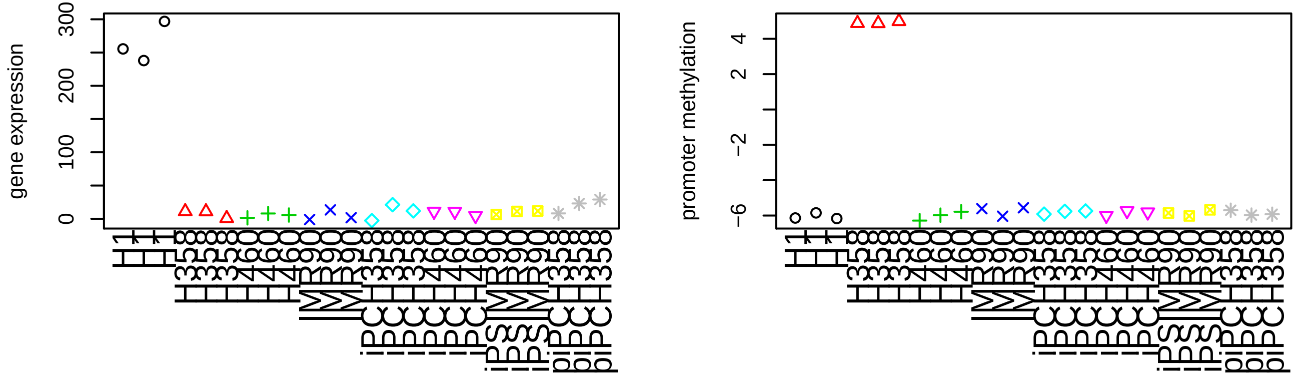

Supplement: Additional file 3: — FE based upon categorical regression. Gene expression/promoter methylation of genes selected by categorical regression. FE based upon categorical regression does not always guarantee a high correlation between gene expression and promoter methylation, because it simply filters those with distinct expression/methylation among samples. However, among eight genes selected, four had a significant (P < 0.05) negative correlation coefficient between gene expression and promoter methylation. This suggested the feasibility of FE based upon categorical regression. The correlation between gene expression and promoter methylation is very high. However, only one gene had a significant positive correlation. (PDF 19 kb) [file 12920_2016_196_MOESM3_ESM.pdf]
