# Supplementary material for: SFRP1 is a possible candidate for epigenetic therapy in non-small cell lung cancer
Source: BMC Med Genomics. 2016 Aug 12;9(Suppl 1):28. doi: 10.1186/s12920-016-0196-3 (PMC4989892; doi:10.1186/s12920-016-0196-3)

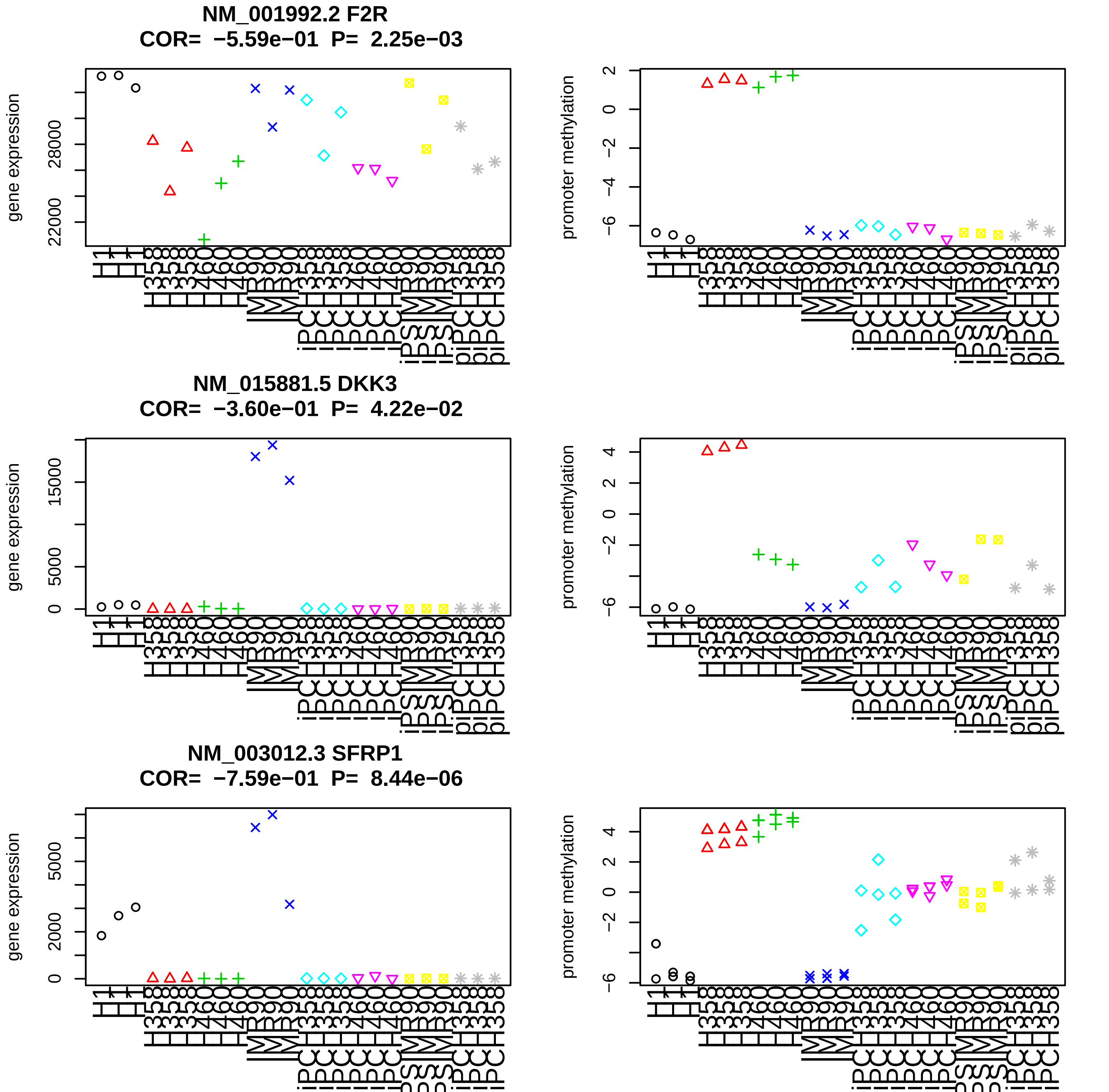
NM_001992.2 F2R
COR= −5.59e−01 P= 2.25e−03
gene expression
promoter methylation
NM_015881.5 DKK3
COR= −3.60e−01 P= 4.22e−02
gene expression
promoter methylation
NM_003012.3 SFRP1
COR= −7.59e−01 P= 8.44e−06
gene expression
promoter methylation

NM_213606.1 SLC16A12
COR= −2.09e−01 P= 1.63e−01

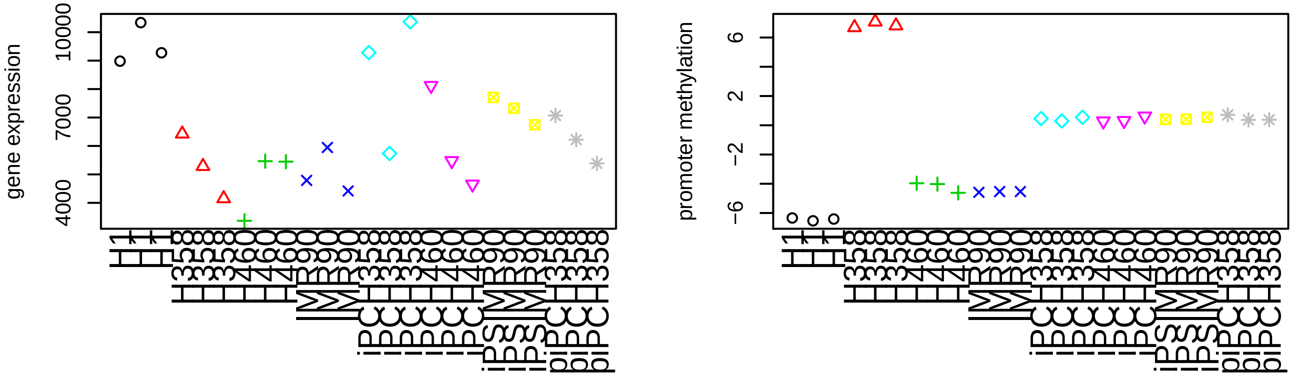

NM_019102.2 HOXA5
COR= −5.36e−01 P= 3.49e−03

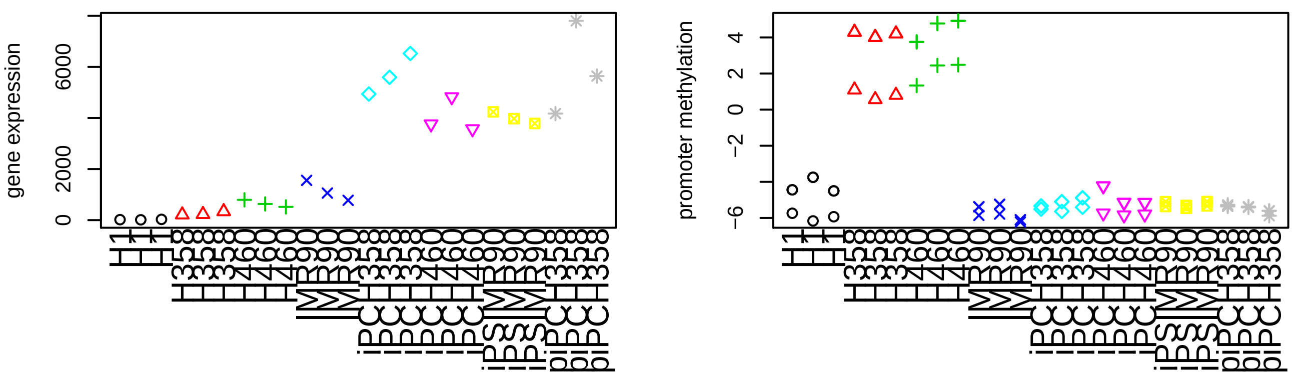

NM_004321.4 KIF1A
COR= −3.44e−01 P= 4.97e−02

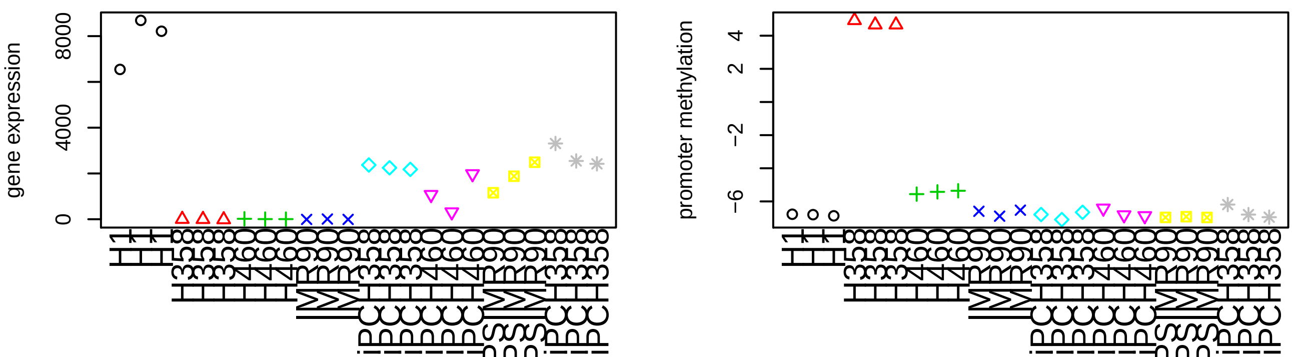

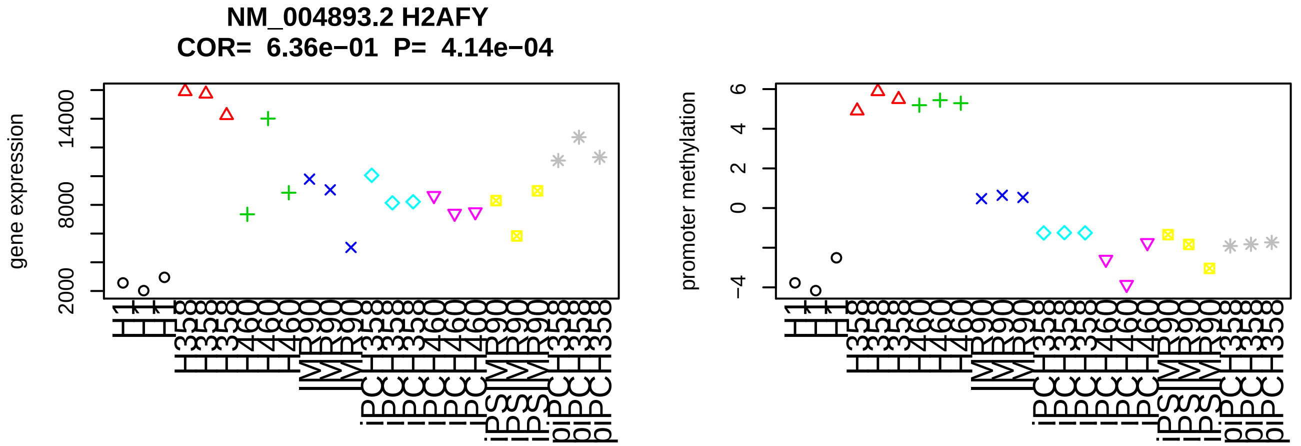
NM_004893.2 H2AFY
COR= 6.36e−01 P= 4.14e−04
gene expression
promoter methylation
2000
8000
14000
−4
0
2
4
6

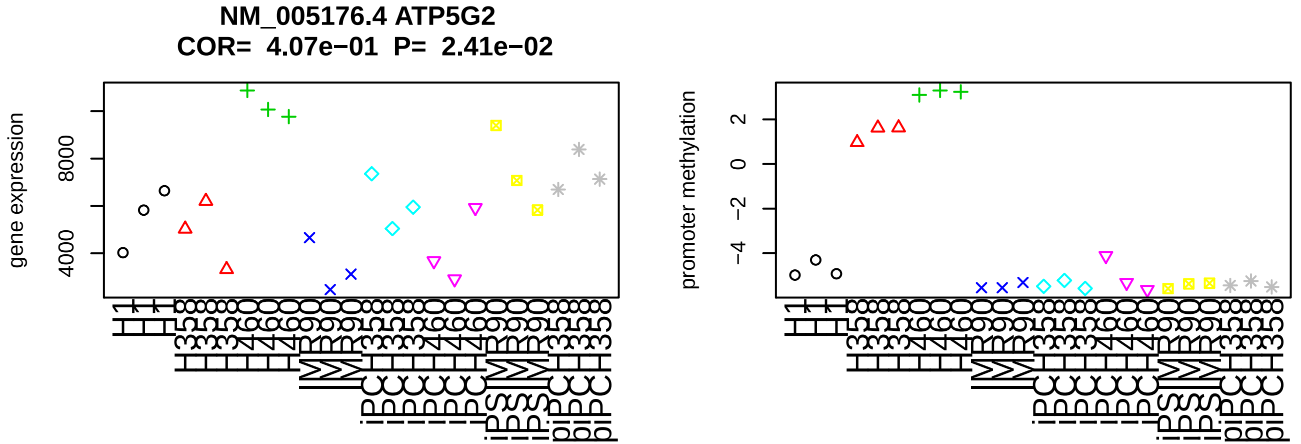
NM_005176.4 ATP5G2
COR= 4.07e−01 P= 2.41e−02
gene expression
promoter methylation
4000
8000
−4
−2
0
2

NM_014220.2 TM4SF1
COR= −7.96e−01 P= 1.61e−06

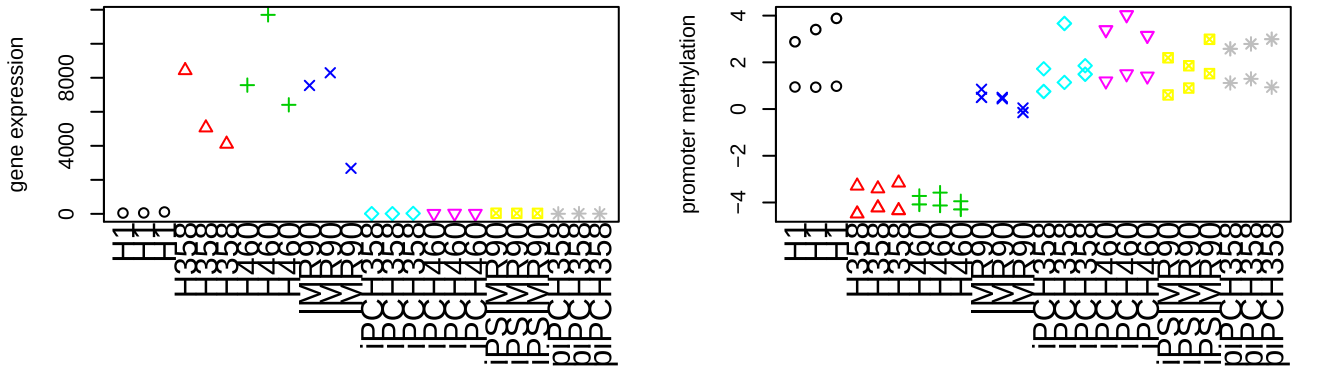

NM_201525.1 GPR56
COR= −8.40e−01 P= 1.42e−07

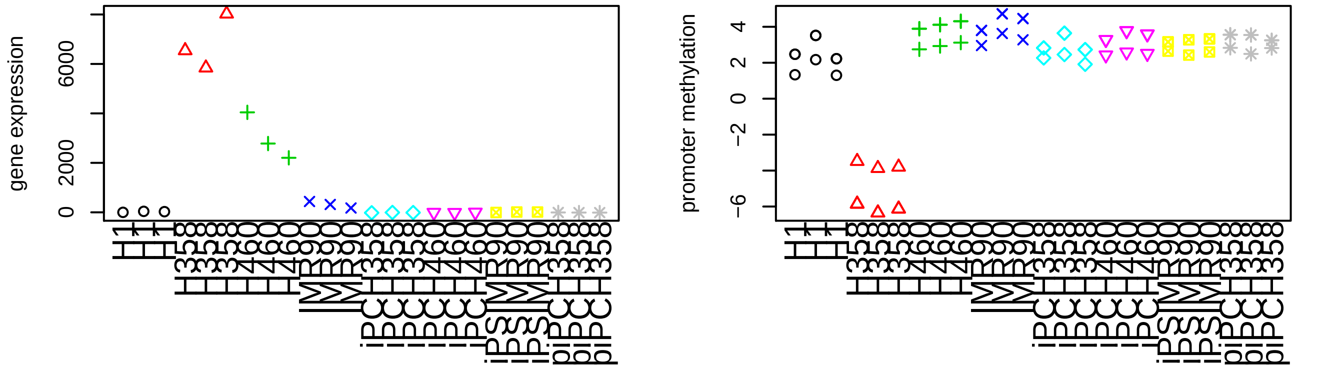

NM_005980.2 S100P
COR= −8.23e−01 P= 3.99e−07

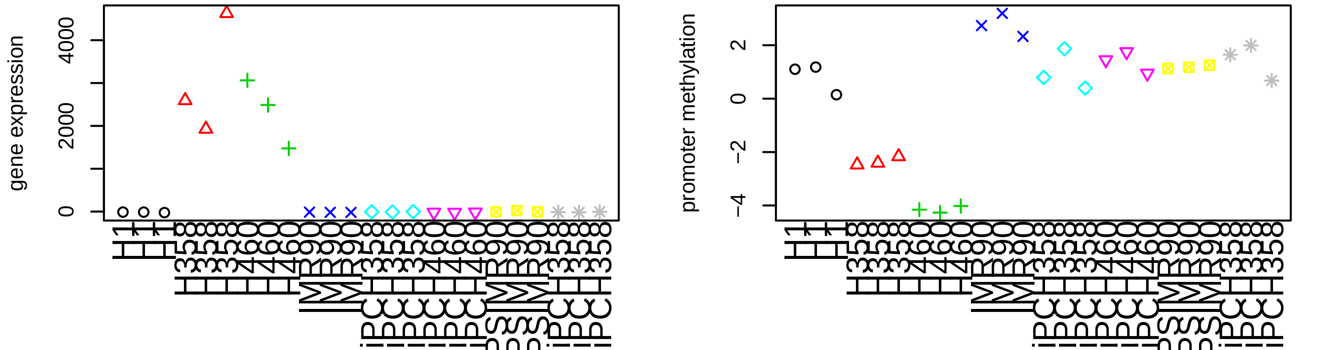

Supplement: Additional file 4: — Genes selected based upon PC3. Gene expression/promoter methylation of genes selected by PCA based unsupervised FE employing PC3. Among eleven selected genes, eight genes had a significant (P < 0.05) negative correlation between gene expression and promoter methylation. Because we did not restrict the selection of genes to those with negative correlations, large numbers of genes with negative correlations demonstrate the feasibility of our methodology. (PDF 26 kb) [file 12920_2016_196_MOESM4_ESM.pdf]
