# Supplementary material for: SFRP1 is a possible candidate for epigenetic therapy in non-small cell lung cancer
Source: BMC Med Genomics. 2016 Aug 12;9(Suppl 1):28. doi: 10.1186/s12920-016-0196-3 (PMC4989892; doi:10.1186/s12920-016-0196-3)

NM_021102.2 SPINT2
COR= −3.71e−01 P= 3.72e−02

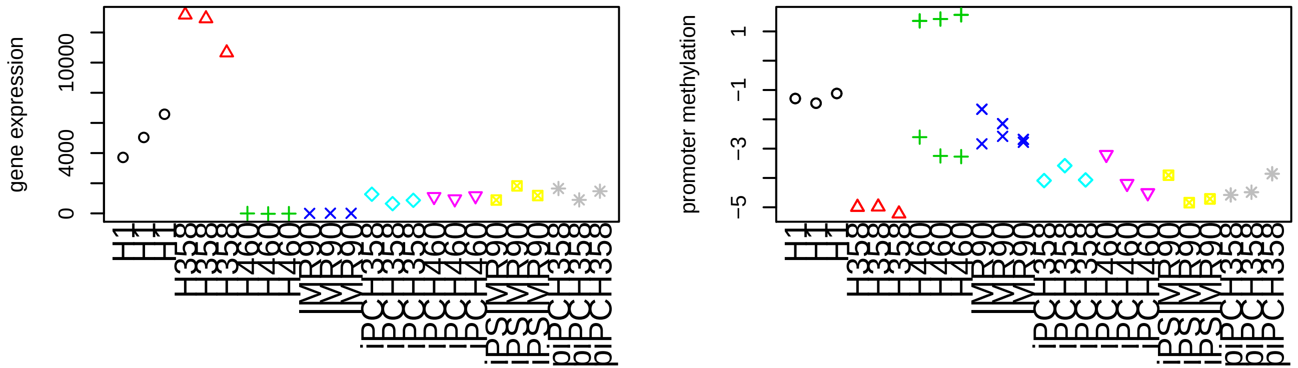

NM_002354.1 TACSTD1
COR= −7.79e−01 P= 3.71e−06

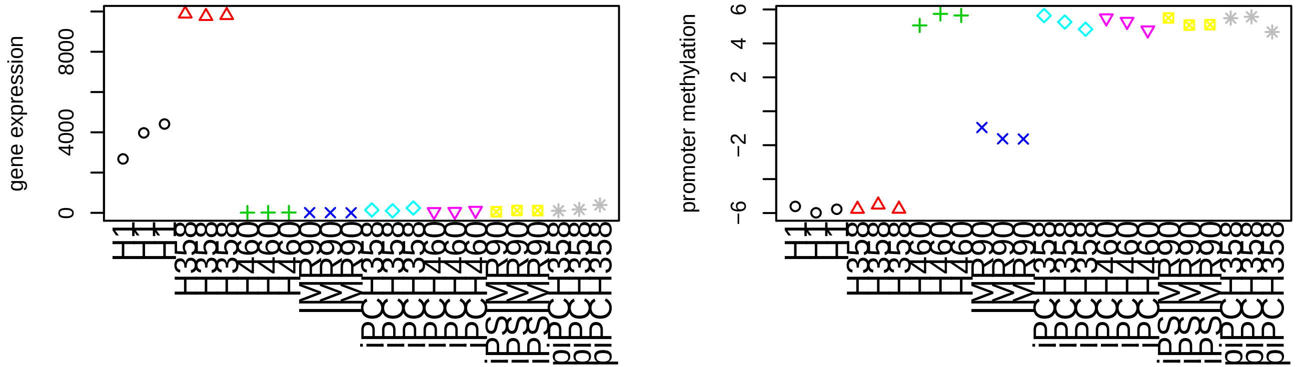

NM_004360.2 CDH1
COR= −4.19e−01 P= 2.07e−02

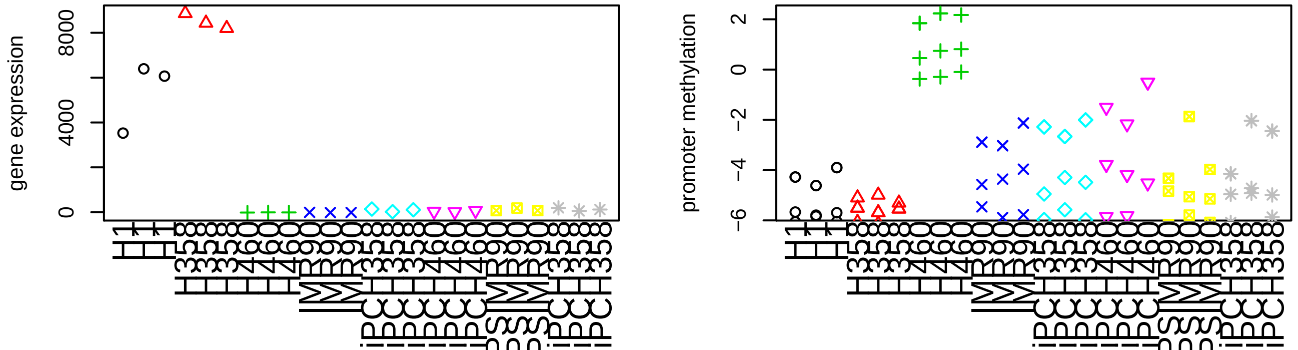

NM_002353.1 TACSTD2
COR= −6.07e−01 P= 8.31e−04

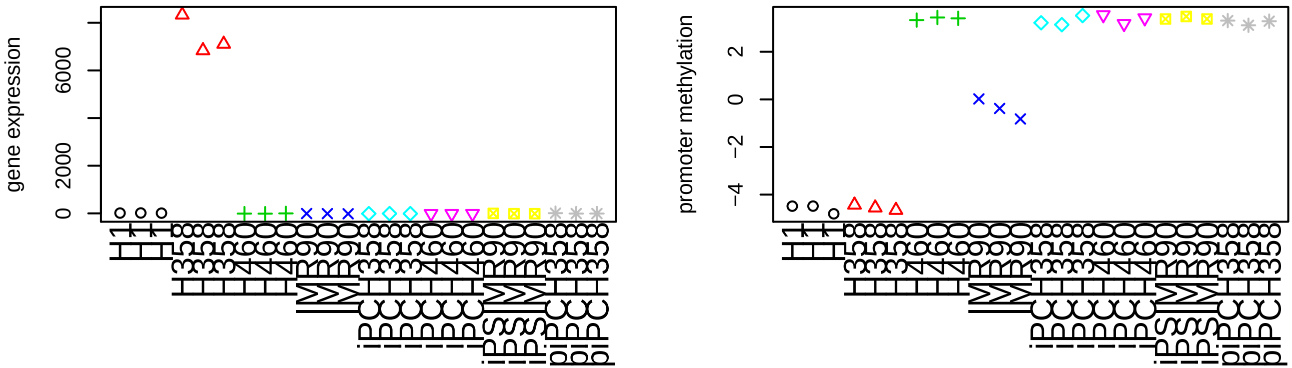

NM_005562.1 LAMC2
COR= −7.07e−01 P= 5.59e−05

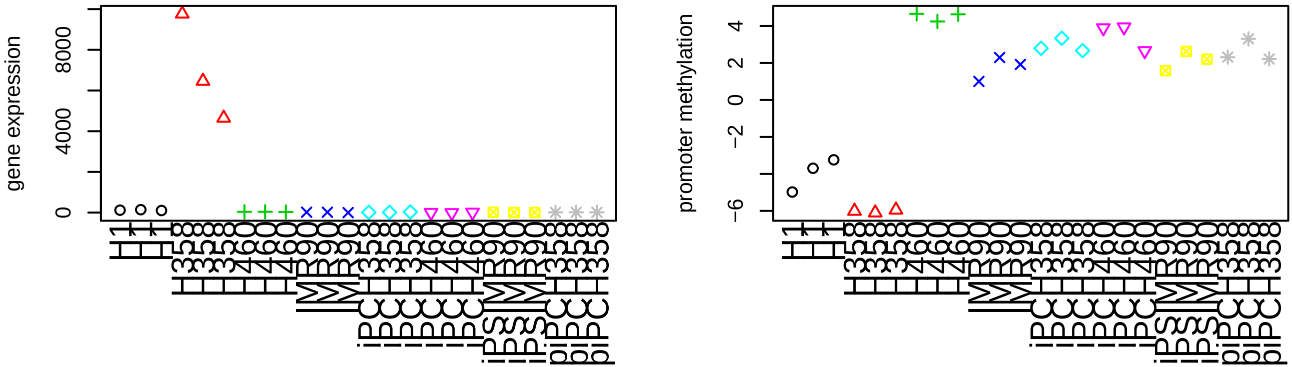

NM_145899.1 HMGA1
COR= −8.23e−01 P= 4.03e−07

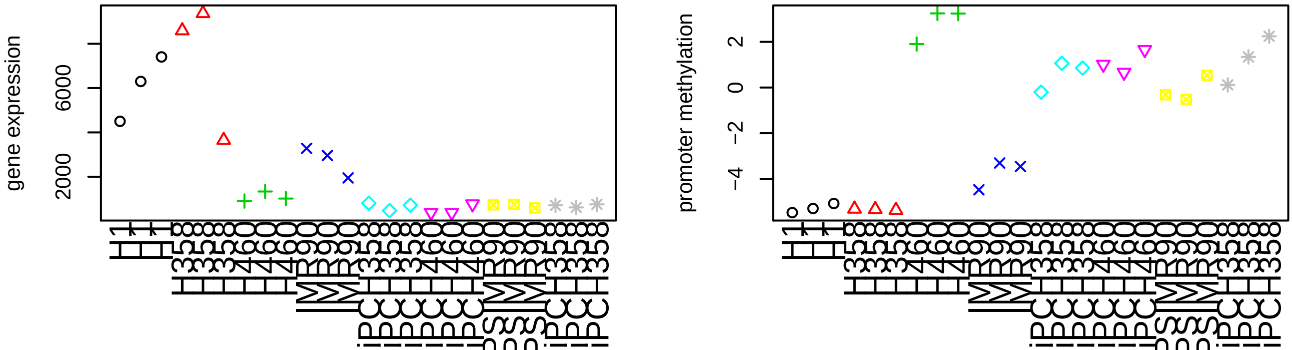

NM_005558.3 LAD1
COR= −7.85e−01 P= 2.76e−06

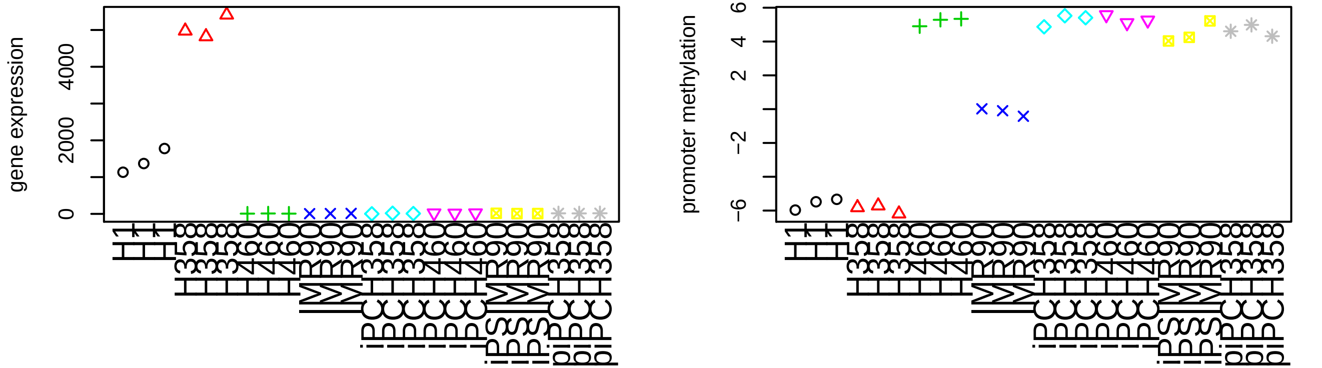

NM_201525.1 GPR56
COR= −8.45e−01 P= 1.00e−07

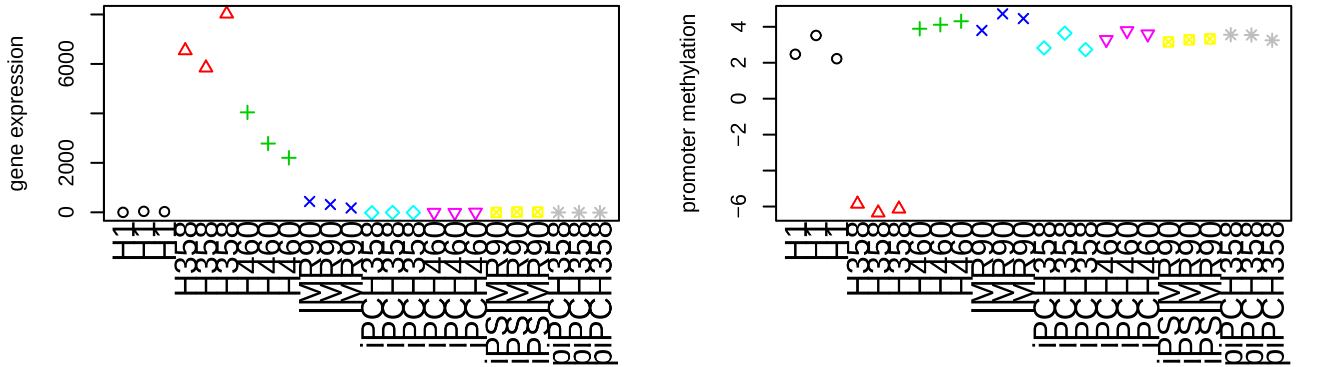

NM_004566.2 PFKFB3
COR= −1.89e−01 P= 1.88e−01

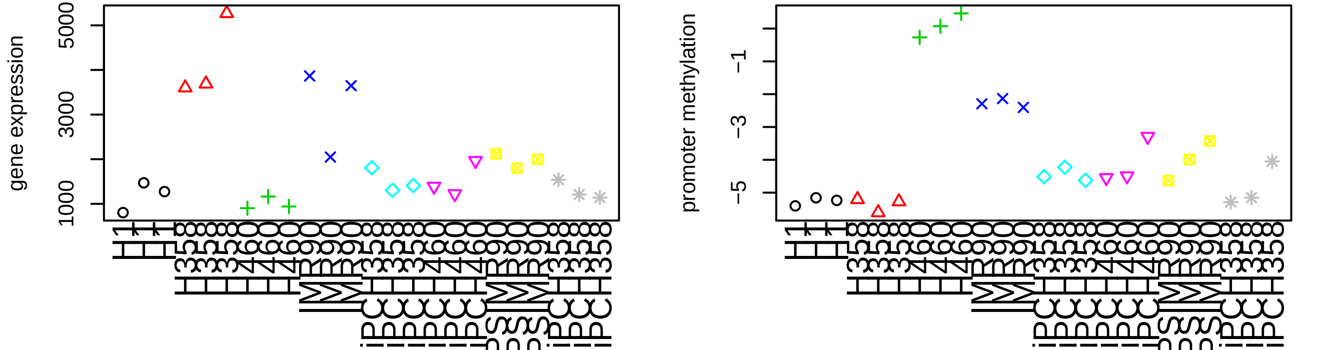

NM_005218.3 DEFB1
COR= 6.08e−01 P= 8.18e−04

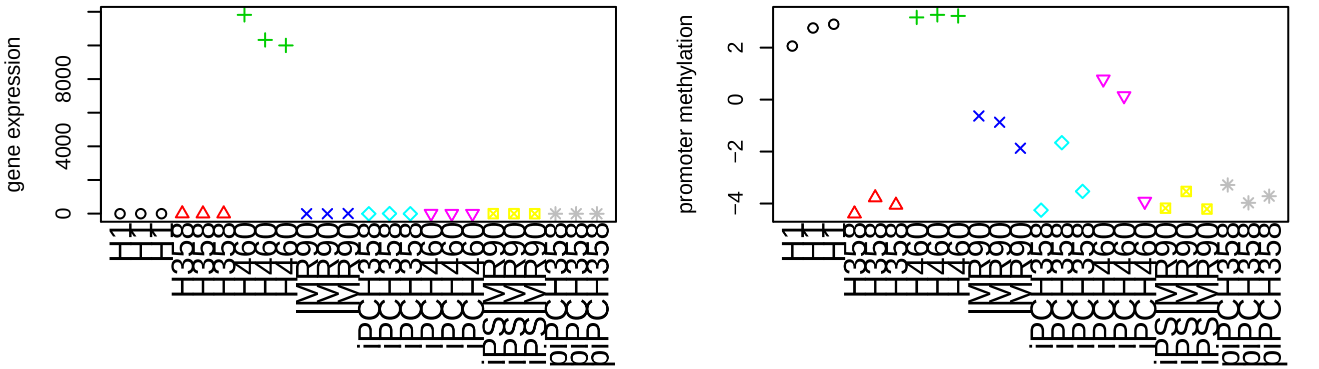

NM_002727.2 SRGN
COR= −9.49e−01 P= 7.88e−13

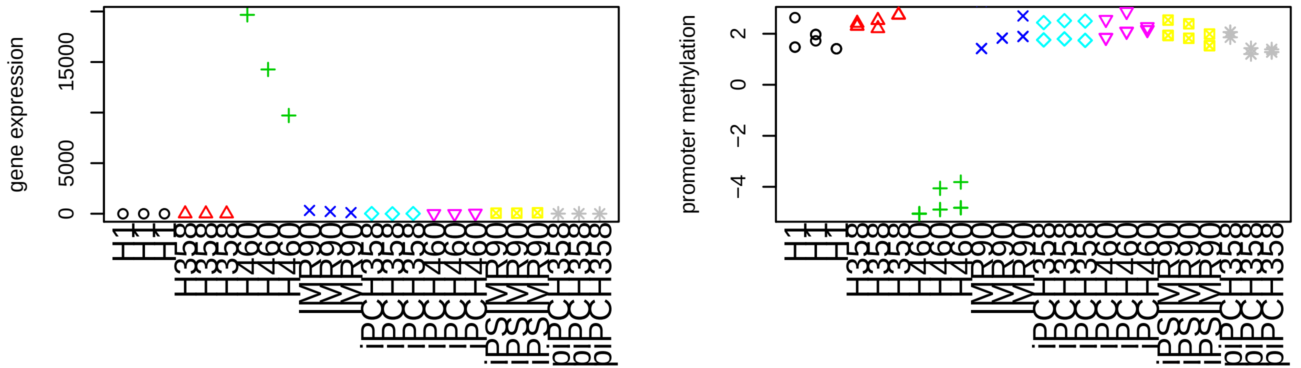

NM_004181.3 UCHL1
COR= −4.87e−01 P= 7.86e−03

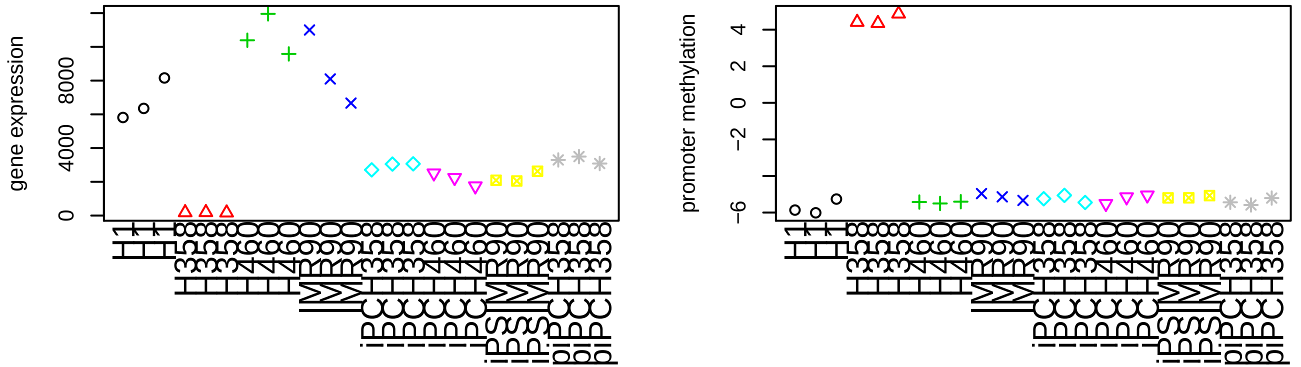

NM_000691.3 ALDH3A1
COR= −5.88e−01 P= 1.25e−03

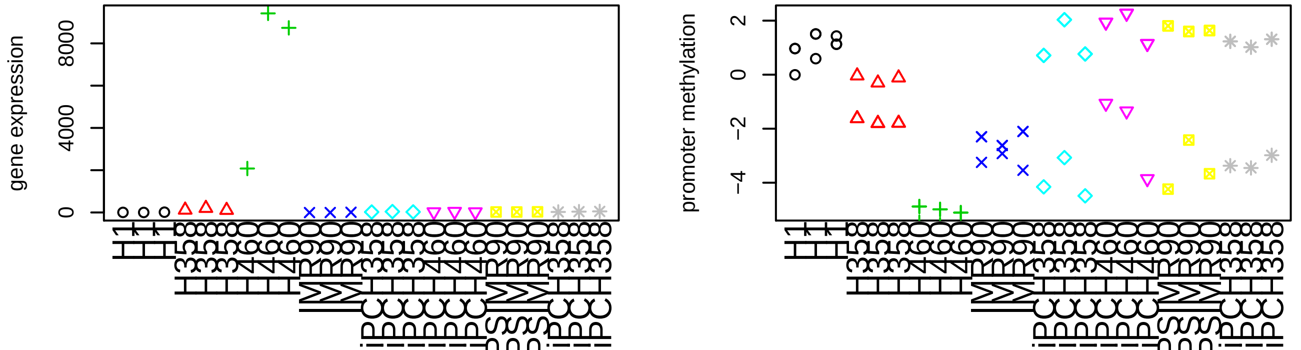

NM_012307.2 EPB41L3
COR= −2.39e−01 P= 1.30e−01

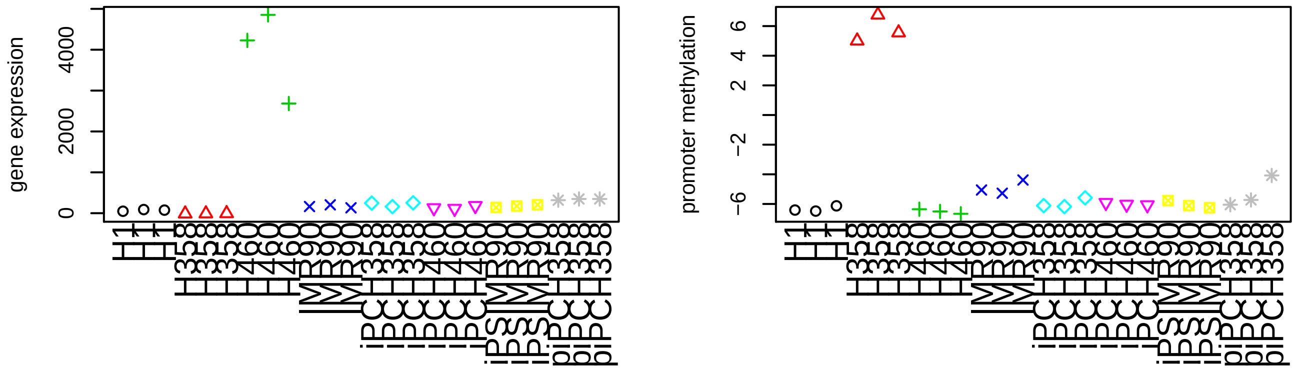

NM_021136.2 RTN1
COR= −3.62e−01 P= 4.10e−02

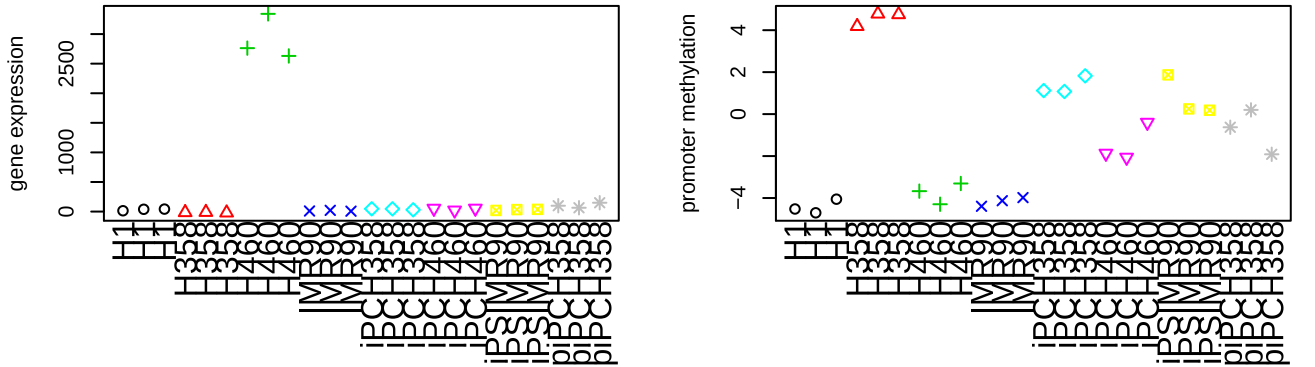

NM_005559.2 LAMA1
COR= −1.92e−01 P= 1.84e−01

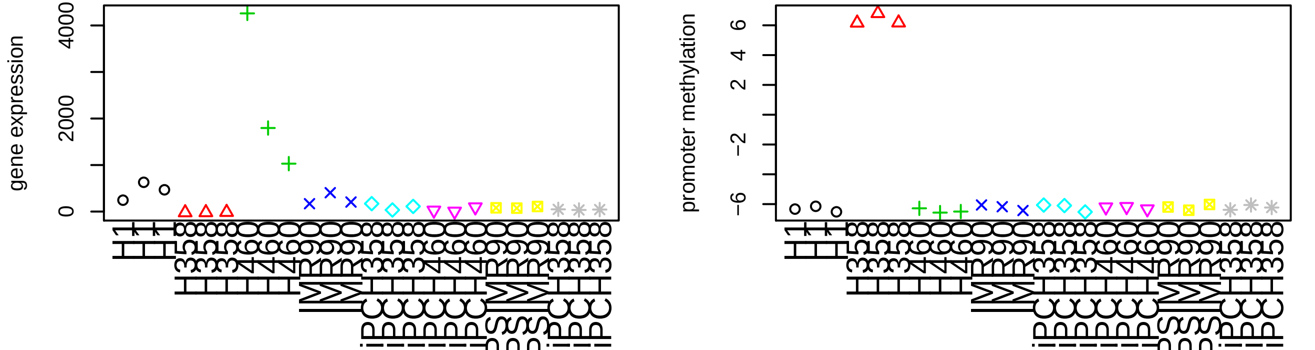

Supplement: Additional file 5: — Genes selected based upon PC4. Gene expression/promoter methylation of genes selected by PCA based unsupervised FE employing PC4. Among 16 selected genes, 12 genes had a significant (P < 0.05) negative correlation between gene expression and promoter methylation. Because we did not restrict the selection of genes to those with negative correlations, large numbers of genes with negative correlations indicate the feasibility of our methodology. (PDF 36 kb) [file 12920_2016_196_MOESM5_ESM.pdf]
