# Supplementary material for: SFRP1 is a possible candidate for epigenetic therapy in non-small cell lung cancer
Source: BMC Med Genomics. 2016 Aug 12;9(Suppl 1):28. doi: 10.1186/s12920-016-0196-3 (PMC4989892; doi:10.1186/s12920-016-0196-3)
Supplement: Additional file 6: — Disease associations of genes in Table 1. Disease associations listed with Gendoo server and associated P-values. (PDF 99 kb) [file 12920_2016_196_MOESM6_ESM.pdf]

Disease associations by gendoo server

Categorical regression

NM_020436.2 SALL4

Plasmacytoma 0.00408
Lymphoma 0.0305

NM_002354.1 TACSTD1 (EPCAM)

Neoplasms 2.61e-21
Colorectal Neoplasms 1.00e-18
Adenocarcinoma 1.73e-15
Colonic Neoplasms 4.25e-14
Carcinoma 1.20e-13
Neoplastic Cells, Circulating 1.76e-13
Bone Marrow Neoplasms 3.00e-12
Lung Neoplasms 6.50e-10
Neoplasm Metastasis 1.02e-9
Breast Neoplasms 1.88e-9
Ovarian Neoplasms 2.81e-9
Stomach Neoplasms 3.32e-7
Lymphatic Metastasis 5.12e-7
Carcinoma, Squamous Cell 9.87e-7
Common Bile Duct Neoplasms 6.98e-5
Carcinoma, Non-Small-Cell Lung 0.000103
Head and Neck Neoplasms 0.000106
Carcinoma, Renal Cell 0.000175
Carcinoma, Signet Ring Cell 0.000179
Retinal Neoplasms 0.000260
Liver Neoplasms 0.000492
Kidney Neoplasms 0.000615
Neoplasms, Glandular and Epithelial 0.000857
Adenocarcinoma, Clear Cell 0.0012
Neoplasm Invasiveness 0.00189
Retinoblastoma 0.00193
Pancreatic Neoplasms 0.00212
Esophageal Neoplasms 0.00262

Prostatic Neoplasms 0.00655
Carcinoma, Small Cell 0.0130
Carcinoma, Papillary 0.0135
Mouth Neoplasms 0.0185
Neoplasms, Squamous Cell 0.0186
Adenocarcinoma, Bronchiolo-Alveolar 0.01968
Adenoma, Oxyphilic 0.0202
Gallbladder Neoplasms 0.0313
Carcinoma, Large Cell 0.0334
Peritoneal Neoplasms 0.0396

NM_001146.3 ANGPT1

Liver Neoplasms 1.21e-109
Glioblastoma 7.27e-7
Astrocytoma 1.53e-6
Brain Neoplasms 2.43e-6
Carcinoma, Hepatocellular 6.44e-6
Adenoma, Liver Cell 1.29e-5
Mammary Neoplasms, Experimental 0.000256
Kidney Neoplasms 0.000860
Breast Neoplasms 0.00109
Goiter 0.00113
Adenocarcinoma 0.00320
Skin Ulcer 0.00787
Colonic Neoplasms 0.00908
Colorectal Neoplasms 0.0140
Ameloblastoma 0.0152
Jaw Neoplasms 0.0168
Carcinoma, Signet Ring Cell 0.0208
Retinal Neoplasms 0.0250
Gastrointestinal Stromal Tumors 0.0261
Uveal Neoplasms 0.0271
Neoplasm Metastasis 0.0316
Esophageal Neoplasms 0.0356
Carcinoma, Renal Cell 0.0362
Ovarian Neoplasms 0.0413
Peritoneal Neoplasms 0.0433
Urinary Bladder Neoplasms 0.0454

NM_002353.1 TACSTD2

Adenocarcinoma 0.00398

Gallbladder Neoplasms 0.00782
Carcinoma 0.0105
Ovarian Neoplasms 0.0127
Colorectal Neoplasms 0.0182
Breast Neoplasms 0.0192
Cell Transformation, Neoplastic 0.0207
Mouth Neoplasms 0.0496

NM_032880.2 IGSF21

NM_004429.3 EFNB1

Neoplasm Invasiveness 0.00801
Adenocarcinoma, Scirrhous 0.000931
Stomach Neoplasms 0.00804
Intestinal Neoplasms 0.0100
Rhabdomyosarcoma 0.0111
Leukemia-Lymphoma, Adult T-Cell 0.0219
Neoplasms 0.0336
Osteosarcoma 0.0432

NM_177524.1 MEST

Carcinoma, Giant Cell 0.000303
Lung Neoplasms 0.000323
Carcinoma, Large Cell 0.00369
Adenocarcinoma 0.00853
Carcinoma, Small Cell 0.0185
Breast Neoplasms 0.0255

NM_013243.2 SCG3

PC3

NM_001992.2 F2R

Neoplasm Invasiveness 1.43e-8

Neoplasm Metastasis 7.95e-4
Melanoma 9.42e-7
Prostatic Neoplasms 2.73e-5
Breast Neoplasms 0.000162
Neoplasms 0.00506
Colonic Neoplasms 0.00612
Glioma 0.0156
Carcinoma, Endometrioid 0.0245
Adenocarcinoma, Clear Cell 0.0251
Brain Neoplasms 0.0295
Adenocarcinoma, Mucinous 0.0311

NM_015881.5 DKK3 (dickkopf WNT signaling pathway inhibitor 3)

Prostatic Neoplasms 1.33e-8
Cell Transformation, Neoplastic 2.78e-6
Lung Neoplasms 1.44e-5
Urinary Bladder Neoplasms 6.34e-5
Carcinoma, Non-Small-Cell Lung 0.000197
Gastrointestinal Neoplasms 0.000222
Carcinoma, Transitional Cell 0.000485
Osteosarcoma 0.00125
Neoplasm Invasiveness 0.00128
Precursor Cell Lymphoblastic Leukemia-Lymphoma 0.00162
Carcinoma, Renal Cell 0.00208
Ganglioneuroma 0.00266
Glioma 0.00397
Kidney Neoplasms 0.00400
Carcinoma, Neuroendocrine 0.00444
Neuroblastoma 0.00634
Neoplasms, Germ Cell and Embryonal 0.00990
Carcinoma 0.0105
Melanoma 0.0110
Colorectal Neoplasms 0.0182
Testicular Neoplasms 0.0256
Lymphoma, Non-Hodgkin 0.0286
Adenocarcinoma 0.0405
Carcinoma, Small Cell 0.0414

NM_003012.3 SFRP1

Colorectal Neoplasms 1.57e-8

Carcinoma, Renal Cell 3.70e-7
Kidney Neoplasms 1.87e-6
Breast Neoplasms 3.43e-6
Cell Transformation, Neoplastic 8.26e-6
Urinary Bladder Neoplasms 2.26e-5
Adenoma 7.18e-5
Esophageal Neoplasms 0.000402
Adenocarcinoma 0.000604
Carcinoma, Hepatocellular 0.00117
Liver Neoplasms 0.00177
Carcinoma, Transitional Cell 0.00216
Pancreatic Neoplasms 0.00280
Carcinoma 0.00442
Precancerous Conditions 0.00496
Neoplasms 0.00587
Urologic Neoplasms 0.00853
Uterine Cervical Neoplasms 0.00885
Leukemia, Myeloid, Acute 0.0101
Hepatoblastoma 0.0159
Sarcoma, Ewing 0.0299
Leiomyoma 0.0306
Carcinoma in Situ 0.0387
Stomach Neoplasms 0.0444

NM_213606.1 SLC16A12

NM_019102.2 HOXA5

Breast Neoplasms 7.69e-13
Papilloma, Intraductal 0.000621
Leukemia, Myeloid, Acute 0.00171
Leukemia, Myeloid 0.00196
Pulmonary Emphysema 0.00333
Hemangioma 0.00413
Precursor B-Cell Lymphoblastic Leukemia-Lymphoma 0.00647
Leukemia, Lymphoid 0.00972
Cell Transformation, Neoplastic 0.0153
Carcinoma, Ductal, Breast 0.0340
Leukemia, Myelogenous, Chronic, BCR-ABL Positive 0.0394
Precursor Cell Lymphoblastic Leukemia-Lymphoma 0.0490

NM_004321.4 KIF1A

NM_004893.2 H2AFY

Cerebellar Neoplasms 0.000657
Medulloblastoma 0.000950

NM_005176.4 ATP5G2

NM_014220.2 TM4SF1

Colorectal Neoplasms 7.62e-5
Neoplasm Invasiveness 8.56e-5
Lung Neoplasms 0.000260
Neoplasms 0.000330
Neoplasm Metastasis 0.00179
Adenocarcinoma 0.00743
Carcinoma, Renal Cell 0.0272
Neoplasm Recurrence, Local 0.0283
Kidney Neoplasms 0.0378
Carcinoma, Non-Small-Cell Lung 0.0450

NM_201525.1 GPR56

See below, PC4

NM_005980.2 S100P (calcium binding protein P)

Pancreatic Neoplasms 3.33e-12
Adenocarcinoma 3.53e-9
Neoplasm Metastasis 2.84e-8
Carcinoma, Pancreatic Ductal 1.05e-6
Prostatic Neoplasms 1.08-6
Neoplasms 4.78e-5
Carcinoma in Situ 0.000230
Breast Neoplasms 0.000535

Ovarian Neoplasms 0.00109
Colonic Neoplasms 0.00130
Neoplasm Invasiveness 0.00206
Urologic Neoplasms 0.00475
Lung Neoplasms 0.00590
Gastrointestinal Stromal Tumors 0.00697
Carcinoma, Non-Small-Cell Lung 0.00755
Mammary Neoplasms, Animal 0.0134
Carcinoma 0.0144
Tumor Virus Infections 0.0145
Adenocarcinoma, Mucinous 0.0182
Cell Transformation, Neoplastic 0.0281
Carcinoma, Transitional Cell 0.0369
Carcinoma, Ductal, Breast 0.0464
Carcinoma, Papillary 0.0494

PC4

NM_021102.2 SPINT2

Ovarian Neoplasms 1.02e-22
Neoplasm Invasiveness 6.55e-14
Peritoneal Neoplasms 7.13e-6
Carcinoma, Hepatocellular 0.000140
Neoplasms 0.000237
Neoplasm Metastasis 0.000251
Kidney Neoplasms 0.000387
Brain Neoplasms 0.00136
Liver Neoplasms 0.00187
Carcinoma, Renal Cell 0.00189
Ureteral Neoplasms 0.00521
Carcinoma 0.0185
Chondrosarcoma 0.0259
Lung Neoplasms 0.0264
Sarcoma, Kaposi 0.0364
Adenocarcinoma, Clear Cell 0.0440
Glioma 0.0445

NM_002354.1 EPCAM

(see categorical regression)

NM_004360.2 CDH1

Stomach Neoplasms 8.67e-150
Neoplasm Invasiveness 3.48e-67
Breast Neoplasms 1.06e-42
Lymphatic Metastasis 2.79e-37
Adenocarcinoma 8.70e-36
Carcinoma, Signet Ring Cell 1.39e-36
Carcinoma, Squamous Cell 1.32e-24
Neoplasm Metastasis 1.12e-21
Colorectal Neoplasms 1.24e-21
Carcinoma 1.30e-21
Carcinoma, Lobular 2.84e-21
Prostatic Neoplasms 1.03e-18
Colonic Neoplasms 1.14e-16
Urinary Bladder Neoplasms 3.79e-12
Carcinoma, Ductal, Breast 1.34e-11
Carcinoma in Situ 2.33e-10
Neoplasm Recurrence, Local 2.82e-10
Neoplastic Syndromes, Hereditary 6.33e-10
Neoplasms 8.77e-9
Carcinoma, Transitional Cell 1.55e-8
Esophageal Neoplasms 2.96e-8
Adenoma 1.75e-7
Liver Neoplasms 2.18e-7
Lung Neoplasms 2.89e-7
Carcinoma, Non-Small-Cell Lung 3.20e-6
Rectal Neoplasms 4.00e-6
Gallbladder Neoplasms 4.21e-6
Carcinoma, Hepatocellular 8.13e-6
Pancreatic Neoplasms 1.26e-5
Ovarian Neoplasms 2.24e-5
Carcinoma, Papillary 2.76e-5
Carcinoma, Ductal 2.94e-5
Uterine Cervical Neoplasms 3.41e-5
Carcinoma, Adenoid Cystic 5.48e-5
Skin Neoplasms 5.60e-5
Thyroid Neoplasms 6.54e-5
Head and Neck Neoplasms 0.000148
Cell Transformation, Neoplastic 0.000217
Melanoma 0.000303
Mucoepidermoid Tumor 0.000533
Mouth Neoplasms 0.000633
Carcinoma, Intraductal, Noninfiltrating 0.000668

Salivary Gland Neoplasms 0.000680
Cervical Intraepithelial Neoplasia 0.000699
Adenocarcinoma, Follicular 0.000745
Meningioma 0.00135
Carcinoma, Pancreatic Ductal 0.00137
Carcinoma, Adenosquamous 0.00179
Plasma Cell Granuloma, Pulmonary 0.00213
Vulvar Neoplasms 0.00224
Sarcoma, Synovial 0.00325
Carcinoma, Large Cell 0.00562
Pulmonary Sclerosing Hemangioma 0.00638
Carcinoma, Verrucous 0.00849
Pseudomyxoma Peritonei 0.00955
Neoplasms, Neuroepithelial 0.0117
Meningeal Neoplasms 0.0124
Adenoma, Villous 0.0132
Pinealoma 0.0138
Neoplasms, Multiple Primary 0.0155
Adenocarcinoma, Mucinous 0.0183
Ureteral Neoplasms 0.0190
Choroid Plexus Neoplasms 0.0190
Gingival Neoplasms 0.0200
Spinal Cord Neoplasms 0.0289
Hypopharyngeal Neoplasms 0.0289
Laryngeal Neoplasms 0.0331
Gastrointestinal Neoplasms 0.0334
Endometrial Neoplasms 0.0344
Bronchial Neoplasms 0.0361
Kidney Neoplasms 0.0363
Duodenal Neoplasms 0.0366
Carcinoma, Mucoepidermoid 0.0382
Biliary Tract Neoplasms 0.0459
Testicular Neoplasms 0.0464
Pituitary Neoplasms 0.0475
Carcinoma, Papillary, Follicular 0.0479

NM_002353.1 TACSTD2

(see categorical regression)

NM_005562.1 LAMC2

Carcinoma, Squamous Cell 2.51e-43

Neoplasm Invasiveness 3.00e-36
Mouth Neoplasms 5.60e-14
Tongue Neoplasms 1.49e-13
Adenocarcinoma 1.27e-11
Neoplasm Recurrence, Local 4.53e-11
Colorectal Neoplasms 6.48e-10
Neoplasm Metastasis 2.05e-8
Esophageal Neoplasms 5.30e-8
Lymphatic Metastasis 1.19e-6
Adenocarcinoma, Clear Cell 7.60e-6
Precancerous Conditions 1.45e-5
Head and Neck Neoplasms 2.90e-5
Cervical Intraepithelial Neoplasia 0.000672
Uterine Cervical Neoplasms 0.00100
Carcinoma, Pancreatic Ductal 0.00107
Stomach Neoplasms 0.00123
Fibrosarcoma 0.00167
Lung Neoplasms 0.00227
Skin Neoplasms 0.00611
Anus Neoplasms 0.00611
Pancreatic Neoplasms 0.00682
Carcinoma, Small Cell 0.00692
Carcinoma 0.0106
Endometrial Neoplasms 0.0108
Neoplasms, Squamous Cell 0.0133
Carcinoma, Adenosquamous 0.0135
Vulvar Neoplasms 0.0151
Carcinoma, Adenoid Cystic 0.0156
Uveal Neoplasms 0.0179
Urinary Bladder Neoplasms 0.0210
Carcinoma, Large Cell 0.0241
Neoplasms, Glandular and Epithelial 0.0299
Carcinoid Tumor 0.0331
Liver Neoplasms 0.0338
Cholangiocarcinoma 0.0372
Bile Duct Neoplasms 0.0410
Carcinoma, Non-Small-Cell Lung 0.0424

NM_145899.1 HMGA1

Breast Neoplasms 2.03e-6
Cell Transformation, Neoplastic 8.98e-5
Neuroblastoma 0.000278

Pancreatic Neoplasms 0.000372
Ganglioneuroblastoma 0.000857
Ganglioneuroma 0.000282
Hamartoma 0.00449
Adenocarcinoma 0.00468
Genital Neoplasms, Female 0.00572
Neoplasms, Germ Cell and Embryonal 0.0105
Neoplasm Metastasis 0.0116
Carcinoma 0.0117
Adenocarcinoma, Follicular 0.0138
Leiomyoma 0.0154
Carcinoma, Pancreatic Ductal 0.0170
Neoplasm Invasiveness 0.0216
Testicular Neoplasms 0.0270
Uterine Neoplasms 0.0357
Carcinoma, Ductal, Breast 0.0421
Carcinoma, Papillary 0.0446
Neoplasms 0.0498

NM_005558.3 LAD1

NM_201525.1 GPR56

Neoplasm Metastasis 0.00154
Melanoma 0.00163
Esophageal Neoplasms 0.0251
Glioblastoma 0.0256
Glioma 0.0350
Pancreatic Neoplasms 0.0489

NM_004566.2 PFKFB3

Neoplasms 4.47e-9
Pancreatic Neoplasms 0.00151
Cell Transformation, Neoplastic 0.0042
Astrocytoma 0.0184
Breast Neoplasms 0.0255
Carcinoma, Renal Cell 0.0291
Glioblastoma 0.0295
Kidney Neoplasms 0.0404

NM_005218.3 DEFB1

Carcinoma, Renal Cell 4.49e-5
Adenoma, Oxyphilic 9.96e-5
Kidney Neoplasms 0.000161
Fibroma 0.0103
Adenoma, Pleomorphic 0.0138
Carcinoma, Adenoid Cystic 0.0152
Plasmacytoma 0.0164
Carcinoma, Squamous Cell 0.0347
Adenocarcinoma, Clear Cell 0.0351
Salivary Gland Neoplasms 0.0356
Cholangiocarcinoma 0.0365
Prostatic Neoplasms 0.0492

NM_002727.2 SRGN

Leukemia, Promyelocytic, Acute 4.87e-5
Leukemia, Myeloid, Acute 0.000194
Multiple Myeloma 0.0188
Leukemia 0.0255

NM_004181.3 UCHL1

Carcinoma, Renal Cell 1.74e-7
Kidney Neoplasms 8.88e-7
Granular Cell Tumor 0.00346
Digestive System Neoplasms 0.00544
Colorectal Neoplasms 0.00649
Neoplasm Metastasis 0.0323

NM_000691.3 ALDH3A1

Lung Neoplasms 3.04e-5
Mouth Neoplasms 0.00417
Breast Neoplasms 0.00434
Neoplasms, Germ Cell and Embryonal 0.00584
Adenocarcinoma 0.0150
Carcinoma, Small Cell 0.0246
Neoplasm Recurrence, Local 0.0403

NM_012307.2 EPB41L3

Meningioma 2.41e-23
Meningeal Neoplasms 1.17e-12
Lung Neoplasms 3.24e-10
Carcinoma, Non-Small-Cell Lung 8.60e-10
Breast Neoplasms 6.88e-7
Spinal Cord Neoplasms 1.95e-6
Ependymoma 1.00e-5
Neurilemmoma 1.50e-5
Neoplasm Metastasis 0.000377
Neurofibromatosis 2 0.00243
Brain Neoplasms 0.00645
Carcinoma 0.00879
Central Nervous System Neoplasms 0.00882
Intestinal Neoplasms 0.0107
Prostatic Neoplasms 0.0281
Astrocytoma 0.0377

NM_021136.2 RTN1

Lung Neoplasms 2.76e-9
Carcinoma, Small Cell 3.21e-9
Neuroendocrine Tumors 8.60e-9
Carcinoma, Neuroendocrine 1.78e-6
Carcinoid Tumor 1.21e-5
Carcinoma, Non-Small-Cell Lung 1.56e-5
Neuroblastoma 0.00124
Neuroectodermal Tumors, Primitive, Peripheral 0.00184
Adenoma, Islet Cell 0.00196
Carcinoma, Squamous Cell 0.00527
Adenocarcinoma 0.00853
Adrenal Gland Neoplasms 0.0137
Pheochromocytoma 0.0164
Thyroid Neoplasms 0.0431

NM_005559.2 LAMA1

Choriocarcinoma 2.63e-12
Fibrosarcoma 1.23e-5
Uterine Neoplasms 5.78e-5
Carcinoma, Adenoid Cystic 6.31e-5
Salivary Gland Neoplasms 0.000350

Carcinoma 0.00442
Myoepithelioma 0.00487
Adenoma, Oxyphilic 0.0106
Choristoma 0.0120
Neurilemmoma 0.0129
Tongue Neoplasms 0.0164
Glioma 0.0169
Kidney Neoplasms 0.0170
Lung Neoplasms 0.0288
Wilms Tumor 0.0301
Brain Neoplasms 0.0319
Meningioma 0.0336
Gastrointestinal Neoplasms 0.0447
