# Supplementary material for: SFRP1 is a possible candidate for epigenetic therapy in non-small cell lung cancer
Source: BMC Med Genomics. 2016 Aug 12;9(Suppl 1):28. doi: 10.1186/s12920-016-0196-3 (PMC4989892; doi:10.1186/s12920-016-0196-3)
Supplement: Additional file 7: — KEGG pathways associated with genes in Table 1. Pathway image files downloaded from KEGG and the html file is linked to these images. (ZIP 963 kb) [file 12920_2016_196_MOESM7_ESM.zip › Kegg_Figs/Search PATHWAY.html]

Search PATHWAY


### Pathway Search Result

Following object(s) was/were not found ko:ALDH3A1 ko:DEFB1 ko:DKK3 ko:EFNB1 ko:EPB41L3 ko:EPCAM ko:GPR56 ko:H2AFY ko:HMGA1 ko:HOXA5 ko:IGSF21 ko:KIF1A ko:LAD1 ko:LAMA1 ko:MEST ko:PFKFB3 ko:RTN1 ko:S100P ko:SALL4 ko:SCG3 ko:SLC16A12 ko:SPINT2 ko:SRGN ko:TM4SF1 ko:TROP2

---


Sort by the pathway list

Show all objects

```
- ko04151 PI3K-Akt signaling pathway (3)

  ko:ANGPT1 
  ko:F2R 
  ko:LAMC2
- ko04015 Rap1 signaling pathway (2)

  ko:ANGPT1 
  ko:F2R
- ko05200 Pathways in cancer (2)

  ko:F2R 
  ko:LAMC2
- ko04611 Platelet activation (1)

  ko:F2R
- ko04120 Ubiquitin mediated proteolysis (1)

  ko:CDH1
- ko05146 Amoebiasis (1)

  ko:LAMC2
- ko04810 Regulation of actin cytoskeleton (1)

  ko:F2R
- ko04014 Ras signaling pathway (1)

  ko:ANGPT1
- ko05323 Rheumatoid arthritis (1)

  ko:ANGPT1
- ko04610 Complement and coagulation cascades (1)

  ko:F2R
- ko04510 Focal adhesion (1)

  ko:LAMC2
- ko05145 Toxoplasmosis (1)

  ko:LAMC2
- ko05012 Parkinson's disease (1)

  ko:UCHL1
- ko04111 Cell cycle - yeast (1)

  ko:CDH1
- ko04066 HIF-1 signaling pathway (1)

  ko:ANGPT1
- ko04914 Progesterone-mediated oocyte maturation (1)

  ko:CDH1
- ko04512 ECM-receptor interaction (1)

  ko:LAMC2
- ko05222 Small cell lung cancer (1)

  ko:LAMC2
- ko04024 cAMP signaling pathway (1)

  ko:F2R
- ko04144 Endocytosis (1)

  ko:F2R
- ko04310 Wnt signaling pathway (1)

  ko:SFRP1
- ko04080 Neuroactive ligand-receptor interaction (1)

  ko:F2R
- ko04020 Calcium signaling pathway (1)

  ko:F2R
- ko04110 Cell cycle (1)

  ko:CDH1
```

{"version":"0.1.0.562","isExtension":true,"extensionName":"Chrome"}
