# Supplementary material for: SFRP1 is a possible candidate for epigenetic therapy in non-small cell lung cancer
Source: BMC Med Genomics. 2016 Aug 12;9(Suppl 1):28. doi: 10.1186/s12920-016-0196-3 (PMC4989892; doi:10.1186/s12920-016-0196-3)
Supplement: Additional file 8: — Literature searches of genes in Table 1. (PDF 127 kb) [file 12920_2016_196_MOESM8_ESM.pdf]

Journal, Vol. XXI, No. 1, 1-5, 2013
Additional note

Supplementary document for "SFRP1 is possible candidate of epigeteic therapy in non-small cell lung cancer"

Y-h. Taguchi[1]*, Mitsuo Iwadate[2], Hideaki Umeyama[2]

Abstract
Literature search was performed for 31 genes selected by our methodology

Keywords
Non-small cell lung cancer — histone deacetylase inhibitor — feature extraction — principal component analysis

[1] *Department of Physics, Chuo University, 1-13-27 Kasuga, Bunkyo-ku, Tokyo 112-8551, Japan*
[2] *Department of Biological Science, Chuo University, 1-13-27 Kasuga, Bunkyo-ku, Tokyo 112-8551, Japan*
***Corresponding author**: tag@granular.com

SALL4

Transcription factor SALL4 was generally known to be a master regulator that contributes to cell stemness in biological development and tumor growth [1].

tumorgenesis

Rodriguez et al [2] found that the expressions of SALL4 and OCT4 were correlated with the tumor differentiation, pathological stage, and patients' clinical information in NSCLC (On the relationship between Oct4 and SALL4, see the next subsection below, too). In addition to this, Kobayashi et al [3] found overexpression of SALL4 in NSCLC compared with normal tissue. They also found that suppression of SALL4 expression revealed catastrophic growth inhibition of SBC-1 lung cancer cells. Although there were not so many reported direct evidences between NSCLC and SALL4, since there were huge number of studies that suggest relationships between SALL4 and various cancers, SALL4 is highly likely to be related to NSCLC, too. In addition to this, SALL4 was reported to bind to various genes that have known relationship with NSCLC. For example, SALL4 was reported to bind to SUMO1 [4] while association of SUMO1 and UBC9 genotypes with tumor response was reported in NSCLC treated with irinotecan-based chemotherapy [5]. SALL4 was also know to bind to ELAVL1, also known as HUR, [6], whose expression in NSCLC correlates with vascular endothelial growth factor-C expression and lymph node metastasis [7]. SALL4 was also related to OCT4 and NANOG [8] that have huge number of reported studies that relate to NSCLC (e.g., [9]).

pluripotency

Buganim et al [10] found that ectopic expression of SALL4, Nanog, Esrrb, and Lin28 (SNEL) in mouse embryonic fibroblasts (MEFs) generated high-quality iPSCs more efficiently than other combinations of factors including Oct4, Sox2, Klf4, and Myc (OSKM), which were well known reprogramming factors. In this regard, Yang et al [11] identified positive feedback loop between Oct4 and SALL4. They also found that SALL4 also has suppressive self-feedback and is suppressor of other SALL family members, which competes with the activation of these genes by OCT4. Their findings indicated that SALL4 is a master regulator that controls its own expression and the expression of OCT4. Ni et al [12] found that sphere formation may render somatic cells more susceptible to reprogramming, since Oct4 alone is sufficient to reprogram monolayer-cultured adult mouse ciliary body epithelial cells to iPS cells through sphere formation. SALL4 was expressed together with OCT4 in this experiment.

TACSTD1/2

TACSTD1/2 (type I membrane proteins, also known as EpCAM and TROP2, respectively) form TACSTD family.

tumorgenesis

Although the roles of EpCAM and TROP2 are not yet fully understood, both proteins are thought to participate in growth and proliferation of carcinoma cells. EpCAM can transduce an intracellular signal through its cleavage of an intracytoplasmic portion [13]. TROP2 is also believed to be a true oncogene involved in initiating signaling mechanisms that can result in increased tumorigenicity, aggressiveness, and metastasis [14].

Pak et al [15] recently investigated TACSTD1/2 expression and tried to relate their expression to clinicopathologic factors in NSCLC. They considered two subtypes, (AdC) and squamous cell carcinoma (SCC), and found many clinicopathologic factors related to TACSTD1/2 expression. Eberlein et al [16] investigated EpCAM function in NSCLC in the relation to cancer-associated fibroblasts. They found that tu-

mour cells that activated fibroblasts were associated with E-Cadherin and EpCAM expression and expression of integrin $\alpha v\beta 6$. Co-culture of activating tumour cells with fibroblasts resulted in induction of transcripts associated with tumour cell invasion and growth, TGFβ1 and TGFBR1, SERPINE-1, BMP6, SPHK1 and MMP9. This strongly suggested that EpCAM contributed to NSCLC progression through activation of cancer-associated fibroblasts. Liao et al [17] successfully identified the cell migration and invasion abilities in NSCLC metastasis, by targeting EpCAM-positive circulating tumor cells using gemcitabine via the HGF/cMET pathway. This also suggested that EpCAM induces metastasis in NSCLC through the activation of HGF/cMET pathway. There were several studies that report binding of EPCAM to NSCLC related genes. For example, although EpCAM was repoted to bind to MDM2 [18], MDM2 was also known to play critical roles in NSCLC, e.g., mutation was related to NSCLC [19], it interacts with RASSF3 whose downregulation increases malignant phenotypes of NSCLC [20], p53 stabilazation in NSCLC by inhibition of MDM2 by lncRNA MEG3 was reported [21], and p53 loss by MDM2 results in DDX3 loss that promotes tumor malignancy and poor patient outcome in NSCLC [22], MDM2 knockdown could inhibit tumor growth via induction of cell cycle arrest and cancer cell apoptosis in NSCLC [23], MDM2 overespression induced DNMT3A that silences genes including tumor suppressor genes in NSCLC [24]. EpCAM was also known to bind to CFTR [25] whose promoter hypermethylation were associated with clinical/pathological features in NSCLC [26]. Although there are some additional reports about aberrant expression of TROP2 in NSCLC [27, 28], no reports existed about mechanical background how TROP2 induces progression of NSCLC. However, TROP2 was reported to bind to SIRT3 [29] that regulates cell proliferation and apoptosis related to energy metabolism in NSCLC cells through deacetylation of NMNAT2 [30].

pluripotency

Huang et al [31] reported that EpCAM complex proteins promote transcription factor-mediated pluripotency reprogramming. They found that OSKM infected mouse embryonic fibroblasts (MEFs) indicated that EpCAM and Cldn7 were upregulated during reprogramming and that inhibition of either EpCAM or Cldn7 expression resulted in impairment in reprogramming efficiency, whereas overexpression of EpCAM, EpCAM plus Cldn7, or EpCAM intercellular domain (EpICD) significantly enhanced reprogramming efficiency in MEFs. They also suggested that EpCAM signaling may enhance reprogramming through up-regulation of Oct4 and possible suppression of the p53-p21 pathway. There are more reports that suggest relation between expression of EpCAM and pluripotency. There were no reported studies about the relationship between pluripotency and TROP2.

ANGPT1

ANGPT1, that is an abbreviation of Angiopoietin 1, is protein that regulates angiogenesis as its name says. As angiogenesis is an important step to cancer progression, it is likely that ANGPT1 plays critical roles in tumorgenesis. It is also recognised to be expressive during the differentiation from embryonic tissues and stem cells.

tumorgenesis

Takahama et al [32] investigated angiogenesis-associated genes expression in NSCLC with the comparison of paired adjacent normal tissues. They have found that several genes including ANGPT1 were expressive in NSCLC than in normal tissues. Based on the correlation analysis, they have concluded that ANGPT1 as well as VEGF are important angiogenic factors in human NSCLC. There were more researches that reported critical roles of ANGPT1 in NSCLC, in collaboration with TIE2 and VEGF [33, 34]. TIE2, or known as also TEK, was reported to bind to ANGPT1 [35] and was known to be upregulated in early-stage NSCLC as one of genes associated with the angiogenic process [36]. TEK was targeted by XL-184, kinase inhibitor, for the treatment of NSCLC [37]. This can suggest the mechanism by which ANGPT1 contributes to the progress of NSCLC and the way by which NSCLC was treated with targeting ANGPT1, too.

pluripotency

Joo et al [38] concluded that ANGPT1/TIE2 signaling has a pivotal role in embryonic stem cell (ESC)- endothelial cell (EC) differentiation and that this effect can be exploited to expand EC populations.

IGSF21

IGSF21 encodes a protein which has two immunoglobulin (Ig) domains and is a member of the immunoglobulin superfamily. Proteins in this superfamily are usually found on or in cell membranes and act as receptors in immune response pathways.

tumorgenesis

Although there were no reports about the direct relationship between IGSF21 and NSCLC, IGSF21 was recently suggested to have protein-protein interaction with HSPB1, KRAS, TMSB4X and DGKD, based on bioinformatic analysis [39]. HSPB1, also known as HSP27, was extensively reported to be related to NSCLC [40, 41, 42, 43, 44, 45, 46, 47]. KRAS was also extensively reported to be related to NSCLC [48, 49, 50, 51, 52, 53, 54, 55]. Thus, it was plausible that IGSF21 has tight relationship with NSCLC as well. Bindings to other proteins were also validated experimentally. For examples, IGSF21 was reported to bind to GADD45A [56] while GADD45A was downregulated in the group of NSCLCs with telomere shortening [57], treatment of A549 cells with a low concentration of sagopilone revealed an upregulation of direct transcriptional target genes of GADD45A [58], hypermethylation of growth arrest GADD45A in NSCLC was observed [59], GADD45A was upregulated in A549 cell treated with

some marine derived agents [60]. IGSF21 was also reported to bind to GSK3B [56] that was listed as one of biomarkers of cisplatin sensitivity in NSCLC cells [61], to PAEP, also known as GD [62], immunolocalization of which was observed in NSCLC [63], to ANXA3 [62] whose expression was significantly correlated with survival in NSCLC [64], to ATF3 [62] whose expression increased by the treatment of nelfinavir and bortezomib that synergistically inhibited cell proliferation and induced cell death in NSCLC [65], to ALDH2 [62] whose SNP was associated with the phase II NSCLC [66]. Thus, although binding of IGSF21 to other proteins does not always help to understand its functionalities related to NSCLS, it is very likely that binding of IGSF21 to other proteins play critical roles in NSCLC.

pluripotency

Proteome profiling of mouse embryonic stem cells identified HSPB1 as markers for cell differentiation and embryotoxicity [67]. In addition, pluripotent stem cells have an antioxidative system comprising HSPB1 [68]. Resveratrol inhibits pancreatic cancer stem cell characteristics in human and KRAS^{G12D} transgenic mice by inhibiting pluripotency maintaining factors [69]. HSPD1, and KRAS were reported to be upregulated in human embryonic germ cells [70]. Thus, even if there were no reports that observe direct relationship between IGSF21 and pluripotency, IGSF21 can have tight relationship with pluripotency through the interaction with HSPD1 and/or KRAS.

EFNB1

The protein encoded by this gene is a type I membrane protein and a ligand of Eph-related receptor tyrosine kinases. It may play a role in cell adhesion and function in the development or maintenance of the nervous system. EFNB1 was also known as LERK2.

tumorgenesis

Downregulation of EFNB1 leading to reduced tyrosine phosphorylation of EphB3 and resulting in the activation of NSCLC was reported to be in NSCLC [71]. EphB3 was reported to be related to NSCLC in some studies [72, 73]. Thus, EFNB1 is likely to be related to NSCLC through binding to EphB3. EFNB1 was also reported to bind to another Eph-related receptor, EPHA3 that was also known as HEK [74]. EPHA3 was also reported to be related to lung cancer [75, 76]. EPHB1 was another Eph-related receptor, to which EFNB1 was reported to bind [77]. EPHB1 was also reported to be related to NSCLC [78]. Ref. [78] also reported various mutated Eph-related receptors seems to be related to NSCLC. As a conclusion, EFNB1 is likely to be related to NSCLC through binding to various Eph-related receptors.

pluripotency

EphB3 and EphA3 were reported to be expressive in bone marrow mesenchymal stromal cells if compared with bone marrow cell-derived hematopoietic stem/progenitor cells [79]. Since EFNB1 was supposed to bind to these two Eph-related receptors, it is likely to play potential roles during reprogramming.

MEST

MEST, also known as PEG1, encodes a member of the alpha/beta hydrolase superfamily.

tumorgenesis

Frequent loss of imprinting of MEST has been reported in lung AdCs, a subtype of NSCLC [80, 81]. MEST was reported to bind to APP [82] whose expression was altered associated with alternative splicing in NSCLC [83], to DBN1 [56] that was reported to be postoperative recurrence-free survival biomarker of NSCLC [84], and to have protein-protein interaction with ABCD3 [85] that was reported to be one of useful biomarkers of NSCLC [86].

pluripotency

Tada et al [87] compared somatic (embryonic germ (EG)-thymic lymphocyte hybrid) cells with EG that has similar epigenotype to reprogrammed primordial germ cells. They found that observed epigenetic modifications were heritable and affected gene expression as judged by re-activation of the silent maternal allele of PEG1/MEST imprinted gene in the somatic nucleus. Jiang et al [88] investigated pluripotent parthenogenetic embryonic stem (pES) cells and found activation of MEST.

SCG3

SCG3 encodes the gene that is a member of the chromogranin/secretogranin family of neuroendocrine secretory proteins.

tumorgenesis

Moss et al [89] investigated SCG3 expression in peripheral blood of lung cancer patients and found that 16% of NCSLC exhibits SCG3 expression. Välk et al [90] investigated gene expression of NSCLC and found that SCG3 is downregulated in NSCLC. SCG3 was also reported to bind to CHGA [91] that was reported to be related to NSCLC [92, 93] and to DNM1L [94] that interacts with PKP3 that was freequently upregulated in NSCLC [95], and to LYN [96] that was suggested to be related to resistance to cetuximab observed in NSCLC [97]. Thus SCG3 likely contributes to tumorgenesis of NSCLC directly or through binding to CHGA and/or DNML, LYN.

pluripotency

SCG3 was reported to be expressive in REST-deficient ES cell [98].

F2R

F2R is a 7-transmembrane receptor involved in the regulation of thrombotic response. Proteolytic cleavage leads to the activation of the receptor. F2R is a G-protein coupled receptor family member.

tumorgenesis

Huang et al [99] observed gene expression of a human lung AdC cell line, CL1-5, and found that F2R was upregulated and suggested to be involved in the calcium signaling pathway (hsa04020, KEGG). Guo et al [100] also previously reported aberrant methylation of F2R in A549 cell line, which is related to drug resistance. Martinez [101] et al measured gene expression of a nontumorigenic (HPL1A) and a malignant, tumorigenic lung cell line (A549) and found that F2R was the target of 2,3,7,8-Tetrachlorodibenzo-p-dioxin, exposure to high levels of which is associated with chronic obstructive pulmonary disease and lung cancer. F2R was reported to interact with PROCR [102] whose expression was altered by radiotherapy in NSCLC [103]. PROCR and F2R bind to CAV1 [102] that was reported to be downregulated in NSCLC [104]. Although there were no reports that studied interaction between PROCR and F2R in NSCLC, they were reported to interplay in malignant pleural mesothelioma (MPM) [105] (F2R mediate progression of MPM while PROCR suppress tumor growth). Thus, it is not surprising if these two interplay in NSCLC, too. F2R was reported to bind to GNA13 [106] that was identified as biomarker of gemcitabine that was one of the most widely used drugs for the treatment of advanced NSCLC [107]. F2R was also reported to interact with SNX1 [108], a protein that interacts with EGFR, exhibited negative regulation of EGFR trafficking out of early to late endosomes in gefitinib-resistant NSCLC cell lines [109]. Finally, F2R was reported to bind to PDCD6IP [110] whose SNP was recently reported to be relate to NSCLC [111] although there were no known mechanisms that describe the direct interaction between F2R and PDCD6IP.

pluripotency

Yasuda et al [112] identified F2R as one of upregulated genes during the cardiac differentiation of pluripotent stem cells. Layden et al [113] identified F2R as one of highly expressed G protein coupled receptors in embryonic stem cells. Sainz et al [114] found that F2R was expressed in mouse stem cell lines.

DKK3

The secreted protein encoded by DKK3 contains two cysteine rich regions and is involved in embryonic development through its interactions with the Wnt signaling pathway.

tumorgenesis

Nozaki et al [115] found that DKK3 was downregulated in NSCLC tissue than normal tissues. They demonstrated that expression of the exogenous DKK3 gene in NSCLC tumor cells inhibited cell growth [116]. Kobayashi et al [117] found that DDK3 downregulation in NSCLC was mediated by promoter methylation. Since DKK3 was one of Wnt antagonists, downregulaton of DKK3 mediated by promoter metylation activated Wnt signalling pathway, which resulted in progression of NSCLC [118]. Adenovirus vector of DKK3 was even used for NSCLC therapy [119].

pluripotency

DKK3 protein was internalized specifically by differentiated cells located at the periphery of embryoid bodies [120]. Karamariti et al [121] showed that DKK3 mediated Smooth muscle cells differentiated from reprogrammed embryonic lung fibroblasts, through transcriptional regulation of SM22 by potentiation of Wnt signaling. Bharti et al [122] showed that DKK3 promotes retina formation in embryos by inhibiting canonical WNT signaling and stimulating the expression of retinogenic genes, including Six6 and Vsx2. DKK3 was also reported to be reduced in induced neural precursor cells from fibroblast [123].

SFRP1

SFRP1 encodes a member of the SFRP family that contains a cysteine-rich domain homologous to the putative Wnt-binding site of Frizzled proteins.

tumorgenesis

SFRP1 gene was frequently downregulated by promoter hypermethylation and suppresses tumor growth activity of lung cancer cells, which suggests that SFRP1 is a candidate tumor suppressor gene for lung cancer [124]. Methylation of SFRP1 was reversely correlated with EGFR mutation which progress NSCLC [125]. SFRP1 also modulated taxane resistance of human lung AdC, sub type of NSCLC [126]. Zhang et al [127] found that SFRP1 exhibits a significantly higher frequency of methylation in NSCLC compared with the normal tissues. Promoter hypermethylation of SFRP1 [128] was found in 32.1% NSCLC specimens and was closely correlated with loss of expression, besides SFRP1 hypermethylation was associated with lymph metastasis and disease progression within one year. SFRP1 was dramatically downregulated in transforming growth factor $\beta 1$ (TGF-$\beta 1$)-induced Epithelial-mesenchymal transition (EMT) in the A549 human lung cancer cell line. Restoration of SFRP1 could inhibit the TGF-$\beta 1$-induced EMT phenotype and tumor metastasis of the A549 cell line both in vitro and in vivo through inhibition of the Wnt pathway [129]. SFRP1 was reported to bind to PPP1CA [130] whose downregulation in NSCLC was reported to contribute to tumourigenesis [131], to bind to WNT2 [132] that activates Wnt signaling [133] and inhibition of Wnt2-mediated signaling induces programmed cell death[134] in NSCLC, to bind to WNT1 [132, 135] that was an independent poor prognostic marker of NSCLC after surgery [136] and whose overexpression promotes tumour progression [137] and is associated with tumor proliferation and a poor prognosis [138] in NSCLC. SFRP1 was also reported to bind to FZD6 [132] that was a key protein of Wnt signalin pathway. By competeing with Wnt for binding to FZD, SFRP1 can suppress the acitivation of Wnt signaling pathway. All of these suggests that SFRP1 directly or indirectly contributes to progression of NSCLC.

Although it is not the study about NSCLC, Silva et al recetntly invesitigated how hypermethylation of the Wnt antagonists including SFRP1 affects the progress of colorectal

cancer. Then they have found that hypermethylation of the Wnt antagonists progressed from normal to tumor and further to metastasis. They also conformed that hypermethylation of the Wnt antagonists are correlated with loss of expression of these genes and an increase in nuclear Wnt pathway activity [139]. This suggests that hypermethylation of the Wnt antagonists was critical driver of colorectal cancer. Thus, it is not surprising if loss of hypermethylation of SFRP1 caused by reprogramming and accompanied with induction of gene expression can be used for theraphy of NSCLC.

pluripotency

Kwon et al [140] found that SFRP1, a counter-acting molecule of Wnt, was more suppressed in protein-based iPS cells than in mouse embryonic stem cells, while Wnt signaling was upregulated. SFRP1 turned out to be direct transcriptional target of TFAP2C, whose deficient primordial germ cell-like cells display cancer related deregulations in epigenetic remodeling, cell cycle and pluripotency control [141].

SLC16A12

tumorgenesis

Although nothing was reported about the association with NSCLC for SLC16A12, since its methylation was reported to be promising biomarker of prostate, colon and breast cancer [142], it is not surprising if methylation of SLC16A12 is also associated with NSCLC. More studies are waited.

pluripotency

None.

HOXA5

HOXA5 is one of homeobox genes that are supposed to contribute to embryonic development.

tumorgenesis

Kim et al [143] found that downregulation of the HOXA5 gene by aberrant promoter methylation occurs in the vast majority of NSCLCs and that it may play a role in the pathogenesis of NSCLC. Abe et al [144] suggested that the disordered patterns of HOX gene expressions were involved not only in the development of NSCLC but also in the histologically aberrant diversity such as AdC and SCC. Wang et al [145] found that NSCLC tissue expressed miRNA-130a that plays a role in antagonizing the inhibitory effects of HoxA5. MicroRNA-196a promotes NSCLC cell proliferation and invasion through targeting HOXA5 [146]. The effect of miR-196a on proliferation, colony formation assays, cell migration and invasion were evaluated. Liu et al [147] indicates that HOTAIR is significantly up-regulated in NSCLC tissues, and regulates NSCLC cell invasion and metastasis, partially via the down-regulation of HOXA5.

pluripotency

HoxA5 genes were specifically expressed in white adipocyte progenitors during differentiation of human induced pluripotent stem cells[148].

KIF1A

The protein encoded by this gene is a member of the kinesin family and functions as an anterograde motor protein that transports membranous organelles along axonal microtubules.

tumorgenesis

The promoter of KIF1A was found to be methylated associated with lung cancer [149]. Methylation of KIF1A in sputum had also significant odds ratio between lung cancer and control [150].

pluripotency

None.

H2AFY

This gene encodes a member of the histone H2A family. It replaces conventional H2A histones in a subset of nucleosomes where it represses transcription and participates in stable X chromosome inactivation.

tumorgenesis

H2AFY isoforms predict the risk of lung cancer recurrence [151].

pluripotency

MacroH2A histone variants act as a barrier upon reprogramming towards pluripotency [152].

ATP5G2

This gene encodes a subunit of mitochondrial ATP synthase.

tumorgenesis

Although there were no reports related to lung cancer, ATP5G2 showed frequent promoter region methylation in primary renal cell carcinoma tumour samples [153]. ATP5G2 was also reported to be expressive in estrogen and progesterone treated human endometrial Ishikawa cancer cell line [154].

pluripotency

None.

TM4SF1

The protein encoded by this gene is a member of the transmembrane 4 superfamily, also known as the tetraspanin family. Most of these members are cell-surface proteins that are characterized by the presence of four hydrophobic domains.

tumorgenesis

TM4SF1, also known as L6, promoted the invasiveness of lung cancer cells and was inversely correlated with disease-free survival of squamous lung carcinoma patients [155]. TM4SF1 was also reported to be upregulated in cancer-stem-cell in human lung AdC A549 cells [156]. TM4SF4 overexpression in radiation-resistant lung carcinoma cells activates IGF1R via elevation of IGF1 [157].

pluripotency
Combined omics analysis identifies TM4SF1 as a surface protein marker specific to human mesenchymal stem cells [158].

S100P

S100 proteins are localized in the cytoplasm and/or nucleus of a wide range of cells, and involved in the regulation of a number of cellular processes such as cell cycle progression and differentiation.

tumorgenesis
SIX3 was down-regulated in lung AdC tissues compared to their matched adjacent normal tissues, and restoration of SIX3 in lung cancer cells lacking endogenous SIX3 downregulated S100P [159]. S100P was reported to be disregulated in lung cancer [160]. The upregulation of either S100A2 or S100P was detected in early but less in advanced tumour stages of NSCLC [161].

pluripotency
S100P was detected in the original paper that studied this experiment [162].

SPINT2

This gene encodes a transmembrane protein with two extracellular Kunitz domains that inhibits a variety of serine proteases. There are numerous reports that suggest the relationship between SPINT2 and cancers, although not so many were reported on the relationship with NSCLC.

tumorgenesis
SPINT2 was reported to be downregulated in A549 NSCLC cell line [163].

pluripotency
SPINT2 was reported to be related to hematopoietic stem cells [164].

CDH1

This gene is a classical cadherin from the cadherin superfamily. The encoded protein is a calcium dependent cell-cell adhesion glycoprotein comprised of five extracellular cadherin repeats, a transmembrane region and a highly conserved cytoplasmic tail. Many were reported to suggest the relationship between CDH1 and cancers including NSCLC.

tumorgenesis
Bisulphite sequencing showed that bovine parthenogenetic oocyte extract induced significant demethylation at the promoters of the tumour suppressor genes RUNX3 and CDH1 [165]. Loss of CDH1 up-regulates epidermal growth factor receptor via phosphorylation of YBX1 in NSCLC cells [166]. WT1 promotes invasion of NSCLC via suppression of CDH1 [167]. CDH1, promoter methylation was less frequent in adenosquamous carcinomas than AdCs [168]. CDH1 was detected using the theory of coevolution to predict protein-protein interactions in NSCLC [169].

pluripotency
CDH1 expression was reactivated in Egg White treated MDA-MB-231 cells [170]. CDH1 expression was positively correlated with the pluripotency genes expression induced by CTGF [171].

LAMC2

Laminins, a family of extracellular matrix glycoproteins, are the major noncollagenous constituent of basement membranes. They have been implicated in a wide variety of biological processes including cell adhesion, differentiation, migration, signaling, neurite outgrowth and metastasis.

tumorgenesis
Sathyanarayana et al [172] investigated epigenetic inactivation of lamin 5 encoding genes, one of which is LAMC2, in NSCLC cell lines. They have found the frequent loss of LAMC2 expression and found that the promoter methylation mediated this loss. Manda et al observed differential expression of the LAMC2 between small cell and non-small cell lung carcinomas [173]. LCMA2 plasma level in NSCLC was significantly lower than controls [174].

pluripotency
During *in vitro* differentiation of hESCs/iPSCs into retinal pigment epithelial cells, stage-specific DNA methylation patterns of LAMC2 was observed [175].

HMGA1

This gene encodes a non-histone protein involved in many cellular processes, including regulation of inducible gene transcription, integration of retroviruses into chromosomes, and the metastatic progression of cancer cells.

tumorgenesis
Overexpression of HMGA1 in blood was proposed to be suitable for diagnosis of lung AdC and SCC, sub-types of NSCLC [176]. RNA profiling of lung epithelial cells (BEAS-2B) expressing a mutant allele of PIK3 (E545K) identified a network of transcription factors such as MYC, FOS and HMGA1 [177]. In a cohort of NSCLC tumors, HMGA1 overexpression was immediately associated with enhanced expression of an oncogenic miRNA, namely, miR-222 [178]. Upregulation of MMP2 by HMGA1 promotes transformation in undifferentiated, large-cell lung cancer [179]. HMGA2 participated in transformation in human lung cancer [180]. Increased expression of HMGA1 proteins was observed in lung cancer [181]. HMGA1 was upregulated in squamous cell lung cancer, a subtype of NSCLC [182].

pluripotency
Transient E2F2 silencing in hESC significantly inhibited expression of the proto-oncogenes HMGA1 [183]. HMGA1 reprograms somatic cells into pluripotent stem cells by inducing stem cell transcriptional networks [184].

LAD1

The protein encoded by this gene may be an anchoring filament that is a component of basement membranes. It may contribute to the stability of the association of the epithelial layers with the underlying mesenchyme.

tumorgenesis

Although there were no reports that studied the direct relationship between NSCLC and LAD1 excluding a report that suggested that it may be associated with tumor cell differentiation in NSCLC [185], it was reported to form protein complex [82] with APP whose expression was altered associated with alternative splicing in NSCLC [83]. LAD1 was also reported to form protein complex [186] with SFN, also known as 14-3-3 Sigma, whose expression was distinct between metastasis-negative and -positive lymph nodes in NSCLC [187] and increased associated with promoter hypomethylayion in NSCLC [188].

pluripotency

None.

PFKFB3

PFKFB3 (6-Phosphofructo-2-Kinase /Fructose-2, 6- Biphosphatase 3) is a Protein Coding gene. Among its related pathways are Akt Signaling and Metabolism. GO annotations related to this gene include fructose-2,6- bisphosphate 2- phosphatase activity and 6- phosphofructo-2-kinase activity.

tumorgenesis

mRNA and protein over expression of PFKFB3 was observed in human lung cancers [189]. Although we could not find any other studies that report the relationship between PFKFB3 and NSCLC, PFKFB3 was well known to contribute to tumor genesis [190].

pluripotency

None.

DEFB1

This gene encodes defensin, beta 1, an antimicrobial peptide implicated in the resistance of epithelial surfaces to microbial colonization.

tumorgenesis

DEFB1, also known as HBD1, was higher in patients with lung cancer than healthy subjects [191].

pluripotency

None.

SRGN

This gene encodes a protein best known as a hematopoietic cell granule proteoglycan. Proteoglycans stored in the secretory granules of many hematopoietic cells also contain a protease-resistant peptide core, which may be important for neutralizing hydrolytic enzymes

tumorgenesis

SRGN was reported to be differently expressed inn human lung AdCs and aquamous cell carcinomas [192]. Although there were no studies that report the direct relationship between SRGN and NSCLC, SRGN was generally regareded to be related to cancers [193]. Thus it is not surprising even if there appears additional reports about the direct relationship between SRGN and NSCLC.

pluripotency

Serglycin proteoglycan was expressed in embryonic stem cells [194]. Serglycin is synthesized by endothelial cells [195].

UCHL1

The protein encoded by this gene belongs to the peptidase C12 family.

tumorgenesis

UCHL1, also known as PGP9.5, was upregulated in the non-metastatic CL1-0 than in highly metastatic CL1-5 cell lines [196]. UCHL1 was reported to be methylated in NSCLC [197]. In non-small cell lung carcinoma primary tumour samples, UCHL1 was highly expressed and is associated with an advanced tumour stage [198]. Proteomics-based Identification of PGP 9.5 as a Tumor Antigen That Induces a Humoral Immune Response in Lung Cancer [199].

pluripotency

None.

ALDH3A1

Aldehyde dehydrogenases oxidize various aldehydes to the corresponding acids. They are involved in the detoxification of alcohol-derived acetaldehyde and in the metabolism of corticosteroids, biogenic amines, neurotransmitters, and lipid peroxidation.

tumorgenesis

ALDH3A1 was over expressed in NSCLC [200]. ALDH3A1 was identified as potential diagnostic markers in NSCLC [201]. ALDH3A1 was upregulated in lung cancer [202].

pluripotency

None.

EPB41L3

EPB41L3 is known as both protein coding gene and lncRNA, but its function is not well understood.

tumorgenesis

Loss of expression of the differentially expressed in AdC of the lung EPB41L3, also known as DAL-1, protein is associated with metastasis of non-small cell lung carcinoma cells [203]. FRMD3 that is paralog of EPB41L3 was known as a novel putative tumour suppressor in NSCLC [204]. Loss of DAL-1 expression was seen in 14 of 16 (87%) NSCLC cell lines and DAL-1 methylation was observed in 17 of 39 (44%) NSCLC cell lines, in tumors of NSCLC patients with

stage II-III disease, DAL-1 methylation was seen at a statistically significant higher frequency compared to tumors of patients with stage I disease and overall, 65% of primary NSCLCs had either TSLC1 or DAL-1 methylated [205]. Promoter methylation of DAL-1/4.1B predicts poor prognosis in NSCLC [206]. In lung tumor cells, expression of NSP-A and most likely also NSP-C is restricted to cells with a neuroendocrine phenotype [207].

pluripotency

None.

RTN1

This gene belongs to the family of reticulon-encoding genes. Reticulons are associated with the endoplasmic reticulum, and are involved in neuroendocrine secretion or in membrane trafficking in neuroendocrine cells.

tumorgenesis

Aberrant expression of RTN1 was identified among comparison between NSCLC, matched normal bronchial epithelium, and peripheral lung tissue from both smokers and non-smokers [208]. Expression of RTN1, also knows as NSP-reticulon, is restricted to lung carcinoma cells with a neuroendocrine (NE) phenotype [209]. that has also been found to be a feature of a proportion of non-small cell lung carcinomas [210]. On the other hand, RTN1 was known to bind to BCL2L1, also known as Bcl-X [211], to which RBM4 that inhibited NSCLC cell growth [212] and BAG3 that promotes resistance to apoptosis through Bcl-2 family members in NSCLC [213] binds, and over-expression of BCL2L1 was frequently found in NSCLC where it potentially contributes to tumor development [214].

pluripotency

None.

LAMA1

LAMA1 is known as protein coding gene, but its function is not well understood.

tumorgenesis

The mutated genes in the most significant extracellular matrix remodeling gene set in NSCLC include LAMA1[215]. LAMA1 is also a part of KEGG pathway hsa05222 "Small cell lung cancer". A genome-wide association study reveals susceptibility variants for NSCLC in the Korean population [216].

pluripotency

Although there were no reports that suggest direct relationship between pluripotency and LAMA1, there were several reports that suggest the relationship between development and LAMA1. Mutations in LAMA1 disrupt retinal vascular development and inner limiting membrane formation [217]. LAMA1 mutations lead to vitreoretinal blood vessel formation, persistence of fetal vasculature, and epiretinal membrane formation in mice [218]. LAMA1 is essential for mouse cerebellar development[219].

GPR56

The gene product is a member of the adhesion-GPCR family of receptors.

tumorgenesis

GPR56 was generally known as tumor suppressor [220]. However, it was also known to work as oncogene and to be regarded as potential therapy target. Ke et al [221] confirmed GPR56 overexpression *in vivo* in NSCLC. In addition to this, they observed that reduction of GPR56 expression reduced soft agar growth in NCI-H460 cell line and that GPR56 silencing reduced cell adhesion in A2058 cells that is melanoma cell line. Guo et al [100] also previously reported aberrant methylation of GPR56 in A549 cell line, which is related to drug resistance. Thus, GPR56 can be a promising therapy target of NSCLC. However, GPR56 is multifunctional protein. It was reported to act as tumor suppressor for melanoma. In this regard, GPR56 tried to reduce progression of melanoma by suppressing production of VEGF that is a major stimulator for angiogenesis, which is a critical step during cancer progression [222]. On the other hand, when GPR56 played critical roles in cortical development, GPR56 couples to the Gα(12/13) family of G proteins and activates RhoA pathway upon ligand (COL3A1) binding [223]. Thus, it is likely that GPR56 will obey completely different mechanisms from those, when GPR56 act as onocogene for NSCLC. At the moment, the mechanisms were not known at all. More experiment-oriented researches are waited [224].

pluripotency

None

References

[1] J. Xiong. SALL4: Engine of Cell Stemness. *Curr Gene Ther*, Aug 2014.

[2] E. Rodriguez, L. Chen, M. H. Ao, S. Geddes, E. Gabrielson, F. Askin, H. Zhang, and Q. K. Li. Expression of transcript factors SALL4 and OCT4 in a subset of non-small cell lung carcinomas (NSCLC). *Transl Respir Med*, 2(1):10, Dec 2014.

[3] D. Kobayashi, K. Kuribayashi, M. Tanaka, and N. Watanabe. Overexpression of SALL4 in lung cancer and its importance in cell proliferation. *Oncol. Rep.*, 26(4):965–970, Oct 2011.

[4] F. Yang, Y. Yao, Y. Jiang, L. Lu, Y. Ma, and W. Dai. Sumoylation is important for stability, subcellular localization, and transcriptional activity of SALL4, an essential stem cell transcription factor. *J. Biol. Chem.*, 287(46):38600–38608, Nov 2012.

[5] J. Y. Han, G. K. Lee, S. Y. Yoo, S. J. Yoon, E. Y. Cho, H. T. Kim, and J. S. Lee. Association of SUMO1 and UBC9 genotypes with tumor response in non-small-cell lung cancer treated with irinotecan-based chemotherapy. *Pharmacogenomics J.*, 10(2):86–93, Apr 2010.

[6] K. Abdelmohsen, S. Srikantan, X. Yang, A. Lal, H. H. Kim, Y. Kuwano, S. Galban, K. G. Becker, D. Kamara, R. de Cabo, and M. Gorospe. Ubiquitin-mediated proteolysis of HuR by heat shock. *EMBO J.*, 28(9):1271–1282, May 2009.

[7] J. Wang, W. Zhao, Y. Guo, B. Zhang, Q. Xie, D. Xiang, J. Gao, B. Wang, and Z. Chen. The expression of RNA-binding protein HuR in non-small cell lung cancer correlates with vascular endothelial growth factor-C expression and lymph node metastasis. *Oncology*, 76(6):420–429, 2009.

[8] J. Yang, L. Chai, T. C. Fowles, Z. Alipio, D. Xu, L. M. Fink, D. C. Ward, and Y. Ma. Genome-wide analysis reveals Sall4 to be a major regulator of pluripotency in murine-embryonic stem cells. *Proc. Natl. Acad. Sci. U.S.A.*, 105(50):19756–19761, Dec 2008.

[9] S. H. Chiou, M. L. Wang, Y. T. Chou, C. J. Chen, C. F. Hong, W. J. Hsieh, H. T. Chang, Y. S. Chen, T. W. Lin, H. S. Hsu, and C. W. Wu. Coexpression of Oct4 and Nanog enhances malignancy in lung adenocarcinoma by inducing cancer stem cell-like properties and epithelial-mesenchymal transdifferentiation. *Cancer Res.*, 70(24):10433–10444, Dec 2010.

[10] Y. Buganim, S. Markoulaki, N. van Wietmarschen, H. Hoke, T. Wu, K. Ganz, B. Akhtar-Zaidi, Y. He, B. J. Abraham, D. Porubsky, E. Kulenkampff, D. A. Faddah, L. Shi, Q. Gao, S. Sarkar, M. Cohen, J. Goldmann, J. R. Nery, M. D. Schultz, J. R. Ecker, A. Xiao, R. A. Young, P. M. Lansdorp, and R. Jaenisch. The developmental potential of iPSCs is greatly influenced by reprogramming factor selection. *Cell Stem Cell*, 15(3):295–309, Sep 2014.

[11] J. Yang, C. Gao, L. Chai, and Y. Ma. A novel SALL4/OCT4 transcriptional feedback network for pluripotency of embryonic stem cells. *PLoS ONE*, 5(5):e10766, 2010.

[12] A. Ni, M. J. Wu, and S. H. Chavala. Sphere Formation Permits Oct4 Reprogramming of Ciliary Body Epithelial Cells into Induced Pluripotent Stem Cells. *Stem Cells Dev.*, Aug 2014.

[13] P. A. Baeuerle and O. Gires. EpCAM (CD326) finding its role in cancer. *Br. J. Cancer*, 96(3):417–423, Feb 2007.

[14] R. Cubas, M. Li, C. Chen, and Q. Yao. Trop2: a possible therapeutic target for late stage epithelial carcinomas. *Biochim. Biophys. Acta*, 1796(2):309–314, Dec 2009.

[15] M. G. Pak, D. H. Shin, C. H. Lee, and M. K. Lee. Significance of EpCAM and TROP2 expression in non-small cell lung cancer. *World J Surg Oncol*, 10:53, 2012.

[16] C. Eberlein, C. Rooney, S. J. Ross, M. Farren, H. M. Weir, and S. T. Barry. E-Cadherin and EpCAM expression by NSCLC tumour cells associate with normal fibroblast activation through a pathway initiated by integrin $\alpha v\beta 6$ and maintained through TGFβ signalling. *Oncogene*, Feb 2014.

[17] Z. J. Liao, Y. H. Guo, Z. Zhao, J. T. Yao, R. Xu, and K. J. Nan. Gemcitabine inhibits the micrometastasis of non-small cell lung cancer by targeting the EpCAM-positive circulating tumor cells via the HGF/cMET pathway. *Int. J. Oncol.*, 45(2):651–658, Aug 2014.

[18] J. Nicholson, A. Scherl, L. Way, E. A. Blackburn, M. D. Walkinshaw, K. L. Ball, and T. R. Hupp. A systems wide mass spectrometric based linear motif screen to identify dominant in-vivo interacting proteins for the ubiquitin ligase MDM2. *Cell. Signal.*, 26(6):1243–1257, Jun 2014.

[19] Q. Kong, P. Li, Q. Tian, and M. W. Ha. Role of MDM2 T309G polymorphism in susceptibility and prognosis of nonsmall cell lung cancer: a meta-analysis. *Genet Test Mol Biomarkers*, 18(5):357–365, May 2014.

[20] A. Fukatsu, F. Ishiguro, I. Tanaka, T. Kudo, K. Nakagawa, K. Shinjo, Y. Kondo, M. Fujii, Y. Hasegawa, K. Tomizawa, T. Mitsudomi, H. Osada, Y. Hata, and Y. Sekido. RASSF3 downregulation increases malignant phenotypes of non-small cell lung cancer. *Lung Cancer*, 83(1):23–29, Jan 2014.

[21] K. H. Lu, W. Li, X. H. Liu, M. Sun, M. L. Zhang, W. Q. Wu, W. P. Xie, and Y. Y. Hou. Long non-coding RNA MEG3 inhibits NSCLC cells proliferation and induces apoptosis by affecting p53 expression. *BMC Cancer*, 13:461, 2013.

[22] D. W. Wu, M. C. Lee, J. Wang, C. Y. Chen, Y. W. Cheng, and H. Lee. DDX3 loss by p53 inactivation promotes tumor malignancy via the MDM2/Slug/E-cadherin pathway and poor patient outcome in non-small-cell lung cancer. *Oncogene*, 33(12):1515–1526, Mar 2014.

[23] H. Yu, Y. Zou, L. Jiang, Q. Yin, X. He, L. Chen, Z. Zhang, W. Gu, and Y. Li. Induction of apoptosis in non-small cell lung cancer by downregulation of MDM2 using pH-responsive PMPC-b-PDPA/siRNA complex nanoparticles. *Biomaterials*, 34(11):2738–2747, Apr 2013.

[24] Y. A. Tang, R. K. Lin, Y. T. Tsai, H. S. Hsu, Y. C. Yang, C. Y. Chen, and Y. C. Wang. MDM2 overexpression deregulates the transcriptional control of RB/E2F leading to DNA methyltransferase 3A overexpression

in lung cancer. *Clin. Cancer Res.*, 18(16):4325–4333, Aug 2012.

[25] X. Wang, J. Venable, P. LaPointe, D. M. Hutt, A. V. Koulov, J. Coppinger, C. Gurkan, W. Kellner, J. Matteson, H. Plutner, J. R. Riordan, J. W. Kelly, J. R. Yates, and W. E. Balch. Hsp90 cochaperone Aha1 downregulation rescues misfolding of CFTR in cystic fibrosis. *Cell*, 127(4):803–815, Nov 2006.

[26] J. W. Son, Y. J. Kim, H. M. Cho, S. Y. Lee, S. M. Lee, J. K. Kang, J. U. Lee, Y. M. Lee, S. J. Kwon, E. Choi, M. J. Na, J. Y. Park, and D. S. Kim. Promoter hypermethylation of the CFTR gene and clinical/pathological features associated with non-small cell lung cancer. *Respirology*, 16(8):1203–1209, Nov 2011.

[27] C. D. Coldren, B. A. Helfrich, S. E. Witta, M. Sugita, R. Lapadat, C. Zeng, A. Baron, W. A. Franklin, F. R. Hirsch, M. W. Geraci, and P. A. Bunn. Baseline gene expression predicts sensitivity to gefitinib in non-small cell lung cancer cell lines. *Mol. Cancer Res.*, 4(8):521–528, Aug 2006.

[28] S. Benlloch, J. M. Galbis-Caravajal, C. Alenda, F. M. Peiro, M. Sanchez-Ronco, J. M. Rodriguez-Paniagua, B. Baschwitz, E. Rojas, and B. Massuti. Expression of molecular markers in mediastinal nodes from resected stage I non-small-cell lung cancer (NSCLC): prognostic impact and potential role as markers of occult micrometastases. *Ann. Oncol.*, 20(1):91–97, Jan 2009.

[29] Y. Y. Lin, S. Kiihl, Y. Suhail, S. Y. Liu, Y. H. Chou, Z. Kuang, J. Y. Lu, C. N. Khor, C. L. Lin, J. S. Bader, R. Irizarry, and J. D. Boeke. Functional dissection of lysine deacetylases reveals that HDAC1 and p300 regulate AMPK. *Nature*, 482(7384):251–255, Feb 2012.

[30] H. Li, Z. Feng, W. Wu, J. Li, J. Zhang, and T. Xia. SIRT3 regulates cell proliferation and apoptosis related to energy metabolism in non-small cell lung cancer cells through deacetylation of NMNAT2. *Int. J. Oncol.*, 43(5):1420–1430, Nov 2013.

[31] H. P. Huang, P. H. Chen, C. Y. Yu, C. Y. Chuang, L. Stone, W. C. Hsiao, C. L. Li, S. C. Tsai, K. Y. Chen, H. F. Chen, H. N. Ho, and H. C. Kuo. Epithelial cell adhesion molecule (EpCAM) complex proteins promote transcription factor-mediated pluripotency reprogramming. *J. Biol. Chem.*, 286(38):33520–33532, Sep 2011.

[32] M. Takahama, M. Tsutsumi, T. Tsujiuchi, K. Nezu, K. Kushibe, S. Taniguchi, Y. Kotake, and Y. Konishi. Enhanced expression of Tie2, its ligand angiopoietin-1, vascular endothelial growth factor, and CD31 in human non-small cell lung carcinomas. *Clin. Cancer Res.*, 5(9):2506–2510, Sep 1999.

[33] W. Naumnik, B. Naumnik, K. Niewiarowska, M. Ossolinska, and E. Chyczewska. Angiogenic axis angiopoietin-1 and angiopoietin-2/Tie-2 in non-small cell lung cancer: a bronchoalveolar lavage and serum study. *Adv. Exp. Med. Biol.*, 788:341–348, 2013.

[34] M. P. Wong, S. Y. Chan, K. H. Fu, S. Y. Leung, N. Cheung, S. T. Yuen, and L. P. Chung. The angiopoietins, tie2 and vascular endothelial growth factor are differentially expressed in the transformation of normal lung to non-small cell lung carcinomas. *Lung Cancer*, 29(1):11–22, Jul 2000.

[35] U. Fiedler, T. Krissl, S. Koidl, C. Weiss, T. Koblizek, U. Deutsch, G. Martiny-Baron, D. Marme, and H. G. Augustin. Angiopoietin-1 and angiopoietin-2 share the same binding domains in the Tie-2 receptor involving the first Ig-like loop and the epidermal growth factor-like repeats. *J. Biol. Chem.*, 278(3):1721–1727, Jan 2003.

[36] S. N. Metodieva, D. N. Nikolova, R. V. Cherneva, I. I. Dimova, D. B. Petrov, and D. I. Toncheva. Expression analysis of angiogenesis-related genes in Bulgarian patients with early-stage non-small cell lung cancer. *Tumori*, 97(1):86–94, 2011.

[37] Y. Zhang, F. Guessous, A. Kofman, D. Schiff, and R. Abounader. XL-184, a MET, VEGFR-2 and RET kinase inhibitor for the treatment of thyroid cancer, glioblastoma multiforme and NSCLC. *IDrugs*, 13(2):112–121, Feb 2010.

[38] H. J. Joo, H. Kim, S. W. Park, H. J. Cho, H. S. Kim, D. S. Lim, H. M. Chung, I. Kim, Y. M. Han, and G. Y. Koh. Angiopoietin-1 promotes endothelial differentiation from embryonic stem cells and induced pluripotent stem cells. *Blood*, 118(8):2094–2104, Aug 2011.

[39] N. Orii and M. K. Ganapathiraju. Wiki-pi: a web-server of annotated human protein-protein interactions to aid in discovery of protein function. *PLoS ONE*, 7(11):e49029, 2012.

[40] Q. Pang, Q. Wei, T. Xu, X. Yuan, J. L. Lopez Guerra, L. B. Levy, Z. Liu, D. R. Gomez, Y. Zhuang, L. E. Wang, R. Mohan, R. Komaki, and Z. Liao. Functional promoter variant rs2868371 of HSPB1 is associated with risk of radiation pneumonitis after chemoradiation for non-small cell lung cancer. *Int. J. Radiat. Oncol. Biol. Phys.*, 85(5):1332–1339, Apr 2013.

[41] T. Xu, Q. Wei, J. L. Lopez Guerra, L. E. Wang, Z. Liu, D. Gomez, M. O'Reilly, S. H. Lin, Y. Zhuang, L. B. Levy, R. Mohan, H. Zhou, and Z. Liao. HSPB1 gene polymorphisms predict risk of mortality for US patients after radio(chemo)therapy for non-small cell lung cancer. *Int. J. Radiat. Oncol. Biol. Phys.*, 84(2):e229–235, Oct 2012.

[42] J. L. Lopez Guerra, Q. Wei, X. Yuan, D. Gomez, Z. Liu, Y. Zhuang, M. Yin, M. Li, L. E. Wang, J. D. Cox, and Z. Liao. Functional promoter rs2868371 variant of HSPB1 associates with radiation-induced esophageal toxicity in patients with non-small-cell lung cancer

treated with radio(chemo)therapy. *Radiother Oncol*, 101(2):271–277, Nov 2011.

[43] G. Y. Zhao, J. Y. Ding, C. L. Lu, Z. W. Lin, and J. Guo. The overexpression of 14-3-3*zeta* and Hsp27 promotes non-small cell lung cancer progression. *Cancer*, 120(5):652–663, Mar 2014.

[44] M. Zimmermann, S. Nickl, C. Lambers, S. Hacker, A. Mitterbauer, K. Hoetzenecker, A. Rozsas, G. Ostoros, V. Laszlo, H. Hofbauer, F. Renyi-Vamos, W. Klepetko, B. Dome, and H. J. Ankersmit. Discrimination of clinical stages in non-small cell lung cancer patients by serum HSP27 and HSP70: a multi-institutional case-control study. *Clin. Chim. Acta*, 413(13-14):1115–1120, Jul 2012.

[45] M. Zimmermann, T. Mueller, B. Dieplinger, C. Bekos, L. Beer, H. Hofbauer, B. Dome, and H. J. Ankersmit. Circulating heat shock protein 27 as a biomarker for the differentiation of patients with lung cancer and healthy controls–a clinical comparison of different enzyme linked immunosorbent assays. *Clin. Lab.*, 60(6):999–1006, 2014.

[46] H. Guo, Y. Bai, P. Xu, Z. Hu, L. Liu, F. Wang, G. Jin, F. Wang, Q. Deng, Y. Tu, M. Feng, D. Lu, H. Shen, and T. Wu. Functional promoter -1271G¿C variant of HSPB1 predicts lung cancer risk and survival. *J. Clin. Oncol.*, 28(11):1928–1935, Apr 2010.

[47] E. Malusecka, S. Krzyzowska-Gruca, J. Gawrychowski, A. Fiszer-Kierzkowska, Z. Kolosza, and Z. Krawczyk. Stress proteins HSP27 and HSP70i predict survival in non-small cell lung carcinoma. *Anticancer Res.*, 28(1B):501–506, 2008.

[48] T. Shimamura, Z. Chen, M. Soucheray, J. Carretero, E. Kikuchi, J. H. Tchaicha, Y. Gao, K. A. Cheng, T. J. Cohoon, J. Qi, E. Akbay, A. C. Kimmelman, A. L. Kung, J. E. Bradner, and K. K. Wong. Efficacy of BET bromodomain inhibition in Kras-mutant non-small cell lung cancer. *Clin. Cancer Res.*, 19(22):6183–6192, Nov 2013.

[49] P. Zhao, M. S. Damerow, P. Stern, A. H. Liu, A. Sweet-Cordero, K. Siziopikou, J. R. Neilson, P. A. Sharp, and C. Cheng. CD44 promotes Kras-dependent lung adenocarcinoma. *Oncogene*, 32(43):5186–5190, Oct 2013.

[50] S. Licciulli, J. L. Avila, L. Hanlon, S. Troutman, M. Cesaroni, S. Kota, B. Keith, M. C. Simon, E. Pure, F. Radtke, A. J. Capobianco, and J. L. Kissil. Notch1 is required for Kras-induced lung adenocarcinoma and controls tumor cell survival via p53. *Cancer Res.*, 73(19):5974–5984, Oct 2013.

[51] S. Shen, C. Q. Mao, X. Z. Yang, X. J. Du, Y. Liu, Y. H. Zhu, and J. Wang. Cationic lipid-assisted polymeric nanoparticle mediated GATA2 siRNA delivery for synthetic lethal therapy of KRAS mutant non-small-cell lung carcinoma. *Mol. Pharm.*, 11(8):2612–2622, Aug 2014.

[52] N. Zhao, M. D. Wilkerson, U. Shah, X. Yin, A. Wang, M. C. Hayward, P. Roberts, C. B. Lee, A. M. Parsons, L. B. Thorne, B. E. Haithcock, J. E. Grilley-Olson, T. E. Stinchcombe, W. K. Funkhouser, K. K. Wong, N. E. Sharpless, and D. N. Hayes. Alterations of LKB1 and KRAS and risk of brain metastasis: Comprehensive characterization by mutation analysis, copy number, and gene expression in non-small-cell lung carcinoma. *Lung Cancer*, 86(2):255–261, Nov 2014.

[53] A. Yilmaz, N. Mohamed, K. A. Patterson, Y. Tang, K. Shilo, M. A. Villalona-Calero, M. E. Davis, X. P. Zhou, W. Frankel, G. A. Otterson, and W. Zhao. Clinical and metabolic parameters in non-small cell lung carcinoma and colorectal cancer patients with and without KRAS mutations. *Int J Environ Res Public Health*, 11(9):8645–8660, Sep 2014.

[54] A. Yilmaz, N. Mohamed, K. A. Patterson, Y. Tang, K. Shilo, M. A. Villalona-Calero, M. E. Davis, X. Zhou, W. Frankel, G. A. Otterson, H. D. Beall, and W. Zhao. Increased NQO1 but not c-MET and survivin expression in non-small cell lung carcinoma with KRAS mutations. *Int J Environ Res Public Health*, 11(9):9491–9502, Sep 2014.

[55] M. K. Maus, P. P. Grimminger, P. C. Mack, S. H. Astrow, C. Stephens, G. Zeger, J. Hsiang, J. Brabender, M. Friedrich, H. Alakus, A. H. Holscher, P. Lara, K. D. Danenberg, H. J. Lenz, and D. R. Gandara. KRAS mutations in non-small-cell lung cancer and colorectal cancer: implications for EGFR-targeted therapies. *Lung Cancer*, 83(2):163–167, Feb 2014.

[56] A. Vinayagam, U. Stelzl, R. Foulle, S. Plassmann, M. Zenkner, J. Timm, H. E. Assmus, M. A. Andrade-Navarro, and E. E. Wanker. A directed protein interaction network for investigating intracellular signal transduction. *Sci Signal*, 4(189):rs8, Sep 2011.

[57] T. Fernandez-Marcelo, A. Moran, C. de Juan, I. Pascua, J. Head, A. Gomez, F. Hernando, J. A. Lopez-Asenjo, S. Hernandez, A. Sanchez-Pernaute, A. J. Torres, M. Benito, and P. Iniesta. Differential expression of senescence and cell death factors in non-small cell lung and colorectal tumors showing telomere attrition. *Oncology*, 82(3):153–164, 2012.

[58] S. Winsel, A. Sommer, J. Eschenbrenner, K. Mittelstaedt, U. Klar, S. Hammer, and J. Hoffmann. Molecular mode of action and role of TP53 in the sensitivity to the novel epothilone sagopilone (ZK-EPO) in A549 non-small cell lung cancer cells. *PLoS ONE*, 6(4):e19273, 2011.

[59] Y. K. Na, S. M. Lee, H. S. Hong, J. B. Kim, J. Y. Park, and D. S. Kim. Hypermethylation of growth arrest DNA-damage-inducible gene 45 in non-small cell

lung cancer and its relationship with clinicopathologic features. *Mol. Cells*, 30(1):89–92, Jul 2010.

[60] A. Catassi, A. Cesario, D. Arzani, P. Menichini, A. Alama, C. Bruzzo, A. Imperatori, N. Rotolo, P. Granone, and P. Russo. Characterization of apoptosis induced by marine natural products in non small cell lung cancer A549 cells. *Cell. Mol. Life Sci.*, 63(19-20):2377–2386, Oct 2006.

[61] A. e. l. S. Mohammed, H. Eguchi, S. Wada, N. Koyama, M. Shimizu, K. Otani, M. Ohtaki, K. Tanimoto, K. Hiyama, M. S. Gaber, and M. Nishiyama. TMEM158 and FBLP1 as novel marker genes of cisplatin sensitivity in non-small cell lung cancer cells. *Exp. Lung Res.*, 38(9-10):463–474, Nov 2012.

[62] U. Stelzl, U. Worm, M. Lalowski, C. Haenig, F. H. Brembeck, H. Goehler, M. Stroedicke, M. Zenkner, A. Schoenherr, S. Koeppen, J. Timm, S. Mintzlaff, C. Abraham, N. Bock, S. Kietzmann, A. Goedde, E. Toksoz, A. Droege, S. Krobitsch, B. Korn, W. Birchmeier, H. Lehrach, and E. E. Wanker. A human protein-protein interaction network: a resource for annotating the proteome. *Cell*, 122(6):957–968, Sep 2005.

[63] C. Kunert-Keil, F. Steinmuller, U. Jeschke, T. Gredes, and T. Gedrange. Immunolocalization of glycodelin in human adenocarcinoma of the lung, squamous cell carcinoma of the lung and lung metastases of colonic adenocarcinoma. *Acta Histochem.*, 113(8):798–802, Dec 2011.

[64] B. Gy?rffy, P. Surowiak, J. Budczies, and A. Lanczky. Online survival analysis software to assess the prognostic value of biomarkers using transcriptomic data in non-small-cell lung cancer. *PLoS ONE*, 8(12):e82241, 2013.

[65] S. Kawabata, J. J. Gills, J. R. Mercado-Matos, J. Lopiccolo, W. Wilson, M. C. Hollander, and P. A. Dennis. Synergistic effects of nelfinavir and bortezomib on proteotoxic death of NSCLC and multiple myeloma cells. *Cell Death Dis*, 3:e353, 2012.

[66] S. Zienolddiny, D. Campa, H. Lind, D. Ryberg, V. Skaug, L. B. Stangeland, F. Canzian, and A. Haugen. A comprehensive analysis of phase I and phase II metabolism gene polymorphisms and risk of non-small cell lung cancer in smokers. *Carcinogenesis*, 29(6):1164–1169, Jun 2008.

[67] A. M. Osman, D. A. van Dartel, E. Zwart, M. Blokland, J. L. Pennings, and A. H. Piersma. Proteome profiling of mouse embryonic stem cells to define markers for cell differentiation and embryotoxicity. *Reprod. Toxicol.*, 30(2):322–332, Sep 2010.

[68] G. Saretzki, L. Armstrong, A. Leake, M. Lako, and T. von Zglinicki. Stress defense in murine embryonic stem cells is superior to that of various differentiated murine cells. *Stem Cells*, 22(6):962–971, 2004.

[69] S. Shankar, D. Nall, S. N. Tang, D. Meeker, J. Passarini, J. Sharma, and R. K. Srivastava. Resveratrol inhibits pancreatic cancer stem cell characteristics in human and KrasG12D transgenic mice by inhibiting pluripotency maintaining factors and epithelial-mesenchymal transition. *PLoS ONE*, 6(1):e16530, 2011.

[70] N. Pashai, H. Hao, A. All, S. Gupta, R. Chaerkady, A. De Los Angeles, J. D. Gearhart, and C. L. Kerr. Genome-wide profiling of pluripotent cells reveals a unique molecular signature of human embryonic germ cells. *PLoS ONE*, 7(6):e39088, 2012.

[71] G. Li, X. D. Ji, H. Gao, J. S. Zhao, J. F. Xu, Z. J. Sun, Y. Z. Deng, S. Shi, Y. X. Feng, Y. Q. Zhu, T. Wang, J. J. Li, and D. Xie. EphB3 suppresses non-small-cell lung cancer metastasis via a PP2A/RACK1/Akt signalling complex. *Nat Commun*, 3:667, 2012.

[72] X. D. Ji, G. Li, Y. X. Feng, J. S. Zhao, J. J. Li, Z. J. Sun, S. Shi, Y. Z. Deng, J. F. Xu, Y. Q. Zhu, H. P. Koeffler, X. J. Tong, and D. Xie. EphB3 is overexpressed in non-small-cell lung cancer and promotes tumor metastasis by enhancing cell survival and migration. *Cancer Res.*, 71(3):1156–1166, Feb 2011.

[73] J. U. Kang, S. H. Koo, K. C. Kwon, J. W. Park, and J. M. Kim. Identification of novel candidate target genes, including EPHB3, MASP1 and SST at 3q26.2-q29 in squamous cell carcinoma of the lung. *BMC Cancer*, 9:237, 2009.

[74] M. Lackmann, R. J. Mann, L. Kravets, F. M. Smith, T. A. Bucci, K. F. Maxwell, G. J. Howlett, J. E. Olsson, T. Vanden Bos, D. P. Cerretti, and A. W. Boyd. Ligand for EPH-related kinase (LERK) 7 is the preferred high affinity ligand for the HEK receptor. *J. Biol. Chem.*, 272(26):16521–16530, Jun 1997.

[75] G. Zhuang, W. Song, K. Amato, Y. Hwang, K. Lee, M. Boothby, F. Ye, Y. Guo, Y. Shyr, L. Lin, D. P. Carbone, D. M. Brantley-Sieders, and J. Chen. Effects of cancer-associated EPHA3 mutations on lung cancer. *J. Natl. Cancer Inst.*, 104(15):1182–1197, Aug 2012.

[76] L. D. Wood, E. S. Calhoun, N. Silliman, J. Ptak, S. Szabo, S. M. Powell, G. J. Riggins, T. L. Wang, H. Yan, A. Gazdar, S. E. Kern, L. Pennacchio, K. W. Kinzler, B. Vogelstein, and V. E. Velculescu. Somatic mutations of GUCY2F, EPHA3, and NTRK3 in human cancers. *Hum. Mutat.*, 27(10):1060–1061, Oct 2006.

[77] E. Becker, U. Huynh-Do, S. Holland, T. Pawson, T. O. Daniel, and E. Y. Skolnik. Nck-interacting Ste20 kinase couples Eph receptors to c-Jun N-terminal kinase and integrin activation. *Mol. Cell. Biol.*, 20(5):1537–1545, Mar 2000.

[78] S. Maki-Nevala, V. Kaur Sarhadi, K. Tuononen, S. Lagstrom, P. Ellonen, M. Ronty, A. Wirtanen, A. Knuuttila, and S. Knuutila. Mutated ephrin receptor genes in non-small cell lung carcinoma and their

occurrence with driver mutations-targeted resequencing study on formalin-fixed, paraffin-embedded tumor material of 81 patients. *Genes Chromosomes Cancer*, 52(12):1141–1149, Dec 2013.

[79] K. Anam and T. A. Davis. Comparative analysis of gene transcripts for cell signaling receptors in bone marrow-derived hematopoietic stem/progenitor cell and mesenchymal stromal cell populations. *Stem Cell Res Ther*, 4(5):112, 2013.

[80] H. Nakanishi, T. Suda, M. Katoh, A. Watanabe, T. Igishi, M. Kodani, S. Matsumoto, M. Nakamoto, Y. Shigeoka, T. Okabe, M. Oshimura, and E. Shimizu. Loss of imprinting of PEG1/MEST in lung cancer cell lines. *Oncol. Rep.*, 12(6):1273–1278, Dec 2004.

[81] M. Kohda, H. Hoshiya, M. Katoh, I. Tanaka, R. Masuda, T. Takemura, M. Fujiwara, and M. Oshimura. Frequent loss of imprinting of IGF2 and MEST in lung adenocarcinoma. *Mol. Carcinog.*, 31(4):184–191, Aug 2001.

[82] J. Olah, O. Vincze, D. Virok, D. Simon, Z. Bozso, N. Tokesi, I. Horvath, E. Hlavanda, J. Kovacs, A. Magyar, M. Sz?cs, F. Orosz, B. Penke, and J. Ovadi. Interactions of pathological hallmark proteins: tubulin polymerization promoting protein/p25, beta-amyloid, and alpha-synuclein. *J. Biol. Chem.*, 286(39):34088–34100, Sep 2011.

[83] C. M. Misquitta-Ali, E. Cheng, D. O'Hanlon, N. Liu, C. J. McGlade, M. S. Tsao, and B. J. Blencowe. Global profiling and molecular characterization of alternative splicing events misregulated in lung cancer. *Mol. Cell. Biol.*, 31(1):138–150, Jan 2011.

[84] R. Mitra, J. Lee, J. Jo, M. Milani, J. N. McClintick, H. J. Edenberg, K. A. Kesler, K. M. Rieger, S. Badve, O. W. Cummings, A. Mohiuddin, D. G. Thomas, X. Luo, B. E. Juliar, L. Li, C. Mesaros, I. A. Blair, A. Srirangam, R. A. Kratzke, C. J. McDonald, J. Kim, and D. A. Potter. Prediction of postoperative recurrence-free survival in non-small cell lung cancer by using an internationally validated gene expression model. *Clin. Cancer Res.*, 17(9):2934–2946, May 2011.

[85] R. M. Ewing, P. Chu, F. Elisma, H. Li, P. Taylor, S. Climie, L. McBroom-Cerajewski, M. D. Robinson, L. O'Connor, M. Li, R. Taylor, M. Dharsee, Y. Ho, A. Heilbut, L. Moore, S. Zhang, O. Ornatsky, Y. V. Bukhman, M. Ethier, Y. Sheng, J. Vasilescu, M. Abu-Farha, J. P. Lambert, H. S. Duewel, I. I. Stewart, B. Kuehl, K. Hogue, K. Colwill, K. Gladwish, B. Muskat, R. Kinach, S. L. Adams, M. F. Moran, G. B. Morin, T. Topaloglou, and D. Figeys. Large-scale mapping of human protein-protein interactions by mass spectrometry. *Mol. Syst. Biol.*, 3:89, 2007.

[86] Q. N. Tran. A novel method for finding non-small cell lung cancer diagnosis biomarkers. *BMC Med Genomics*, 6 Suppl 1:S11, 2013.

[87] M. Tada, T. Tada, L. Lefebvre, S. C. Barton, and M. A. Surani. Embryonic germ cells induce epigenetic reprogramming of somatic nucleus in hybrid cells. *EMBO J.*, 16(21):6510–6520, Nov 1997.

[88] H. Jiang, B. Sun, W. Wang, Z. Zhang, F. Gao, G. Shi, B. Cui, X. Kong, Z. He, X. Ding, Y. Kuang, J. Fei, Y. J. Sun, Y. Feng, and Y. Jin. Activation of paternally expressed imprinted genes in newly derived germline-competent mouse parthenogenetic embryonic stem cell lines. *Cell Res.*, 17(9):792–803, Sep 2007.

[89] A. C. Moss, G. M. Jacobson, L. E. Walker, N. W. Blake, E. Marshall, and J. M. Coulson. SCG3 transcript in peripheral blood is a prognostic biomarker for REST-deficient small cell lung cancer. *Clin. Cancer Res.*, 15(1):274–283, Jan 2009.

[90] K. Valk, T. Vooder, R. Kolde, M. A. Reintam, C. Petzold, J. Vilo, and A. Metspalu. Gene expression profiles of non-small cell lung cancer: survival prediction and new biomarkers. *Oncology*, 79(3-4):283–292, 2010.

[91] M. Hosaka, T. Watanabe, Y. Sakai, Y. Uchiyama, and T. Takeuchi. Identification of a chromogranin A domain that mediates binding to secretogranin III and targeting to secretory granules in pituitary cells and pancreatic beta-cells. *Mol. Biol. Cell*, 13(10):3388–3399, Oct 2002.

[92] Y. Zhao, H. Zhou, K. Ma, J. Sun, X. Feng, J. Geng, J. Gu, W. Wang, H. Zhang, Y. He, S. Guo, X. Zhou, J. Yu, and Q. Lin. Abnormal methylation of seven genes and their associations with clinical characteristics in early stage non-small cell lung cancer. *Oncol Lett*, 5(4):1211–1218, Apr 2013.

[93] B. Nisman, N. Heching, H. Biran, V. Barak, and T. Peretz. The prognostic significance of circulating neuroendocrine markers chromogranin a, pro-gastrin-releasing peptide and neuron-specific enolase in patients with advanced non-small-cell lung cancer. *Tumour Biol.*, 27(1):8–16, 2006.

[94] J. DeGrado-Warren, M. Dufford, J. Chen, P. L. Bartel, D. Shattuck, and G. C. Frech. Construction and characterization of a normalized yeast two-hybrid library derived from a human protein-coding clone collection. *BioTechniques*, 44(2):265–273, Feb 2008.

[95] C. Furukawa, Y. Daigo, N. Ishikawa, T. Kato, T. Ito, E. Tsuchiya, S. Sone, and Y. Nakamura. Plakophilin 3 oncogene as prognostic marker and therapeutic target for lung cancer. *Cancer Res.*, 65(16):7102–7110, Aug 2005.

[96] J. Zhu, H. B. Larman, G. Gao, R. Somwar, Z. Zhang, U. Laserson, A. Ciccia, N. Pavlova, G. Church,

W. Zhang, S. Kesari, and S. J. Elledge. Protein interaction discovery using parallel analysis of translated ORFs (PLATO). *Nat. Biotechnol.*, 31(4):331–334, Apr 2013.

[97] M. Iida, T. M. Brand, D. A. Campbell, C. Li, and D. L. Wheeler. Yes and Lyn play a role in nuclear translocation of the epidermal growth factor receptor. *Oncogene*, 32(6):759–767, Feb 2013.

[98] H. F. J?rgensen, A. Terry, C. Beretta, C. F. Pereira, M. Leleu, Z. F. Chen, C. Kelly, M. Merkenschlager, and A. G. Fisher. REST selectively represses a subset of RE1-containing neuronal genes in mouse embryonic stem cells. *Development*, 136(5):715–721, Mar 2009.

[99] C. W. Huang, H. Y. Chen, M. H. Yen, J. J. Chen, T. H. Young, and J. Y. Cheng. Gene expression of human lung cancer cell line CL1-5 in response to a direct current electric field. *PLoS ONE*, 6(10):e25928, 2011.

[100] R. Guo, G. Wu, H. Li, P. Qian, J. Han, F. Pan, W. Li, J. Li, and F. Ji. Promoter methylation profiles between human lung adenocarcinoma multidrug resistant A549/cisplatin (A549/DDP) cells and its progenitor A549 cells. *Biol. Pharm. Bull.*, 36(8):1310–1316, 2013.

[101] J. M. Martinez, C. A. Afshari, P. R. Bushel, A. Masuda, T. Takahashi, and N. J. Walker. Differential toxicogenomic responses to 2,3,7,8-tetrachlorodibenzo-p-dioxin in malignant and nonmalignant human airway epithelial cells. *Toxicol. Sci.*, 69(2):409–423, Oct 2002.

[102] J. S. Bae, L. Yang, and A. R. Rezaie. Factor X/Xa elicits protective signaling responses in endothelial cells directly via PAR-2 and indirectly via endothelial protein C receptor-dependent recruitment of PAR-1. *J. Biol. Chem.*, 285(45):34803–34812, Nov 2010.

[103] Q. Y. Xu, Y. Gao, Y. Liu, W. Z. Yang, and X. Y. Xu. Identification of differential gene expression profiles of radioresistant lung cancer cell line established by fractionated ionizing radiation in vitro. *Chin. Med. J.*, 121(18):1830–1837, Sep 2008.

[104] K. A. Arkhipova, A. N. Sheyderman, K. K. Laktionov, V. V. Mochalnikova, and I. B. Zborovskaya. Simultaneous expression of flotillin-1, flotillin-2, stomatin and caveolin-1 in non-small cell lung cancer and soft tissue sarcomas. *BMC Cancer*, 14:100, 2014.

[105] S. Keshava, S. Sahoo, T. A. Tucker, S. Idell, L. V. Rao, and U. R. Pendurthi. Endothelial cell protein C receptor opposes mesothelioma growth driven by tissue factor. *Cancer Res.*, 73(13):3963–3973, Jul 2013.

[106] C. P. El-Haibi, P. Sharma, R. Singh, P. Gupta, D. D. Taub, S. Singh, and J. W. Lillard. Differential G protein subunit expression by prostate cancer cells and their interaction with CXCR5. *Mol. Cancer*, 12:64, 2013.

[107] H. H. Zhang, Z. Y. Zhang, C. L. Che, Y. F. Mei, and Y. Z. Shi. Array analysis for potential biomarker of gemcitabine identification in non-small cell lung cancer cell lines. *Int J Clin Exp Pathol*, 6(9):1734–1746, 2013.

[108] Y. Wang, Y. Zhou, K. Szabo, C. R. Haft, and J. Trejo. Down-regulation of protease-activated receptor-1 is regulated by sorting nexin 1. *Mol. Biol. Cell*, 13(6):1965–1976, Jun 2002.

[109] Y. Nishimura, S. Takiguchi, S. Ito, and K. Itoh. Evidence that depletion of the sorting nexin 1 by siRNA promotes HGF-induced MET endocytosis and MET phosphorylation in a gefitinib-resistant human lung cancer cell line. *Int. J. Oncol.*, 44(2):412–426, Feb 2014.

[110] M. R. Dores, B. Chen, H. Lin, U. J. Soh, M. M. Paing, W. A. Montagne, T. Meerloo, and J. Trejo. ALIX binds a YPX(3)L motif of the GPCR PAR1 and mediates ubiquitin-independent ESCRT-III/MVB sorting. *J. Cell Biol.*, 197(3):407–419, Apr 2012.

[111] S. G. Liu, S. H. Yuan, H. Y. Wu, C. S. Huang, and J. Liu. The programmed cell death 6 interacting protein insertion/deletion polymorphism is associated with non-small cell lung cancer risk in a Chinese Han population. *Tumour Biol.*, 35(9):8679–8683, Sep 2014.

[112] S. Yasuda, T. Hasegawa, T. Hosono, M. Satoh, K. Watanabe, K. Ono, S. Shimizu, T. Hayakawa, T. Yamaguchi, K. Suzuki, and Y. Sato. AW551984: a novel regulator of cardiomyogenesis in pluripotent embryonic cells. *Biochem. J.*, 437(2):345–355, Jul 2011.

[113] B. T. Layden, M. Newman, F. Chen, A. Fisher, and W. L. Lowe. G protein coupled receptors in embryonic stem cells: a role for Gs-alpha signaling. *PLoS ONE*, 5(2):e9105, 2010.

[114] J. Sainz, F. Garcia-Alcalde, A. Blanco, and A. Concha. Genome-wide gene expression analysis in mouse embryonic stem cells. *Int. J. Dev. Biol.*, 55(10-12):995–1006, 2011.

[115] I. Nozaki, T. Tsuji, O. Iijima, Y. Ohmura, A. Andou, M. Miyazaki, N. Shimizu, and M. Namba. Reduced expression of REIC/Dkk-3 gene in non-small cell lung cancer. *Int. J. Oncol.*, 19(1):117–121, Jul 2001.

[116] T. Tsuji, I. Nozaki, M. Miyazaki, M. Sakaguchi, H. Pu, Y. Hamazaki, O. Iijima, and M. Namba. Antiproliferative activity of REIC/Dkk-3 and its significant down-regulation in non-small-cell lung carcinomas. *Biochem. Biophys. Res. Commun.*, 289(1):257–263, Nov 2001.

[117] K. Kobayashi, M. Ouchida, T. Tsuji, H. Hanafusa, M. Miyazaki, M. Namba, N. Shimizu, and K. Shimizu. Reduced expression of the REIC/Dkk-3 gene by promoter-hypermethylation in human tumor cells. *Gene*, 282(1-2):151–158, Jan 2002.

[118] J. D. Licchesi, W. H. Westra, C. M. Hooker, E. O. Machida, S. B. Baylin, and J. G. Herman. Epigenetic alteration of Wnt pathway antagonists in progressive glandular neoplasia of the lung. *Carcinogenesis*, 29(5):895–904, May 2008.

[119] K. Shien, N. Tanaka, M. Watanabe, J. Soh, M. Sakaguchi, K. Matsuo, H. Yamamoto, M. Furukawa, H. Asano, K. Tsukuda, Y. Nasu, N. H. Huh, S. Miyoshi, H. Kumon, and S. Toyooka. Anti-cancer effects of REIC/Dkk-3-encoding adenoviral vector for the treatment of non-small cell lung cancer. *PLoS ONE*, 9(2):e87900, 2014.

[120] K. Kataoka, M. Sakaguchi, K. P. Li, C. Taketa, K. Yamamoto, G. Du, H. Funahashi, H. Murata, and N. H. Huh. Internalization of REIC/Dkk-3 protein by induced pluripotent stem cell-derived embryoid bodies and extra-embryonic tissues. *Int. J. Mol. Med.*, 26(6):853–859, Dec 2010.

[121] E. Karamariti, A. Margariti, B. Winkler, X. Wang, X. Hong, D. Baban, J. Ragoussis, Y. Huang, J. D. Han, M. M. Wong, C. M. Sag, A. M. Shah, Y. Hu, and Q. Xu. Smooth muscle cells differentiated from reprogrammed embryonic lung fibroblasts through DKK3 signaling are potent for tissue engineering of vascular grafts. *Circ. Res.*, 112(11):1433–1443, May 2013.

[122] K. Bharti, M. Gasper, J. Ou, M. Brucato, K. Clore-Gronenborn, J. Pickel, and H. Arnheiter. A regulatory loop involving PAX6, MITF, and WNT signaling controls retinal pigment epithelium development. *PLoS Genet.*, 8(7):e1002757, Jul 2012.

[123] E. Lujan, S. Chanda, H. Ahlenius, T. C. Sudhof, and M. Wernig. Direct conversion of mouse fibroblasts to self-renewing, tripotent neural precursor cells. *Proc. Natl. Acad. Sci. U.S.A.*, 109(7):2527–2532, Feb 2012.

[124] T. Fukui, M. Kondo, G. Ito, O. Maeda, N. Sato, H. Yoshioka, K. Yokoi, Y. Ueda, K. Shimokata, and Y. Sekido. Transcriptional silencing of secreted frizzled related protein 1 (SFRP 1) by promoter hypermethylation in non-small-cell lung cancer. *Oncogene*, 24(41):6323–6327, Sep 2005.

[125] J. Zhu, Y. Wang, J. Duan, H. Bai, Z. Wang, L. Wei, J. Zhao, M. Zhuo, S. Wang, L. Yang, T. An, M. Wu, and J. Wang. DNA Methylation status of Wnt antagonist SFRP5 can predict the response to the EGFR-tyrosine kinase inhibitor therapy in non-small cell lung cancer. *J. Exp. Clin. Cancer Res.*, 31:80, 2012.

[126] J. Ren, R. Wang, H. Song, G. Huang, and L. Chen. Secreted frizzled related protein 1 modulates taxane resistance of human lung adenocarcinoma. *Mol. Med.*, 20:164–178, 2014.

[127] Y. Zhang, R. Wang, H. Song, G. Huang, J. Yi, Y. Zheng, J. Wang, and L. Chen. Methylation of multiple genes as a candidate biomarker in non-small cell lung cancer. *Cancer Lett.*, 303(1):21–28, Apr 2011.

[128] Y. W. Zhang, Y. F. Miao, J. Yi, J. Geng, R. Wang, and L. B. Chen. Transcriptional inactivation of secreted frizzled-related protein 1 by promoter hypermethylation as a potential biomarker for non-small cell lung cancer. *Neoplasma*, 57(3):228–233, 2010.

[129] J. Ren, R. Wang, G. Huang, H. Song, Y. Chen, and L. Chen. sFRP1 inhibits epithelial-mesenchymal transition in A549 human lung adenocarcinoma cell line. *Cancer Biother. Radiopharm.*, 28(7):565–571, Sep 2013.

[130] M. Fardilha, S. L. Esteves, L. Korrodi-Gregorio, A. P. Vintem, S. C. Domingues, S. Rebelo, N. Morrice, P. T. Cohen, O. A. da Cruz e Silva, and E. F. da Cruz e Silva. Identification of the human testis protein phosphatase 1 interactome. *Biochem. Pharmacol.*, 82(10):1403–1415, Nov 2011.

[131] S. Molina-Pinelo, I. Ferrer, C. Blanco-Aparicio, S. Peregrino, M. D. Pastor, J. Alvarez-Vega, R. Suarez, M. Verge, J. J. Marin, J. Hernandez-Losa, S. Ramon y Cajal, L. Paz-Ares, and A. Carnero. Down-regulation of spinophilin in lung tumours contributes to tumourigenesis. *J. Pathol.*, 225(1):73–82, Sep 2011.

[132] A. Bafico, A. Gazit, T. Pramila, P. W. Finch, A. Yaniv, and S. A. Aaronson. Interaction of frizzled related protein (FRP) with Wnt ligands and the frizzled receptor suggests alternative mechanisms for FRP inhibition of Wnt signaling. *J. Biol. Chem.*, 274(23):16180–16187, Jun 1999.

[133] D. T. Bravo, Y. L. Yang, K. Kuchenbecker, M. S. Hung, Z. Xu, D. M. Jablons, and L. You. Frizzled-8 receptor is activated by the Wnt-2 ligand in non-small cell lung cancer. *BMC Cancer*, 13:316, 2013.

[134] L. You, B. He, Z. Xu, K. Uematsu, J. Mazieres, I. Mikami, N. Reguart, T. W. Moody, J. Kitajewski, F. McCormick, and D. M. Jablons. Inhibition of Wnt-2-mediated signaling induces programmed cell death in non-small-cell lung cancer cells. *Oncogene*, 23(36):6170–6174, Aug 2004.

[135] D. J. Elzi, M. Song, K. Hakala, S. T. Weintraub, and Y. Shiio. Wnt antagonist SFRP1 functions as a secreted mediator of senescence. *Mol. Cell. Biol.*, 32(21):4388–4399, Nov 2012.

[136] X. Xu, P. L. Sun, J. Z. Li, S. Jheon, C. T. Lee, and J. H. Chung. Aberrant Wnt1/β-catenin expression is an independent poor prognostic marker of non-small cell lung cancer after surgery. *J Thorac Oncol*, 6(4):716–724, Apr 2011.

[137] C. L. Huang, D. Liu, S. Ishikawa, T. Nakashima, N. Nakashima, H. Yokomise, K. Kadota, and M. Ueno. Wnt1 overexpression promotes tumour progression

in non-small cell lung cancer. *Eur. J. Cancer*, 44(17):2680–2688, Nov 2008.

[138] T. Nakashima, D. Liu, J. Nakano, S. Ishikawa, H. Yokomise, M. Ueno, K. Kadota, and C. L. Huang. Wnt1 overexpression associated with tumor proliferation and a poor prognosis in non-small cell lung cancer patients. *Oncol. Rep.*, 19(1):203–209, Jan 2008.

[139] A. L. Silva, S. N. Dawson, M. J. Arends, K. Guttula, N. Hall, E. A. Cameron, T. H. Huang, J. D. Brenton, S. Tavare, M. Bienz, and A. E. Ibrahim. Boosting Wnt activity during colorectal cancer progression through selective hypermethylation of Wnt signaling antagonists. *BMC Cancer*, 14:891, 2014.

[140] Y. W. Kwon, Y. J. Chung, J. Kim, H. J. Lee, J. Park, T. Y. Roh, H. J. Cho, C. H. Yoon, B. K. Koo, and H. S. Kim. Comparative study of efficacy of dopaminergic neuron differentiation between embryonic stem cell and protein-based induced pluripotent stem cell. *PLoS ONE*, 9(1):e85736, 2014.

[141] J. Schemmer, M. J. Arauzo-Bravo, N. Haas, S. Schafer, S. N. Weber, A. Becker, D. Eckert, A. Zimmer, D. Nettersheim, and H. Schorle. Transcription factor TFAP2C regulates major programs required for murine fetal germ cell maintenance and haploinsufficiency predisposes to teratomas in male mice. *PLoS ONE*, 8(8):e71113, 2013.

[142] W. Chung, B. Kwabi-Addo, M. Ittmann, J. Jelinek, L. Shen, Y. Yu, and J. P. Issa. Identification of novel tumor markers in prostate, colon and breast cancer by unbiased methylation profiling. *PLoS ONE*, 3(4):e2079, 2008.

[143] D. S. Kim, M. J. Kim, J. Y. Lee, S. M. Lee, J. Y. Choi, G. S. Yoon, Y. K. Na, H. S. Hong, S. G. Kim, J. E. Choi, S. Y. Lee, and J. Y. Park. Epigenetic inactivation of Homeobox A5 gene in nonsmall cell lung cancer and its relationship with clinicopathological features. *Mol. Carcinog.*, 48(12):1109–1115, Dec 2009.

[144] M. Abe, J. Hamada, O. Takahashi, Y. Takahashi, M. Tada, M. Miyamoto, T. Morikawa, S. Kondo, and T. Moriuchi. Disordered expression of HOX genes in human non-small cell lung cancer. *Oncol. Rep.*, 15(4):797–802, Apr 2006.

[145] X. C. Wang, L. L. Tian, H. L. Wu, X. Y. Jiang, L. Q. Du, H. Zhang, Y. Y. Wang, H. Y. Wu, D. G. Li, Y. She, Q. F. Liu, F. Y. Fan, and A. M. Meng. Expression of miRNA-130a in nonsmall cell lung cancer. *Am. J. Med. Sci.*, 340(5):385–388, Nov 2010.

[146] X. H. Liu, K. H. Lu, K. M. Wang, M. Sun, E. B. Zhang, J. S. Yang, D. D. Yin, Z. L. Liu, J. Zhou, Z. J. Liu, W. De, and Z. X. Wang. MicroRNA-196a promotes non-small cell lung cancer cell proliferation and invasion through targeting HOXA5. *BMC Cancer*, 12:348, 2012.

[147] X. H. Liu, Z. L. Liu, M. Sun, J. Liu, Z. X. Wang, and W. De. The long non-coding RNA HOTAIR indicates a poor prognosis and promotes metastasis in non-small cell lung cancer. *BMC Cancer*, 13:464, 2013.

[148] T. Mohsen-Kanson, A. L. Hafner, B. Wdziekonski, Y. Takashima, P. Villageois, A. Carriere, M. Svensson, C. Bagnis, B. Chignon-Sicard, P. A. Svensson, L. Casteilla, A. Smith, and C. Dani. Differentiation of human induced pluripotent stem cells into brown and white adipocytes: role of Pax3. *Stem Cells*, 32(6):1459–1467, Jun 2014.

[149] K. L. Ostrow, M. O. Hoque, M. Loyo, M. Brait, A. Greenberg, J. M. Siegfried, J. R. Grandis, A. Gaither Davis, W. L. Bigbee, W. Rom, and D. Sidransky. Molecular analysis of plasma DNA for the early detection of lung cancer by quantitative methylation-specific PCR. *Clin. Cancer Res.*, 16(13):3463–3472, Jul 2010.

[150] S. Leng, K. Do, C. M. Yingling, M. A. Picchi, H. J. Wolf, T. C. Kennedy, W. J. Feser, A. E. Baron, W. A. Franklin, M. V. Brock, J. G. Herman, S. B. Baylin, T. Byers, C. A. Stidley, and S. A. Belinsky. Defining a gene promoter methylation signature in sputum for lung cancer risk assessment. *Clin. Cancer Res.*, 18(12):3387–3395, Jun 2012.

[151] J. C. Sporn, G. Kustatscher, T. Hothorn, M. Collado, M. Serrano, T. Muley, P. Schnabel, and A. G. Ladurner. Histone macroH2A isoforms predict the risk of lung cancer recurrence. *Oncogene*, 28(38):3423–3428, Sep 2009.

[152] A. Gaspar-Maia, Z. A. Qadeer, D. Hasson, K. Ratnakumar, N. A. Leu, G. Leroy, S. Liu, C. Costanzi, D. Valle-Garcia, C. Schaniel, I. Lemischka, B. Garcia, J. R. Pehrson, and E. Bernstein. MacroH2A histone variants act as a barrier upon reprogramming towards pluripotency. *Nat Commun*, 4:1565, 2013.

[153] M. R. Morris, C. J. Ricketts, D. Gentle, F. McRonald, N. Carli, H. Khalili, M. Brown, T. Kishida, M. Yao, R. E. Banks, N. Clarke, F. Latif, and E. R. Maher. Genome-wide methylation analysis identifies epigenetically inactivated candidate tumour suppressor genes in renal cell carcinoma. *Oncogene*, 30(12):1390–1401, Mar 2011.

[154] K. Tamm-Rosenstein, J. Simm, M. Suhorutshenko, A. Salumets, and M. Metsis. Changes in the transcriptome of the human endometrial Ishikawa cancer cell line induced by estrogen, progesterone, tamoxifen, and mifepristone (RU486) as detected by RNA-sequencing. *PLoS ONE*, 8(7):e68907, 2013.

[155] Y. W. Chang, S. C. Chen, E. C. Cheng, Y. P. Ko, Y. C. Lin, Y. R. Kao, Y. G. Tsay, P. C. Yang, C. W. Wu, and S. R. Roffler. CD13 (aminopeptidase N) can associate with tumor-associated antigen L6 and enhance

the motility of human lung cancer cells. *Int. J. Cancer*, 116(2):243–252, Aug 2005.

[156] D. C. Seo, J. M. Sung, H. J. Cho, H. Yi, K. H. Seo, I. S. Choi, D. K. Kim, J. S. Kim, A. El-Aty AM, and H. C. Shin. Gene expression profiling of cancer stem cell in human lung adenocarcinoma A549 cells. *Mol. Cancer*, 6:75, 2007.

[157] S. I. Choi, S. Y. Kim, J. Lee, E. W. Cho, and I. G. Kim. TM4SF4 overexpression in radiation-resistant lung carcinoma cells activates IGF1R via elevation of IGF1. *Oncotarget*, 5(20):9823–9837, Oct 2014.

[158] S. Bae, S. H. Shim, C. W. Park, H. K. Son, H. J. Lee, J. Y. Son, C. Jeon, and H. Kim. Combined omics analysis identifies transmembrane 4 L6 family member 1 as a surface protein marker specific to human mesenchymal stem cells. *Stem Cells Dev.*, 20(2):197–203, Feb 2011.

[159] M. L. Mo, J. Okamoto, Z. Chen, T. Hirata, I. Mikami, G. Bosco-Clement, H. Li, H. M. Zhou, D. M. Jablons, and B. He. Down-regulation of SIX3 is associated with clinical outcome in lung adenocarcinoma. *PLoS ONE*, 8(8):e71816, 2013.

[160] J. T. Amelung, R. Buhrens, M. Beshay, and M. A. Reymond. Key genes in lung cancer translational research: a meta-analysis. *Pathobiology*, 77(2):53–63, 2010.

[161] B. Bartling, G. Rehbein, W. D. Schmitt, H. S. Hofmann, R. E. Silber, and A. Simm. S100A2-S100P expression profile and diagnosis of non-small cell lung carcinoma: impairment by advanced tumour stages and neoadjuvant chemotherapy. *Eur. J. Cancer*, 43(13):1935–1943, Sep 2007.

[162] D. Mahalingam, C. M. Kong, J. Lai, L. L. Tay, H. Yang, and X. Wang. Reversal of aberrant cancer methylome and transcriptome upon direct reprogramming of lung cancer cells. *Sci Rep*, 2:592, 2012.

[163] A. Agathanggelou, I. Bieche, J. Ahmed-Choudhury, B. Nicke, R. Dammann, S. Baksh, B. Gao, J. D. Minna, J. Downward, E. R. Maher, and F. Latif. Identification of novel gene expression targets for the Ras association domain family 1 (RASSF1A) tumor suppressor gene in non-small cell lung cancer and neuroblastoma. *Cancer Res.*, 63(17):5344–5351, Sep 2003.

[164] F. M. Roversi, M. R. Lopes, J. A. Machado-Neto, A. L. Longhini, A. d. a. S. Duarte, M. O. Baratti, B. Palodetto, F. A. Corrocher, F. V. Pericole, P. d. e. M. Campos, P. Favaro, F. Traina, and S. T. Saad. Serine protease inhibitor kunitz-type 2 is downregulated in myelodysplastic syndromes and modulates cell-cell adhesion. *Stem Cells Dev.*, 23(10):1109–1120, May 2014.

[165] Z. Wang, R. Dao, L. Bao, Y. Dong, H. Wang, P. Han, Y. Yue, and H. Yu. Epigenetic reprogramming of human lung cancer cells with the extract of bovine parthenogenetic oocytes. *J. Cell. Mol. Med.*, 18(9):1807–1815, Sep 2014.

[166] X. Liu, L. Su, and X. Liu. Loss of CDH1 up-regulates epidermal growth factor receptor via phosphorylation of YBX1 in non-small cell lung cancer cells. *FEBS Lett.*, 587(24):3995–4000, Dec 2013.

[167] C. Wu, W. Zhu, J. Qian, S. He, C. Wu, Y. Chen, and Y. Shu. WT1 promotes invasion of NSCLC via suppression of CDH1. *J Thorac Oncol*, 8(9):1163–1169, Sep 2013.

[168] P. W. Xu, H. Y. Xu, X. N. Liu, C. Y. Zhang, C. Tan, C. M. Chen, H. Zhang, and Y. T. Jin. Aberrant promoter methylation of cell adhesion-related genes associated with clinicopathologic features in non-small cell lung cancer in China. *Cancer Biomark*, 13(2):115–122, 2013.

[169] M. Zhang, M. H. Chan, W. J. Tu, L. R. He, C. M. Lee, and M. He. Using the theory of coevolution to predict protein-protein interactions in non-small cell lung cancer. *Chin J Cancer*, 32(2):91–98, Feb 2013.

[170] F. D'Anselmi, M. G. Masiello, A. Cucina, S. Proietti, S. Dinicola, A. Pasqualato, G. Ricci, G. Dobrowolny, A. Catizone, A. Palombo, and M. Bizzarri. Microenvironment promotes tumor cell reprogramming in human breast cancer cell lines. *PLoS ONE*, 8(12):e83770, 2013.

[171] S. C. Brown, S. A. Bronson, and M. A. Holubec. Improving a hospital pharmacy externship program. *Am J Hosp Pharm*, 47(6):1364–1369, Jun 1990.

[172] U. G. Sathyanarayana, S. Toyooka, A. Padar, T. Takahashi, E. Brambilla, J. D. Minna, and A. F. Gazdar. Epigenetic inactivation of laminin-5-encoding genes in lung cancers. *Clin. Cancer Res.*, 9(7):2665–2672, Jul 2003.

[173] R. Manda, T. Kohno, T. Niki, T. Yamada, S. Takenoshita, H. Kuwano, and J. Yokota. Differential expression of the LAMB3 and LAMC2 genes between small cell and non-small cell lung carcinomas. *Biochem. Biophys. Res. Commun.*, 275(2):440–445, Aug 2000.

[174] T. Xiao, W. Ying, L. Li, Z. Hu, Y. Ma, L. Jiao, J. Ma, Y. Cai, D. Lin, S. Guo, N. Han, X. Di, M. Li, D. Zhang, K. Su, J. Yuan, H. Zheng, M. Gao, J. He, S. Shi, W. Li, N. Xu, H. Zhang, Y. Liu, K. Zhang, Y. Gao, X. Qian, and S. Cheng. An approach to studying lung cancer-related proteins in human blood. *Mol. Cell Proteomics*, 4(10):1480–1486, Oct 2005.

[175] Z. Liu, R. Jiang, S. Yuan, N. Wang, Y. Feng, G. Hu, X. Zhu, K. Huang, J. Ma, G. Xu, Q. Liu, Z. Xue, and G. Fan. Integrated analysis of DNA methylation and RNA transcriptome during in vitro differentiation of human pluripotent stem cells into retinal pigment epithelial cells. *PLoS ONE*, 9(3):e91416, 2014.

[176] D. Barh, N. Jain, S. Tiwari, J. K. Field, E. Padin-Iruegas, A. Ruibal, R. Lopez, M. Herranz, A. Bhattacharya, L. Juneja, C. Viero, A. Silva, A. Miyoshi, A. Kumar, K. Blum, V. Azevedo, P. Ghosh, and T. Liloglou. A novel in silico reverse-transcriptomics-based identification and blood-based validation of a panel of sub-type specific biomarkers in lung cancer. *BMC Genomics*, 14 Suppl 6:S5, 2013.

[177] M. Scrima, C. De Marco, F. Fabiani, R. Franco, G. Pirozzi, G. Rocco, M. Ravo, A. Weisz, P. Zoppoli, M. Ceccarelli, G. Botti, D. Malanga, and G. Viglietto. Signaling networks associated with AKT activation in non-small cell lung cancer (NSCLC): new insights on the role of phosphatydil-inositol-3 kinase. *PLoS ONE*, 7(2):e30427, 2012.

[178] Y. Zhang, T. Ma, S. Yang, M. Xia, J. Xu, H. An, Y. Yang, and S. Li. High-mobility group A1 proteins enhance the expression of the oncogenic miR-222 in lung cancer cells. *Mol. Cell. Biochem.*, 357(1-2):363–371, Nov 2011.

[179] J. Hillion, L. J. Wood, M. Mukherjee, R. Bhattacharya, F. Di Cello, J. Kowalski, O. Elbahloul, J. Segal, J. Poirier, C. M. Rudin, S. Dhara, A. Belton, B. Joseph, S. Zucker, and L. M. Resar. Upregulation of MMP-2 by HMGA1 promotes transformation in undifferentiated, large-cell lung cancer. *Mol. Cancer Res.*, 7(11):1803–1812, Nov 2009.

[180] F. Di Cello, J. Hillion, A. Hristov, L. J. Wood, M. Mukherjee, A. Schuldenfrei, J. Kowalski, R. Bhattacharya, R. Ashfaq, and L. M. Resar. HMGA2 participates in transformation in human lung cancer. *Mol. Cancer Res.*, 6(5):743–750, May 2008.

[181] V. K. Sarhadi, H. Wikman, K. Salmenkivi, E. Kuosma, T. Sioris, J. Salo, A. Karjalainen, S. Knuutila, and S. Anttila. Increased expression of high mobility group A proteins in lung cancer. *J. Pathol.*, 209(2):206–212, Jun 2006.

[182] E. Kettunen, S. Anttila, J. K. Seppanen, A. Karjalainen, H. Edgren, I. Lindstrom, R. Salovaara, A. M. Nissen, J. Salo, K. Mattson, J. Hollmen, S. Knuutila, and H. Wikman. Differentially expressed genes in nonsmall cell lung cancer: expression profiling of cancer-related genes in squamous cell lung cancer. *Cancer Genet. Cytogenet.*, 149(2):98–106, Mar 2004.

[183] D. E. Suzuki, A. M. Nakahata, and O. K. Okamoto. Knockdown of E2F2 inhibits tumorigenicity, but preserves stemness of human embryonic stem cells. *Stem Cells Dev.*, 23(11):1266–1274, Jun 2014.

[184] S. N. Shah, C. Kerr, L. Cope, E. Zambidis, C. Liu, J. Hillion, A. Belton, D. L. Huso, and L. M. Resar. HMGA1 reprograms somatic cells into pluripotent stem cells by inducing stem cell transcriptional networks. *PLoS ONE*, 7(11):e48533, 2012.

[185] A. R. Pandiri, R. C. Sills, V. Ziglioli, T. V. Ton, H. H. Hong, S. A. Lahousse, K. E. Gerrish, S. S. Auerbach, K. R. Shockley, P. R. Bushel, S. D. Peddada, and M. J. Hoenerhoff. Differential transcriptomic analysis of spontaneous lung tumors in B6C3F1 mice: comparison to human non-small cell lung cancer. *Toxicol Pathol*, 40(8):1141–1159, Dec 2012.

[186] A. Benzinger, N. Muster, H. B. Koch, J. R. Yates, and H. Hermeking. Targeted proteomic analysis of 14-3-3 sigma, a p53 effector commonly silenced in cancer. *Mol. Cell Proteomics*, 4(6):785–795, Jun 2005.

[187] M. Inoue, K. Hiyama, K. Nakabayashi, E. Morii, M. Minami, N. Sawabata, Y. Shintani, T. Nakagiri, Y. Susaki, J. Maeda, M. Higashiyama, J. Okami, Y. Yoshida, J. Ding, Y. Otomo, and M. Okumura. An accurate and rapid detection of lymph node metastasis in non-small cell lung cancer patients based on one-step nucleic acid amplification assay. *Lung Cancer*, 78(3):212–218, Dec 2012.

[188] V. M. Radhakrishnan, T. J. Jensen, H. Cui, B. W. Futscher, and J. D. Martinez. Hypomethylation of the 14-3-3σ promoter leads to increased expression in non-small cell lung cancer. *Genes Chromosomes Cancer*, 50(10):830–836, Oct 2011.

[189] O. H. Minchenko, T. Ogura, I. L. Opentanova, D. O. Minchenko, A. Ochiai, J. Caro, S. V. Komisarenko, and H. Esumi. 6-Phosphofructo-2-kinase/fructose-2,6-bisphosphatase gene family overexpression in human lung tumor. *Ukr. Biokhim. Zh.*, 77(6):46–50, 2005.

[190] A. C. Klarer, J. O'Neal, Y. Imbert-Fernandez, A. Clem, S. R. Ellis, J. Clark, B. Clem, J. Chesney, and S. Telang. Inhibition of 6-phosphofructo-2-kinase (PFKFB3) induces autophagy as a survival mechanism. *Cancer Metab*, 2(1):2, 2014.

[191] Y. Arimura, J. Ashitani, S. Yanagi, M. Tokojima, K. Abe, H. Mukae, and M. Nakazato. Elevated serum beta-defensins concentrations in patients with lung cancer. *Anticancer Res.*, 24(6):4051–4057, 2004.

[192] A. L. McDoniels-Silvers, C. F. Nimri, G. D. Stoner, R. A. Lubet, and M. You. Differential gene expression in human lung adenocarcinomas and squamous cell carcinomas. *Clin. Cancer Res.*, 8(4):1127–1138, Apr 2002.

[193] X. J. Li and C. N. Qian. Serglycin in human cancers. *Chin J Cancer*, 30(9):585–589, Sep 2011.

[194] B. P. Schick, H. C. Ho, K. C. Brodbeck, C. W. Wrigley, and J. Klimas. Serglycin proteoglycan expression and synthesis in embryonic stem cells. *Biochim. Biophys. Acta*, 1593(2-3):259–267, Feb 2003.

[195] B. P. Schick, J. F. Gradowski, and J. D. San Antonio. Synthesis, secretion, and subcellular localization of serglycin proteoglycan in human endothelial cells. *Blood*, 97(2):449–458, Jan 2001.

[196] T. Tian, J. Hao, A. Xu, J. Hao, C. Luo, C. Liu, L. Huang, X. Xiao, and D. He. Determination of metastasis-associated proteins in non-small cell lung cancer by comparative proteomic analysis. *Cancer Sci.*, 98(8):1265–1274, Aug 2007.

[197] M. Kusakabe, T. Kutomi, K. Watanabe, N. Emoto, N. Aki, H. Kage, E. Hamano, H. Kitagawa, T. Nagase, A. Sano, Y. Yoshida, T. Fukami, T. Murakawa, J. Nakajima, S. Takamoto, S. Ota, M. Fukayama, Y. Yatomi, N. Ohishi, and D. Takai. Identification of G0S2 as a gene frequently methylated in squamous lung cancer by combination of in silico and experimental approaches. *Int. J. Cancer*, 126(8):1895–1902, Apr 2010.

[198] K. S. Orr, Z. Shi, W. M. Brown, K. A. O'Hagan, T. R. Lappin, P. Maxwell, and M. J. Percy. Potential prognostic marker ubiquitin carboxyl-terminal hydrolase-L1 does not predict patient survival in non-small cell lung carcinoma. *J. Exp. Clin. Cancer Res.*, 30:79, 2011.

[199] F. Brichory, D. Beer, F. Le Naour, T. Giordano, and S. Hanash. Proteomics-based identification of protein gene product 9.5 as a tumor antigen that induces a humoral immune response in lung cancer. *Cancer Res.*, 61(21):7908–7912, Nov 2001.

[200] M. Patel, L. Lu, D. S. Zander, L. Sreerama, D. Coco, and J. S. Moreb. ALDH1A1 and ALDH3A1 expression in lung cancers: correlation with histologic type and potential precursors. *Lung Cancer*, 59(3):340–349, Mar 2008.

[201] B. Kim, H. J. Lee, H. Y. Choi, Y. Shin, S. Nam, G. Seo, D. S. Son, J. Jo, J. Kim, J. Lee, J. Kim, K. Kim, and S. Lee. Clinical validity of the lung cancer biomarkers identified by bioinformatics analysis of public expression data. *Cancer Res.*, 67(15):7431–7438, Aug 2007.

[202] M. D. Pastor, A. Nogal, S. Molina-Pinelo, R. Melendez, A. Salinas, M. Gonzalez De la Pena, J. Martin-Juan, J. Corral, R. Garcia-Carbonero, A. Carnero, and L. Paz-Ares. Identification of proteomic signatures associated with lung cancer and COPD. *J Proteomics*, 89:227–237, Aug 2013.

[203] Y. Zhang, R. Xu, G. Li, X. Xie, J. Long, and H. Wang. Loss of expression of the differentially expressed in adenocarcinoma of the lung (DAL-1) protein is associated with metastasis of non-small cell lung carcinoma cells. *Tumour Biol.*, 33(6):1915–1925, Dec 2012.

[204] D. Haase, M. Meister, T. Muley, J. Hess, S. Teurich, P. Schnabel, B. Hartenstein, and P. Angel. FRMD3, a novel putative tumour suppressor in NSCLC. *Oncogene*, 26(30):4464–4468, Jun 2007.

[205] G. Heller, K. M. Fong, L. Girard, S. Seidl, A. End-Pfutzenreuter, G. Lang, A. F. Gazdar, J. D. Minna, C. C. Zielinski, and S. Zochbauer-Muller. Expression and methylation pattern of TSLC1 cascade genes in lung carcinomas. *Oncogene*, 25(6):959–968, Feb 2006.

[206] S. Kikuchi, D. Yamada, T. Fukami, M. Masuda, M. Sakurai-Yageta, Y. N. Williams, T. Maruyama, H. Asamura, Y. Matsuno, M. Onizuka, and Y. Murakami. Promoter methylation of DAL-1/4.1B predicts poor prognosis in non-small cell lung cancer. *Clin. Cancer Res.*, 11(8):2954–2961, Apr 2005.

[207] H. J. van de Velde, N. H. Senden, T. A. Roskams, J. L. Broers, F. C. Ramaekers, A. J. Roebroek, and W. J. Van de Ven. NSP-encoded reticulons are neuroendocrine markers of a novel category in human lung cancer diagnosis. *Cancer Res.*, 54(17):4769–4776, Sep 1994.

[208] M. Woenckhaus, L. Klein-Hitpass, U. Grepmeier, J. Merk, M. Pfeifer, P. Wild, M. Bettstetter, P. Wuensch, H. Blaszyk, A. Hartmann, F. Hofstaedter, and W. Dietmaier. Smoking and cancer-related gene expression in bronchial epithelium and non-small-cell lung cancers. *J. Pathol.*, 210(2):192–204, Oct 2006.

[209] N. Senden, I. Linnoila, E. Timmer, H. van de Velde, A. Roebroek, W. Van de Ven, J. Broers, and F. Ramaekers. Neuroendocrine-specific protein (NSP)-reticulons as independent markers for non-small cell lung cancer with neuroendocrine differentiation. An in vitro histochemical study. *Histochem. Cell Biol.*, 108(2):155–165, Aug 1997.

[210] J. S?odkowska. The value of immunohistochemical identification of neuroendocrine differentiation in non small cell lung carcinoma. *Rocz. Akad. Med. Bialymst.*, 42 Suppl 1:23–27, 1997.

[211] S. Tagami, Y. Eguchi, M. Kinoshita, M. Takeda, and Y. Tsujimoto. A novel protein, RTN-XS, interacts with both Bcl-XL and Bcl-2 on endoplasmic reticulum and reduces their anti-apoptotic activity. *Oncogene*, 19(50):5736–5746, Nov 2000.

[212] Y. Wang, D. Chen, H. Qian, Y. S. Tsai, S. Shao, Q. Liu, D. Dominguez, and Z. Wang. The splicing factor RBM4 controls apoptosis, proliferation, and migration to suppress tumor progression. *Cancer Cell*, 26(3):374–389, Sep 2014.

[213] Y. Zhang, J. H. Wang, Q. Lu, and Y. J. Wang. Bag3 promotes resistance to apoptosis through Bcl-2 family members in non-small cell lung cancer. *Oncol. Rep.*, 27(1):109–113, Jan 2012.

[214] S. H. Leech, R. A. Olie, O. Gautschi, A. P. Simoes-Wust, S. Tschopp, R. Haner, J. Hall, R. A. Stahel, and U. Zangemeister-Wittke. Induction of apoptosis in lung-cancer cells following bcl-xL anti-sense treatment. *Int. J. Cancer*, 86(4):570–576, May 2000.

[215] D. Xiong, G. Li, K. Li, Q. Xu, Z. Pan, F. Ding, P. Vedell, P. Liu, P. Cui, X. Hua, H. Jiang, Y. Yin, Z. Zhu, X. Li, B. Zhang, D. Ma, Y. Wang, and M. You. Exome sequencing identifies MXRA5 as a novel cancer gene frequently mutated in non-small cell lung carcinoma from

Chinese patients. *Carcinogenesis*, 33(9):1797–1805, Sep 2012.

[216] K. A. Yoon, J. H. Park, J. Han, S. Park, G. K. Lee, J. Y. Han, J. I. Zo, J. Kim, J. E. Lee, A. Takahashi, M. Kubo, Y. Nakamura, and J. S. Lee. A genome-wide association study reveals susceptibility variants for non-small cell lung cancer in the Korean population. *Hum. Mol. Genet.*, 19(24):4948–4954, Dec 2010.

[217] M. M. Edwards, E. Mammadova-Bach, F. Alpy, A. Klein, W. L. Hicks, M. Roux, P. Simon-Assmann, R. S. Smith, G. Orend, J. Wu, N. S. Peachey, J. K. Naggert, O. Lefebvre, and P. M. Nishina. Mutations in Lama1 disrupt retinal vascular development and inner limiting membrane formation. *J. Biol. Chem.*, 285(10):7697–7711, Mar 2010.

[218] M. M. Edwards, D. S. McLeod, R. Grebe, C. Heng, O. Lefebvre, and G. A. Lutty. Lama1 mutations lead to vitreoretinal blood vessel formation, persistence of fetal vasculature, and epiretinal membrane formation in mice. *BMC Dev. Biol.*, 11:60, 2011.

[219] N. Ichikawa-Tomikawa, J. Ogawa, V. Douet, Z. Xu, Y. Kamikubo, T. Sakurai, S. Kohsaka, H. Chiba, N. Hattori, Y. Yamada, and E. Arikawa-Hirasawa. Laminin Î $\pm$ 1 is essential for mouse cerebellar development. *Matrix Biol.*, 31(1):17–28, Jan 2012.

[220] L. Xu and R. O. Hynes. GPR56 and TG2: possible roles in suppression of tumor growth by the microenvironment. *Cell Cycle*, 6(2):160–165, Jan 2007.

[221] N. Ke, R. Sundaram, G. Liu, J. Chionis, W. Fan, C. Rogers, T. Awad, M. Grifman, D. Yu, F. Wong-Staal, and Q. X. Li. Orphan G protein-coupled receptor GPR56 plays a role in cell transformation and tumorigenesis involving the cell adhesion pathway. *Mol. Cancer Ther.*, 6(6):1840–1850, Jun 2007.

[222] L. Yang, G. Chen, S. Mohanty, G. Scott, F. Fazal, A. Rahman, S. Begum, R. O. Hynes, and L. Xu. GPR56 Regulates VEGF production and angiogenesis during melanoma progression. *Cancer Res.*, 71(16):5558–5568, Aug 2011.

[223] R. Luo, S. J. Jeong, Z. Jin, N. Strokes, S. Li, and X. Piao. G protein-coupled receptor 56 and collagen III, a receptor-ligand pair, regulates cortical development and lamination. *Proc. Natl. Acad. Sci. U.S.A.*, 108(31):12925–12930, Aug 2011.

[224] L. Yang and L. Xu. GPR56 in cancer progression: current status and future perspective. *Future Oncol*, 8(4):431–440, Apr 2012.
